# The global distribution and risk prediction of Anaplasmataceae species: a systematic review and geospatial modelling analysis

**DOI:** 10.1016/j.ebiom.2025.105722

**Published:** 2025-04-23

**Authors:** Xiao-Bin Huang, Tian Tang, Jin-Jin Chen, Yuan-Yuan Zhang, Chen-Long Lv, Qiang Xu, Guo-Lin Wang, Ying Zhu, Yue-Hong Wei, Simon I. Hay, Li-Qun Fang, Wei Liu

**Affiliations:** a State Key Laboratory of Pathogen and Biosecurity, Academy of Military Medical Sciences, Beijing, 100071, China; b School of Public Health, Sun Yat-sen University, Guangzhou, 510080, China; c Department of Parasitic Disease and Endemic Disease Control and Prevention, Guangzhou Center for Disease Control and Prevention, Guangzhou, China; d Department of Health Metrics Sciences, School of Medicine, University of Washington, USA; e Institute for Health Metrics and Evaluation, University of Washington, USA; f Department of Epidemiology and Biostatistics, School of Public Health, Wuhan, China

**Keywords:** Anaplasmataceae species, Distribution, Risk prediction, Modelling analysis

## Abstract

**Background:**

The family Anaplasmataceae, reclassified under the order Rickettsiales, represents a highly complex group that poses an increasing global threat. However, their infection risk remains poorly understood. We aimed to map the diversity, distribution, and potential infection risk of Anaplasmataceae members.

**Methods:**

We searched PubMed, Web of Science, bioRvix, and MedRvix for published articles to extract data on the detection of Anaplasmatacea species in vectors, animals, and humans from 1910 to 2022. We mapped the richness and global distribution of identified Anaplasmatacea species. Machine learning algorithms were applied to determine the ecological and vector-related factors contributing to the occurrence of major Anaplasmatacea members and project their potential risk distributions.

**Findings:**

A total of 2605 studies meeting our inclusion criteria were used for data extraction. We identified 85 species of Anaplasmataceae family from 134 tick species, 312 wild animals, and 12 domestic animals. *Anaplasma phagocytophilum* had the widest range of vectors (97 species), followed by *Anaplasma marginale* (54 species), *Anaplasma bovis* (46 species), *Anaplasma ovis* (37 species), and *Anaplasma platys* (35 species). *Aanaplasmaphagocytophilum* was also detected in the widest range of wildlife (208 species), followed by *Ehrlichia chaffeensis* (46 species), *Candidatus* Neoehrlichia mikurensis (36 species), *Ehrlichia canis* (35 species), and *A. bovis* (32 species). In total, 52,315 human cases involving 15 Anaplasmataceae species were recorded, *A. phagocytophilum* and *E*. *chaffeensis* accounted for majority of human infections (66·5% and 32·4%, respectively). According to our modelling analysis, the geographic distribution of six major Anaplasmatacea species is primarily influenced by the projected habitat suitability index of tick vectors and climatic conditions. Among these, *A*. *phagocytophilum* presents the highest risk, with an estimated 3·97 billion individuals and 8·95 million km^2^ area potentially affected.

**Interpretation:**

The widespread distribution of Anaplasmataceae species emphasizes the need to enhance identification, surveillance, and diagnosis efforts in high-risk areas, particularly within low-income regions.

**Funding:**

The 10.13039/501100012166National Key Research and Development Program of China (2023YFC2605603) and the 10.13039/501100001809Natural Science Foundation of China (82330103).

**Research in context**

**Evidence before this study**

On Dec 31, 2022, we conducted a comprehensive literature search on PubMed, Web of Science, bioRvix, and MedRvix, to identify all studies concerning members of the family Anaplasmataceae since the inception of databases. The search used the following terms: (“Anaplasmataceae” OR “*Anaplasma*” OR “*Ehrlichia*” OR “*Neorickettsia*” OR “*Wolbachia*” OR “*Aegyptianella*” OR “*Candidatus* Neoehrlichia” OR “*Candidatus* Xenohaliotis” OR “*Candidatus* Allocryptoplasma” OR “*Candidatus* Mesenet” OR “*Candidatus* Neowolbachia” OR “*Candidatus* Xenolissocinum”) AND (“spatial” OR “distribution”), with no restrictions on language or publication date. Of 3730 studies identified, 873 focused on the detection of Anaplasmataceae members in animals, vectors, and/or humans; 2805 were related to experimental, genomic, or mechanism studies; 52 described the spatial distribution of Anaplasmataceae members. Among these 52 spatial distribution studies, 46 addressed the geospatial distribution of Anaplasmataceae members within specific countries or smaller regions, three covered multiple countries, and the remaining three presented meta-analyses of the global distribution of *Anaplasma phagocytophilum*. Notably, among these 52 spatial distribution studies reviewed, seven focused on ecological modelling analysis at the national scale, five used the Bayesian spatio-temporal models to predict the prevalence of *Anaplasma* or *Ehrlichia* species in the United States, incorporating climatic, geographic and socio-economic variables. Additionally, two studies separately applied logistic regression models to estimate the distribution of *Ehrlichia chaffeensis* and *A. phagocytophilum* in the United States based on ecological factors. To date, no study has provided a comprehensive quantification of the global risk associated with infections caused by Anaplasmataceae members.

**Added value of this study**

In this study, we conducted an exhaustive review of all published articles to extract data on the detection of Anaplasmataceae members in vectors, animals, and humans during the period from Jan 1, 1910 to Dec 31, 2022. A total of 85 species belonging to the Anaplasmataceae family were identified in 134 tick species, 312 wild animals, and 12 domestic animals. Among these, 15 species were determined to cause human infections. Among all 85 Anaplasmataceae members, *A. phagocytophilum* was detected in the largest number of both tick and animal species and was associated with the highest number of reported human cases. We conducted a comprehensive analysis of the global spatial distribution of reported locations for various arthropod vectors carrying Anaplasmataceae members, as well as 85 confirmed pathogens or endosymbionts. Using machine-learning models, we assessed the influence of potential environmental, ecoclimatic, biological, and socioeconomic factors on the spatial distributions of the six major Anaplasmataceae members. Additionally, we predicted areas and population sizes at potential risk of infection by these species. Our findings indicate that the habitat suitability index for vector ticks was the most significant contributor to this predicted effect, followed by climatic factors.

**Implications of all the available evidence**

This study provides a comprehensive and up-to-date picture of the global distributions of members of Anaplasmataceae family. The potential risk areas for six major Anaplasmataceae members are more extensive than previously reported, highlighting the necessity for enhanced surveillance of Anaplasmataceae infections. Overall, our findings may help guide the diagnosis, surveillance, and control of Anaplasmataceae species infections in potential high-risk areas and resource-limited countries.

## Introduction

The family Anaplasmataceae is a major group of obligate intracellular bacteria carried and potentially transmitted by arthropod vectors, mainly ticks. The recent reclassification of the family Anaplasmataceae within the order Rickettsiales has expanded to encompass all species of the Alphaproteobacteria presently contained in the genera *Anaplasma*, as well as genera *Ehrlichia*, *Wolbachia*, *Neorickettsia*, and *Candidatus* Neoehrlichia, etc., that accurately reflects their phylogenetic positions.^1^ The Anaplasmataceae members exhibit a wide distribution and can infect a diverse range of wild and domestic animals.^2^ They have been detected in a variety arthropod vectors, such as ticks, mosquitoes, fleas, lice, and mites, which may be potential vectors of Anaplasmataceae species.^3^, ^4^, ^5^, ^6^, ^7^ Despite being prevalent in natural environment, human infections with Anaplasmataceae species are currently under-monitored, with most cases reported in developed regions like Europe and the United States.^8^^,^^9^

Recent advancements in molecular technologies have facilitated the classification of Anaplasmataceae species, and led to the discovery of new members within this family.^10^^,^^11^ In recent years, new species, such as *Candidatus* Anaplasma cinensis, *Candidatus* Anaplasma boleense, and *Candidatus* Anaplasma gabonensis have been detected in vectors and animal hosts across developing countries in Africa and Asia.^12^, ^13^, ^14^ *Candidatus* Anaplasma sparouinense, a new human pathogen within the genus *Anaplasma*, has caused infections among humans living in rainforest of French Guiana.^11^ These findings called attention to the Anaplasmataceae species in diminishing human health.

Due to limited surveillance efforts, the prevalence of Anaplasmataceae species infections remains unclear.^15^ Previous meta-analyses have primarily focused on estimating the prevalence of specific species such as *A. phagocytophilum*, *Candidatus* Neoehrlichia mikurensis, and *Anaplasma capra* in vectors, animals, or humans. These studies have provided valuable insights into particular members of the Anaplasmataceae family within specific geographic regions.^2^^,^^15^, ^16^, ^17^, ^18^ However, there is currently a notable lack of systematic global-level analyses of Anaplasmataceae family following its reclassification, which has hindered niche modelling and risk-assessment for predicting undetected infections.

In this study, we conducted a comprehensive review of up-to-date data on Anaplasmataceae species from 1910 to 2022. Based on this data review, we performed a global mapping to determine the spatial distribution of these species in vectors, animals, and humans. By utilizing multiple machine-learning algorithms, we aimed to predict the ecological niches for major vectors and potential distribution of major Anaplasmataceae species.

## Methods

### Literature search

We conducted a systematic literature search on PubMed, Web of Science, MedRvix, and bioRvix databases for published studies or reports from January 1, 1910 to December 31, 2022 without any language restrictions. The protocol followed the Preferred Reporting Items for Systematic Reviews and Meta-Analyses (PRISMA) statement (PRISMA Checklist), and has been registered with PROSPERO, an international prospective register of systematic reviews (CRD42023477378) (further detailed in Appendix 1 Supplementary Table S1). The search included Anaplasmataceae species infections in vectors, animals, and humans, and clinical information about human cases using specific terms in the title and abstract: “Anaplasmataceae” OR “*Anaplasma*” OR “*Ehrlichia*” OR “*Neorickettsia*” OR “*Wolbachia*” OR “*Aegyptianella*” OR “*Candidatus* Neoehrlichia” OR “*Candidatus* Xenohaliotis” OR “*Candidatus* Allocryptoplasma” OR “*Candidatus* Mesenet” OR “*Candidatus* Neowolbachia” OR “*Candidatus* Xenolissocinum”, and the detailed search strategies are given in Appendix 1 Supplementary Table S2. Furthermore, the identical search terms were used to search GenBank database, thereby including all uploaded sequences and relevant studies that might have been missed during the literature review.

### Inclusion and exclusion criteria

The inclusion criteria for the study were established based on the Population, Exposure, Comparator, and Outcomes (PECO) framework. Specifically, the target populations include vectors, animal hosts, and human subjects. The diagnosis methods selected for this study include molecular detection, microscopy, and serological testing. The outcome comprises the detection results of Anaplasmataceae species in vectors, animals, and humans. Briefly, studies on Anaplasmataceae species detected in vectors, animals, or humans with clear test methods and occurrence locations were included. The following types of studies were excluded: reviews, symposia, oral presentations, opinion, and editorial articles; drug or vaccine trails; modelling analysis of the transmission dynamics of Anaplasmataceae species; transstadial transmission research in vectors; clinical trial of infection in animals; mechanistic research at the molecular level for Anaplasmataceae species; and studies where test methods or geographic information were not specified or ambiguously described. More details regarding the inclusion and exclusion criteria are given in Appendix 1 Supplementary Table S3. Infection with Anaplasmataceae species was determined based on the following criteria: (I) Detection through PCR or sequencing, isolation, or morphology identification under microscope examination in humans, vectors, or animals; (II) A four-fold increase or seroconversion in the titre of specific antibodies in blood sera from acute phase to convalescent phase in clinically diagnosed patients with Anaplasmataceae species infection (Appendix 1 Supplementary Table S4). To obtain a more comprehensive understanding of the distribution and prevalence of Anaplasmataceae species, positive serologic test results in animals were also included.

In addition, given that 16S rRNA is not an sufficiently informative marker for species identification within Anaplasmataceae family, and to further enhance the reliability of modelling analysis results, thereby we revalidated all included articles using molecular assays and excluded data derived from serological tests, pathogen isolation, microscopic examinations, and studies that merely referenced molecular tests without specifying gene fragments or relied solely on 16S rRNA fragments in the modelling analyses. Finally, data explicitly providing gene fragments for molecular testing and results utilizing 16S rRNA fragments in conjunction with other fragments (e.g., msp, dsb, etc.) were incorporated into the subsequent phase of modelling analyses.

### Data extraction

All relevant articles were exported to EndNote version 19 (Clarivate, Philadelphia, PA, USA). After removing duplicates, two authors (XBH and JJC) independently conducted the initial screening of titles and abstracts. They subsequently reviewed the full texts and extracted data from the eligible articles. Any disagreements were resolved through discussions with a third author (TT). For each eligible study, we extracted data on 15 variables, which were categorized into five groups: literation information, laboratory testing method, basic information of Anaplasmataceae members, testing information of Anaplasmataceae species in vectors and animals, and testing information of Anaplasmataceae species in humans (Appendix 1 Supplementary Table S5).

### Clinical spectrum of Anaplasmataceae species

For Anaplasmataceae species with ≥10 human cases that have reported symptoms, we extracted clinical data from the database. For each pathogenic Anaplasmataceae species, the frequency of each symptom was recorded. Given that common symptoms are more likely to be reported while rarer ones may be underreported or overlooked, unreported symptoms were estimated using two approaches: (I) recorded as zero, assuming the symptom is absent; (II) recorded with the same frequency as the minimum level among all the reported symptoms. By applying this strategy, we obtained a lower bound and an upper bound on the frequency of each symptom for each Anaplasmataceae member. Symptoms with a lower bound frequency of 30% or higher were defined as major symptoms.

### Assembling occurrence data and covariates

Ecoclimatic, environmental, and biological variables have been extensively used to predict the distribution of tick species and the risk of tick-borne diseases. To balance the global data availability and their priori importance in predicting the occurrence probabilities of Anaplasmataceae species, we have pre-confirmed potential associations between the occurrence risk of Anaplasmataceae species infection and these variables (Appendix 1 Supplementary Table S6). In this study, we included a total of 44 variables previously associated with tick-borne pathogen infections into our modelling analysis. These variables encompassed 19 ecoclimatic factors, 15 environmental factors, seven biological factors, and three socioeconomic factors (Appendix 1 Supplementary Tables S7‒S9).

We created a global grid-map with a resolution of 10 km∗10 km resolution using ArcGIS 10·7 (Esri Inc, Redlands, CA, USA) for subsequent modelling analyses and then associated each cell with ecological variables.^19^, ^20^, ^21^ The map in raster format was summarized into a processable data format at the study level based on the grid-map, and the R package “Raster” was used for resampling in order to change the resolution of raster data. Mean values for each variable were calculated for every cell within the grid map.

The occurrence of Anaplasmataceae members was classified as either point or polygon occurrences based on geocoordinates availability. Each occurrence was matched to the grid-map according to its coordinates. For a polygon occurrence record, we assigned the grid containing the centroid of the polygon as the occurrence grid. To minimize potential ecological fallacy, we first excluded all huge polygon occurrence records from ecological modelling due to insufficient resolution.^20^^,^^22^ For polygon occurrence records with an area no larger than 900 km^2^, we calculated the mean of each ecological variable across all grids within the polygon and associated the mean value with the occurrence grids, that was, the grids containing the centroid of the polygon.^20^^,^^23^^,^^24^

### Geo-localization of occurrence data for Anaplasmataceae species

For each Anaplasmataceae species, an occurrence was defined as one or more confirmed infection(s) at a unique location (geo-coordinates, polygons), regardless of the type of the hosts or the time of detection. To gain the locations of Anaplasmataceae species occurrences, we extracted the geo-coordinates from peer-reviewed articles reporting Anaplasmataceae species occurrences as point occurrence. When point information was not available, we extracted the smallest area unit (county, city or province) as polygon occurrence. For each polygon occurrence, the coordinates of its geographic centroid were queried from Google Maps. Results from serological testing in humans and animals were excluded from the modelling analyses due to potential cross-reactivity between Anaplasmataceae species. The occurrence data of details of the Anaplasmataceae species prior to and after molecular validation were detailed in Appendix 1 Supplementary Tables S10, S11. Finally, we identified six major members of the family Anaplasmataceae for the next step in the modelling analysis based on >80 occurrence sites within an area <900 km^2^, including *A. phagocytophilum*, *Candidatus* N. mikurensis, *Ehrlichia canis*, *Anaplasma platys*, *A. ovis*, and *Anaplasma marginale*, which have taken into account both the requirement of a minimum number of positive samples (e.g., 50–100) necessary for machine learning models and the data loss resulting from different area thresholds applied to polygon occurrence data.

### Niche modelling of the main tick species

In the first stage, a meta-analysis estimated the prevalence of six major species in tick species using molecular tests. Data from individual tick test were included instead of tick pools due to inconsistency of pooling methods. Ticks that met simultaneously met the criteria of greater than 100 test positives, greater than 2·0% positive rate, and greater than 2 reported articles as the dominant tick species of the major Anaplasmataceae species. Based on the results of meta-analysis, we identified eight dominant vector ticks of six Anaplasmataceae members (*Ixodes scapularis*, *Ixodes pacificus*, *Ixodes persulcatus*, *Ixodes ricinus*, *Haemaphysalis longicornis*, *Dermacentor marginatus*, *Rhipicephalus microplus*, and *Rhipicephalus sanguineus*) (Appendix 1 Supplementary Table S12; Appendix 4).

In the second stage, the habitat suitability index (HSI) was calculated for each of the eight tick species. For five previously studied species (*I. scapularis*, *I. pacificus*, *I*. *persulcatus*, *I*. *ricinus*, and *Ha. longicornis*), records were directly extracted to create tick distribution datasets.^24^^,^^25^ For the remaining three tick vectors (*D. marginatus*, *Rh. microplus*, and *Rh. sanguineus*), an extensive literature search was performed between 1980 and 2023 following the same protocol as that applied for the five tick species using Web of Science and PubMed. Geographic coordinates from included studies along with supplementary data from Global Biodiversity Information Facility database were merged by using the time of investigation (Appendix 1 Supplementary Table S13; Appendix 2). Then a case–control dataset was established for each specific tick,^21^^,^^26^ and a boosted regression trees (BRT) model was fitted to predict the occurrence probability for each tick species,^19^ by using environmental, ecoclimatic and biological variables as predictors.^27^ The HSI for each tick species was estimated as the average probability of each cell.^24^^,^^25^ The synthesized HSI for a cell with multiple tick species serving as potential carriers of a pathogen was established by selecting the maximum HSI value among these tick species. For example, in a cell where *A. phagocytophilum* had more than one vector tick species (*I. ricinus*, *I. scapularis*, *I. pacificus*, *I. persulcatus*, and *Ha. longicornis*), the highest HSI value among these vectors was chosen as the final HSI included in the niche modelling analysis of *A. phagocytophilum*. While for the other three major species, only a single major vector tick species was considered, i.e., *I. ricinus* for *Candidatus* N. mikurensis, *D. marginatus* for *A. ovis*, and *Rh. microplus* for *A. marginale*.

To avoid introducing additional ecological bias to the niche modelling of Anaplasmataceae species, we performed internal and external validation to the predicted HSI for each tick species. The internal validation was evaluated the robustness of the model itself by calculating the relative uncertainty of the predicted distribution for each tick species. The relative uncertainty for each grid was computed as the ratio of the 95% uncertainty intervals to the predicted suitability. External validation was performed by the occurrence grids after 2020, which used to calculate predictive accuracy, i.e., the probability these occurrence grids appeared within suitable habitats predicted by the models.

### Niche modelling of the major Anaplasmataceae species

To explore the relationship between the risk of major Anaplasmataceae species occurrences and ecoclimatic, environmental, biological, and socioeconomic variables, three machine-learning models, including BRT, random forest (RF) and least absolute shrinkage and selection operator (LASSO) logistic regression, were performed and compared to obtain the best predictive performance. The optimal method was determined through 10-fold block cross-validation base on Area Under Curve (AUC, area under the receiver operating characteristic curve that measures a classification model's performance by plotting the true positive rate against the false positive rate) metric to make final modelling predictions (Appendix 1 Supplementary Figure S22).

We constructed an assembly of 100 models for each of the three methods described above. The Relative Contributions (RCs) of all predictors and the AUCs for the test sets were averaged across the 100 models within the assembly to represent the final estimation results and predictive performance. We identified the optimal algorithm based on the highest average test AUC to map the potential distribution of Anaplasmataceae species. To determine high-risk areas as predicted by the model for each Anaplasmataceae species, we chose a cut-off value that maximizes the sum of sensitivity and specificity along the average ROC curve derived from the assembly of the chosen algorithm.^28^^,^^29^ Grids with an average predicted probability (across the 100 models) exceeding the cut-off value were classified as high-risk areas for the presence of the corresponding Anaplasmataceae species. For each Anaplasmataceae species, we quantified both the area and population size within these high-risk areas as predicted by the models. To evaluate the influence of different selection thresholds for polygon occurrences on niche modelling analysis, we conducted a comparative analysis of the performance using thresholds of 100 km^2^, 400 km^2^, and 900 km^2^. The comparative analysis indicated that 400 km^2^ was an appropriate threshold for polygon occurrences (Appendix 1 Supplementary Table S23).

### Role of the funding source

The funder of the study had no role in study design, data collection, data analysis, finding interpretation, or writing of the paper.

## Results

**Fig. 1:**
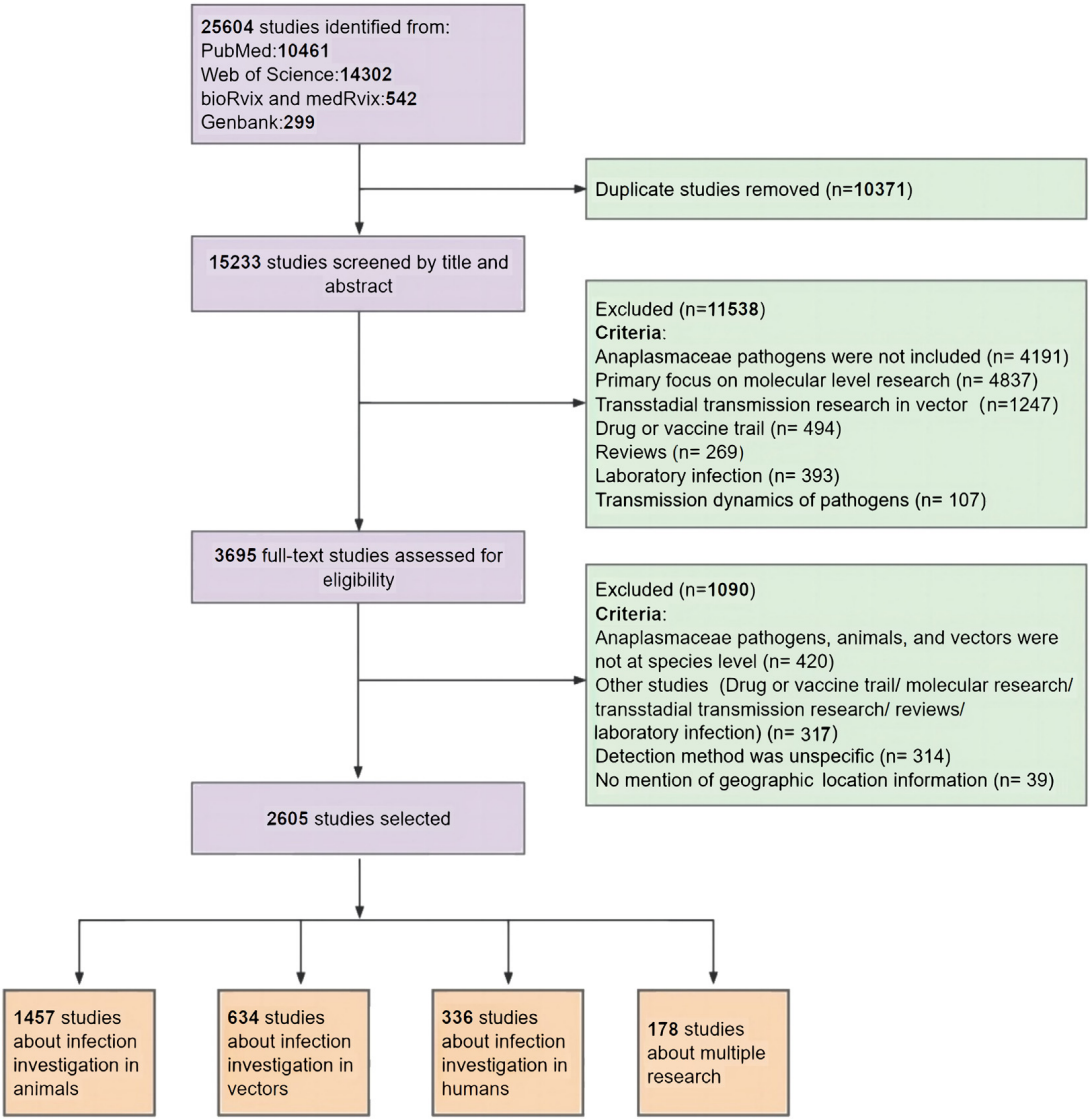
**The flow diagram of literature review**.

**Fig. 2:**
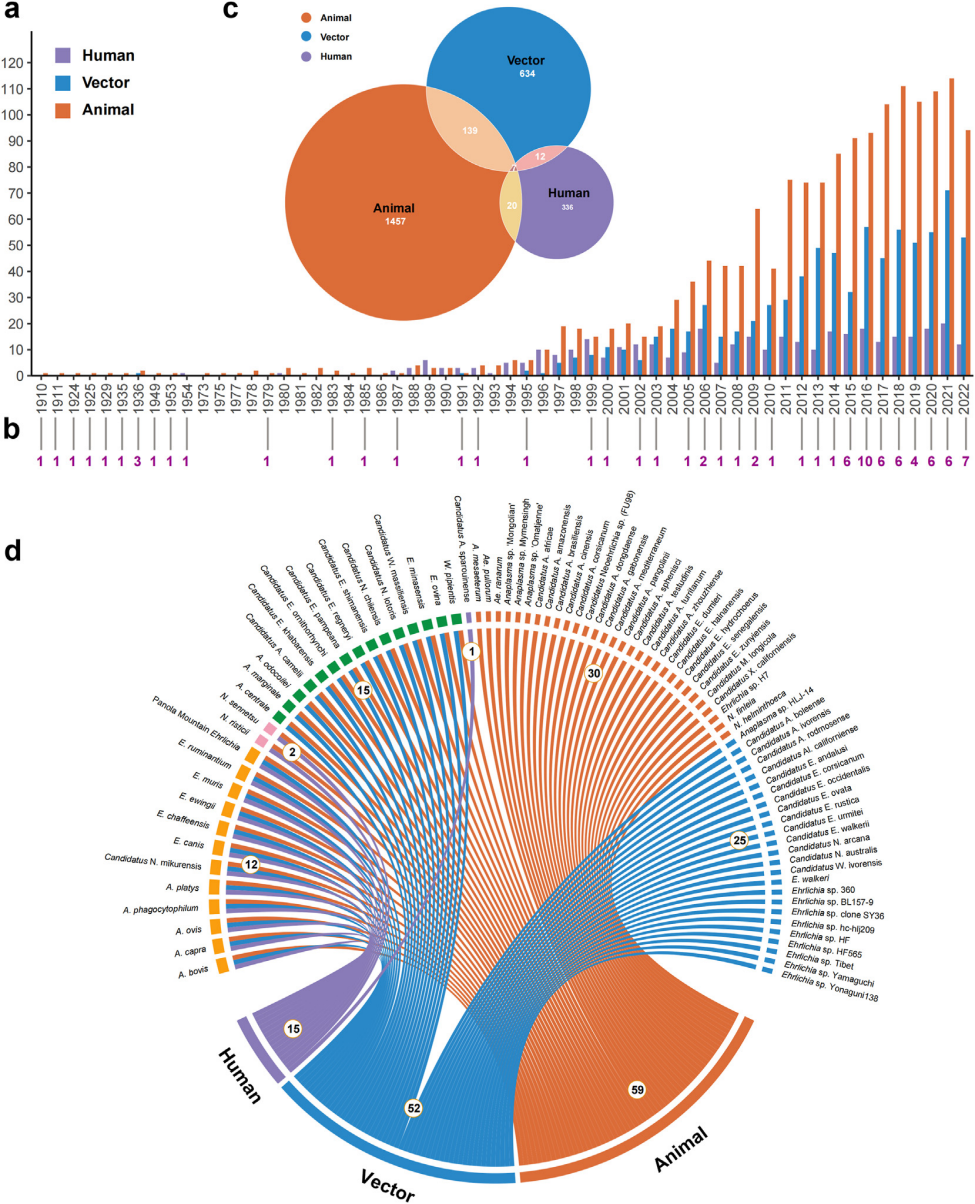
**Publications on Anaplasmataceae species infections in animals, vectors, and humans from 1910 to 2022.** (a) Annual number of publications stratified by host type; (b) Number of newly identified Anaplasmataceae members per year; (c) Total number of publications on Anaplasmataceae species detected in vectors, animals, and humans; (d) Chord diagram illustrating the relationship between Anaplasmataceae species and host types. Panels a and c present the distribution of literature categorized by host type, while panels b and d indicate the detection and distribution of Anaplasmataceae species across different hosts.

Our search strategy identified a total of 15,233 studies, with 2605 unique studies meeting the eligibility criteria and included in this analysis (Fig. 1). The first documented Anaplasmataceae species, *A. marginale*, was reported in 1910.^30^ By the end of 2022, a total of 85 Anaplasmataceae species had been identified, with the majority (53 species, 62·4%) discovered within the last decade. This surge in discoveries has also led to an increase in studies focussing on infections in animals and vectors (Fig. 2a and b). Anaplasmataceae species infections were most frequently reported from animals, followed by vectors and humans (Fig. 2c). Fifteen Anaplasmataceae species infected humans (Fig. 2d), among them, *A. phagocytophilum* was most frequently reported (in 1264 papers), followed by *E. canis* (518 papers), *A. platys* (276 papers), *E. chaffeensis* (257 papers), and *A. ovis* (150 papers) (Appendix 1 Supplementary Table S14).

Overall, 29 out of the 85 (34·1%) determined Anaplasmataceae species were associated with multiple hosts, including 15 linked to both arthropod vectors and animals, two linked to both animals and humans, 12 linked to all three types of hosts. The others were exclusively detected in animals, arthropod vectors, or humans (Fig. 2d).

**Fig. 3:**
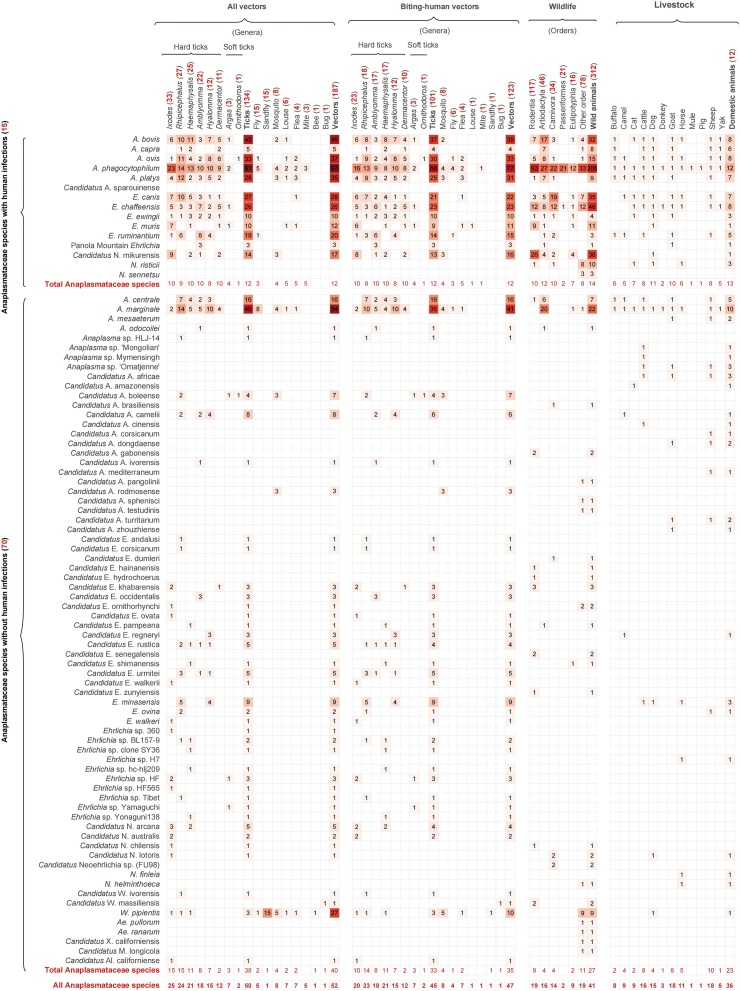
**Vectors and numbers of animal species from which each Anaplasmataceae species was detected.** The column headers indicate the total number of distinct host species that harbour various Anaplasmataceae species within each host category, with specific counts provided in parentheses. The numbers in the matrix present the total number of host species harbouring each specific Anaplasmataceae species. Vectors include tick, fly, sandfly, mosquito, louse, flea, mite, bee, and bug, with ticks further classified by genus. Animals include wildlife and domestic animals. Wildlife are categorized according to their taxonomic orders, while domestic animals such as dogs, cats, and cattle are not subdivided further in this context. Therefore, for these domestic animals, a single number of “one” is shown in the matrix for certain specific Anaplasmataceae species, indicating the presence of that particular Anaplasmataceae species identified in the respective domestic animal.

A total of 187 arthropod species, including 123 documented to bite humans, were found to be infected with 52 Anaplasmataceae members (Fig. 3; Appendix 1 Supplementary Table S15). Ticks (134 species) were the most predominant, with four soft tick species from two genera carrying eight species, and 130 hard tick species from six tick genera harbouring 46 species. *Ixodes* ticks hosted the highest number of Anaplasmataceae species (25 species), followed by *Rhipicephalus* (24 species), *Haemaphysalis* (21 species), *Amblyomma* (18 species), *Hyalomma* (15 species), and *Dermacentor* (12 species). Considering both the number of human-biting tick species and their pathogenicity, *Ixodes* ticks posed the highest risk in potentially transmitting Anaplasmataceae species, with 23 human biting species carrying ten Anaplasmataceae members. Eighteen species of *Rhipicephalus* carry nine Anaplasmataceae species; 17 species of *Amblyomma* carry ten members of Anaplasmataceae. In addition to ticks, flies and sandflies also carry Anaplasmataceae members (15 species each) (Fig. 3; Appendix 1 Supplementary Figures S1, S2).

Anaplasmataceae species infections were reported in totally 312 wildlife species and 12 domestic animal species (Fig. 3). The order Rodentia hosted the highest number of Anaplasmataceae species (n = 19), followed by Artiodactyla (n = 16), and Carnivora (n = 14). Livestock animals harbour a total of 36 Anaplasmataceae species, with goats and sheep having the largest number of detection (both 18 species), followed by cattle (n = 16), dogs (n = 15), and horses (n = 11) (Fig. 3; Appendix 1 Supplementary Figure S3).

Multiple Anaplasmataceae species were identified in a variety vectors, with *A. phagocytophilum* having the broadest range of vectors (97 vector species, including 81 tick species), followed by *A. marginale* (54 vector species, including 40 tick species), *Aanplasma bovis* (46 vector species, including 43 tick species), *A. ovis* (37 vector species, including 33 tick species), *A. platys* (35 vector species, including 28 tick species), *E. canis* (28 vector species, including 27 tick species), and *E. chaffeensis* (26 tick species) (Fig. 3; Appendix 1 Supplementary Table S16). Coinfections of two or three Anaplasmataceae species (mainly involving *A. phagocytophilum* and *E. chaffeensis*) were documented in 12 vector species (Appendix 1 Supplementary Table S17). *Anaplasma* *phagocytophilum* was also detected in the widest range of wildlife (208 species), followed by *E. chaffeensis* (46 species), *Candidatus* N. mikurensis (36 species), *E. canis* (35 species), and *A. bovis* (32 species) (Fig. 3; Appendix 1 Supplementary Table S16). A host specificity was observed, with *A. phagocytophilum* and *Candidatus* N. mikurensis mainly detected in wild animals, while *A. marginale*, *E. canis*, *A. ovis*, and *A. platys* mainly detected in cattle, dog, sheep, and dog respectively (Appendix 1 Supplementary Table S18).

A total of 52,315 human cases of Anaplasmataceae species infection was reported worldwide, with the majority (41,231 cases) confirmed by molecular assays (Appendix 1 Supplementary Table S19). Infection with *A. phagocytophilum* and *E. chaffeensis* accounted for 66·5% (34,796/52,315) and 32·4% (16,961/52,315) of overall cases, respectively, followed in number by *Candidatus* N. mikurensis (160 cases), *Ehrlichia muris* (139 cases), *E. canis* (123 cases), and *Ehrlichia ewingii* (71 cases). We analysed clinical symptoms of 7070 patients infected with eight pathogens. For *A. phagocytophilum* infection, fever (89·6–89·7%) and myalgia (85·7–86·4%) were the predominant symptoms. Similar flu-like and gastrointestinal symptoms were observed in *E. chaffeensis* infection and *E. canis* infection. A higher proportion of *A. capra* and *E. canis* cases showed rash as a symptom, whereas arthralgia was more common in *Neorickettsia sennetsu* infection (Appendix 1 Supplementary Table S20).

**Fig. 4:**
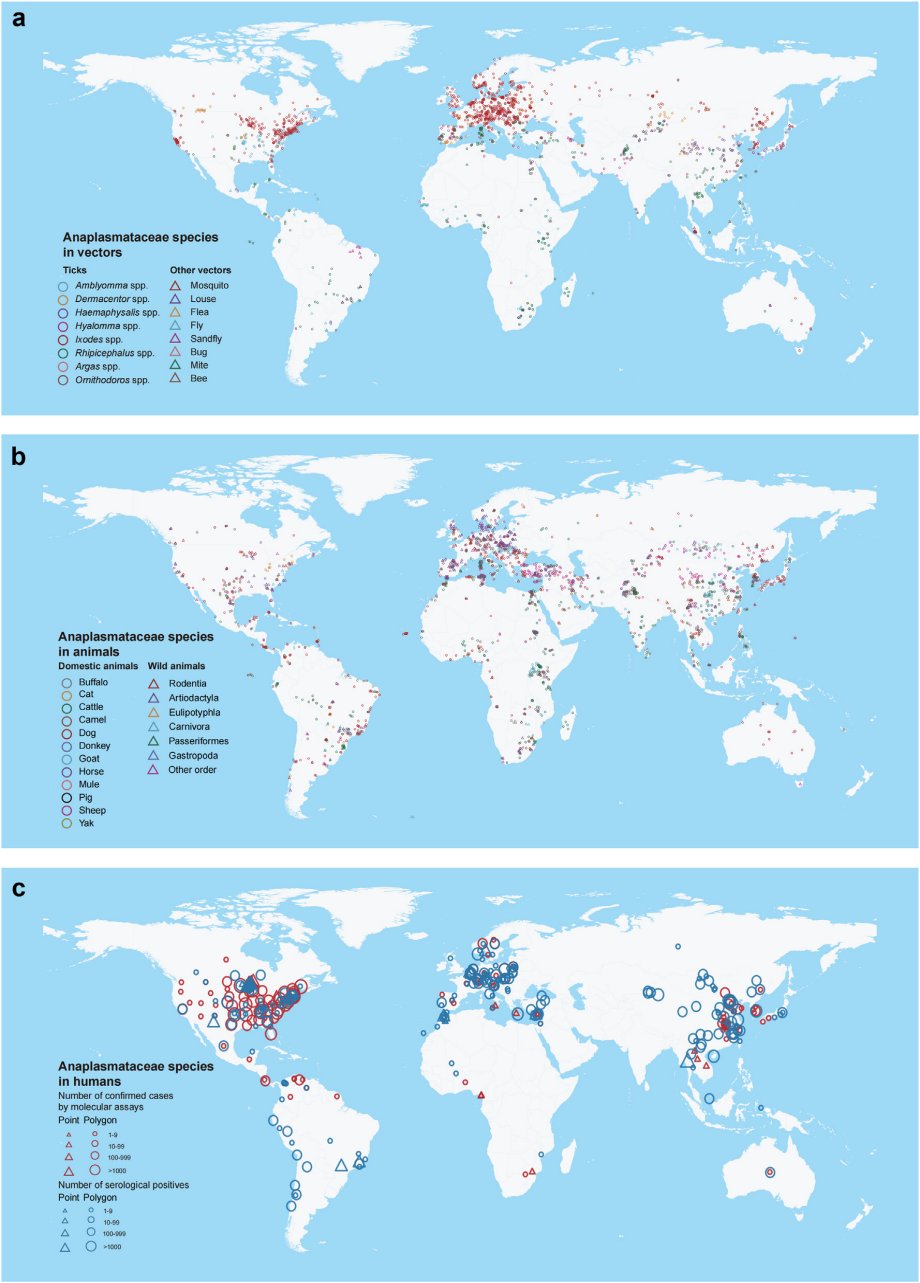
**Global distribution of laboratory-confirmed Anaplasmataceae species detected in vectors, animals, and humans based on literature sources.** (a) Vectors; (b) Animals; (c) Humans. In panel a, different shapes are used to differentiate ticks from other vectors. In panel b, shapes distinguish domestic animals from wild animals. In panel c, colours denote the detection methods employed, while shapes represent the type of location where Anaplasmataceae infections were identified, with the size of the shapes indicating the number of reported human cases.

The spatial distribution of Anaplasmataceae species in vectors varied based on latitudes and continents, primarily linked to tick vector distribution (Fig. 4a). For instance, *Amblyomma* ticks predominantly carry Anaplasmataceae species found in the Americas and Africa; *Ixodes* and *Dermacentor* mostly harbour Anaplasmataceae members at higher latitudes in the Northern Hemisphere; *Haemaphysalis* and *Hyalomma* are mainly distributed in Eurasia, while *Rhipicephalus* have a global pathogens distribution. Overall, Anaplasmataceae members had broader distribution within tick populations compared to other arthropod vectors. Anaplasmataceae species were mainly detected among wildlife and dogs in Europe and the Americas; while detection in sheep, goats, and cattle was reported throughout Eurasia (Fig. 4b). Human infections differed geographically regarding the test method (Fig. 4c).

**Fig. 5:**
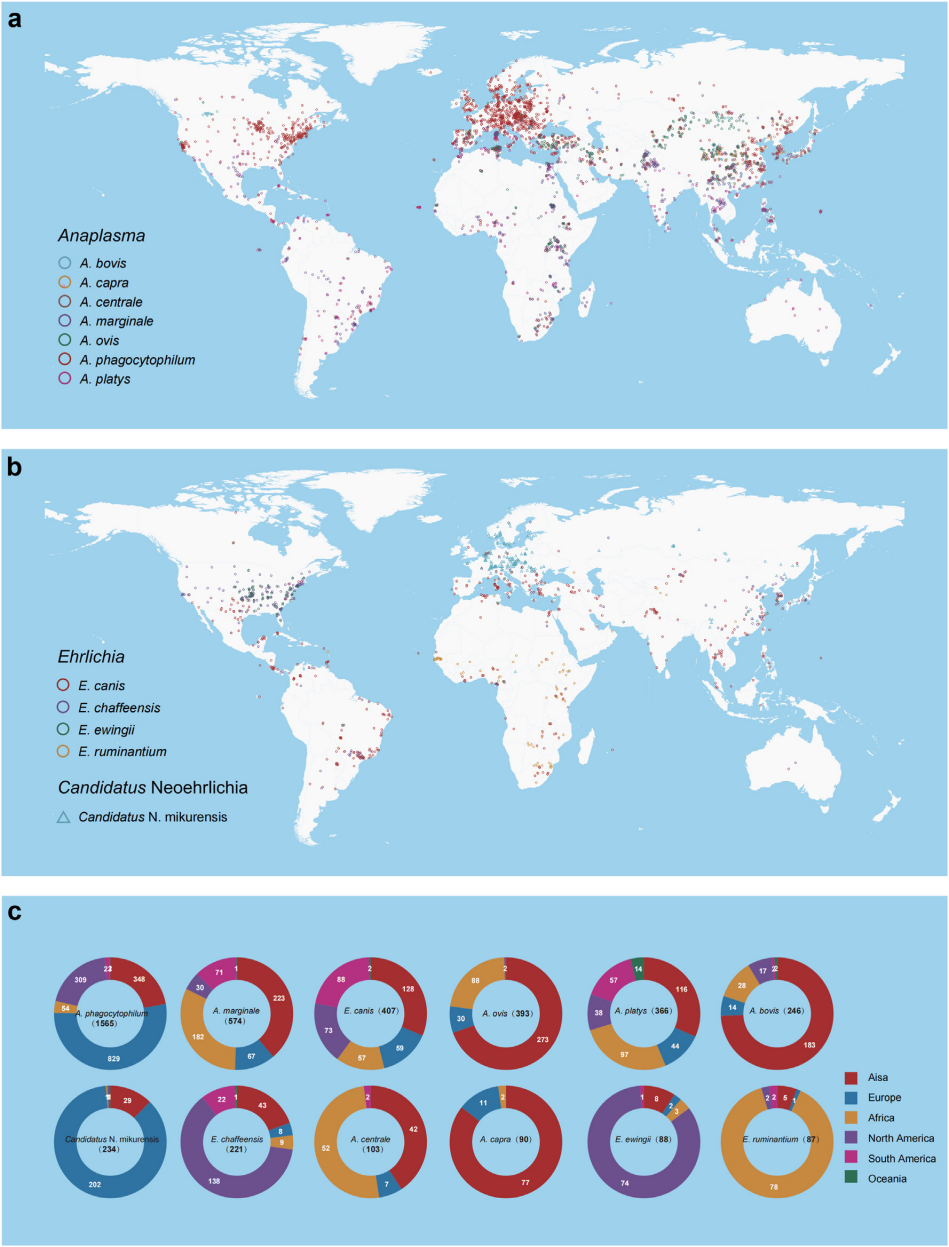
**Spatial distribution of the major Anaplasmataceae species on a global scale**. (a) The global distribution of major *Anaplasma* species; (b) The global distribution of major *Ehrlichia* and *Candidatus* Neoehrlichia species; (c) Number of detection locations in six continents for each predominant Anaplasmataceae species.

We mapped the distribution of 12 major species belonging to three genera (*Anaplasma*, *Ehrlichia*, and *Candidatus* Neoehrlichia), with each species recorded in over 80 occurrence locations. All 12 species were found in Eurasia and Africa. Specifically, *A. phagocytophilum* and *E. canis* were found on six continents, with higher prevalence in America and Eurasia. *Anaplasma* *marginale*, *A. ovis*, *A. platys*, and *Anaplasma centrale* were mainly reported in Asia and Africa. *Aanplasma bovis* and *A. capra* were mainly observed in Asia, while *E. chaffeensis* and *E. ewingii* were primarily distributed in North America. *Candidatus* Neoehrlichia mikurensis and *Ehrlichia ruminantium* were mainly found in Europe and Africa, respectively (Fig. 5a‒c, Appendix 1 Supplementary Table S10). The distribution of the other 73 Anaplasmataceae species was shown in Appendix 1 Supplementary Figure S4. When considering the infection in tick species, the meta-analysis revealed *A. phagocytophilum* had the highest prevalence in *I*. *persulcatus* (6·95%; 95% CI: 4·89–9·78%), *I. scapularis* (6·64%; 95% CI: 4·76–9·18%), *and I. ricinus* (4·55%; 95% CI: 3·90–5·32%); *Candidatus* N. mikurensis had the highest prevalence in *I. ricinus*, and both *E. canis* and *A. platys* had the highest prevalence in *Rh. sanguineus* (Appendix 1 Supplementary Table S12).

In addition, we also mapped the distribution of infections of the Anaplasmataceae species confirmed in this study after molecular validation. A total of 77 Anaplasmataceae members have been detected in 133 vectors, 158 wildlife species, and 10 domestic animals up to the present time, and the addition information of the validation are supplemented in Appendix 1 Supplementary Figures S35–S42.

The BRT models performed well for the eight vector tick species, with AUC values surpassing 0·9 and predictive accuracy exceeding 0·8 (Appendix 1 Supplementary Table S21 and Figure S5). The model results confirmed consistent distribution patterns projected in our previous studies for *I. pacificus*, *I. scapularis*, *I. persulcatus*, and *I. ricinus* as projected.^24^ Other vector ticks exhibited expanded distribution beyond what was previously projected in the year 2019 and 2020 respectively.^31^ *Dermacentor* *marginatus* is mainly distributed in the Mediterranean Sea region, and central/east Asia, while *Ha. longicornis* is projected to extend further into North America and Europe compared to previous findings (Appendix 1 Supplementary Figures S6–S13). The BRT models revealed that climatic covariates had the most influence on all evaluated tick species, while leaf area index primarily affected *I. persulcatus* and sheep density mainly influenced *D. marginatus* (Appendix 1 Supplementary Table S22; Figures S14–S21).

In ecological niche modelling analyses of the six major Anaplasmataceae species after molecular validation, when using data with polygon areas less than 900 km^2^, RF exhibited superior predictive performance compared to BRT and LASSO, as indicated by higher average AUC values (Appendix 1 Supplementary Figure S22). Data with polygon areas less than 100 km^2^ and 400 km^2^ were further used in RF model, and RF models demonstrated that higher predictive performance was obtained using a threshold of 400 km^2^. The average AUC varied between 0·925 for *A. ovis* and 0·968 for *E. canis* (Appendix 1 Supplementary Table S23). The models revealed that temperature and precipitation related variables were significant factors influencing the distribution of six major Anaplasmataceae species. Moreover, the HSIs of vector ticks had the most significant impact on the occurrence of *A. phagocytophilum*, *Candidatus* N. mikurensis, and *A. platys*, while urban built-up land areas and population density were key factors in determining the distribution of *E. canis*, *A. ovis*, and *A. marginale*. As for the impact of biological factors, goat density and mammalian richness primarily impacted occurrence of *A. ovis* while cattle density was closely associated with *A. marginale* (Appendix 1 Supplementary Table S24; Figures S23–S28).

By applying RF models, potential risk areas for six Anaplasmataceae members are projected to be located across larger regions than currently documented (Appendix 1 Supplementary Figures S29–S34). For the three Anaplasmataceae species with confirmed human pathogenicity and wide distribution, *A. phagocytophilum* poses the greatest risk, with 3·97 billion people and 8·95 million km^2^ area projected to be potentially affected, primary in Eurasia, North America and parts of Africa (Table 1). *Ehrlichia* *canis* is projected to cover approximately 3·94 million km^2^ of potentially suitable habitat, and *Candidatus* N. mikurensis is mainly predicted to distribute in Eurasia with potential risk areas of 2·36 million km^2^.

**Table 1:** Average AUC, population sizes and areas predicted by the RF models at potentially high risk of exposure to the six major Anaplasmataceae species.

Anaplasmataceae species	Average testing AUC	Population size (million)					Area (10,000 km^2^)				
		Eurasia	Africa	Americas	Oceania	Worldwide	Eurasia	Africa	Americas	Oceania	Worldwide
*A. phagocytophilum*	0·964 (0·949–0·978)	2706·04	411·34	825·04	28·57	3970·99	618·74	26·57	220·28	29·13	894·72
*Candidatus* N. mikurensis	0·957 (0·912–0·983)	530·86	1·18	–	–	532·04	235·72	0·02	–	–	235·74
*E. canis*	0·968 (0·925–0·999)	3127·76	892·10	938·84	25·98	4984·68	188·44	98·26	103·45	3·58	393·73
*A. ovis*	0·925 (0·871–0·967)	–	–	–	–	–	669·51	253·04	–	–	922·55
*A. platys*	0·926 (0·852–0·981)	–	–	–	–	–	399·63	234·66	103·15	16·06	753·50
*A. marginale*	0·926 (0·868–0·963)	–	–	–	–	–	442·52	355·23	292·56	60·75	1151·06

## Discussion

In this study, we compiled a comprehensive database detailing the spatial distribution of Anaplasmataceae species identified in vectors, animals, and humans. This resource enables researchers to gain an in-depth understanding of the relationships between Anaplasmataceae species and their potential vectors and animal hosts. Furthermore, by mapping the distribution of Anaplasmataceae species and conducting prediction models for major members, we can achieve a thorough comprehension of their global distribution patterns. Our findings not only help to strengthen surveillance in high-risk areas but also provide a robust foundation for guiding future public health initiatives focused on the prevention and control of Anaplasmataceae species.

Based on extensive data curation and analysis, 85 Anaplasmataceae species have been determined, with over 50% discovered in the last decade (Fig. 2b). This highlights their potential expansion in diversity and scope, which is inextricably linked to the intensive research undertaken in recent years.^10^^,^^32^^,^^33^ More than 60% of Anaplasmataceae members have been detected in 187 vectors species, 48·2% were detected in 312 species of wild animals, while 36 Anaplasmataceae species had been identified in domestic animals (Fig. 3; Appendix 1 Supplementary Table S16). The vector and host plasticity observed for Anaplasmataceae species highlight their ability to infect human beings.^1^^,^^12^^,^^34^ With 17·6% of Anaplasmataceae members currently were known to cause human infections (Fig. 2d; Appendix 1 Supplementary Table S20), it is necessary to enhanced active surveillance to track changes in their dynamic and emerging threats to humans.

Our findings emphasize ticks as the primary vectors in the distribution of Anaplasmataceae species, with hard ticks primarily responsible for their transmission, however, Anaplasmataceae members can also be detected in certain soft ticks such as *Ornithodoros lahorensis*, *Argas japonicus*, and *Argas vespertilionis* (Fig. 3; Appendix 1 Supplementary Figure S1), which may also serve as potential vectors.^35^ Based on our niche modelling analysis, HSIs of the eight major tick vectors were robustly predicted, significantly contributing to the distribution of each major Anaplasmataceae species (Appendix 1 Supplementary Table S24; Figures S23–S28). In this study, the HSI values for multiple tick species potentially carrying a pathogen such as *A. phagocytophilum* were determined by selecting the maximum value. This approach prioritized species with highest transmission potential across different regions, thereby enhancing model performance. It also elucidates the roles of locally distributed tick species that significantly contribute to the occurrence of specific Anaplasmataceae species in various regions. For instance, *I. scapularis* and *I. pacificus* in North America, *I. ricinus* and *I. persulcatus* in Europe, as well as *I. persulcatus* in East Asia are key vectors for *A. phagocytophilum*.^2^ Notably, while most vector ticks have distinct ecological niche due to local adaptions, some ticks have expanded their habitats as a result of climate changes and human activities (Appendix 1 Supplementary Figures S6–S13).^25^^,^^36^ For example, *Ha. longicornis* has been observed to expand from its original habitat in East Asia to northeastern United States, Australia, and New Zealand, underscoring the importance of monitoring ticks in both endemic and newly colonized regions.^37^^,^^38^ This geographic expansion may influence the spread of tick-borne pathogens, highlighting the need for enhanced surveillance and control strategies. However, the prevalence of tick-borne pathogens can't be determined solely by the reported presence of vectors. In many economically disadvantaged regions, limited research infrastructure and surveillance capabilities often lead to underreporting, thereby resulting in an underestimation of the true disease burden.^39^^,^^40^ Thereby, strengthening monitoring and diagnostic capacity in these areas is essential for improving our understanding of tick-borne disease dynamics and addressing existing research gaps.

Wildlife serves as natural hosts or accidental hosts for tick-borne pathogens, often neglected in the prevention and control strategies against these pathogens. Notably, the prevalence of *A. phagocytophilum* and *Candidatus* N. mikurensis is particularly high among wildlife, especially within Cervidae animals like *Capreolus capreolus* and rodent species such as *Myodes glareolus* (Appendix 1 Supplementary Tables S16, S18). These animals play a crucial role in the distribution and transmission of tick-borne pathogens. They share habitat with major tick vectors of Anaplasmataceae species such as *I. persulcatus*, *Ha. longicornis*, and *I. ricinus*. When these ticks bite the animal hosts, they can become infected and subsequently facilitate the propagation and transmission of these pathogens.^41^^,^^42^ Furthermore, environmental changes, such as climate change and habitat fragmentation, have led to the expansion of certain wildlife populations, notably rodents, even into human-inhabited areas, thereby increasing the risk of Anaplasmataceae infections in humans.^42^, ^43^, ^44^ Unlike the two Anaplasmataceae species mentioned above, *A. marginale* and *A. ovis* show higher prevalence in domestic animals, especially in cattle, sheep, and goats, which is mainly attributed to the presence of specific tick species such as *D. marginatus* and *Rh. microplus* (Appendix 1 Supplementary Tables S16, S18). Moreover, companion animals, especially dogs, play an important role as “bridges” for the transmission of tick-borne pathogens.^45^^,^^46^ Although dogs themselves rarely directly transmit tick-borne pathogens, the ticks they harbour can disseminate Anaplasmataceae species, such as *A. platys* and *E. canis* (Appendix 1 Supplementary Tables S16, S18), to humans through bites. This intermediary role highlights the necessity of monitoring companion animals, especially in areas where dogs are in close contact with human and wildlife habitats, thereby facilitating pathogen transmission pathways.^46^^,^^47^ Surveillance of wild, domestic, and companion animals is essential for elucidating pathogen dynamics and mitigating economic losses in the livestock sector. Furthermore, it provides crucial data to veterinary and environmental authorities for implementing timely interventions and enhancing public health protection.

*Anaplamsaphagocytophilum* had the highest number of reported human cases among Anaplasmataceae species, followed by *E. chaffeensis* and *Candidatus* N. mikurensis (Appendix 1 Supplementary Table S19). Our modelling analysis revealed that their potential distribution areas significantly exceeded the currently observed regions (Appendix 1 Supplementary Figures S29–S34). In recent years, tick species such as *I. persulcatus*, *Ha. longicornis*, and *I. ricinus*, which serve as important vector of these pathogenic Anaplasmataceae species, have expanded their suitable habitats due to factors like global warming, alterations in microclimate and human activities.^25^^,^^36^^,^^48^ Additionally, for Anaplasmataceae members that were predominantly found in animal hosts (Appendix 1 Supplementary Figure S17), for example, *E. canis* and *A. platys* in dogs,^49^^,^^50^ an increasing risk for transmission of might be accelerated by urbanization and increased population density.^49^ This highlights the critical importance of an integrated One Health approach, which links human, animal, and environmental health.^51^ Early surveillance among vectors and animals in areas with predicted risks, along with serological surveys of high-risk populations in close contact with competent animal hosts, is essential for early detection and effective management.^52^^,^^53^ Strengthening collaboration between veterinary, medical, and environmental sectors is crucial for developing comprehensive strategies that mitigate the risk of Anaplasmataceae transmission and safeguard public health.

There are several limitations to our study. First, the data regarding occurrences of Anaplasmataceae species infections for this study were extracted from published literature. While multiple databases provide a broad spectrum of information, there remain limitations in terms of data quality and completeness. Variations in testing methods, sample sizes, and reporting standards across different countries and regions, especially in low-income areas, may lead to false negatives due to constrained testing capacities, thereby underestimating the infection risk. Furthermore, some studies were excluded from this study due to insufficient methodological descriptions or improper sample handling, potentially resulting in a partial loss of positive data. Second, our study relied on existing literature, which is inherently susceptible to publication bias and selection bias. This may compromise the representativeness of the data, as unpublished studies or under-surveyed regions could have been overlooked. Third, during the modelling analyses, we excluded serological data from predictive models, which may have led to an underestimation of pathogen infection risks. Finally, the modelling relied on a single static dataset of ecological variables, failing to account for temporal dynamics and potentially overlooking seasonal or interannual fluctuations in species distributions. Future research should prioritize enhancing data quality by incorporating field surveillance data and utilizing dynamic datasets to improve modelling accuracy.

Despite these limitations, our study created a database covering the currently recognized species of the family Anaplasmataceae, which provided fundamental data to support the understanding of the current distribution of Anaplasmataceae species. The study identified the potential vectors and animal hosts of Anaplasmataceae species and revealed the human distribution of pathogenic pathogen infections and their associated symptoms. By modelling the ecological niche of six major Anaplasmataceae members, key ecological factors affecting their distributions and suitable habitats were revealed. These findings are of great practical significance for improving clinical diagnosis, optimizing public health surveillance systems, and developing effective prevention and control strategies. Meanwhile, this study was conducted on a global scale, with particular emphasis on low- and middle-income countries and regions that have limited data resources. This study can contribute to the broader management of zoonotic diseases and provide a scientific foundation for the development of relevant public health policies, thereby enabling a more effective response to the global public health challenges posed by Anaplasmataceae species infections. Future research should further improve data quality and develop dynamic models adapted to environmental changes in order to continuously optimize prevention and control strategies.

## Contributors

The authors contributed as follows: WL, LQF, SIH, and YHW conceived, designed, and supervised this study. XBH, TT, JJC, YYZ, QX, GLW, and YZ searched databases, screened literatures and evaluated their quality. XBH, TT, JJC, YYZ, and CLL created data extraction forms and extracted and analysed the data. XBH, TT, JJC, YYZ, and CLL helped to collate data and construct graphs and provided models and statistical analysis methods. XBH, TT, WL, and LQF wrote draughts of the manuscript and interpreted the findings. WL, LQF, SIH, and YHW critiqued and revised the manuscript. WL, LQF, XBH, TT, and JJC validated the underlying data, had access to the data in the study, and were responsible for the completeness and accuracy of the data. All the authors read the manuscript, provided feedback, and approved the final version.

## Data sharing statement

All the data collected in this study will be made publicly available after the acceptance of this manuscript for publication.

## Declaration of interests

We declare no competing interests.

## Appendix A Supplementary data

Appendix 3

Appendix 4_A. marginale_forest plot

Appendix4_A. ovis_forest plot

Appendix4_A. phagocytophilum_forest plot

Appendix4_Candidatus N. mikurensis_forest plot

Appendix4_E. canis_forest plot

## References

[1] Dumler J.S., Barbet A.F., Bekker C.P. (2001). Reorganization of genera in the families Rickettsiaceae and Anaplasmataceae in the order Rickettsiales: unification of some species of *Ehrlichia* with *Anaplasma, Cowdria* with *Ehrlichia* and *Ehrlichia* with *Neorickettsia*, descriptions of six new species combinations and designation of *Ehrlichia equi* and “HGE agent” as subjective synonyms of *Ehrlichia phagocytophila*. Int J Syst Evol Microbiol.

[2] Karshima S.N., Ahmed M.I., Mohammed K.M. (2023). Worldwide meta-analysis on *Anaplasma phagocytophilum* infections in animal reservoirs: prevalence, distribution and reservoir diversity. Vet Parasitol Reg Stud Reports.

[3] Oechslin C.P., Heutschi D., Lenz N. (2017). Prevalence of tick-borne pathogens in questing *Ixodes ricinus* ticks in urban and suburban areas of Switzerland. Parasit Vectors.

[4] Sarwar M.S., Jahan N., Shahbaz F. (2018). Molecular detection and characterization of *Wolbachia pipientis* from *Culex quinquefasciatus* collected from Lahore, Pakistan. Am J Trop Med Hyg.

[5] Torina A., Blanda V., Antoci F. (2013). A molecular survey of *Anaplasma* spp., *Rickettsia* spp., *Ehrlichia canis* and *Babesia microti* in foxes and fleas from Sicily. Transbound Emerg Dis.

[6] Hernández-Velasco A., Sánchez-Montes S., Romero-Salas D. (2020). First record of natural infection with *Anaplasma marginale* in sucking lice infesting the water buffalo (*Bubalus bubalis*) in Mexico. Parasitol Res.

[7] Skoracki M., Michalik J., Skotarczak B. (2006). First detection of *Anaplasma phagocytophilum* in quill mites (Acari: Syringophilidae) parasitizing passerine birds. Microbes Infect.

[8] Maurer F.P., Keller P.M., Beuret C. (2013). Close geographic association of human neoehrlichiosis and tick populations carrying “*Candidatus* Neoehrlichia mikurensis” in eastern Switzerland. J Clin Microbiol.

[9] Johnson D.K.H., Schiffman E.K., Davis J.P. (2015). Human infection with *Ehrlichia muris*-like pathogen, United States, 2007-2013(1). Emerg Infect Dis.

[10] Vieira T.S.W.J., Collere F.C.M., Ferrari L.D.R. (2022). Novel Anaplasmataceae agents *Candidatus* Ehrlichia hydrochoerus and *Anaplasma* spp. infecting Capybaras, Brazil. Emerg Infect Dis.

[11] Duron O., Koual R., Musset L. (2022). Novel chronic anaplasmosis in splenectomized patient, Amazon Rainforest. Emerg Infect Dis.

[12] Zobba R., Murgia C., Dahmani M. (2022). Emergence of *Anaplasma* species related to *A. phagocytophilum* and *A. platys* in Senegal. Int J Mol Sci.

[13] Guo W.P., Tian J.H., Lin X.D. (2016). Extensive genetic diversity of Rickettsiales bacteria in multiple mosquito species. Sci Rep.

[14] Mangombi J.B., N’dilimabaka N., Lekana-Douki J.B. (2021). First investigation of pathogenic bacteria, protozoa and viruses in rodents and shrews in context of forest-savannah-urban areas interface in the city of Franceville (Gabon). PLoS One.

[15] Lin Z.T., Ye R.Z., Liu J.Y. (2023). Epidemiological and phylogenetic characteristics of emerging *Anaplasma capra*: a systematic review with modeling analysis. Infect Genet Evol.

[16] Karshima S., Ahmed M., Mohammed K., Pam V. (2023). Global status of *Anaplasma phagocytophilum* infections in human population: a 50-year (1970–2020) meta-analysis. J Vector Borne Dis.

[17] Silaghi C., Beck R., Oteo J.A. (2016). Neoehrlichiosis: an emerging tick-borne zoonosis caused by *Candidatus* Neoehrlichia mikurensis. Exp Appl Acarol.

[18] Matei I.A., Estrada-Peña A., Cutler S.J. (2019). A review on the eco-epidemiology and clinical management of human granulocytic anaplasmosis and its agent in Europe. Parasit Vectors.

[19] Aidoo O.F., Souza P.G.C., Silva R.S. (2023). Modeling climate change impacts on potential global distribution of *Tamarixia radiata* Waterston (Hymenoptera: Eulophidae). Sci Total Environ.

[20] Zhang Y.Y., Sun Y.Q., Chen J.J. (2023). Mapping the global distribution of spotted fever group rickettsiae: a systematic review with modeling analysis. Lancet Digit Health.

[21] Charbonnel A., Lambert P., Lassalle G. (2023). Developing species distribution models for critically endangered species using participatory data: the European sturgeon marine habitat suitability. Estuar Coast Shelf Sci.

[22] Sage K.M., Johnson T.L., Teglas M.B. (2017). Ecological niche modeling and distribution of *Ornithodoros hermsi* associated with tick-borne relapsing fever in western North America. PLoS Negl Trop Dis.

[23] Messina J.P., Pigott D.M., Golding N. (2015). The global distribution of Crimean-Congo hemorrhagic fever. Trans R Soc Trop Med Hyg.

[24] Tang T., Zhu Y., Zhang Y.Y. (2024). The global distribution and the risk prediction of relapsing fever group Borrelia: a data review with modeling analysis. Lancet Microbe.

[25] Miao D., Dai K., Zhao G.P. (2020). Mapping the global potential transmission hotspots for severe fever with thrombocytopenia syndrome by machine learning methods. Emerg Microbes Infect.

[26] Che T.L., Jiang B.G., Xu Q. (2022). Mapping the risk distribution of *Borrelia burgdorferi* sensu lato in China from 1986 to 2020: a geospatial modeling analysis. Emerg Microbes Infect.

[27] Arenas-Castro S., Sillero N. (2021). Cross-scale monitoring of habitat suitability changes using satellite time series and ecological niche models. Sci Total Environ.

[28] Schisterman E.F., Perkins N.J., Liu A., Bondell H. (2005). Optimal cut-point and its corresponding Youden Index to discriminate individuals using pooled blood samples. Epidemiology.

[29] Ruopp M.D., Perkins N.J., Whitcomb B.W., Schisterman E.F. (2008). Youden Index and optimal cut-point estimated from observations affected by a lower limit of detection. Biom J.

[30] Theiler A. (1910).

[31] Wang F., Wang D., Guo G. (2019). Species delimitation of the *Dermacentor* ticks based on phylogenetic clustering and niche modeling. PeerJ.

[32] Crosby F.L., Wellehan J.F.X., Pertierra L. (2021). Molecular characterization of “*Candidatus* Anaplasma testudinis”: an emerging pathogen in the threatened Florida gopher tortoise (*Gopherus polyphemus*). Ticks Tick Borne Dis.

[33] Buysse M., Koual R., Binetruy F. (2024). Detection of *Anaplasma* and *Ehrlichia* bacteria in humans, wildlife, and ticks in the Amazon rainforest. Nat Commun.

[34] Harris R.M., Couturier B.A., Sample S.C. (2016). Expanded geographic distribution and clinical characteristics of *Ehrlichia ewingii* infections, United States. Emerg Infect Dis.

[35] Zhao L., Lin X.M., Li F. (2018). A survey of argasid ticks and tick-associated pathogens in the Peripheral Oases around Tarim Basin and the first record of *Argas japonicus* in Xinjiang, China. PLoS One.

[36] Ma R., Li C., Gao A. (2024). Tick species diversity and potential distribution alternation of dominant ticks under different climate scenarios in Xinjiang, China. PLoS Negl Trop Dis.

[37] P Price K.J., Ayres B.N., Maes S.E. (2022). First detection of human pathogenic variant of *Anaplasma phagocytophilum* in field-collected *Haemaphysalis longicornis*, Pennsylvania, USA. Zoonoses Public Health.

[38] Rainey T., Occi J.L., Robbins R.G., Egizi A. (2018). Discovery of *Haemaphysalis longicornis* (Ixodida: Ixodidae) parasitizing a sheep in New Jersey, United States. J Med Entomol.

[39] Yean S., Prasetyo D.B., Marcombe S. (2024). Challenges for ticks and tick-borne diseases research in Southeast Asia: insight from the first international symposium in Cambodia. PLoS Negl Trop Dis.

[40] Gondard M., Cabezas-Cruz A., Charles R.A. (2017). Ticks and Tick-borne pathogens of the Caribbean: current understanding and future directions for more comprehensive surveillance. Front Cell Infect Microbiol.

[41] Kazimírová M., Hamšíková Z., Špitalská E. (2018). Diverse tick-borne microorganisms identified in free-living ungulates in Slovakia. Parasit Vectors.

[42] Sato M., Ikeda S., Arai R. (2021). Diversity and distribution of ticks in Niigata prefecture, Japan (2016-2018): changes since 1950. Ticks Tick Borne Dis.

[43] Gilbert L. (2021). The impacts of climate change on ticks and tick-borne disease risk. Annu Rev Entomol.

[44] Nova N., Athni T.S., Childs M.L. (2022). Global change and emerging infectious diseases. Annu Rev Resour Economics.

[45] Król N., Kiewra D., Szymanowski M. (2015). The role of domestic dogs and cats in the zoonotic cycles of ticks and pathogens. Preliminary studies in the Wrocław Agglomeration (SW Poland). Vet Parasitol.

[46] Giannelli A., Schnyder M., Wright I., Charlier J. (2024). Control of companion animal parasites and impact on One Health. One Health.

[47] Skotarczak B. (2018). The role of companion animals in the environmental circulation of tick-borne bacterial pathogens. Ann Agric Environ Med.

[48] Uusitalo R., Siljander M., Lindén A. (2022). Predicting habitat suitability for *Ixodes ricinus* and *Ixodes persulcatus* ticks in Finland. Parasit Vectors.

[49] Tadesse H., Grillini M., Simonato G. (2023). Epidemiological survey on Tick-borne pathogens with zoonotic potential in dog populations of Southern Ethiopia. Trop Med Infect Dis.

[50] Nicholson W.L., Allen K.E., McQuiston J.H. (2010). The increasing recognition of rickettsial pathogens in dogs and people. Trends Parasitol.

[51] Shaheen M.N.F. (2022). The concept of one health applied to the problem of zoonotic diseases. Rev Med Virol.

[52] Sharan M., Vijay D., Yadav J.P. (2023). Surveillance and response strategies for zoonotic diseases: a comprehensive review. Sci One Health.

[53] Leifels M., Khalilur Rahman O., Sam I.C. (2022). The one health perspective to improve environmental surveillance of zoonotic viruses: lessons from COVID-19 and outlook beyond. ISME Commun.

