# Supplementary material for: The global distribution and risk prediction of Anaplasmataceae species: a systematic review and geospatial modelling analysis
Source: eBioMedicine. 2025 Apr 23;115:105722. doi: 10.1016/j.ebiom.2025.105722 (PMC12051633; doi:10.1016/j.ebiom.2025.105722)

**Supplementary appendix**

Supplement to: The global distribution and the risk prediction of Anaplasmataceae species: a systematic review and geospatial modelling analysis

Content

[Supplementary Results 4](#_Toc193101444)

[Supplementary table 1: The PRISMA Checklist 5](#_Toc193101445)

[Supplementary table 2: Literature search strategy for two major databases. 8](#_Toc193101446)

[Supplementary table 3: The inclusion and exclusion criteria of screening publications. 9](#_Toc193101447)

[Supplementary table 4: The laboratory tests used to detect Anaplasmataceae species in this study. 10](#_Toc193101448)

[Supplementary table 5: Variables extracted from selected studies. 11](#_Toc193101449)

[Supplementary table 6: Ecological factors potentially associated with the ticks and tick-borne pathogens used in the modelling analysis. 12](#_Toc193101450)

[Supplementary table 7: The spatial resolution, study duration and source of the included variables. 15](#_Toc193101451)

[Supplementary table 8: Variables used for ecological modelling in this study. 16](#_Toc193101452)

[Supplementary table 9: Variables on the occurrence of Anaplasmataceae species infections and 44 ecoclimatic, environmental, biological, and socioeconomic covariates used in this study. 18](#_Toc193101453)

[Supplementary table 10: The information on the location occurrences at different scales of all Anaplasmataceae species. 19](#_Toc193101454)

[Supplementary table 11: The information on the location occurrences at different scales of all Anaplasmataceae species after molecular validation. 23](#_Toc193101455)

[Supplementary table 12: Meta-analysis of six major Anaplasmataceae species in ticks. 27](#_Toc193101456)

[Supplementary table 13: The number of studies and occurrence locations for the three tick species. 31](#_Toc193101457)

[Supplementary table 14: The references for all the 85 Anaplasmataceae species. 32](#_Toc193101458)

[Supplementary table 15: The evidence for vectors carrying Anaplasmataceae species are capable of biting humans. 39](#_Toc193101459)

[Supplementary table 16: Vector/Host type and number infected by species in the Anaplasmataceae family. 45](#_Toc193101460)

[Supplementary table 17: The coinfection of Anaplasmataceae species and their infected vectors. 49](#_Toc193101461)

[Supplementary table 18: The source of detected positions for six main Anaplasmataceae species and their main host animal information. 50](#_Toc193101462)

[Supplementary table 19: The number of human cases with pathogenic Anaplasmataceae species infection by diagnosis methods. 51](#_Toc193101463)

[Supplementary table 20: Clinical characteristics of human infections with Anaplasmataceae species. 52](#_Toc193101464)

[Supplementary table 21: The predictive accuracy of the predictive models for the eight ticks based on BRT models. 54](#_Toc193101465)

[Supplementary table 22: The RCs of significant contributors to the occurrence of eight main species of ticks based on BRT models. 55](#_Toc193101466)

[Supplementary table 23: Sensitive analysis of different threshold for polygon occurrences used in RF models of the six Anaplasmataceae species· 57](#_Toc193101467)

[Supplementary table 24: The RCs of significant contributors to the spatial distribution of the six major Anaplasmataceae species based on RF models· 58](#_Toc193101468)

[Supplementary figure 1: The relationship matrix of Anaplasmataceae species and involved ticks. 59](#_Toc193101469)

[Supplementary figure 2: The relationship matrix of Anaplasmataceae species and other vectors. 60](#_Toc193101470)

[Supplementary figure 3: The relationship matrix of Anaplasmataceae species and animals. 61](#_Toc193101471)

[Supplementary figure 4: The global distribution of the other 73 Anaplasmataceae species. 62](#_Toc193101472)

[Supplementary figure 5: Predictive performance of BRT models for eight main ticks. 63](#_Toc193101473)

[Supplementary figure 6: The predicted HSI of *Ixodes scapularis* in North America and results of internal and external validation of models. 64](#_Toc193101474)

[Supplementary figure 7: The predicted HSI of *Ixodes pacificus* in North America and results of internal and external validation of models. 65](#_Toc193101475)

[Supplementary figure 8: The predicted HSI of *Ixodes persulcatus* in Eurasia and results of internal and external validation of models. 66](#_Toc193101476)

[Supplementary figure 9: The predicted HSI of *Ixodes ricinus* and results of internal and external validation of models. 67](#_Toc193101477)

[Supplementary figure 10: The predicted HSI of *Haemaphysalis longicornis* at worldwide and results of internal and external validation of models. 68](#_Toc193101478)

[Supplementary figure 11: The predicted HSI of *Dermacentor marginatus* in Eurasia and Africa and results of internal and external validation of models. 69](#_Toc193101479)

[Supplementary figure 12: The predicted HSI of *Rhipicephalus microplus* at worldwide and results of internal and external validation of models. 70](#_Toc193101480)

[Supplementary figure 13: The predicted HSI of *Rhipicephalus sanguineus* at worldwide and results of internal and external validation of models. 71](#_Toc193101481)

[Supplementary figure 14: Effects of major predictors (RCs >3%) for presence of *Ixodes scapularis* based on BRT models. 72](#_Toc193101482)

[Supplementary figure 15: Effects of major predictors (RCs >3%) for presence of *Ixodes pacificus* based on BRT models. 73](#_Toc193101483)

[Supplementary figure 16: Effects of major predictors (RCs >3%) for presence of *Ixodes persulcatus* based on BRT models. 74](#_Toc193101484)

[Supplementary figure 17: Effects of major predictors (RCs >3%) for presence of *Ixodes ricinus* based on BRT models. 75](#_Toc193101485)

[Supplementary figure 18: Effects of major predictors (RCs >3%) for presence of *Haemaphysalis longicornis* based on BRT models. 76](#_Toc193101486)

[Supplementary figure 19: Effects of major predictors (RCs >3%) for presence of *Dermacentor marginatus* based on BRT models. 77](#_Toc193101487)

[Supplementary figure 20: Effects of major predictors (RCs >3%) for presence of *Rhipicephalus microplus* based on BRT models. 78](#_Toc193101488)

[Supplementary figure 21: Effects of major predictors (RCs >3%) for presence of *Rhipicephalus sanguineus* based on BRT models. 79](#_Toc193101489)

[Supplementary figure 22: Predictive performance of the three machine-learning algorithms. 80](#_Toc193101490)

[Supplementary figure 23: Effects of major predictors (RCs >3%) for presence of *Anaplasma phagocytophilum* based on RF model. 82](#_Toc193101491)

[Supplementary figure 24: Effects of major predictors (RCs >3%) for presence of *Candidatus* Neoehrlichia mikurensis based on RF models. 83](#_Toc193101492)

[Supplementary figure 25: Effects of major predictors (RCs >3%) for presence of *Ehrlichia canis* based on RF models. 84](#_Toc193101493)

[Supplementary figure 26: Effects of major predictors (RCs >3%) for presence of *Anaplasma ovis* based on RF models. 85](#_Toc193101494)

[Supplementary figure 27: Effects of major predictors (RCs >3%) for presence of *Anaplasma platys* based on RF models. 86](#_Toc193101495)

[Supplementary figure 28: Effects of major predictors (RCs >3%) for presence of *Anaplasma marginale* based on RF models. 87](#_Toc193101496)

[Supplementary figure 29: The global recorded and predicted distributions of *A. phagocytophilum*. 88](#_Toc193101497)

[Supplementary figure 30: The recorded and predicted distributions of *Candidatus* N. mikurensis in Eurasia and Africa. 89](#_Toc193101498)

[Supplementary figure 31: The global recorded and predicted distributions of 90](#_Toc193101499)

[Supplementary figure 32: The recorded and predicted distributions of *A. ovis* in Eurasia and Africa. 91](#_Toc193101500)

[Supplementary figure 33: The global recorded and predicted distributions of 92](#_Toc193101501)

[Supplementary figure 34: The global recorded and predicted distributions of *A. marginale*. 93](#_Toc193101502)

[Supplementary figure 35: Publications searched from 1910 to 2022 on Anaplasmataceae species infection in animals, vectors, and humans after molecular validation. 94](#_Toc193101503)

[Supplementary figure 36: Vectors and numbers of animal species from which each Anaplasmataceae species was detected after molecular validation. 95](#_Toc193101504)

[Supplementary figure 37: Global distribution of laboratory confirmed Anaplasmataceae species detection in vectors, animals, and humans from literature sources after molecular validation. 96](#_Toc193101505)

[Supplementary figure 38: Spatial distribution of the major Anaplasmataceae species on a global scale after molecular validation. 97](#_Toc193101506)

[Supplementary figure 39: The relationship matrix of Anaplasmataceae species and involved ticks after molecular validation. 98](#_Toc193101507)

[Supplementary figure 40: The relationship matrix of Anaplasmataceae species and other vectors after molecular validation. 99](#_Toc193101508)

[Supplementary figure 41: The relationship matrix of Anaplasmataceae species and animals after molecular validation. 100](#_Toc193101509)

[Supplementary figure 42: The global distribution of the other 65 Anaplasmataceae species after molecular validation. 101](#_Toc193101510)

# **Supplementary Results**

*Symptom characteristics of different Anaplasmataceae species infections*

There were eight Anaplasmataceae species confirmed in 10 or more human cases with clinical symptoms recorded, yielding a total number of 7070 patients with symptom profiles (appendix 1 Supplementary table 20). Human granulocytic anaplasmosis (HGA) caused by *Anplasma phagocytophilum* was the most clinically reported disease in this study, influenza-like symptoms were the most common symptoms of this disease, with fever (89·6‒89·7%) and myalgia (85·7‒86·4%) being the main symptoms, followed by malaise (74·9%‒79·9%), chills (66·0‒71·2%) and fatigue (55·2‒59·0%). In addition, gastrointestinal symptoms were also common symptoms in HGA, mainly manifested as anorexia (65·0‒67·6%), diarrhea (46·3‒49·4%), vomit (35·6‒39·0%) and nausea (33·5‒39·1%). The main clinical manifestation of human monocytic ehrlichiosis (HME) caused by *Ehrlichia chaffeensis* was similar to HGA, with fever (87·7%) being the main manifestation. Fever and headache were two major symptoms for all eight species, and myalgia, malaise, nausea, fatigue, and chills were major symptoms for four, four, four, four and three species, respectively. Vomit and diarrhea were major symptoms for two species. In particular, neurological symptoms were rare.

*Ecological associations of vector tick occurrences*

The habitat suitability of vector ticks is mainly affected by environmental, ecoclimatic, and biological features. Among them, ecoclimates are the main associated factors of the HSI for most species of vector ticks, but the impacts vary within tick species (appendix 1 Supplementary table 22). For example, the suitable habitat of *Ixodes scapularis* is mainly affected by mean temperature of warmest quarter, and shows positive correlation. *Ixodes. pacificus* and *Haemaphysalis longicornis* is well-influenced by precipitation of warmest quarter, although the effects of this influence are reversed to them. *Ixodes pacificus* is well-suitable to areas with precipitation of warmest quarter below 200mm, but *Ha. longicornis* prefers areas with more than 200 mm. The distribution of *Ixodes persulcatus* is associated to leaf area index, whereas *Dermacentor marginatus* is affected by annual mean temperature and cropland area. The most predominant factor affecting the distribution of *Ixodes ricinus*, *Rhipicephalus microplus*, and *Rhipicephalus sanguineus* is annual precipitation, temperature seasonality, and min temperature of coldest month respectively.

# **Supplementary table 1: The PRISMA Checklist**

We followed the Preferred Reporting Items for Systematic Reviews and Meta-analysis (PRISMA) statement (S1 PRISMA Checklist). The protocol was registered with PROSPERO (CRD42023477378).

| **Section and Topic** | **Item #** | **Checklist item** | **Location where item is reported** |
| --- | --- | --- | --- |
| **TITLE** | | |  |
| Title | 1 | Identify the report as a systematic review. | 1 |
| **ABSTRACT** | | |  |
| Abstract | 2 | See the PRISMA 2020 for Abstracts checklist. | 2-3 |
| **INTRODUCTION** | | |  |
| Rationale | 3 | Describe the rationale for the review in the context of existing knowledge. | N/A |
| Objectives | 4 | Provide an explicit statement of the objective(s) or question(s) the review addresses. | N/A |
| **METHODS** | | |  |
| Eligibility criteria | 5 | Specify the inclusion and exclusion criteria for the review and how studies were grouped for the syntheses. | 8-9 |
| Information sources | 6 | Specify all databases, registers, websites, organisations, reference lists and other sources searched or consulted to identify studies. Specify the date when each source was last searched or consulted. | 7 |
| Search strategy | 7 | Present the full search strategies for all databases, registers and websites, including any filters and limits used. | 7 |
| Selection process | 8 | Specify the methods used to decide whether a study met the inclusion criteria of the review, including how many reviewers screened each record and each report retrieved, whether they worked independently, and if applicable, details of automation tools used in the process. | 8-9 |
| Data collection process | 9 | Specify the methods used to collect data from reports, including how many reviewers collected data from each report, whether they worked independently, any processes for obtaining or confirming data from study investigators, and if applicable, details of automation tools used in the process. | 8-9 |
| Data items | 10a | List and define all outcomes for which data were sought. Specify whether all results that were compatible with each outcome domain in each study were sought (e.g. for all measures, time points, analyses), and if not, the methods used to decide which results to collect. | 9 |
|  | 10b | List and define all other variables for which data were sought (e.g. participant and intervention characteristics, funding sources). Describe any assumptions made about any missing or unclear information. | 9 |
| Study risk of bias assessment | 11 | Specify the methods used to assess risk of bias in the included studies, including details of the tool(s) used, how many reviewers assessed each study and whether they worked independently, and if applicable, details of automation tools used in the process. | 9 |
| Effect measures | 12 | Specify for each outcome the effect measure(s) (e.g. risk ratio, mean difference) used in the synthesis or presentation of results. | N/A |
| Synthesis methods | 13a | Describe the processes used to decide which studies were eligible for each synthesis (e.g. tabulating the study intervention characteristics and comparing against the planned groups for each synthesis (item #5)). | N/A |
|  | 13b | Describe any methods required to prepare the data for presentation or synthesis, such as handling of missing summary statistics, or data conversions. | N/A |
|  | 13c | Describe any methods used to tabulate or visually display results of individual studies and syntheses. | N/A |
|  | 13d | Describe any methods used to synthesize results and provide a rationale for the choice(s). If meta-analysis was performed, describe the model(s), method(s) to identify the presence and extent of statistical heterogeneity, and software package(s) used. | N/A |
|  | 13e | Describe any methods used to explore possible causes of heterogeneity among study results (e.g. subgroup analysis, meta-regression). | N/A |
|  | 13f | Describe any sensitivity analyses conducted to assess robustness of the synthesized results. | N/A |
| Reporting bias assessment | 14 | Describe any methods used to assess risk of bias due to missing results in a synthesis (arising from reporting biases). | N/A |
| Certainty assessment | 15 | Describe any methods used to assess certainty (or confidence) in the body of evidence for an outcome. | N/A |
| **RESULTS** | | |  |
| Study selection | 16a | Describe the results of the search and selection process, from the number of records identified in the search to the number of studies included in the review, ideally using a flow diagram. | 14 |
|  | 16b | Cite studies that might appear to meet the inclusion criteria, but which were excluded, and explain why they were excluded. | N/A |
| Study characteristics | 17 | Cite each included study and present its characteristics. | N/A |
| Risk of bias in studies | 18 | Present assessments of risk of bias for each included study. | N/A |
| Results of individual studies | 19 | For all outcomes, present, for each study: (a) summary statistics for each group (where appropriate) and (b) an effect estimate and its precision (e.g. confidence/credible interval), ideally using structured tables or plots. | 14–18 |
| Results of syntheses | 20a | For each synthesis, briefly summarise the characteristics and risk of bias among contributing studies. | N/A |
|  | 20b | Present results of all statistical syntheses conducted. If meta-analysis was done, present for each the summary estimate and its precision (e.g. confidence/credible interval) and measures of statistical heterogeneity. If comparing groups, describe the direction of the effect. | 17 |
|  | 20c | Present results of all investigations of possible causes of heterogeneity among study results. | N/A |
|  | 20d | Present results of all sensitivity analyses conducted to assess the robustness of the synthesized results. | N/A |
| Reporting biases | 21 | Present assessments of risk of bias due to missing results (arising from reporting biases) for each synthesis assessed. | N/A |
| Certainty of evidence | 22 | Present assessments of certainty (or confidence) in the body of evidence for each outcome assessed. | N/A |
| **DISCUSSION** | | |  |
| Discussion | 23a | Provide a general interpretation of the results in the context of other evidence. | 19 |
|  | 23b | Discuss any limitations of the evidence included in the review. | 22-23 |
|  | 23c | Discuss any limitations of the review processes used. | 22-23 |
|  | 23d | Discuss implications of the results for practice, policy, and future research. | 23 |
| **OTHER INFORMATION** | | |  |
| Registration and protocol | 24a | Provide registration information for the review, including register name and registration number, or state that the review was not registered. | 7 |
|  | 24b | Indicate where the review protocol can be accessed, or state that a protocol was not prepared. | 7 |
|  | 24c | Describe and explain any amendments to information provided at registration or in the protocol. | 7 |
| Support | 25 | Describe sources of financial or non-financial support for the review, and the role of the funders or sponsors in the review. | N/A |
| Competing interests | 26 | Declare any competing interests of review authors. | N/A |
| Availability of data, code and other materials | 27 | Report which of the following are publicly available and where they can be found: template data collection forms; data extracted from included studies; data used for all analyses; analytic code; any other materials used in the review. | S1 File,  S2 File.  S3 File.  S4 File. |

# **Supplementary table 2: Literature search strategy for two major databases.**

| **Database** | **Search strategy** |
| --- | --- |
| PubMed | (((((((((((Anaplasmataceae[Title/Abstract]) OR (*Anaplasma*[Title/Abstract])) OR (*Ehrlichia*[Title/Abstract])) OR (*Neorickettsia*[Title/Abstract])) OR (*Wolbachia*[Title/Abstract])) OR (*Aegyptianella*[Title/Abstract])) OR (*Candidatus* Neoehrlichia[Title/Abstract])) OR (*Candidatus* Xenohaliotis[Title/Abstract])) OR (*Candidatus* Crytoplasma[Title/Abstract])) OR (*Candidatus* Mesenet[Title/Abstract])) OR (*Candidatus* Neowolbachia[Title/Abstract])) OR (*Candidatus* Xenolissocinum[Title/Abstract]) |
| Web of science | (((((((((((((((((((((((TI=(Anaplasmataceae)) OR TI=(*Anaplasma*)) OR TI=(*Ehrlichia*)) OR TI=(*Neorickettsia*)) OR TI=(*Wolbachia*)) OR TI=(*Aegyptianella*)) OR TI=(*Candidatus* Neoehrlichia)) OR TI=(*Candidatus* Xenohaliotis)) OR TI=(*Candidatus* Crytoplasma)) OR TI=(*Candidatus* Mesenet)) OR TI=(*Candidatus* Neowolbachia)) OR TI=(*Candidatus* Xenolissocinum)) OR AB=(Anaplasmataceae)) OR AB=(*Anaplasma*)) OR AB=(*Ehrlichia*)) OR AB=(*Neorickettsia*)) OR AB=(*Wolbachia*)) OR AB=(*Aegyptianella*)) OR AB=(*Candidatus* Neoehrlichia)) OR AB=(*Candidatus* Xenohaliotis)) OR AB=(*Candidatus* Crytoplasma)) OR AB=(*Candidatus* Mesenet)) OR AB=(*Candidatus* Neowolbachia)) OR AB=(*Candidatus* Xenolissocinum) |

# **Supplementary table 3: The inclusion and exclusion criteria of screening publications.**

| **Criteria** | **Guidance** | **Outcome** |
| --- | --- | --- |
| **Title/Abstract screening** |  |  |
| #1: Anaplasmataceae species | Does the Title/Abstract refer the Anaplasmataceae species which are detected from natural environment? | If Yes, remain and evaluate #2. If No, exclude. |
| #2: Tested objects | Does the Title/Abstract refer the Anaplasmataceae species which are detected from vectors, animals, or humans? | If Yes, remain and evaluate #3. If No, exclude. |
| #3: Not review | Does the Title/Abstract refer the article which is not a review? (Not presenting new primary data) | If No, remain for full text review. If Yes, exclude. |
| **Full text screening** |  |  |
| #1: Re-screening | Does the article meet the screening criteria following? 1-species belong to the family Anaplasmataceae 2-infection in natural environment 3-detected from vectors, animals, or humans 4-not drug or vaccine trials 5-not research of modelling the transmission dynamics of Anaplasmataceae species  6-not transstadial transmission research in vectors  7-not research of clinical trials of infections in animals  8-not mechanistic research at the molecular level of Anaplasmataceae species  9-not reviews | If Yes, remain and evaluate #2. If No, exclude. |
| #2: Test objects and methods | Does the article refer the specific detection objectives and methods? 1-Anaplasmataceae members identified in the detection (species level)  2-vector and animal identified in detection (species level)  3-detailed specimen used for testing (vectors or blood/spleen from animals or humans) 4-testing method for Anaplasmataceae species (molecular, isolation, microscopical or serological test) | If Yes, remain and evaluate #3. If No, exclude. |
| #3: Geographical information | Does the article provide the geographical information? 1-geographic location information at administrative division levels 2-exact locations or marked the latitude and longitude 3-explicit locations of getting infections or sampling | If Yes, remain for data set.  If No, exclude. |

# **Supplementary table 4: The laboratory tests used to detect Anaplasmataceae species in this study.**

|  | **Detection methods** |
| --- | --- |
| **Detection in vectors** | (1) molecular detection with PCR or sequencing;  (2) isolation and cultivation of Anaplasmataceae species from vectors;  (3) morphology identification under microscope. |
| **Detection in animals** | (1) molecular detection with PCR or sequencing;  (2) isolation and cultivation of Anaplasmataceae species from animals;  (3) morphology identification under microscope.  (4) serologic testing with single sera sample. |
| **Confirmed human cases** | (1) molecular detection with PCR or sequencing;  (2) isolation and cultivation of Anaplasmataceae species from humans;  (3) morphology identification under microscope;  (4) a four-fold increase or seroconversion in titer of specific antibodies in blood sera from acute phase to convalescent phase in patients with clinical diagnosis of Anaplasmataceae species infection. |

PCR, Polymerase Chain Reaction.

# **Supplementary table 5: Variables extracted from selected studies.**

| **Variable** | **Specific meaning of the variable** |
| --- | --- |
| **Literature information** |  |
| Reference ID | Unique number for the literature. |
| Article title | Article title that included in the review. |
| Authors | All Authors of the included article. |
| Publication time | Publication time of the included article. |
| Study period | The start and end year for the researches. |
| Study site | Locations of survey sampling (country, province/state, city, county, township, village, nature reserve or precise latitude/longitude coordinate information) |
| **Laboratory testing method** |  |
| Detection method | Detection methods for Anaplasmataceae species (including molecular testing, pathogen isolation and culture, and serologic testing) |
| **Basic information of Anaplasmataceae members** |  |
| Anaplasmataceae species | Standard taxonomy term of Anaplasmataceae species tested in the study. |
| **Testing information of Anaplasmataceae species in vectors and animals** |  |
| Species of vectors/animals | Information on vector/host animal species detected |
| Number of tested all | Total number of samples tested for Anaplasmataceae species |
| Number of tested positive | Number of samples tested positive for Anaplasmataceae species |
| **Testing information of Anaplasmataceae species in humans** |  |
| Study type | Case report; Case series; Surveillance. |
| Number of tested individuals | Number of individuals tested for Anaplasmataceae species. |
| Number of individuals tested positive | Number of individuals tested as positive for Anaplasmataceae species. |
| Clinical manifestation | Number of human cases reporting each of the clinical symptom |

# **Supplementary table 6: Ecological factors potentially associated with the ticks and tick-borne pathogens used in the modelling analysis.**

| Variable | Reference | Content | Method | Usage and/or result |
| --- | --- | --- | --- | --- |
| BIO1‒19 | Lin ZT, et al. [1] | *Anaplasma capra* in China | Model: ecological niche model (ENM) | Used in the *Anaplasma capra* ecological niche model. |
|  | Miao D, et al [2] | Tick in global scale  Tick-borne disease in China | Model: ecological niche model (ENM)  Model: BRT | Used in tick ecological niche model.  Used in SFTS ecological niche model. |
|  | Zhang YY, et.al. [3] | Tick-borne pathogens in global scale | Model: Logistic regression and RF | Used in tick-borne pathogens ecological niche model. |
|  | Kylie Sage et al. [5] | RFGB and tick in America | Model: MaxEnt | Used in the RFGB and tick ecological niche model. |
|  | Okely M et al [6] | Tick in global scale | Model: MaxEnt | Used in tick ecological niche model. |
|  | Abdelghafar A., et al. [7] | Tick in Europe | Model: MaxEnt | Used in the tick ecological niche model. |
|  | Lu Zhang, et.al. [8] | Tick in America | Model: MaxEnt | Precipitation of the driest month contributed 19.6% in the model. |
|  | Eisen RJ, et al. [9] | Tick in America | Model: Logistic regression, RF and MaxEnt | Precipitation of coldest quarter contributed 37.8‒70.3 in the model. |
|  | Wang SS, et.al. [10] | Tick in global scale | Model: MaxEnt | Used in the tick ecological niche model. |
| Leaf area index | Zhang YY, et.al. [3] | Tick-borne pathogens in global scale | Model: Logistic regression and RF | Used in tick-borne pathogens ecological niche model. |
|  | Fuller T, et.al. [11] | Monkeypox disease in the Congo Basin | Model: Logistic regression and MaxEnt | Used in the monkeypox ecological niche model. |
| Elevation | Lin ZT, et al. [1] | *Anaplasma capra* in China | Model: ecological niche model (ENM) | Used in the *Anaplasma capra* ecological niche model. |
|  | Miao D, et al [2] | Tick in global scale  Tick-borne disease in China | Model: ecological niche model (ENM)  Model: BRT | Used in tick ecological niche model.  Used in SFTS ecological niche model. |
|  | Zhang YY, et.al. [3] | Tick-borne pathogens in global scale | Model: Logistic regression and RF | Used in tick-borne pathogens ecological niche model. |
|  | Kylie Sage et al. [5] | RFGB and tick in America | Model: MaxEnt | Used in the RFGB and tick ecological niche model. |
| Mixed cropland and nature vegetation | Zhang YY, et.al. [3] | Tick-borne pathogens in global scale | Model: Logistic regression and RF | Used in tick-borne pathogens ecological niche model. |
|  | Allen T, et.al. [4] | Emerging zoonotic diseases in global scale | Model: Logistic regression | Land cover was used in the analysis of zoonotic pathogens ecological niche model. |
| Shrubland | Zhang YY, et.al. [3] | Tick-borne pathogens in global scale | Model: Logistic regression and RF | Used in tick-borne pathogens ecological niche model. |
|  | Allen T, et.al. [4] | Emerging zoonotic diseases in global scale | Model: Logistic regression | Land cover was used in the analysis of zoonotic pathogens ecological niche model. |
| Mixed tree, shrub and herbaceous | Zhang YY, et.al. [3] | Tick-borne pathogens in global scale | Model: Logistic regression and RF | Used in tick-borne pathogens ecological niche model. |
|  | Allen T, et.al. [4] | Emerging zoonotic diseases in global scale | Model: Logistic regression | Land cover was used in the analysis of zoonotic pathogens ecological niche model. |
| Grassland | Miao D, et al [2] | Tick in global scale  Tick-borne disease in China | Model: ecological niche model (ENM)  Model: BRT | Used in tick ecological niche model.  Used in SFTS ecological niche model. |
|  | Zhang YY, et.al. [3] | Tick-borne pathogens in global scale | Model: Logistic regression and RF | Used in tick-borne pathogens ecological niche model. |
|  | Allen T, et.al. [4] | Emerging zoonotic diseases in global scale | Model: Logistic regression | Land cover was used in the analysis of zoonotic pathogens ecological niche model. |
| Sparse vegetation land | Zhang YY, et.al. [3] | Tick-borne pathogens in global scale | Model: Logistic regression and RF | Used in tick-borne pathogens ecological niche model. |
|  | Allen T, et.al. [4] | Emerging zoonotic diseases in global scale | Model: Logistic regression | Land cover was used in the analysis of zoonotic pathogens ecological niche model. |
| Flooded vegetation | Zhang YY, et.al. [3] | Tick-borne pathogens in global scale | Model: Logistic regression and RF | Used in tick-borne pathogens ecological niche model. |
| Bare areas | Zhang YY, et.al. [3] | Tick-borne pathogens in global scale | Model: Logistic regression and RF | Used in tick-borne pathogens ecological niche model. |
| Water body | Miao D, et al [2] | Tick in global scale  Tick-borne disease in China | Model: ecological niche model (ENM)  Model: BRT | Used in tick ecological niche model.  Used in SFTS ecological niche model. |
|  | Allen T, et.al. [4] | Emerging zoonotic diseases in global scale | Model: Logistic regression | Land cover was used in the analysis of zoonotic pathogens ecological niche model. |
| Cropland | Miao D, et al [2] | Tick in global scale  Tick-borne disease in China | Model: ecological niche model (ENM)  Model: BRT | Used in tick ecological niche model.  Used in SFTS ecological niche model. |
|  | Zhang YY, et.al. [3] | Tick-borne pathogens in global scale | Model: Logistic regression and RF | Used in tick-borne pathogens ecological niche model. |
|  | Allen T, et.al. [4] | Emerging zoonotic diseases in global scale | Model: Logistic regression | Land cover was used in the analysis of zoonotic pathogens ecological niche model. |
| Urban construction land | Miao D, et al [2] | Tick in global scale  Tick-borne disease in China | Model: ecological niche model (ENM)  Model: BRT | Used in tick ecological niche model.  Used in SFTS ecological niche model. |
|  | Zhang YY, et.al. [3] | Tick-borne pathogens in global scale | Model: Logistic regression and RF | Used in tick-borne pathogens ecological niche model. |
|  | Allen T, et.al. [4] | Emerging zoonotic diseases in global scale | Model: Logistic regression | Land cover was used in the analysis of zoonotic pathogens ecological niche model. |
| Population count | Miao D, et al [2] | Tick in global scale  Tick-borne disease in China | Model: ecological niche model (ENM)  Model: BRT | Used in tick ecological niche model.  Used in SFTS ecological niche model. |
|  | Allen T, et.al. [4] | Emerging zoonotic diseases in global scale | Model: Logistic regression | Land cover was used in the analysis of zoonotic pathogens ecological niche model. |
|  | Jones KE, et al. [12] | Emerging disease in global scale | Model: Logistic regression | Used in the pathogens ecological niche model. |
| Global Downscaled GDP | Miao D, et al [2] | Tick in global scale  Tick-borne disease in China | Model: ecological niche model (ENM)  Model: BRT | Used in tick ecological niche model.  Used in SFTS ecological niche model. |
|  | Magalhães AR, et.al. [13] | Natural focal disease in global scale | Model: MaxEnt | The most significant predictive variables for relative variable importance = 37 ± 13% standard error. |
|  | Miao D, et.al. [18] | Tick and tick-borne pathogens in China | Model: Logistic regression | Used in tick ecological niche model. |
| Human Footprint | Gallardo B, et.al. [14] | The global distribution of invaders | Model: MaxEnt | Factors related to the human footprint explained a substantial amount (23% on average) of species distributions. |
|  | Skinner EB, et.al. [15] | The vector-borne diseases | Model：RF | Human footprint is an important predictor of local occurrence and that its nonlinear effects vary predictably with the transmission ecology of each vector-borne diseases |
| Rodentia richness | Usman S, et.al. [16] | Transmission dynamic of MPXV | Model: SEIR | Used in the monkeypox dynamic model. |
| Mammalian richness | Miao D, et al [2] | Tick in global scale  Tick-borne disease in China | Model: ecological niche model (ENM)  Model: BRT | Used in tick ecological niche model.  Used in SFTS ecological niche model. |
|  | Zhang YY, et.al. [3] | Tick-borne pathogens in global scale | Model: Logistic regression and RF | Used in tick-borne pathogens ecological niche model. |
|  | Olival KJ, et.al. [17] | Zoonotic spillover from mammals | Model: GAM | Used in the spillover model. |
| Density of goat | Lin ZT, et al. [1] | *Anaplasma capra* in China | Model: ecological niche model (ENM) | Used in the *Anaplasma capra* ecological niche model. |
|  | Zhang YY, et.al. [3] | Tick-borne pathogens in global scale | Model: Logistic regression and RF | Used in tick-borne pathogens ecological niche model. |
| Density of sheep | Lin ZT, et al. [1] | *Anaplasma capra* in China | Model: ecological niche model (ENM) | Used in the *Anaplasma capra* ecological niche model. |
|  | Zhang YY, et.al. [3] | Tick-borne pathogens in global scale | Model: Logistic regression and RF | Used in tick-borne pathogens ecological niche model. |
| Density of cattle | Lin ZT, et al. [1] | *Anaplasma capra* in China | Model: ecological niche model (ENM) | Used in the *Anaplasma capra* ecological niche model. |
|  | Zhang YY, et.al. [3] | Tick-borne pathogens in global scale | Model: Logistic regression and RF | Used in tick-borne pathogens ecological niche model. |

**Supplementary references:**

1 Lin ZT, et al. Epidemiological and phylogenetic characteristics of emerging *Anaplasma capra*: A systematic review with modeling analysis. *Infect Genet Evol*. 2023; 115: 105510.

2 Miao D, et al. Mapping the global potential transmission hotspots for severe fever with thrombocytopenia syndrome by machine learning methods. *Emerg Microbes Infect*. 2020; 9: 817‒826.

3 Zhang YY, et al. Mapping the global distribution of spotted fever group rickettsiae: a systematic review with modelling analysis. *Lancet Digit Health*. 2023; 5: e5‒e15.

4 Allen T, et al. Global hotspots and correlates of emerging zoonotic diseases. *Nat Commun*. 2017; 8: 1124.

5 Kylie Sage, et al. Ecological niche modeling and distribution of *Ornithodoros hermsi* associated with tick-borne relapsing fever in western North America. *PLoS Negl Trop Dis*, 2017; 11: e0006047.

6 Okely M, Al-Khalaf AA. Predicting the potential distribution of the cattle fever tick *Rhipicephalus annulatus* (Acari: Ixodidae) using ecological niche modeling. *Parasitol Res*. 2022;121(12):3467‒3476.

7 Alkishe AA, et al. Climate change influences on the potential geographic distribution of the disease vector tick *Ixodes ricinus*. *PLoS One*. 2017; 12: e0189092.

8 Lu Zhang, et al. Projecting the Potential Distribution Areas of *Ixodes scapularis* (Acari: Ixodidae) Driven by Climate Change. *Biology*. 2022; 11: 107.

9 Eisen RJ, et al. Modeling Climate Suitability of the Western Blacklegged Tick in California. *J Med Entomol*. 2018; 55: 1133-1142.

10 Wang SS, et al. Geographical distribution of *Ixodes persulcatus* and associated pathogens: Analysis of integrated data from a China field survey and global published data. *One Health*. 2023; 16: 100508.

11 Fuller T, et al. Using remote sensing to map the risk of human monkeypox virus in the Congo Basin. *Ecohealth*. 2011; 8: 14‒25.

12 Jones KE, et al. Global trends in emerging infectious diseases. *Nature*. 2008; 451: 990‒3.

13 Magalhães AR, et al. Neglected tropical diseases risk correlates with poverty and early ecosystem destruction. *Infect Dis Poverty*. 2023; 12: 32.

14 Gallardo B, et al. The importance of the human footprint in shaping the global distribution of terrestrial, freshwater and marine invaders. *PLoS One*. 2015; 10: e0125801.

15 Skinner EB, et al. Human footprint is associated with shifts in the assemblages of major vector-borne diseases. Nat Sustain. 2023; 6: 652–661.

16 Usman S, et al. Modeling the transmission dynamics of the monkeypox virus infection with treatment and vaccination interventions. *J App Math Phys*, 2017; 5: 2335.

17 Olival KJ, et al. Host and viral traits predict zoonotic spillover from mammals. *Nature*. 2017; 546: 646‒650.

18 Miao D, et al. Epidemiology and ecology of severe fever with thrombocytopenia syndrome in China, 2010‒2018. *Clin Infect Dis*. 2021; 73: e3851‒e3858.

# **Supplementary table 7: The spatial resolution, study duration and source of the included variables.**

| **Variable** | **Spatial resolution** | **Study duration** | **Source of data** | **Website** | **Reference** |
| --- | --- | --- | --- | --- | --- |
| Climate data | 0°2·5′ | 1975‒2018 | WorldClim | https://www.worldclim.org/ | Fick SE, Hijmans RJ. WorldClim 2: new 1-km spatial resolution climate surfaces for global land areas. Int. J. Climatol., 2017; 37: 4302-15.  Harris I, Jones PD, Osborn TJ, Lister DH. Updated high-resolution grids of monthly climatic observations – the CRU TS3.10 Dataset. Int. J. Climatol., 2014; 34: 623-42. |
| Leaf area index | 8km | 1981‒2019 | Resource and Environment Science and Data Center | https://www.resdc.cn/ | Yang L, Liu R, Chen JM. Retrospective retrieval of long-term consistent global leaf area index (1981-2011) from combined AVHRR and MODIS data. J Geophys Res Biogeosci, 2015; 117. |
| Land cover | 0·3km | 1992‒2019 | European Space Agency | https://maps.elie.ucl.ac.be/CCI/ | European Space Agency. ESA Land Cover Climate Change Initiative (Land_Cover_cci): Global Land Cover Maps, Version 2.0.7. https://catalogue.ceda.ac.uk/uuid/b382ebe6679d44b8b0e68ea4ef4b701c/ (accessed May 28, 2021) |
| Human Footprint | 1km | 2000‒2018 | Scientific Data | https://www.gisrsdata.com/ | Mu, Haowei; Li, Xuecao; Wen, Yanan; Huang, Jianxi; Du, Peijun; Su, Wei; et al. (2021): An annual global terrestrial Human Footprint dataset from 2000 to 2018. figshare. Figure. https://doi.org/10.6084/m9.figshare.16571064.v5 |
| Elevation | 1km | 2010 | EarthEnv (DEM90) | http://www.earthenv.org/ | Robinson N, Regetz J, Guralnick RP. EarthEnv-DEM90: A nearly-global, void-free, multi-scale smoothed, 90m digital elevation model from fused ASTER and SRTM data. ISPRS, 2014; 87: 57-67. |
| Livestock density | 1km | 2010; 2015 | Food and Agriculture Organization (FAO) | http://www.fao.org/livestock-systems/en/ | Gilbert M, Nicolas G, Cinardi G, et al. Global distribution data for cattle, buffaloes, horses, sheep, goats, pigs, chickens and ducks in 2010. Sci Data, 2018; 5: 180227. |
| Mammalian richness | 0°0′30″ | 2013 | International Union for Conservation of Nature (IUCN) | https://sedac.ciesin.columbia.edu/ | International Union for Conservation of Nature - IUCN, and Center for International Earth Science Information Network - CIESIN - Columbia University. 2015. Gridded Species Distribution: Global Mammal Richness Grids, 2015 Release. Palisades, NY: NASA Socioeconomic Data and Applications Center (SEDAC). |
| Rodent richness | 10km | 2018 | BiodiversityMapping.org | https://biodiversitymapping.org/index.php/permissions/ | Jenkins, C.N. & K. Van Houtan. (2016). Global and regional priorities for marine biodiversity protection. Pimm, SL, CN Jenkins, R Abell, TM Brooks, JL Gittleman, LN Joppa, PH Raven, CM Roberts, JO Sexton (2014) The biodiversity of species and their rates of extinction, distribution, and protection. Science 344(6187): 1246752. |
| Population number | 1km | 2020 | WorldPop 2020 | https://www.worldpop.org/ | WorldPop. Population counts, unconstrained global mosaics 2000-2020 (1 km resolution), 2020. https://www.worldpop.org/geodata/listing?id=64/ (accessed Apr 12, 2021). |
| Global downscaled GDP | 0°2·5′ | 1990, 2025 | NASA Socioeconomic Data and Applications Center (SEDAC) | https://sedac.ciesin.columbia.edu/ | Gaffin SR, Xing X, Yetman G. Global 15 x 15 Minute Grids of the Downscaled GDP Based on the SRES B2 Scenario, 1990 and 2025. Palisades, NY: NASA Socioeconomic Data and Applications Center (SEDAC); 2004. |

GDP, gross domestic product.

# **Supplementary table 8: Variables used for ecological modelling in this study.**

| **Classification of variable** | **Variable** | **Description** |
| --- | --- | --- |
| **Ecoclimatic variables** |  |  |
|  | BIO1 | Annual mean temperature (℃) |
|  | BIO2 | Mean diurnal range (Mean of monthly (max temp-min temp)) (℃) |
|  | BIO3 | Isothermality (BIO2/ BIO7) (×100) |
|  | BIO4 | Temperature seasonality (standard deviation×100) |
|  | BIO5 | Max temperature of warmest month (℃) |
|  | BIO6 | Min temperature of coldest month (℃) |
|  | BIO7 | Annual range of temperature (BIO5- BIO6) (℃) |
|  | BIO8 | Mean temperature of wettest quarter (℃) |
|  | BIO9 | Mean temperature of driest quarter (℃) |
|  | BIO10 | Mean temperature of warmest quarter (℃) |
|  | BIO11 | Mean temperature of coldest quarter (℃) |
|  | BIO12 | Annual precipitation (mm) |
|  | BIO13 | Precipitation of wettest month (mm) |
|  | BIO14 | Precipitation of driest month (mm) |
|  | BIO15 | Precipitation seasonality (Coefficient of variation) |
|  | BIO16 | Precipitation of wettest quarter (mm) |
|  | BIO17 | Precipitation of driest quarter (mm) |
|  | BIO18 | Precipitation of warmest quarter (mm) |
|  | BIO19 | Precipitation of coldest quarter (mm) |
| **Environmental variables** |  |  |
|  | Elevation | Average elevation (m) |
|  | Leaf area index | area of leaves (m²) over a unit of land (m²) |
|  | Cropland | Percentage coverage of cropland (%) |
|  | Mixed cropland and nature vegetation | Percentage coverage of mixed cropland and nature vegetation (%) |
|  | Forest | Percentage coverage of forest (%) |
|  | Shrubland | Percentage coverage of shrubland (%) |
|  | Mixed tree, shrub and herbaceous | Percentage coverage of mixed tree, shrub and herbaceous (%) |
|  | Grassland | Percentage coverage of grassland (%) |
|  | Lichens and mosses | Percentage coverage of lichens and mosses (%) |
|  | Sparse vegetation land | Percentage coverage of sparse vegetation land (%) |
|  | Flooded vegetation | Percentage coverage of flooded vegetation (%) |
|  | Urban built-up land | Percentage coverage of urban construction land (%) |
|  | Bare areas | Percentage coverage of bare areas (%) |
|  | Water body | Percentage coverage of inland water body (%) |
|  | Ice and snow | Percentage coverage of ice and snow (%) |
| **Biological variables** |  |  |
|  | Buffalo | Density of buffalo (heads per km²) |
|  | Cattle | Density of cattle (heads per km²) |
|  | Goat | Density of goat (heads per km²) |
|  | Sheep | Density of sheep (heads per km²) |
|  | Horse | Density of horse (heads per km²) |
|  | Mammalian richness* | The number of mammal species per km² |
|  | Rodent richness | The number of rodent species per km² |
| **Socioeconomic variables** |  |  |
|  | Population number | Average of population counts per km² |
|  | Global downscaled GDP | The assessment of GDP per raster |
|  | Human Footprint^#^ | The annual dynamics of the global human footprint per km² |

* These datasets were extracted from the NASA Socioeconomic Data and Applications Center (SEDAC) Gridded Species Distribution collection created from vector data files acquired from the International Union for Conservation of Nature (IUCN) Red List collection. The data represent the species of mammals at one kilometer resolution.

^#^ The dataset was extracted from the studies published on Scientific Data. The annual dynamics of the global human footprint from 2000 to 2018 were mapped with the Mollweide equal-area projection at 1km resolution, using eight variables including built environments, population density, nighttime light, croplands, pasture lands, roadways, railways, and navigable waterways as inputs, which reflect different aspects of human pressures. GDP, gross domestic product.

# **Supplementary table 9: Variables on the occurrence of Anaplasmataceae species infections and 44 ecoclimatic, environmental, biological, and socioeconomic covariates used in this study.**

| **Classification of variables** | **Description of variable** | **Used in this study** | **Detailed information** |
| --- | --- | --- | --- |
| Anaplasmataceae species (15 variables) | Literature information (6 variables)  Laboratory test methods (1 variable)  Basic information of Anaplasmataceae species (1 variable)  Detection information in vectors/animals (3 variables)  Detection information in human beings (4 variables) | Clinical spectrum  Geolocalization  Distribution analyses  Modeling analysis (outcome) | appendix 1 Supplementary table 5 |
| Environmental variables (15 variables) | Elevation, leaf area index, cropland, mixed cropland and nature vegetation, forest, shrubland, mixed tree/shrub and herbaceous, grassland, lichens and mosses, sparse vegetation land, flooded vegetation, urban built-up land, bare areas, water body, and ice and snow | Modeling analysis (predictors) | appendix 1 Supplementary table 8 |
| Ecoclimatic variables (19 variables) | BIO1-BIO19 (19 variables) | Modeling analysis (predictors) | appendix 1 Supplementary table 8 |
| Biological variables (7 variables) | Buffalo, cattle, goat, sheep, horse, mammalian richness, rodent richness | Modeling analysis (predictors) | appendix 1 Supplementary table 8 |
| Socioeconomic variables (3 variables) | Population number, global downscaled GDP, and human footprint | Modeling analysis (predictors) | appendix 1 Supplementary table 8 |

**Supplementary table 10: The information on the location occurrences at different scales of all** **Anaplasmataceae species.**

| Anaplasmataceae species | Number of all occurrence locations | Number of locations occurrence (area less than 900 km^2^) | Number of locations occurrence (area less than 400 km^2^) | Number of locations occurrence (area less than 100 km^2^) |
| --- | --- | --- | --- | --- |
| *A. phagocytophilum** | 1565 | 1007 | 953 | 850 |
| *A. marginale** | 574 | 289 | 273 | 242 |
| *E. canis** | 407 | 223 | 202 | 165 |
| *A. ovis** | 393 | 186 | 171 | 164 |
| *A. platys** | 366 | 176 | 162 | 127 |
| *A. bovis* | 246 | 76 | 70 | 65 |
| *Candidatus* N. mikurensis* | 234 | 167 | 162 | 152 |
| *E. chaffeensis** | 221 | 109 | 101 | 87 |
| *A. centrale* | 103 | 58 | 55 | 53 |
| *A. capra* | 90 | 21 | 21 | 21 |
| *E. ewingii* | 88 | 46 | 46 | 39 |
| *E. ruminantium* | 87 | 50 | 46 | 43 |
| *E. muris* | 58 | 31 | 28 | 22 |
| *W. pipientis* | 57 | 33 | 29 | 15 |
| *E. minasensis* | 24 | 7 | 7 | 7 |
| *N. risticii* | 23 | 15 | 10 | 4 |
| Panola Mountain *Ehrlichia* | 18 | 8 | 8 | 6 |
| *Anaplasma* sp. 'Omatjenne' | 15 | 11 | 10 | 10 |
| *Candidatus* A. camelii | 12 | 4 | 3 | 3 |
| *Candidatus* A. boleense | 11 | 1 | 1 | 1 |
| *Candidatus* E. urmitei | 10 | 4 | 3 | 2 |
| *Candidatus* E. rustica | 7 | 2 | 1 | - |
| *Candidatus* E. khabarensis | 5 | 2 | 2 | 2 |
| *N. sennetsu* | 5 | 3 | 3 | 3 |
| *Candidatus* E. occidentalis | 4 | 4 | 4 | 3 |
| *Candidatus* E. ornithorhynchi | 4 | 1 | 1 | 1 |
| *Candidatus* E. pampeana | 4 | 3 | 3 | 3 |
| *Candidatus* N. lotoris | 4 | 1 | 1 | 1 |
| *E. walkeri* | 4 | 4 | 4 | 4 |
| *Ehrlichia* sp. HF | 4 | 2 | 2 | 1 |
| *A. odocoilei* | 3 | - | - | - |
| *Ae. pullorum* | 3 | 1 | 1 | 1 |
| *Candidatus* A. brasiliensis | 3 | 2 | 2 | 2 |
| *Candidatus* E. regneryi | 3 | - | - | - |
| *Candidatus* E. shimanensis | 3 | - | - | - |
| *Candidatus* N. arcana | 3 | - | - | - |
| *Candidatus* N. australis | 3 | - | - | - |
| *Candidatus* Neoehrlichia sp. (FU98) | 3 | 1 | 1 | 1 |
| *Candidatus* W. massiliensis | 3 | 1 | - | - |
| *Ehrlichia* sp. HF565 | 3 | - | - | - |
| *N. helminthoeca* | 3 | - | - | - |
| *Candidatus* A. africae | 2 | 2 | 2 | 2 |
| *Candidatus* A. amazonensis | 2 | - | - | - |
| *Candidatus* A. dongdaense | 2 | - | - | - |
| *Candidatus* A. gabonensis | 2 | 1 | 1 | - |
| *Candidatus* E. dumleri | 2 | 2 | 2 | 2 |
| *Candidatus* M. longicola | 2 | 2 | 2 | 2 |
| *Candidatus* N. chilensis | 2 | 1 | 1 | 1 |
| *Candidatus* X. californiensis | 2 | - | - | - |
| *E. ovina* | 2 | - | - | - |
| *Ehrlichia* sp. BL157-9 | 2 | 1 | 1 | 1 |
| *Ehrlichia* sp. Yonaguni138 | 2 | 1 | 1 | 1 |
| *A. mesaeterum* | 1 | 1 | 1 | - |
| *Ae. ranarum* | 1 | 1 | 1 | 1 |
| *Anaplasma* sp. 'Mongolian' | 1 | - | - | - |
| *Anaplasma* sp. HLJ-14 | 1 | 1 | 1 | 1 |
| *Anaplasma* sp. Mymensingh | 1 | 1 | 1 | 1 |
| *Candidatus* A. cinensis | 1 | 1 | 1 | 1 |
| *Candidatus* A. corsicanum | 1 | 1 | 1 | 1 |
| *Candidatus* A. ivorensis | 1 | - | - | - |
| *Candidatus* A. mediterraneum | 1 | 1 | 1 | 1 |
| *Candidatus* A. pangolinii | 1 | - | - | - |
| *Candidatus* A. rodmosense | 1 | - | - | - |
| *Candidatus* A. sparouinense | 1 | - | - | - |
| *Candidatus* A. sphenisci | 1 | 1 | 1 | 1 |
| *Candidatus* A. testudinis | 1 | - | - | - |
| *Candidatus* A. turritanum | 1 | 1 | 1 | 1 |
| *Candidatus* A. zhouzhiense | 1 | - | - | - |
| *Candidatus* Al. californiense | 1 | - | - | - |
| *Candidatus* E. andalusi | 1 | - | - | - |
| *Candidatus* E. corsicanum | 1 | 1 | 1 | 1 |
| *Candidatus* E. hainanensis | 1 | - | - | - |
| *Candidatus* E. hydrochoerus | 1 | 1 | 1 | 1 |
| *Candidatus* E. ovata | 1 | 1 | 1 | - |
| *Candidatus* E. senegalensis | 1 | - | - | - |
| *Candidatus* E. walkerii | 1 | - | - | - |
| *Candidatus* E. zunyiensis | 1 | - | - | - |
| *Candidatus* W. ivorensis | 1 | - | - | - |
| *Ehrlichia* sp. 360 | 1 | - | - | - |
| *Ehrlichia* sp. H7 | 1 | - | - | - |
| *Ehrlichia* sp. Tibet | 1 | - | - | - |
| *Ehrlichia* sp. Yamaguchi | 1 | 1 | 1 | 1 |
| *Ehrlichia* sp. clone SY36 | 1 | 1 | 1 | 1 |
| *Ehrlichia* sp. hc-hlj209 | 1 | - | - | - |
| *N. finleia* | 1 | - | - | - |

* Seven major species of family Anaplasmataceae were included in the niche model based on the number of publications and occurrence locations in the database (the number of locations with an area of less than 900 km^2^ was greater than 100).

**Supplementary table 11: The information on the location occurrences at different scales of all** **Anaplasmataceae species after molecular validation.**

| Anaplasmataceae species | Number of all occurrence locations | Number of locations occurrence (area less than 900 km2) | Number of locations occurrence (area less than 400 km2) | Number of locations occurrence (area less than 100 km2) |
| --- | --- | --- | --- | --- |
| *A. phagocytophilum** | 878 | 532 | 502 | 445 |
| *A. marginale** | 403 | 197 | 188 | 167 |
| *A. ovis** | 326 | 155 | 144 | 138 |
| *A. platys** | 165 | 94 | 87 | 71 |
| *Candidatus* N. mikurensis* | 163 | 111 | 107 | 101 |
| *E. canis** | 146 | 86 | 78 | 65 |
| *A. bovis* | 112 | - | - | - |
| *E. chaffeensis* | 100 | 24 | 20 | 15 |
| *A. capra* | 72 | - | - | - |
| *E. ruminantium* | 69 | - | - | - |
| *A. centrale* | 47 | - | - | - |
| *E. ewingii* | 30 | - | - | - |
| *W. pipientis* | 27 | - | - | - |
| *E. muris* | 25 | - | - | - |
| *E. minasensis* | 14 | - | - | - |
| *Candidatus* A. boleense | 11 | - | - | - |
| Panola Mountain *Ehrlichia* | 11 | - | - | - |
| *Candidatus* A. camelii | 10 | - | - | - |
| *Candidatus* E. urmitei | 9 | - | - | - |
| *Candidatus* E. rustica | 7 | - | - | - |
| *Candidatus* E. khabarensis | 5 | - | - | - |
| *N. sennetsu* | 5 | - | - | - |
| *Candidatus* E. occidentalis | 4 | - | - | - |
| *Candidatus* E. pampeana | 4 | - | - | - |
| *Candidatus* N. lotoris | 4 | - | - | - |
| *E. walkeri* | 4 | - | - | - |
| *N. risticii* | 4 | - | - | - |
| *Anaplasma* sp. 'Omatjenne' | 3 | - | - | - |
| *Candidatus* A. brasiliensis | 3 | - | - | - |
| *Candidatus* E. ornithorhynchi | 3 | - | - | - |
| *Candidatus* Neoehrlichia sp. (FU98) | 3 | - | - | - |
| *Ehrlichia* sp. HF | 3 | - | - | - |
| *Ehrlichia* sp. HF565 | 3 | - | - | - |
| *A. odocoilei* | 2 | - | - | - |
| *Ae. pullorum* | 2 | - | - | - |
| *Candidatus* A. africae | 2 | - | - | - |
| *Candidatus* A. dongdaense | 2 | - | - | - |
| *Candidatus* E. dumleri | 2 | - | - | - |
| *Candidatus* E. regneryi | 2 | - | - | - |
| *Candidatus* E. shimanensis | 2 | - | - | - |
| *Ehrlichia* sp. Yonaguni138 | 2 | - | - | - |
| *N. helminthoeca* | 2 | - | - | - |
| *A. capra*-like | 1 | - | - | - |
| *Anaplasma* sp. 'Mongolian' | 1 | - | - | - |
| *Anaplasma* sp. HLJ-14 | 1 | - | - | - |
| *Anaplasma* sp. Mymensingh | 1 | - | - | - |
| *Candidatus* A. amazonensis | 1 | - | - | - |
| *Candidatus* A. cinensis | 1 | - | - | - |
| *Candidatus* A. corsicanum | 1 | - | - | - |
| *Candidatus* A. ivorensis | 1 | - | - | - |
| *Candidatus* A. mediterraneum | 1 | - | - | - |
| *Candidatus* A. rodmosense | 1 | - | - | - |
| *Candidatus* A. sparouinense | 1 | - | - | - |
| *Candidatus* A. sphenisci | 1 | - | - | - |
| *Candidatus* A. turritanum | 1 | - | - | - |
| *Candidatus* A. zhouzhiense | 1 | - | - | - |
| *Candidatus* Al. californiense | 1 | - | - | - |
| *Candidatus* E. andalusi | 1 | - | - | - |
| *Candidatus* E. corsicanum | 1 | - | - | - |
| *Candidatus* E. hainanensis | 1 | - | - | - |
| *Candidatus* E. hydrochoerus | 1 | - | - | - |
| *Candidatus* E. ovata | 1 | - | - | - |
| *Candidatus* E. senegalensis | 1 | - | - | - |
| *Candidatus* E. walkerii | 1 | - | - | - |
| *Candidatus* E. zunyiensis | 1 | - | - | - |
| *Candidatus* N. arcana | 1 | - | - | - |
| *Candidatus* N. australis | 1 | - | - | - |
| *Candidatus* N. chilensis | 1 | - | - | - |
| *Candidatus* W. ivorensis | 1 | - | - | - |
| *Ehrlichia* sp. 360 | 1 | - | - | - |
| *Ehrlichia* sp. BL157-9 | 1 | - | - | - |
| *Ehrlichia* sp. H7 | 1 | - | - | - |
| *Ehrlichia* sp. Tibet | 1 | - | - | - |
| *Ehrlichia* sp. Yamaguchi | 1 | - | - | - |
| *Ehrlichia* sp. clone SY36 | 1 | - | - | - |
| *Ehrlichia* sp. hc-hlj209 | 1 | - | - | - |
| *W. massiliensis* | 1 | - | - | - |

* Six major species of family Anaplasmataceae were included in the niche model based on the number of publications and occurrence locations in the database (the number of locations with an area of less than 900 km^2^ was greater than 80).

# **Supplementary table 12: Meta-analysis of six major Anaplasmataceae species in ticks.**

| Anaplasmataceae species | Ticks | K | n | N | I^2^ (%) | Common effect model positive rate (95% CI) | Random effects model positive rate (95% CI) | Positive rate (%) |
| --- | --- | --- | --- | --- | --- | --- | --- | --- |
| *A. phagocytophilum* | *I. ricinus** | 179 | 10552 | 196475 | 97·7 | 5·37 (5·27, 5·47) | 4·55 (3·90, 5·32) | 4·55 |
|  | *I. scapularis** | 48 | 7526 | 139837 | 98·9 | 5·38 (5·26, 5·50) | 6·64 (4·76, 9·18) | 6·64 |
|  | *Ha. punctata* | 3 | 71 | 470 | 96·2 | 15·11 (12·15, 18·64) | 4·77 (0·56, 30·99) | 4·77 |
|  | *Am. variegatum* | 1 | 5 | 203 | - | 2·46 (-) | 2·46 (-) | 2·46 |
|  | *I. pacificus** | 13 | 272 | 14730 | 97·2 | 1·85 (1·64, 2·08) | 2·01 (1·21, 3·30) | 2·01 |
|  | *D. reticulatus* | 11 | 96 | 4266 | 92·3 | 2·25 (1·85, 2·74) | 1·54 (0·63, 3·73) | 1·54 |
|  | *I. pavlovskyi* | 2 | 20 | 955 | 86·6 | 2·09 (1·35, 3·22) | 1·04 (0·13, 7·71) | 1·04 |
|  | *I. persulcatus** | 16 | 523 | 9487 | 94·4 | 5·51 (5·07, 5·99) | 6·95 (4·89, 9·78) | 6·95 |
|  | *I. hexagonus* | 5 | 55 | 1814 | 75·2 | 3·03 (2·33, 3·93) | 2·15 (0·86, 5·25) | 3·03 |
|  | *Am. americanum* | 3 | 14 | 3122 | 88·8 | 0·45 (0·27, 0·76) | 0·55 (0·15, 1·97) | 0·55 |
|  | *Ha. leporispalustris* | 1 | 4 | 375 | - | 1·07 (-) | 1·07 (-) | 1·07 |
|  | *D. silvarum* | 3 | 55 | 748 | 97·6 | 7·35 (5·69, 9·46) | 5·82 (1·05, 26·46) | 5·82 |
|  | *Ha. longicornis** | 16 | 264 | 10737 | 95·7 | 2·46 (2·18, 2·77) | 2·59 (1·29, 5·10) | 2·59 |
|  | *Rh. sanguineus* | 3 | 21 | 795 | 89 | 2·64 (1·73, 4·02) | 1·85 (0·45, 7·28) | 1·85 |
|  | *Rh. turanicus* | 2 | 53 | 605 | 95·8 | 8·76 (6·75, 11·29) | 9·75 (3·81, 22·77) | 9·75 |
|  | *I. dammini* | 1 | 3 | 760 | - | 0·39 (-) | 0·39 (-) | 0·39 |
|  | *Am. flavomaculatum* | 1 | 2 | 125 | - | 1·60 (-) | 1·60 (-) | 1·60 |
|  | *I. ventalloi* | 1 | 3 | 144 | - | 2·08 (-) | 2·08 (-) | 2·08 |
|  | *Hy. marginatum* | 2 | 54 | 635 | 98·1 | 8·50 (6·57, 10·94) | 5·04 (0·12, 70·64) | 5·04 |
|  | *D. marginatus* | 3 | 62 | 470 | 97·7 | 13·19 (10·42, 16·56) | 7·98 (0·97, 43·45) | 7·98 |
|  | *Hy. lusitanicum* | 1 | 23 | 795 | - | 2·89 (-) | 2·89 (-) | 2·89 |
|  | *D. occidentalis* | 2 | 5 | 1323 | 0·0 | 0·38 (0·16, 0·90) | 0·38 (0·16, 0·90) | 0·38 |
|  | *I. nipponensis* | 1 | 1 | 380 | - | 0·26 (-) | 0·26 (-) | 0·26 |
|  | *Ha. concinna* | 1 | 1 | 106 | - | 0·94 (-) | 0·94 (-) | 0·94 |
|  | *Ha. qinghaiensis* | 2 | 49 | 384 | 89·3 | 12·76 (9·78, 16·49) | 13·31 (7·36, 22·90) | 13·31 |
|  | *Rh. microplus* | 1 | 7 | 216 | - | 3·24 (-) | 3·24 (-) | 3·24 |
|  | *Rh. pumilio* | 1 | 18 | 519 | - | 3·47 (-) | 3·47 (-) | 3·47 |
|  | *Hy. aegyptium* | 1 | 26 | 191 | - | 13·61 (-) | 13·61 (-) | 13·61 |
|  | *D. andersoni* | 2 | 4 | 335 | 0·0 | 1·19 (0·45, 3·14) | 1·19 (0·45, 3·14) | 1·19 |
|  | *I. simplex* | 1 | 2 | 138 | - | 1·45 (-) | 1·45 (-) | 1·45 |
|  | *D. nuttalli* | 1 | 8 | 110 | - | 7·27 (-) | 7·27 (-) | 7·27 |
|  | *D. variabilis* | 1 | 28 | 5853 | - | 0·48 (-) | 0·48 (-) | 0·48 |
|  | *Ha. formosensis* | 1 | 25 | 715 | - | 3·50 (-) | 3·50 (-) | 3·50 |
| *Candidatus* N. mikurensis | *D. reticulatus* | 2 | 2 | 1601 | 0·0 | 0·09 (0·00, 0·34) | 0·09 (0·00, 0·34) | 0·09 |
|  | *I. pavlovskyi* | 2 | 10 | 988 | 81·1 | 0·88 (0·36, 1·58) | 0·81 (0·00, 2·68) | 0·81 |
|  | *I. ricinus** | 42 | 1935 | 39753 | 96·5 | 4·15 (3·96, 4·36) | 4·43 (3·33, 5·68) | 4·43 |
|  | *I. hexagonus* | 2 | 19 | 303 | 0·0 | 6·27 (3·75, 9·33) | 6·27 (3·75, 9·33) | 6·27 |
|  | *Ha. concinna* | 1 | 2 | 187 | - | 1·07 (-) | 1·07 (-) | 1·07 |
|  | *I. persulcatus* | 4 | 17 | 1339 | 6·9 | 1·15 (0·60, 1·83) | 1·23 (0·57, 2·09) | 1·15 |
|  | *I. ovatus* | 1 | 4 | 164 | - | 2·44 (-) | 2·44 (-) | 2·44 |
|  | *Am. cohaerens* | 1 | 2 | 241 | - | 0·83 (-) | 0·83 (-) | 0·83 |
|  | *D. nuttalli* | 1 | 1 | 310 | - | 0·32 (-) | 0·32 (-) | 0·32 |
|  | *I. holocyclus* | 1 | 36 | 460 | - | 7·83 (-) | 7·83 (-) | 7·83 |
| *E. canis* | *Rh. sanguineus** | 22 | 834 | 12192 | 95 | 6·84 (6·41, 7·3) | 4·43 (2·56, 7·55) | 4·43 |
|  | *Rh. bursa* | 2 | 2 | 356 | 0·0 | 0·56 (0·14, 2·22) | 0·56 (0·14, 2·22) | 0·56 |
|  | *D. marginatus* | 1 | 4 | 2011 | - | 0·20 (-) | 0·20 (-) | 0·20 |
|  | *I. canisuga* | 1 | 3 | 2011 | - | 0·15 (-) | 0·15 (-) | 0·15 |
|  | *Ha. longicornis** | 3 | 145 | 2245 | 98·8 | 6·46 (5·51, 7·55) | 2·46 (0·28, 18·37) | 2·46 |
|  | *I. ricinus* | 3 | 9 | 7481 | 12·1 | 0·12 (0·06, 0·23) | 0·12 (0·06, 0·23) | 0·12 |
|  | *Rh. turanicus* | 1 | 1 | 169 | - | 0·59 (-) | 0·59 (-) | 0·59 |
|  | *I. scapularis* | 1 | 40 | 231 | - | 17·32 (-) | 17·32 (-) | 17·32 |
|  | *I. dammini* | 1 | 60 | 609 | - | 9·85 (-) | 9·85 (-) | 9·85 |
|  | *Rh. microplus** | 4 | 105 | 964 | 96·5 | 10·89 (9·08, 13·02) | 6·14 (1·01, 29·51) | 6·14 |
|  | *Rh. pravus* | 1 | 13 | 195 | - | 6·67 (-) | 6·67 (-) | 6·67 |
| *A. ovis* | *Rh. bursa* | 2 | 2 | 358 | 0·0 | 0·56 (0·14, 2·21) | 0·56 (0·14, 2·21) | 0·56 |
|  | *Rh. turanicus* | 2 | 28 | 276 | 85·3 | 10·14 (7·10, 14·30) | 8·02 (3·04, 19·53) | 8·02 |
|  | *Ha. longicornis* | 2 | 64 | 1022 | 97·5 | 6·26 (4·93, 7·92) | 5·71 (1·57, 18·71) | 5·71 |
|  | *Hy. anatolicum* | 2 | 33 | 215 | 0·0 | 15·35 (11·12, 20·81) | 15·35 (11·12, 20·81) | 15·35 |
|  | *Rh. sanguineus* | 4 | 87 | 549 | 85·2 | 15·85 (13·03, 19·15) | 16·71 (10·53, 25·49) | 16·71 |
|  | *D. marginatus** | 3 | 110 | 454 | 97·8 | 24·23 (20·51, 28·38) | 23·69 (7·27, 55·13) | 23·69 |
|  | *Rh. microplus* | 1 | 8 | 276 | - | 2·90 (-) | 2·90 (-) | 2·90 |
|  | *D. abaensis* | 1 | 9 | 104 | - | 8·65 (-) | 8·65 (-) | 8·65 |
|  | *D. nuttalli* | 2 | 44 | 506 | 96·6 | 8·70 (6·53, 11·48) | 7·64 (1·85, 26·63) | 7·64 |
|  | *Ha. qinghaiensis* | 1 | 18 | 164 | - | 10·98 (-) | 10·98 (-) | 10·98 |
|  | *Rh. decoloratus* | 1 | 1 | 101 | - | 0·99 (-) | 0·99 (-) | 0·99 |
| *A. platys* | *Rh. sanguineus** | 17 | 359 | 6167 | 97·5 | 20·45 (18·75, 22·29) | 3·54 (1·88, 6·68) | 3·54 |
|  | *I. persulcatus* | 1 | 4 | 137 | - | 2·92 (-) | 2·92 (-) | 2·92 |
|  | *Ha. longicornis** | 2 | 164 | 2038 | 99·2 | 12·91 (11·22, 14·85) | 8·49 (1·42, 50·97) | 8·49 |
|  | *Ha. flava* | 1 | 11 | 175 | - | 6·29 (-) | 6·29 (-) | 6·29 |
|  | *I. ovatus* | 1 | 4 | 1211 | - | 0·33 (-) | 0·33 (-) | 0·33 |
|  | *Rh. microplus* | 4 | 38 | 1137 | 86·3 | 6·26 (4·61, 8·51) | 1·19 (0·23, 6·15) | 1·19 |
|  | *Rh. annulatus* | 1 | 1 | 120 | - | 0·83 (-) | 0·83 (-) | 0·83 |
|  | *Rh. pravus* | 1 | 52 | 236 | - | 22·03 (-) | 22·03 (-) | 22·03 |
|  | *I. ricinus* | 1 | 1 | 151 | - | 0·66 (-) | 0·66 (-) | 0·66 |
| *A. marginale* | *Am. lepidum* | 1 | 9 | 181 | - | 4·97 (-) | 4·97 (-) | 4·97 |
|  | *Am. variegatum* | 1 | 5 | 203 | - | 2·46 (-) | 2·46 (-) | 2·46 |
|  | *Hy. anatolicum* | 3 | 45 | 745 | 97 | 8·75 (6·88,11·32) | 6·04 (3·12, 8·86) | 6·04 |
|  | *Hy. detritum* | 1 | 9 | 207 | - | 4·35 (-) | 4·35 (-) | 4·35 |
|  | *Hy. dromedarii* | 1 | 14 | 390 | - | 3·59 (-) | 3·59 (-) | 3·59 |
|  | *Rh. haemaphysaloides* | 1 | 6 | 110 | - | 5·45 (-) | 5·45 (-) | 5·45 |
|  | *Rh. microplus** | 12 | 212 | 2764 | 88·9 | 10·32 (9·10, 11·71) | 7·50 (4·62, 12·18) | 7·50 |
|  | *Rh. sanguineus* | 2 | 10 | 345 | 42·0 | 3·18 (1·73, 5·84) | 3·06 (1·36, 6·89) | 3·18 |
|  | *Rh. turanicus* | 1 | 42 | 169 | - | 24·85 (-) | 24·85 (-) | 24·85 |
|  | *Rh. evertsi* | 1 | 20 | 143 | - | 13·99 (-) | 13·99 (-) | 13·99 |
|  | *Hy. marginatum* | 1 | 37 | 106 | - | 34·91 (-) | 34·91 (-) | 34·91 |
|  | *Hy. excavatum* | 1 | 40 | 114 | - | 35·09 (-) | 35·09 (-) | 35·09 |
|  | *Rh. bursa* | 2 | 8 | 309 | 0·0 | 2·70 (1·36, 5·34) | 2·70 (1·36, 5·34) | 2·70 |

*Based on the results of the meta-analysis of tick species infected by the six major Anaplasmataceae species, ticks with more than two article reports, more than 100 positive tests, and a positivity rate of more than 2 0% were identified as the major tick species of the Anaplasmataceae species, and eight species of ticks were finally determined to be the major vectors of the six Anaplasmataceae species and included in the ecological niche model.

k, n, N represents the number of articles, the number of tick positives, and the total number of tested ticks, respectively.

# **Supplementary table 13: The number of studies and occurrence locations for the three tick species.**

| Tick species | Number of studies collected | Number of studies included | Number of locations collected | Number of locations used in modelling analysis |
| --- | --- | --- | --- | --- |
| *Dermacentor marginatus* | 782 | 74 | 2606 | 1284 |
| *Rhipicephalus microplus* | 3226 | 166 | 3185 | 2727 |
| *Rhipicephalus sanguineus* | 2933 | 347 | 2036 | 1716 |

# **Supplementary table 14: The references for all the 85 Anaplasmataceae species.**

| **Anaplasmataceae species** | **Number of literatures (n)** | **Reference ID** |
| --- | --- | --- |
| *Anaplasma phagocytophilum* | 1264 | 2, 6, 11, 23, 24, 34, 42, 48, 52, 54, 55, 56, 69, 75, 81, 83, 93, 96, 106, 107, 109, 112, 116, 117, 120, 121, 129, 132, 135, 140, 149, 150, 154, 159, 160, 164, 165, 166, 169, 173, 177, 194, 195, 203, 222, 226, 227, 235, 237, 239, 247, 249, 250, 252, 253, 265, 268, 269, 273, 275, 283, 290, 292, 302, 303, 315, 316, 324, 329, 331, 332, 343, 346, 349, 352, 353, 362, 367, 371, 372, 376, 380, 381, 383, 385, 399, 401, 403, 410, 415, 417, 427, 429, 431, 441, 447, 460, 464, 468, 470, 475, 478, 479, 482, 487, 500, 505, 508, 512, 516, 517, 518, 528, 535, 536, 542, 549, 551, 552, 554, 560, 562, 566, 570, 571, 574, 581, 583, 592, 593, 602, 613, 614, 616, 623, 625, 626, 627, 629, 634, 635, 636, 638, 639, 642, 645, 646, 650, 654, 658, 659, 662, 663, 664, 673, 678, 684, 685, 690, 695, 697, 700, 701, 703, 704, 705, 707, 709, 710, 722, 727, 728, 730, 731, 744, 746, 748, 749, 750, 754, 759, 760, 766, 769, 770, 775, 778, 782, 783, 784, 788, 793, 800, 804, 805, 811, 812, 815, 817, 823, 825, 826, 834, 840, 849, 853, 854, 856, 867, 870, 883, 887, 888, 889, 890, 900, 912, 914, 924, 928, 934, 939, 945, 946, 958, 961, 969, 974, 975, 976, 980, 985, 988, 997, 998, 1005, 1006, 1014, 1015, 1027, 1031, 1033, 1034, 1036, 1055, 1056, 1057, 1058, 1060, 1063, 1064, 1065, 1070, 1073, 1074, 1077, 1083, 1087, 1090, 1092, 1093, 1095, 1099, 1100, 1107, 1110, 1111, 1124, 1125, 1135, 1140, 1142, 1146, 1148, 1149, 1150, 1160, 1171, 1174, 1180, 1181, 1182, 1184, 1185, 1186, 1188, 1189, 1190, 1192, 1208, 1210, 1217, 1218, 1221, 1223, 1249, 1253, 1258, 1263, 1264, 1268, 1269, 1270, 1273, 1274, 1279, 1286, 1293, 1295, 1298, 1300, 1302, 1304, 1305, 1307, 1308, 1315, 1317, 1325, 1327, 1328, 1329, 1331, 1335, 1342, 1346, 1349, 1350, 1354, 1358, 1359, 1360, 1362, 1363, 1369, 1372, 1375, 1376, 1378, 1381, 1382, 1383, 1384, 1387, 1388, 1390, 1394, 1397, 1399, 1400, 1401, 1402, 1405, 1406, 1411, 1413, 1421, 1423, 1425, 1429, 1430, 1431, 1433, 1437, 1439, 1440, 1441, 1446, 1447, 1450, 1452, 1462, 1463, 1465, 1467, 1473, 1474, 1475, 1478, 1481, 1482, 1484, 1488, 1489, 1490, 1492, 1502, 1506, 1508, 1510, 1526, 1528, 1530, 1535, 1538, 1540, 1541, 1545, 1546, 1548, 1552, 1553, 1554, 1556, 1557, 1559, 1560, 1561, 1563, 1565, 1566, 1569, 1570, 1571, 1574, 1579, 1584, 1585, 1587, 1592, 1594, 1595, 1597, 1598, 1600, 1603, 1612, 1617, 1618, 1625, 1627, 1631, 1633, 1635, 1638, 1643, 1650, 1660, 1661, 1662, 1663, 1669, 1671, 1672, 1674, 1685, 1686, 1687, 1695, 1697, 1702, 1703, 1705, 1706, 1708, 1710, 1714, 1715, 1718, 1720, 1721, 1723, 1725, 1727, 1728, 1743, 1747, 1748, 1760, 1761, 1764, 1768, 1772, 1773, 1776, 1779, 1780, 1781, 1788, 1795, 1796, 1798, 1802, 1805, 1807, 1811, 1813, 1817, 1818, 1821, 1823, 1832, 1835, 1838, 1846, 1847, 1851, 1852, 1853, 1854, 1871, 1872, 1874, 1889, 1894, 1902, 1911, 1912, 1915, 1921, 1925, 1928, 1944, 1949, 1956, 1957, 1958, 1962, 1965, 1974, 1975, 1986, 1987, 1989, 1993, 1999, 2005, 2007, 2008, 2012, 2035, 2038, 2039, 2042, 2050, 2053, 2059, 2062, 2064, 2065, 2068, 2073, 2075, 2078, 2123, 2127, 2160, 2179, 2207, 2216, 2218, 2226, 2228, 2236, 2239, 2245, 2248, 2250, 2251, 2257, 2263, 2266, 2277, 2279, 2280, 2282, 2283, 2288, 2291, 2293, 2294, 2298, 2305, 2306, 2317, 2320, 2321, 2331, 2333, 2335, 2337, 2345, 2355, 2359, 2364, 2368, 2377, 2379, 2380, 2381, 2382, 2383, 2385, 2386, 2387, 2393, 2394, 2397, 2398, 2399, 2400, 2402, 2406, 2411, 2415, 2419, 2421, 2436, 2438, 2446, 2450, 2452, 2454, 2455, 2459, 2462, 2464, 2465, 2466, 2467, 2472, 2478, 2486, 2487, 2489, 2490, 2491, 2493, 2494, 2496, 2498, 2499, 2500, 2503, 2505, 2507, 2508, 2516, 2521, 2524, 2525, 2542, 2551, 2561, 2570, 2578, 2591, 2603, 4, 5, 9, 13, 15, 27, 29, 33, 39, 44, 53, 66, 67, 76, 77, 87, 97, 105, 110, 111, 113, 124, 126, 127, 131, 155, 167, 168, 171, 174, 180, 182, 183, 186, 199, 205, 207, 208, 218, 219, 220, 221, 241, 246, 254, 256, 258, 260, 262, 266, 278, 280, 284, 286, 287, 291, 297, 307, 309, 327, 337, 344, 345, 358, 370, 386, 395, 406, 411, 412, 413, 414, 420, 423, 434, 439, 440, 442, 444, 446, 449, 455, 456, 463, 486, 490, 498, 503, 506, 507, 513, 515, 532, 540, 543, 561, 563, 564, 565, 567, 575, 594, 595, 600, 609, 617, 637, 643, 651, 652, 674, 681, 687, 689, 698, 702, 708, 713, 717, 718, 719, 720, 721, 724, 726, 732, 738, 753, 755, 756, 762, 791, 792, 797, 798, 802, 806, 809, 810, 814, 816, 818, 833, 837, 845, 846, 852, 857, 858, 868, 872, 874, 880, 884, 892, 903, 905, 911, 916, 919, 926, 937, 940, 944, 947, 949, 979, 984, 987, 989, 996, 1008, 1010, 1013, 1028, 1029, 1049, 1059, 1061, 1072, 1082, 1088, 1098, 1101, 1108, 1109, 1115, 1116, 1118, 1128, 1139, 1151, 1154, 1156, 1157, 1159, 1162, 1163, 1165, 1166, 1167, 1169, 1170, 1176, 1191, 1193, 1194, 1196, 1197, 1199, 1203, 1204, 1206, 1207, 1211, 1237, 1243, 1247, 1250, 1252, 1254, 1260, 1265, 1275, 1276, 1281, 1282, 1284, 1285, 1287, 1288, 1292, 1294, 1296, 1297, 1299, 1301, 1306, 1309, 1316, 1319, 1340, 1341, 1347, 1351, 1356, 1367, 1370, 1371, 1393, 1398, 1407, 1408, 1417, 1432, 1434, 1436, 1443, 1444, 1449, 1451, 1454, 1455, 1457, 1469, 1483, 1493, 1495, 1497, 1498, 1499, 1500, 1515, 1517, 1519, 1524, 1534, 1543, 1555, 1562, 1577, 1590, 1596, 1611, 1613, 1615, 1626, 1629, 1636, 1648, 1654, 1657, 1667, 1675, 1676, 1677, 1684, 1693, 1698, 1709, 1711, 1716, 1719, 1722, 1724, 1733, 1734, 1749, 1750, 1751, 1754, 1755, 1770, 1775, 1778, 1782, 1786, 1787, 1808, 1809, 1820, 1829, 1830, 1837, 1841, 1848, 1850, 1856, 1859, 1869, 1879, 1887, 1888, 1890, 1891, 1895, 1898, 1899, 1900, 1901, 1913, 1926, 1936, 1943, 1950, 1953, 1955, 1976, 1990, 1995, 1997, 1998, 2004, 2022, 2027, 2030, 2036, 2055, 2057, 2083, 2092, 2095, 2167, 2193, 2215, 2240, 2243, 2249, 2267, 2278, 2299, 2303, 2304, 2307, 2318, 2330, 2339, 2340, 2349, 2353, 2374, 2376, 2390, 2401, 2412, 2422, 2427, 2437, 2442, 2443, 2448, 2482, 2484, 2485, 2497, 2502, 2510, 2513, 2515, 2520, 2533, 2539, 2550, 2552, 2553, 2559, 2587, 2590, 17, 18, 19, 25, 64, 142, 143, 146, 151, 152, 197, 198, 204, 257, 270, 277, 320, 333, 351, 363, 365, 369, 396, 421, 436, 451, 454, 458, 483, 484, 485, 539, 545, 556, 557, 589, 596, 601, 604, 615, 618, 715, 743, 747, 758, 771, 831, 882, 893, 894, 913, 955, 960, 964, 968, 982, 1004, 1068, 1078, 1089, 1091, 1106, 1120, 1129, 1130, 1161, 1178, 1236, 1238, 1262, 1267, 1313, 1322, 1326, 1338, 1373, 1380, 1389, 1412, 1414, 1426, 1427, 1468, 1479, 1491, 1504, 1505, 1547, 1549, 1550, 1572, 1575, 1576, 1578, 1588, 1605, 1606, 1616, 1620, 1624, 1628, 1656, 1658, 1659, 1683, 1729, 1730, 1735, 1736, 1737, 1738, 1762, 1763, 1765, 1766, 1767, 1777, 1784, 1785, 1799, 1825, 1849, 1855, 1862, 1865, 1870, 1875, 1877, 1884, 1885, 1886, 1906, 1907, 1917, 1919, 1927, 1929, 1930, 1931, 1932, 1933, 1938, 1939, 1948, 1964, 1977, 1991, 1994, 2010, 2011, 2013, 2029, 2032, 2033, 2040, 2045, 2046, 2049, 2060, 2061, 2067, 2070, 2080, 2085, 2094, 2101, 2128, 2213, 2253, 2273, 2313, 2362, 2365, 2366, 2395, 2396, 2445, 2469, 2470, 2476, 2480, 2492, 2512, 2514, 2529, 2536, 2537, 2543, 2562, 2568, 2571, 2573, 2580, 2583, 2592, 2593, 2181, 2182, 2183, 2184, 2185, 2186, 2187, 2188, 2189, 2190, 2191, 2192 |
| *Ehrlichia canis* | 518 | 1, 3, 8, 10, 26, 45, 57, 59, 70, 80, 82, 99, 104, 116, 117, 119, 124, 128, 130, 132, 135, 163, 176, 178, 209, 211, 215, 216, 229, 231, 232, 238, 242, 251, 253, 272, 274, 275, 276, 296, 301, 308, 321, 331, 332, 336, 357, 359, 360, 372, 374, 379, 397, 426, 428, 432, 433, 435, 441, 443, 459, 461, 466, 467, 468, 493, 494, 496, 509, 512, 519, 521, 524, 527, 544, 546, 547, 552, 553, 558, 559, 584, 605, 606, 610, 624, 661, 665, 667, 670, 686, 693, 699, 701, 714, 716, 736, 737, 740, 742, 745, 761, 763, 772, 776, 786, 788, 789, 795, 796, 804, 812, 815, 823, 824, 825, 828, 835, 836, 838, 843, 847, 861, 863, 864, 870, 871, 878, 883, 886, 895, 897, 901, 907, 921, 922, 925, 930, 932, 933, 939, 952, 953, 954, 958, 959, 962, 965, 971, 974, 975, 983, 985, 994, 1001, 1002, 1006, 1014, 1016, 1018, 1022, 1023, 1032, 1037, 1038, 1039, 1041, 1042, 1043, 1044, 1052, 1062, 1065, 1067, 1069, 1071, 1075, 1079, 1087, 1095, 1117, 1134, 1138, 1141, 1142, 1144, 1168, 1179, 1180, 1181, 1182, 1185, 1188, 1190, 1195, 1200, 1204, 1209, 1225, 1226, 1227, 1229, 1230, 1234, 1235, 1239, 1240, 1245, 1246, 1248, 1257, 1259, 1269, 1272, 1273, 1283, 1303, 1321, 1327, 1331, 1336, 1342, 1343, 1344, 1349, 1357, 1363, 1364, 1368, 1374, 1376, 1377, 1378, 1381, 1384, 1387, 1388, 1396, 1403, 1419, 1430, 1453, 1456, 1459, 1460, 1461, 1463, 1465, 1480, 1492, 1501, 1507, 1508, 1509, 1511, 1516, 1520, 1521, 1523, 1528, 1532, 1537, 1551, 1554, 1567, 1580, 1583, 1587, 1589, 1592, 1602, 1603, 1610, 1621, 1630, 1637, 1642, 1652, 1655, 1662, 1664, 1668, 1670, 1681, 1696, 1699, 1703, 1704, 1707, 1715, 1727, 1728, 1731, 1745, 1758, 1760, 1764, 1769, 1771, 1773, 1788, 1791, 1794, 1800, 1803, 1806, 1812, 1824, 1827, 1831, 1832, 1835, 1836, 1845, 1851, 1861, 1882, 1894, 1896, 1905, 1910, 1946, 1952, 1957, 1959, 1960, 1967, 1988, 1989, 1996, 2002, 2015, 2017, 2018, 2019, 2020, 2031, 2041, 2044, 2052, 2078, 2087, 2088, 2097, 2104, 2108, 2109, 2120, 2127, 2129, 2135, 2159, 2164, 2199, 2202, 2220, 2223, 2225, 2229, 2233, 2234, 2235, 2237, 2241, 2244, 2246, 2248, 2252, 2254, 2255, 2258, 2259, 2262, 2264, 2265, 2269, 2271, 2272, 2274, 2276, 2286, 2293, 2295, 2302, 2306, 2308, 2309, 2310, 2324, 2332, 2333, 2341, 2346, 2358, 2364, 2370, 2373, 2381, 2382, 2389, 2391, 2399, 2408, 2417, 2428, 2430, 2432, 2435, 2440, 2446, 2451, 2460, 2468, 2471, 2473, 2477, 2478, 2488, 2490, 2491, 2504, 2509, 2522, 2523, 2526, 2527, 2528, 2534, 2535, 2538, 2544, 2549, 2554, 2556, 2557, 2565, 2566, 2584, 2589, 2594, 2595, 2596, 2604, 7, 39, 41, 43, 44, 62, 65, 78, 127, 175, 263, 285, 305, 334, 341, 388, 391, 438, 541, 569, 582, 672, 724, 765, 794, 833, 857, 905, 978, 1028, 1088, 1187, 1250, 1251, 1252, 1277, 1320, 1332, 1334, 1435, 1444, 1458, 1518, 1740, 1954, 2092, 2119, 2211, 2221, 2238, 2327, 2424, 2444, 2453, 364, 801, 827, 1738, 2112, 2114, 2118, 2121, 2122, 2128, 2130, 2131, 2132, 2133, 2136, 2137, 2140, 2141, 2153, 2162, 2601 |
| *Anaplasma marginale* | 373 | 28, 31, 32, 49, 50, 51, 52, 58, 63, 68, 72, 79, 83, 85, 86, 88, 90, 91, 94, 100, 108, 114, 117, 144, 147, 161, 162, 181, 187, 193, 201, 210, 223, 224, 228, 230, 239, 243, 244, 248, 252, 255, 259, 261, 264, 271, 279, 294, 296, 299, 310, 312, 314, 325, 326, 328, 342, 347, 350, 354, 355, 356, 361, 377, 378, 398, 402, 408, 422, 429, 430, 437, 445, 453, 464, 469, 474, 477, 480, 492, 495, 516, 521, 524, 534, 538, 555, 577, 578, 599, 603, 622, 623, 628, 631, 632, 633, 640, 644, 657, 668, 669, 673, 675, 677, 680, 686, 704, 705, 711, 773, 777, 780, 782, 787, 794, 808, 822, 829, 830, 832, 850, 898, 899, 904, 912, 917, 923, 924, 943, 948, 956, 972, 973, 992, 997, 1007, 1009, 1020, 1024, 1027, 1030, 1035, 1045, 1053, 1076, 1086, 1097, 1122, 1123, 1127, 1132, 1133, 1136, 1150, 1153, 1172, 1177, 1183, 1198, 1205, 1212, 1219, 1220, 1233, 1256, 1271, 1278, 1314, 1318, 1330, 1345, 1348, 1350, 1353, 1361, 1366, 1379, 1416, 1424, 1431, 1448, 1464, 1470, 1472, 1513, 1529, 1531, 1539, 1544, 1595, 1604, 1607, 1614, 1631, 1645, 1646, 1647, 1674, 1690, 1695, 1717, 1741, 1743, 1752, 1796, 1797, 1805, 1811, 1814, 1815, 1816, 1819, 1826, 1828, 1857, 1858, 1860, 1864, 1876, 1918, 1924, 1934, 1961, 1982, 2014, 2028, 2037, 2043, 2051, 2056, 2066, 2069, 2071, 2098, 2105, 2115, 2116, 2117, 2126, 2134, 2138, 2142, 2144, 2145, 2146, 2147, 2148, 2149, 2150, 2151, 2152, 2154, 2155, 2156, 2157, 2163, 2170, 2203, 2222, 2227, 2230, 2236, 2242, 2256, 2257, 2266, 2268, 2285, 2286, 2288, 2290, 2292, 2296, 2305, 2311, 2316, 2326, 2336, 2342, 2343, 2344, 2360, 2361, 2363, 2367, 2375, 2377, 2378, 2384, 2388, 2392, 2413, 2414, 2418, 2429, 2431, 2433, 2434, 2441, 2456, 2457, 2458, 2463, 2474, 2475, 2479, 2495, 2511, 2531, 2532, 2540, 2541, 2555, 2564, 2567, 2574, 2585, 2586, 2588, 2597, 2602, 14, 38, 43, 60, 62, 65, 77, 89, 92, 115, 122, 127, 196, 200, 213, 219, 232, 233, 289, 327, 335, 382, 392, 406, 440, 472, 525, 568, 681, 724, 765, 851, 865, 884, 926, 936, 1029, 1173, 1204, 1265, 1266, 1352, 1593, 1653, 2247, 2261, 2281, 2307, 2314, 2453, 2553 |
| *Anaplasma platys* | 276 | 3, 42, 51, 59, 61, 63, 72, 80, 82, 83, 99, 117, 128, 132, 135, 176, 188, 209, 215, 216, 225, 228, 230, 232, 237, 240, 242, 243, 248, 251, 253, 255, 265, 274, 293, 296, 301, 314, 317, 328, 331, 332, 350, 353, 372, 378, 384, 397, 405, 409, 419, 426, 435, 437, 441, 443, 453, 459, 481, 491, 493, 495, 496, 514, 516, 519, 521, 524, 526, 527, 544, 547, 584, 610, 656, 667, 691, 704, 714, 733, 734, 735, 737, 742, 744, 751, 767, 772, 776, 784, 786, 789, 796, 819, 836, 841, 842, 847, 860, 862, 870, 901, 902, 906, 907, 908, 912, 922, 925, 930, 932, 935, 939, 952, 958, 971, 972, 985, 986, 994, 1002, 1003, 1011, 1015, 1016, 1019, 1023, 1037, 1041, 1042, 1084, 1087, 1094, 1104, 1112, 1113, 1137, 1141, 1144, 1147, 1150, 1152, 1158, 1168, 1198, 1200, 1227, 1228, 1229, 1231, 1240, 1242, 1244, 1280, 1283, 1303, 1312, 1337, 1350, 1355, 1357, 1396, 1501, 1507, 1511, 1512, 1516, 1583, 1589, 1610, 1631, 1637, 1655, 1673, 1691, 1726, 1728, 1742, 1773, 1801, 1806, 1835, 1892, 1894, 1941, 1942, 1947, 1957, 1963, 1967, 2002, 2054, 2076, 2077, 2135, 2199, 2212, 2214, 2218, 2220, 2235, 2246, 2252, 2264, 2266, 2269, 2272, 2286, 2287, 2302, 2305, 2306, 2310, 2315, 2324, 2328, 2329, 2338, 2341, 2347, 2350, 2407, 2417, 2428, 2451, 2491, 2501, 2518, 2547, 2556, 38, 60, 65, 78, 97, 133, 136, 231, 233, 289, 341, 388, 391, 402, 407, 425, 472, 510, 520, 597, 614, 671, 763, 764, 765, 807, 833, 884, 905, 1139, 1143, 1151, 1252, 1334, 1435, 1458, 1786, 1840, 1883, 1970, 2221, 2247, 2307, 2444, 1102, 1215 |
| *Ehrlichia chaffeensis* | 257 | 12, 81, 135, 177, 211, 318, 418, 419, 470, 511, 630, 686, 813, 878, 910, 950, 1054, 1065, 1077, 1087, 1095, 1218, 1257, 1259, 1269, 1325, 1342, 1349, 1350, 1360, 1377, 1473, 1479, 1501, 1542, 1568, 1586, 1641, 1686, 1703, 1759, 1773, 1788, 1796, 1813, 1853, 1861, 1871, 1880, 1903, 1910, 1916, 1923, 1931, 1957, 1968, 1971, 1973, 1980, 1981, 2000, 2006, 2018, 2026, 2035, 2039, 2041, 2050, 2063, 2078, 2086, 2239, 2331, 2345, 2356, 2386, 2402, 2460, 2473, 2486, 2496, 2506, 2582, 62, 127, 172, 280, 289, 295, 462, 681, 694, 774, 802, 820, 869, 879, 891, 967, 1046, 1114, 1121, 1164, 1167, 1175, 1196, 1252, 1422, 1428, 1485, 1486, 1514, 1518, 1522, 1525, 1536, 1558, 1609, 1651, 1681, 1756, 1808, 1834, 1839, 1841, 1879, 1922, 1969, 1972, 1978, 1983, 1992, 1998, 2024, 2034, 2224, 2303, 2334, 2390, 2453, 2481, 2502, 2519, 2545, 2550, 2575, 118, 156, 282, 339, 364, 368, 550, 556, 618, 620, 621, 756, 803, 882, 885, 966, 977, 1040, 1085, 1103, 1178, 1298, 1323, 1389, 1410, 1418, 1427, 1550, 1581, 1608, 1640, 1688, 1689, 1713, 1736, 1738, 1746, 1783, 1784, 1833, 1875, 1877, 1881, 1904, 1909, 1914, 1920, 1930, 1937, 1939, 1940, 1948, 1951, 1985, 2001, 2003, 2009, 2010, 2013, 2016, 2021, 2025, 2046, 2047, 2048, 2049, 2058, 2067, 2070, 2072, 2074, 2079, 2081, 2082, 2090, 2091, 2093, 2096, 2100, 2102, 2103, 2107, 2111, 2125, 2217, 2231, 2260, 2273, 2300, 2301, 2325, 2352, 2365, 2372, 2395, 2403, 2447, 2461, 2470, 2548, 2563, 2569, 2572, 2576, 2577, 2579, 2592, 2598, 2600, 2183, 2195 |
| *Anaplasma ovis* | 150 | 22, 32, 40, 42, 49, 55, 69, 74, 83, 84, 127, 134, 140, 145, 148, 149, 177, 179, 202, 227, 238, 245, 252, 255, 268, 290, 303, 310, 312, 313, 319, 350, 352, 353, 375, 376, 380, 385, 400, 402, 403, 471, 473, 493, 499, 500, 504, 518, 524, 591, 607, 647, 653, 654, 666, 685, 705, 769, 779, 781, 786, 794, 817, 821, 849, 853, 881, 889, 900, 912, 927, 930, 1009, 1031, 1034, 1047, 1066, 1090, 1125, 1150, 1198, 1204, 1214, 1224, 1280, 1354, 1411, 1471, 1488, 1595, 1619, 1631, 1643, 1717, 1732, 1811, 2084, 2089, 2175, 2205, 2216, 2236, 2250, 2266, 2289, 2292, 2322, 2336, 2355, 2378, 2405, 2409, 2410, 2415, 2423, 2458, 2546, 5, 43, 62, 81, 200, 219, 263, 327, 366, 382, 393, 503, 515, 590, 611, 641, 681, 712, 785, 798, 855, 1088, 1299, 1420, 1499, 1582, 2247, 2307, 2339, 2369, 2424, 2453, 2165 |
| *Anaplasma bovis* | 140 | 11, 42, 63, 69, 95, 130, 136, 160, 177, 202, 227, 250, 252, 268, 296, 314, 328, 348, 380, 385, 403, 404, 452, 478, 500, 501, 516, 518, 562, 588, 654, 659, 660, 661, 664, 677, 678, 685, 686, 696, 723, 769, 778, 786, 790, 793, 800, 821, 832, 853, 856, 890, 900, 909, 914, 920, 924, 930, 980, 981, 990, 997, 1020, 1021, 1034, 1110, 1125, 1145, 1212, 1248, 1350, 1354, 1355, 1405, 1438, 1440, 1470, 1472, 1496, 1513, 1586, 1635, 1712, 1748, 2106, 2208, 2216, 2266, 2294, 2305, 2404, 2406, 2483, 2581, 5, 123, 170, 190, 219, 305, 373, 444, 503, 506, 568, 595, 637, 688, 719, 752, 765, 792, 884, 903, 944, 947, 1013, 1048, 1173, 1241, 1243, 1281, 1404, 1435, 1442, 1495, 1499, 1514, 1711, 1869, 2232, 2270, 2303, 2307, 2339, 2348, 2401, 2502, 20, 470 |
| *Candidatus* Neoehrlichia mikurensis | 117 | 138, 195, 470, 482, 566, 574, 634, 650, 859, 870, 875, 942, 975, 998, 1099, 1100, 1131, 1289, 1293, 1310, 1311, 1339, 1365, 1391, 1395, 1476, 1478, 1694, 1721, 1744, 1868, 2161, 2312, 2439, 27, 30, 97, 111, 205, 206, 286, 288, 330, 394, 411, 434, 439, 442, 489, 543, 548, 575, 576, 580, 609, 616, 674, 681, 706, 713, 717, 719, 720, 757, 806, 818, 840, 845, 846, 858, 868, 874, 884, 903, 911, 937, 993, 995, 999, 1012, 1082, 1101, 1105, 1116, 1118, 1128, 1156, 1176, 1202, 1213, 1216, 1232, 1240, 1294, 1297, 1333, 1392, 1639, 2299, 2503, 16, 101, 157, 300, 306, 450, 739, 913, 1080, 1081, 1096, 1201, 1291, 1477, 1494, 1503, 1527 |
| *Anaplasma centrale* | 67 | 54, 161, 189, 223, 243, 255, 296, 310, 312, 328, 516, 521, 523, 524, 603, 623, 628, 657, 680, 686, 705, 777, 778, 832, 856, 914, 924, 972, 980, 997, 1009, 1020, 1027, 1076, 1150, 1198, 1400, 1405, 1424, 1431, 1470, 1513, 1644, 1645, 1741, 1748, 2210, 2263, 2290, 2292, 2377, 2378, 2406, 2458, 2541, 38, 327, 472, 595, 724, 936, 1013, 1173, 1301, 2307, 2401, 2502 |
| *Ehrlichia ruminantium* | 65 | 35, 238, 261, 352, 389, 497, 499, 521, 529, 537, 607, 612, 653, 679, 686, 786, 787, 924, 963, 972, 1027, 1076, 1255, 1280, 1409, 1415, 1466, 1601, 1665, 1680, 1804, 1843, 1844, 1863, 1878, 2204, 2416, 2477, 38, 73, 213, 214, 263, 281, 288, 295, 311, 390, 724, 855, 877, 916, 918, 926, 936, 1173, 1261, 1386, 1623, 1692, 1792, 2275, 2281, 2449, 1793 |
| *Ehrlichia ewingii* | 64 | 135, 153, 457, 522, 950, 1050, 1054, 1065, 1087, 1259, 1331, 1377, 1501, 1564, 1573, 1666, 1681, 1773, 1794, 1880, 1894, 1916, 2018, 2113, 2391, 2473, 136, 170, 200, 234, 448, 462, 694, 764, 774, 820, 866, 869, 879, 967, 1046, 1114, 1121, 1164, 1165, 1167, 1252, 1422, 1428, 1522, 1525, 1756, 1808, 1841, 1842, 1922, 1984, 2224, 2481, 882, 977, 1126, 1833, 2001 |
| *Anaplasma capra* | 47 | 21, 32, 42, 54, 125, 139, 140, 160, 177, 192, 227, 237, 268, 294, 304, 317, 343, 367, 403, 465, 475, 500, 501, 531, 587, 608, 613, 647, 678, 685, 723, 729, 2219, 2266, 2305, 43, 65, 219, 305, 444, 503, 520, 595, 816, 929, 991, 1025 |
| *Ehrlichia muris* | 34 | 371, 875, 1324, 1478, 1678, 1679, 1694, 1789, 1861, 2196, 307, 358, 407, 619, 681, 724, 757, 809, 846, 903, 1048, 1309, 1356, 1454, 1701, 1711, 1775, 1822, 1869, 1936, 2519, 1026, 1412, 1996 |
| *Neorickettsia risticii* | 27 | 36, 340, 598, 799, 873, 1387, 1622, 1662, 1866, 1897, 1945, 1957, 1979, 1986, 1988, 2023, 2099, 2109, 2110, 2124, 2200, 2351, 2357, 2420, 2425, 2517, 2128 |
| *Wolbachia pipientis* | 23 | 502, 1119, 1649, 1867, 2560, 38, 137, 236, 425, 488, 572, 655, 896, 915, 1017, 1222, 1290, 1356, 1458, 1893, 2168, 2198, 2558 |
| *Ehrlichia minasensis* | 19 | 47, 184, 243, 244, 272, 437, 787, 2171, 37, 43, 60, 62, 281, 289, 406, 472, 476, 876, 2354 |
| Panola Mountain *Ehrlichia* | 8 | 1739, 2180, 774, 938, 967, 1114, 2172, 2173 |
| *Anaplasma* sp. 'Omatjenne' | 7 | 296, 686, 705, 787, 924, 997, 1513 |
| *Candidatus* Anaplasma boleense | 6 | 43, 62, 366, 590, 765, 884 |
| *Candidatus* Anaplasma camelii | 5 | 295, 387, 416, 725, 1041 |
| *Neorickettsia sennetsu* | 5 | 1599, 957, 1051, 2128, 2201 |
| *Candidatus* Ehrlichia urmitei | 5 | 38, 402, 406, 798, 936 |
| *Candidatus* Ehrlichia regneryi | 4 | 35, 134, 295, 1041 |
| *Candidatus* Neoehrlichia arcana | 4 | 298, 322, 323, 941 |
| *Candidatus* Neoehrlichia australis | 4 | 298, 322, 323, 941 |
| *Ehrlichia* sp. HF | 4 | 488, 683, 1309, 1677 |
| *Candidatus* Ehrlichia shimanensis | 3 | 153, 1748, 2401 |
| *Neorickettsia helminthoeca* | 3 | 318, 2197, 2297 |
| *Anaplasma odocoilei* | 3 | 511, 2169, 774 |
| *Candidatus* Ehrlichia ornithorhynchi | 3 | 530, 586, 322 |
| *Candidatus* Neoehrlichia sp. (FU98) | 3 | 844, 1000, 2605 |
| *Ehrlichia ovina* | 3 | 2106, 2209, 1173 |
| *Candidatus* Wolbachia massiliensis | 3 | 2284, 2298, 2178 |
| *Candidatus* Anaplasma brasiliensis | 2 | 98, 2194 |
| *Candidatus* Ehrlichia khabarensis | 2 | 338, 2179 |
| *Candidatus* Neoehrlichia chilensis | 2 | 579, 573 |
| *Candidatus* Neoehrlichia lotoris | 2 | 673, 1632 |
| *Candidatus Xenohaliotis californiensis* | 2 | 682, 1966 |
| *Aegyptianella pullorum* | 2 | 2143, 2206 |
| *Candidatus* Anaplasma amazonensis | 2 | 2160, 2194 |
| *Candidatus* Ehrlichia rustica | 2 | 38, 936 |
| *Candidatus* Ehrlichia occidentalis | 2 | 267, 752 |
| *Ehrlichia* sp. BL157-9 | 2 | 765, 839 |
| *Ehrlichia* sp. Yonaguni138 | 2 | 765, 2401 |
| *Candidatus* Anaplasma cinensis | 1 | 2 |
| *Candidatus* Anaplasma turritanum | 1 | 2 |
| *Candidatus* Ehrlichia zunyiensis | 1 | 46 |
| *Candidatus* Ehrlichia dumleri | 1 | 98 |
| *Candidatus* Ehrlichia hydrochoerus | 1 | 102 |
| *Candidatus* Mesenet longicola | 1 | 141 |
| *Candidatus* Ehrlichia pampeana | 1 | 158 |
| *Candidatus* Anaplasma gabonensis | 1 | 185 |
| *Candidatus* Anaplasma testudinis | 1 | 212 |
| *Candidatus* Ehrlichia hainanensis | 1 | 217 |
| *Anaplasma* sp. 'Mongolian' | 1 | 394 |
| *Candidatus* Ehrlichia senegalensis | 1 | 424 |
| *Ehrlichia* sp. H7 | 1 | 523 |
| *Candidatus* Anaplasma africae | 1 | 524 |
| *Candidatus* Anaplasma sphenisci | 1 | 585 |
| *Candidatus* Anaplasma dongdaense | 1 | 647 |
| *Candidatus* Anaplasma zhouzhiense | 1 | 647 |
| *Anaplasma* sp. Mymensingh | 1 | 677 |
| *Candidatus* Anaplasma corsicanum | 1 | 794 |
| *Candidatus* Anaplasma mediterraneum | 1 | 794 |
| *Candidatus* Anaplasma pangolinii | 1 | 909 |
| *Aegyptianella ranarum* | 1 | 2174 |
| *Anaplasma mesaeterum* | 1 | 2175 |
| *Neorickettsia finleia* | 1 | 2177 |
| *Candidatus* Ehrlichia corsicanum | 1 | 406 |
| *Anaplasma* sp. HLJ-14 | 1 | 765 |
| *Ehrlichia* sp. clone SY36 | 1 | 765 |
| *Candidatus* Anaplasma rodmosense | 1 | 884 |
| *Ehrlichia* sp. hc-hlj209 | 1 | 903 |
| *Candidatus* Anaplasma ivorensis | 1 | 936 |
| *Candidatus* Wolbachia ivorensis | 1 | 936 |
| *Candidatus* Allocryptoplasma californiense | 1 | 1072 |
| *Ehrlichia* sp. Yamaguchi | 1 | 1309 |
| *Ehrlichia* sp. 360 | 1 | 1591 |
| *Ehrlichia* sp. HF565 | 1 | 1591 |
| *Ehrlichia walkeri* | 1 | 1790 |
| *Candidatus* Ehrlichia walkerii | 1 | 1873 |
| *Ehrlichia* sp. Tibet | 1 | 1935 |
| *Candidatus* Ehrlichia andalusi | 1 | 2166 |
| *Candidatus* Ehrlichia ovata | 1 | 2176 |
| *Candidatus* Anplasma sparouinense | 1 | 103 |

# **Supplementary table 15: The evidence for vectors carrying Anaplasmataceae** **species are capable of biting humans.**

| Vector | Species | Reference |
| --- | --- | --- |
| Tick | *Amblyomma testudinarium* | Chao L L, Lu C W, Lin Y F, et al. Molecular and morphological identification of a human biting tick, *Amblyomma testudinarium* (Acari: Ixodidae), in Taiwan[J]. Exp Appl Acarol, 2017, 71(4): 401-414. |
|  | *Amblyomma flavomaculatum* | Eisen L. Tick species infesting humans in the United States. Ticks Tick Borne Dis. 2022;13(6):102025. |
|  | *Amblyomma triguttatum* | Graves SR, Stenos J. Tick-borne infectious diseases in Australia. Med J Aust 2017; 206(7): 320-4. |
|  | *Amblyomma sparsum* | Guglielmone, AA, Robbins, R.G. Tick species found feeding on humans.2018. |
|  | *Amblyomma geoemydae* | Kadosaka, T, Fujita, H, Goto, I. et al. B33 Human tick-bite by *Amblyomma geoemydae* in Ishigakijima Island of Okinawa Prefecture, Japan. Medical Entomology and Zoology 2006; 57, 71. |
|  | *Amblyomma nitidum* | Kim J, Joo HS, Moon HJ, et al. A case of *Amblyomma testudinarium* tick bite in a Korean woman. The Korean Journal of Parasitology. 2010; 48(4):313-317. |
|  | *Amblyomma americanum* | Madison-Antenucci S, Kramer LD, Gebhardt LL, Kauffman E. Emerging tick-borne diseases. Clin Microbiol Rev 2020; 33(2). |
|  | *Amblyomma parvum* | Monje LD, Fernandez C, Percara A. Detection of *Ehrlichia* sp. strain San Luis and *Candidatus Rickettsia andeanae* in *Amblyomma parvum* ticks. Ticks Tick Borne 2019; 10(1): 111-4. |
|  | *Amblyomma mixtum* | Novakova M, Literak I, Chevez L, et al. Rickettsial infections in ticks from reptiles, birds and humans in Honduras. Ticks Tick Borne Dis 2015; 6(6): 737-42. |
|  | *Amblyomma variegatum* | Petney TN, Horak IG, Rechav Y. The ecology of the African vectors of heartwater, with particular reference to *Amblyomma hebraeum* and *Amblyomma variegatum*. Onderstepoort J Vet Res 1987; 54(3): 381-95. |
|  | *Amblyomma hebraeum* | Petney TN, Horak IG, Rechav Y. The ecology of the African vectors of heartwater, with particular reference to *Amblyomma hebraeum* and *Amblyomma variegatum*. Onderstepoort J Vet Res 1987; 54(3): 381-95. |
|  | *Amblyomma lepidum* | Petney TN, Horak IG, Rechav Y. The ecology of the African vectors of heartwater, with particular reference to *Amblyomma hebraeum* and *Amblyomma variegatum*. Onderstepoort J Vet Res 1987; 54(3): 381-95. |
|  | *Amblyomma gemma* | Petney TN, Horak IG, Rechav Y. The ecology of the African vectors of heartwater, with particular reference to *Amblyomma hebraeum* and *Amblyomma variegatum*. Onderstepoort J Vet Res 1987; 54(3): 381-95. |
|  | *Amblyomma cohaerens* | Petney TN, Horak IG, Rechav Y. The ecology of the African vectors of heartwater, with particular reference to *Amblyomma hebraeum* and *Amblyomma variegatum*. Onderstepoort J Vet Res 1987; 54(3): 381-95. |
|  | *Amblyomma cajennense* | Szabó MP, Pinter A, Labruna MB. Ecology, biology and distribution of spotted-fever tick vectors in Brazil. Front Cell Infect Microbiol 2013; 3: 27. |
|  | *Amblyomma ovale* | Szabó MP, Pinter A, Labruna MB. Ecology, biology and distribution of spotted-fever tick vectors in Brazil. Front Cell Infect Microbiol 2013; 3: 27. |
|  | *Amblyomma maculatum* | Zemtsova GE, Watkins NE, JRhipicephalus, Levin ML. Multiplex qPCR assay for identification and differentiation of *Amblyomma americanum*, *Amblyomma cajennense*, and *Amblyomma maculatum* (Ixodida: Ixodidae) tick species in the eastern United States. J Med Entomol 2014; 51(4): 795-803. |
|  | *Argas persicus* | Dehhaghi M, Kazemi Shariat Panahi H, Holmes EC, et al. Human tick-borne diseases in Australia. Front Cell Infect Microbiol 2019; 9: 3. |
|  | *Argas vespertilionis* | Socolovschi C, Kernif T, Raoult D, Parola P. Borrelia, Rickettsia, and *Ehrlichia* species in bat ticks, France, 2010. Emerg Infect Dis 2012; 18(12): 1966-75. |
|  | *Argas japonicus* | Yan P, Qiu Z, Zhang T, et al. Microbial diversity in the tick *Argas japonicus* (Acari: Argasidae) with a focus on Rickettsia pathogens. Med Vet Entomol 2019; 33(3): 327-35. |
|  | *Dermacentor auratus* | Bandaranayaka KO, Kularatne SAM, Rajapakse RPVJ, et al. Human Otoacariasis in two climatically diverse districts in Sri Lanka: seasonality, risk factors, and case notes. Acta Parasitol. 2021;66(4):1326-1340. |
|  | *Dermacentor albipictus* | Eisen L. Tick species infesting humans in the United States. Ticks Tick Borne Dis. 2022;13(6):102025. |
| Tick | *Dermacentor marginatus* | Garcia-Vozmediano A, Giglio G, Ramassa E, et al. *Dermacentor marginatus* and *Dermacentor reticulatus*, and Their Infection by SFG Rickettsiae and Francisella-Like Endosymbionts, in Mountain and Periurban Habitats of Northwestern Italy. Vet Sci 2020; 7(4). |
|  | *Dermacentor silvarum* | Guo WB, Shi WQ, Wang Q, et al. Distribution of *Dermacentor silvarum* and associated pathogens: Meta-Analysis of global published data and a field survey in China. Int J Environ Res Public Health 2021; 18(9). |
|  | *Dermacentor reticulatus* | Khasnatinov MA, Liapunov AV, Manzarova EL, et al. The diversity and prevalence of hard ticks attacking human hosts in Eastern Siberia (Russian Federation) with first description of invasion of non-endemic tick species. Parasitol Res 2016; 115(2): 501-10. |
|  | *Dermacentor nuttalli* | Khasnatinov MA, Liapunov AV, Manzarova EL, et al. The diversity and prevalence of hard ticks attacking human hosts in Eastern Siberia (Russian Federation) with first description of invasion of non-endemic tick species. Parasitol Res 2016; 115(2): 501-10. |
|  | *Dermacentor variabilis* | Madison-Antenucci S, Kramer LD, Gebhardt LL, et al. Emerging Tick-borne diseases. Clin Microbiol Rev 2020; 33(2). |
|  | *Dermacentor andersoni* | Merino O, De la Cruz NI, Martinez J, et al. Molecular detection of Rickettsia species in ticks collected in the Mexico-USA transboundary region. Exp Appl Acarol 2020; 80(4): 559-67. |
|  | *Dermacentor occidentalis* | Salkeld DJ, Porter WT, Loh SM, et al. Time of year and outdoor recreation affect human exposure to ticks in California, United States. Ticks Tick Borne Dis 2019; 10(5): 1113-7. |
|  | *Dermacentor nitens* | Szabó MPJ, Martins TF, Barbieri ARM, et al. Ticks biting humans in the Brazilian savannah: Attachment sites and exposure risk in relation to species, life stage and season. Ticks Tick Borne Dis 2020; 11(2): 101328. |
|  | *Haemaphysalis leachi* | Dick G, Lewis E. A rickettsial disease in East Africa transmitted by ticks (*Rhipicephalus simus* and *Haemaphysalis leachi*). Transactions of the Royal Society of Tropical Medicine and Hygiene 1947; 41(3): 295-326. |
|  | *Haemaphysalis bancrofti* | Gofton AW, Doggett S, Ratchford A, et al. Bacterial Profiling Reveals Novel "*Ca. Neoehrlichia*", *Ehrlichia*, and *Anaplasma* Species in Australian Human-Biting Ticks. PLoS One. 2015;10(12): e0145449. |
|  | *Haemaphysalis qinghaiensis* | Guglielmone, AA, Robbins, R.G. Tick species found feeding on humans.2018. |
|  | *Haemaphysalis heinrichi* | Guglielmone, AA, Robbins, R.G. Tick species found feeding on humans.2018. |
|  | *Haemaphysalis humerosa* | Guglielmone, AA, Robbins, R.G. Tick species found feeding on humans.2018. |
|  | *Haemaphysalis shimoga* | Guglielmone, AA, Robbins, R.G. Tick species found feeding on humans.2018. |
|  | *Haemaphysalis campanulata* | Guglielmone, AA, Robbins, R.G. Tick species found feeding on humans.2018. |
|  | *Haemaphysalis leporispalustris* | Hahn MB, Disler G, Durden LA, et al. Establishing a baseline for tick surveillance in Alaska: Tick collection records from 1909-2019. Ticks Tick Borne Dis 2020; 11(5): 101495. |
|  | *Haemaphysalis erinacei* | Keskin A, Keskin A, Bursali A, Tekin S. Ticks (Acari: Ixodida) parasitizing humans in Corum and Yozgat provinces, Turkey. Exp Appl Acarol 2015; 67(4): 607-16. |
|  | *Haemaphysalis sulcata* | Keskin A, Keskin A, Bursali A, Tekin S. Ticks (Acari: Ixodida) parasitizing humans in Corum and Yozgat provinces, Turkey. Exp Appl Acarol 2015; 67(4): 607-16. |
|  | *Haemaphysalis parva* | Keskin A, Keskin A, Bursali A, Tekin S. Ticks (Acari: Ixodida) parasitizing humans in Corum and Yozgat provinces, Turkey. Exp Appl Acarol 2015; 67(4): 607-16. |
|  | *Haemaphysalis concinna* | Khasnatinov MA, Liapunov AV, Manzarova EL, Kulakova NV, Petrova IV, Danchinova GA. The diversity and prevalence of hard ticks attacking human hosts in Eastern Siberia (Russian Federation) with first description of invasion of non-endemic tick species. Parasitol Res 2016; 115(2): 501-10. |
|  | *Haemaphysalis longicornis* | Madison-Antenucci S, Kramer LD, Gebhardt LL, Kauffman E. Emerging tick-borne diseases. Clin Microbiol Rev 2020; 33(2). |
|  | *Haemaphysalis flava* | Ozawa A, Yamaguchi N, Hayakawa K, et al. [A case of tick bite (*Haemaphysalis flava*)--consideration of tularemia infection through tick bite]. Nihon Hifuka Gakkai Zasshi 1982; 92(13): 1415-21. |
|  | *Haemophysalis punctata* | Raad M, Azar D, Perotti MA. First Report of the Ticks *Haemaphysalis punctata* Canestrini et Fanzago, 1878, *Haemaphysalis parva* (Neumann, 1897) and *Dermacentor marginatus* (Sulzer, 1776) (Acari, Amblyommidae) from Humans in Lebanon. Acta Parasitol 2020; 65(2): 541-5. |
| Tick | *Haemaphysalis megaspinosa* | Seishima M, Izumi T, Oyama Z, Kadosaka T. Tick bite by *Haemaphysalis* *megaspinosa* - first case. Eur J Dermatol 2000; 10(5): 389-91. |
|  | *Haemaphysalis juxtakochi* | Valente JDM, Silva PW, Arzua M, et al. Records of ticks (Acari: Ixodidae) on humans and distribution of spotted-fever cases and its tick vectors in Paraná State, southern Brazil. Ticks Tick Borne Dis 2020; 11(6): 101510. |
|  | *Hyalomma detritum* | Aktas M. A survey of ixodid tick species and molecular identification of tick-borne pathogens. Vet Parasitol 2014; 200(3-4): 276-83. |
|  | *Hyalomma impeltatum* | Bursali A, Keskin A, Tekin S. Ticks (Acari: Ixodida) infesting humans in the provinces of Kelkit Valley, a Crimean-Congo Hemorrhagic Fever endemic region in Turkey. Exp Appl Acarol 2013; 59(4): 507-15. |
|  | *Hyalomma schulzei* | Guglielmone, AA, Robbins, R.G. Tick species found feeding on humans. 2018. |
|  | *Hyalomma truncatum* | Horak IG, Fourie LJ, Heyne H, et al. Ixodid ticks feeding on humans in South Africa: with notes on preferred hosts, geographic distribution, seasonal occurrence and transmission of pathogens. Exp Appl Acarol 2002; 27(1-2): 113-36. |
|  | *Hyalomma anatolicum* | Hosseini A, Dalimi A, Abdigoudarzi M. Morphometric Study on Male Specimens of Hyalomma anatolicum (Acari: Ixodidae) in West of Iran. Iran J Arthropod Borne Dis 2011; 5(2): 23-31. |
|  | *Hyalomma excavatum* | Keskin A, Keskin A, Bursali A, Tekin S. Ticks (Acari: Ixodida) parasitizing humans in Corum and Yozgat provinces, Turkey. Exp Appl Acarol 2015; 67(4): 607-16. |
|  | *Hyalomma aegyptium* | Keskin A, Keskin A, Bursali A, Tekin S. Ticks (Acari: Ixodida) parasitizing humans in Corum and Yozgat provinces, Turkey. Exp Appl Acarol 2015; 67(4): 607-16. |
|  | *Hyalomma marginatum* | Keskin A, Keskin A, Bursali A, Tekin S. Ticks (Acari: Ixodida) parasitizing humans in Corum and Yozgat provinces, Turkey. Exp Appl Acarol 2015; 67(4): 607-16. |
|  | *Hyalomma asiaticum* | Liu ZQ, Xia J, Wang GL, Kuermanali N. Cloning and expression of the 4D8 gene from Hyalomma asiaticum tick. Genet Mol Res 2016; 15(2). |
|  | *Hyalomma rufipes* | Medialdea-Carrera R, Melillo T, Micaleff C, Borg ML. Detection of Hyalomma rufipes in a recently arrived asylum seeker to the EU. Ticks Tick Borne Dis 2021; 12(1): 101571. |
|  | *Hyalomma dromedarii* | Mosabah AA, Morsy TA. Tick paralysis: first zoonosis record in Egypt. J Egypt Soc Parasitol 2012; 42(1): 71-8. |
|  | *Hyalomma lusitanicum* | Santos-Silva MM, Beati L, Santos AS, et al. The hard-tick fauna of mainland Portugal (Acari: Ixodidae): an update on geographical distribution and known associations with hosts and pathogens. Exp Appl Acarol 2011; 55(1): 85-121. |
|  | *Ixodes pacificus* | David T Gauthier, Christopher D Paddock, et al. Characterization of a novel transitional group Rickettsia species (Rickettsia tillamookensis sp. nov.) from the western black-legged tick, Ixodes pacificus. Int J Syst Evol Microbiol 2021; 71(7): 004880. |
|  | *Ixodes spinipalpis* | Eisen L. Tick species infesting humans in the United States. Ticks Tick Borne Dis. 2022;13(6):102025. |
|  | *Ixodes cookei* | Eisen L. Tick species infesting humans in the United States. Ticks Tick Borne Dis. 2022;13(6):102025. |
|  | *Ixodes angustus* | Eisen L. Tick species infesting humans in the United States. Ticks Tick Borne Dis. 2022;13(6):102025. |
|  | *Ixodes woodi* | Eisen L. Tick species infesting humans in the United States. Ticks Tick Borne Dis. 2022;13(6):102025. |
|  | *Ixodes hexagonus* | Faulde MK, Rutenfranz M, Hepke J, Rogge M, Görner A, Keth A. Human tick infestation pattern, tick-bite rate, and associated Borrelia burgdorferi s.l. infection risk during occupational tick exposure at the Seedorf military training area, northwestern Germany. Ticks Tick Borne Dis 2014; 5(5): 594-9. |
|  | *Ixodes frontalis* | Gilot B, Beaucournu JC, Chastel C. [Collecting with the flagging method and fixing on man of *Ixodes* (Trichotoixodes) frontalis (Panzer, 1795)]. Parasite 1997; 4(2): 197-9. |
|  | *Ixodes holocyclus* | Graves SR, Stenos J. Tick-borne infectious diseases in Australia. Med J Aust 2017; 206(7): 320-4. |
|  | *Ixodes tasmani* | Guglielmone, AA, Robbins, R.G. Tick species found feeding on humans.2018. |
|  | *Ixodes crenulatus* | Guglielmone, AA, Robbins, R.G. Tick species found feeding on humans.2018. |
| Tick | *Ixodes apronophorus* | Guglielmone, AA, Robbins, R.G. Tick species found feeding on humans.2018. |
|  | *Ixodes tanuki* | Guglielmone, AA, Robbins, R.G. Tick species found feeding on humans.2018. |
|  | *Ixodes trianguliceps* | Hubbard MJ, Baker AS, Cann KJ. Distribution of *Borrelia burgdorferi s.l.* spirochaete DNA in British ticks (Argasidae and Ixodidae) since the 19th century, assessed by PCR. Med Vet Entomol 1998; 12(1): 89-97. |
|  | *Ixodes nipponensis* | Lee SH, Shin NR, Kim CM, et al. First identification of *Anaplasma phagocytophilum* in both a biting tick *Ixodes nipponensis* and a patient in Korea: a case report. BMC Infect Dis 2020; 20(1): 826. |
|  | *Ixodes scapularis* | Madison-Antenucci S, Kramer LD, Gebhardt LL, Kauffman E. Emerging tick-borne diseases. Clin Microbiol Rev 2020; 33(2). |
|  | *Ixodes ricinus* | Madison-Antenucci S, Kramer LD, Gebhardt LL, Kauffman E. Emerging tick-borne diseases. Clin Microbiol Rev 2020; 33(2). |
|  | *Ixodes persulcatus* | Madison-Antenucci S, Kramer LD, Gebhardt LL, Kauffman E. Emerging tick-borne diseases. Clin Microbiol Rev 2020; 33(2). |
|  | *Ixodes acuminatus* | Nasirian, H., Zahirnia, A. Detailed Infestation Spectrums About Biological Stages of Hard Ticks (Acari: Ixodida: Ixodidae) in Humans: A Systematic Review and Meta-Analysis. Acta Parasit. 66, 770–796 (2021). |
|  | *Ixodes simplex* | Péter Á, Barti L, Corduneanu A, Hornok S, Mihalca AD, Sándor AD. First record of Ixodes simplex found on a human host, with a review of cases of human infestation by bat tick species occurring in Europe. Ticks Tick Borne Dis. 2021;12(4):101722. |
|  | *Ixodes ventalloi* | Sanogo YO, Parola P, Shpynov S, et al. Genetic diversity of bacterial agents detected in ticks removed from asymptomatic patients in northeastern Italy. Ann N Y Acad Sci 2003; 990: 182-90. |
|  | *Ixodes ovatus* | Sasaki K, Honma M, Nakao M, et al. Survey to detect tick-borne encephalitis virus from human-feeding ticks in Hokkaido, Japan. J Dermatol 2021; 48(7): 1094-7. |
|  | *Ixodes dammini* | Smith RP Jr, Lacombe EH, Rand PW, Dearborn R. Diversity of tick species biting humans in an emerging area for Lyme disease. Am J Public Health. 1992;82(1):66-69. |
|  | *Ixodes turdus* | Woo IC, Baba S, Suzuki H, Kawabata M. A case of tick bite with Ixodes turdus Nakatsuji--a report from Japan. J Dermatol. 1990;17(1):56-58. |
|  | *Ornithodoros lahorensis* | Chen Z, Liu J. A review of argasid ticks and associated pathogens of China. Front Vet Sci. 2022; 9:865664. |
|  | *Rhipicephalus maculatus* | Chitimia-Dobler L, Mans BJ. An exotic souvenir on a German traveler returning from South Africa. Parasitol Res. 2022;121(5):1527-1531. |
|  | *Rhipicephalus simus* | Dick G, Lewis E. A rickettsial disease in East Africa transmitted by ticks (*Rhipicephalus simus* and *Haemaphysalis leachi*). Transactions of the Royal Society of Tropical Medicine and Hygiene 1947; 41(3): 295-326. |
|  | *Rhipicephalus pravus* | Guglielmone, AA, Robbins, R.G. Tick species found feeding on humans.2018. |
|  | *Rhipicephalus humeralis* | Guglielmone, AA, Robbins, R.G. Tick species found feeding on humans.2018. |
|  | *Rhipicephalus evertsi* | Horak IG, Fourie LJ, Heyne H, et al. Ixodid ticks feeding on humans in South Africa: with notes on preferred hosts, geographic distribution, seasonal occurrence and transmission of pathogens. Exp Appl Acarol 2002; 27(1-2): 113-36. |
|  | *Rhipicephalus muehlensi* | Horak IG, Fourie LJ, Heyne H, et al. Ixodid ticks feeding on humans in South Africa: with notes on preferred hosts, geographic distribution, seasonal occurrence and transmission of pathogens. Exp Appl Acarol 2002; 27(1-2): 113-36. |
|  | *Rhipicephalus appendiculatus* | Horak IG, Fourie LJ, Heyne H, et al. Ixodid ticks feeding on humans in South Africa: with notes on preferred hosts, geographic distribution, seasonal occurrence and transmission of pathogens. Exp Appl Acarol 2002; 27(1-2): 113-36. |
|  | *Rhipicephalus evertsi* | Horak IG, Fourie LJ, Heyne H, et al. Ixodid ticks feeding on humans in South Africa: with notes on preferred hosts, geographic distribution, seasonal occurrence and transmission of pathogens. Exp Appl Acarol 2002; 27(1-2): 113-36. |
|  | *Rhipicephalus gertrudae* | Horak IG, Fourie LJ, Heyne H, et al. Ixodid ticks feeding on humans in South Africa: with notes on preferred hosts, geographic distribution, seasonal occurrence and transmission of pathogens. Exp Appl Acarol 2002; 27(1-2): 113-36. |
|  | *Rhipicephalus* sp. near warburtoni | Horak IG, Fourie LJ, Heyne H, et al. Ixodid ticks feeding on humans in South Africa: with notes on preferred hosts, geographic distribution, seasonal occurrence and transmission of pathogens. Exp Appl Acarol 2002; 27(1-2): 113-36. |
| Tick | *Rhipicephalus pumilio* | Jia N, Zheng YC, Ma L, et al. Human infections with *Rickettsia raoultii*, China. Emerg Infect Dis. 2014; 20(5): 866-868. |
|  | *Rhipicephalus annulatus* | Keskin A, Keskin A, Bursali A, Tekin S. Ticks (Acari: Ixodida) parasitizing humans in Corum and Yozgat provinces, Turkey. Exp Appl Acarol 2015; 67(4): 607-16. |
|  | *Rhipicephalus turanicus* | Keskin A, Keskin A, Bursali A, Tekin S. Ticks (Acari: Ixodida) parasitizing humans in Corum and Yozgat provinces, Turkey. Exp Appl Acarol 2015; 67(4): 607-16. |
|  | *Rhipicephalus bursa* | Keskin A, Keskin A, Bursali A, Tekin S. Ticks (Acari: Ixodida) parasitizing humans in Corum and Yozgat provinces, Turkey. Exp Appl Acarol 2015; 67(4): 607-16. |
|  | *Rhipicephalus sanguineus* | Madison-Antenucci S, Kramer LD, Gebhardt LL, Kauffman E. Emerging tick-borne diseases. Clin Microbiol Rev 2020; 33(2). |
|  | *Rhipicephalus pusillus* | Santos-Silva MM, Beati L, Santos AS, et al. The hard-tick fauna of mainland Portugal (Acari: Ixodidae): an update on geographical distribution and known associations with hosts and pathogens. Exp Appl Acarol 2011; 55(1): 85-121. |
|  | *Rhipicephalus haemaphysaloides* | Soundararajan C, Nagarajan K, Arul Prakash M. Tick infestation in human beings in the Nilgiris and Kancheepuram district of Tamil Nadu, India. J Parasit Dis 2018; 42(1): 50-4. |
|  | *Rhipicephalus microplus* | Szabó MPJ, Martins TF, Barbieri ARM, et al. Ticks biting humans in the Brazilian savannah: Attachment sites and exposure risk in relation to species, life stage and season. Ticks Tick Borne Dis 2020; 11(2): 101328. |
|  | *Rhipicephalus kohlsi* | Yaman, M. & Zerek, A. A Case of *Rhipicephalus* (*Boophilus*) *kohlsi* (Hoogstraal and Kaiser, 1960) in A Mediterranean Chameleon (Chamaeleo chamaeleon). 2016; 30, 55-56. |
| Flea | *Ctenocephalides canis* | Beck W, Clark HH. [Differential diagnosis of medically relevant flea species and their significance in dermatology]. Hautarzt 1997; 48(10): 714-9. |
|  | *Ctenocephalides felis* | Mumcuoglu Y, Rufli T. [Siphonaptera/fleas (author's transl)]. Schweiz Rundsch Med Prax 1979; 68(37): 1172-82. |
|  | *Pulex irritans* | Brouqui P, Raoult D. Arthropod-borne diseases in homeless. Ann N Y Acad Sci 2006; 1078: 223-35. |
|  | *Xenopsylla cheopis* | Brouqui P, Raoult D. Arthropod-borne diseases in homeless. Ann N Y Acad Sci 2006; 1078: 223-35. |
| Fly | *Lipoptena fortisetosa* | Andreani A, Rosi MC, Guidi R, et al. Colour Preference of the Deer Ked *Lipoptena fortisetosa* (Diptera: Hippoboscidae). Insects 2021; 12(9). |
|  | *Lipoptena cervi* | Rantanen T, Reunala T, Vuojolahti P, Hackman W. Persistent pruritic papules from deer ked bites. Acta Derm Venereol 1982; 62(4): 307-11. |
|  | *Haematopota pluvialis* | Krcmar S, Marić S. Analysis of the feeding sites for some horse flies (Diptera, Tabanidae) on a human in Croatia. Coll Antropol. 2006;30(4):901-904. |
|  | *Tabanus bromius* | Krcmar S, Marić S. Analysis of the feeding sites for some horse flies (Diptera, Tabanidae) on a human in Croatia. Coll Antropol. 2006;30(4):901-904. |
|  | *Glossina pallidipes* | Gashururu RS, Githigia SM, Gasana MN, et al. An update on the distribution of Glossina (tsetse flies) at the wildlife-human-livestock interface of Akagera National Park, Rwanda. Parasit Vectors. 2021;14(1):294. |
|  | *Tabanus bovinus* | Veraldi S, Esposito L. Skin lesions caused by Tabanus bovinus bites. J Travel Med. 2017;24(5):10.1093/jtm/tax049. |
| Louse | *Pediculus humanus* | https://www.sciencedirect.com/topics/medicine-and-dentistry/pediculus-humanus |
| Mite | *Neotrombicula autumnalis* | Leigheb, G. Mite Bites and Tick Dermatoses. 2015; In: Katsambas, A.D., Lotti, T.M., Dessinioti, C., D’Erme, A.M. (eds) European Handbook of Dermatological Treatments. Springer, Berlin, Heidelberg. |
| Mosquito | *Culex pipiens* s.l. | Farajollahi A, Fonseca DM, Kramer LD, Marm Kilpatrick A. "Bird biting" mosquitoes and human disease: a review of the role of Culex pipiens complex mosquitoes in epidemiology. Infect Genet Evol. 2011;11(7):1577-1585. |
|  | *Aedes aegypti* | Powell JR. Mosquito-Borne Human Viral Diseases: Why *Aedes aegypti*?. Am J Trop Med Hyg. 2018;98(6):1563-1565. |
|  | *Aedes albopictus* | Abduljalil JM, Abd Al Galil FM. Molecular pathogenesis of dengue virus infection in Aedes mosquitoes. Journal of insect physiology, 2022; 138: 104367. |
| Mosquito | *Culex quinquefasciatus* | Oduola AO, Awe OO. Behavioural biting preference of *Culex quinquefasciatus* in human host in Lagos metropolis Nigeria. J Vector Borne Dis. 2006;43(1):16-20. |
|  | *Anopheles sinensis* | Luo DY, Yan ZT, Che LR, Zhu JJ, Chen B. Repellency and insecticidal activity of seven Mugwort (Artemisia argyi) essential oils against the malaria vector Anopheles sinensis. Scientific reports, 2022; 12: 5337. |
|  | *Armigeres subalbatus* | Muslim A, Fong MY, Mahmud R, Sivanandam S. Vector and reservoir host of a case of human Brugia pahangi infection in Selangor, peninsular Malaysia. Tropical biomedicine, 2013; 30: 727-30. |
|  | *Culex tritaeniorhynchus* | Van den Eynde C, Sohier C, Matthijs S, De Regge N. Japanese Encephalitis Virus Interaction with mosquitoes: a review of vector competence, vector capacity and mosquito immunity. Pathogens (Basel, Switzerland), 2022; 11. |
|  | *Culex theileri* | Aldemir A, Bedir H, Demirci B, Alten B. Biting activity of mosquito species (Diptera: Culicidae) in the Turkey-Armenia border area, Ararat Valley, Turkey. J Med Entomol. 2010;47(1):22-27. |
| Sandfly | *Lutzomyia longipalpis* | Hamilton JG, Ramsoondar TM. Attraction of Lutzomyia longipalpis to human skin odours. Med Vet Entomol. 1994; 8(4):375-380. |
| Bug | *Cimex hemipterus* | Zahran Z, Ab Majid AH. Human skin reactions towards bites of tropical bed bug, *Cimex hemipterus* F. (Hemiptera: Cimicidae): a preliminary case study. Asian Pacific Journal of Tropical Disease 2016; 6(5): 366-71. |

**Supplementary table 16: Vector/Host type and number infected by species in the Anaplasmataceae family.**

| Anaplasmataceae species | Anaplasmataceae genus | Vector | Wildlife | Domestic animal | Vector/Host type |
| --- | --- | --- | --- | --- | --- |
| *Ae. pullorum* | *Aegyptianella* | - | 1 | - | wildlife |
| *Ae. ranarum* | *Aegyptianella* | - | 1 | - | wildlife |
| *A. marginale* | *Anaplasma* | 54 | 22 | 10 | vector; wildlife; domestic animal |
| *A. centrale* | *Anaplasma* | 16 | 7 | 5 | vector; wildlife; domestic animal |
| *A. ovis* | *Anaplasma* | 37 | 15 | 8 | vector; wildlife; domestic animal |
| *A. bovis* | *Anaplasma* | 46 | 32 | 8 | vector; wildlife; domestic animal |
| *A. phagocytophilum* | *Anaplasma* | 97 | 208 | 12 | vector; wildlife; domestic animal |
| *A. mesaeterum* | *Anaplasma* | - | - | 2 | domestic animal |
| *A. platys* | *Anaplasma* | 35 | 9 | 7 | vector; wildlife; domestic animal |
| *A. odocoilei* | *Anaplasma* | 1 | 1 | - | vector; wildlife |
| *A. capra* | *Anaplasma* | 5 | 8 | 6 | vector; wildlife; domestic animal |
| *Candidatus* A. camelii | *Anaplasma* | 8 | - | 1 | vector; domestic animal |
| *Candidatus* A. pangolinii | *Anaplasma* | - | 1 | - | wildlife |
| *Candidatus* A. boleense | *Anaplasma* | 7 | - | - | vector |
| *Candidatus* A. rodmosense | *Anaplasma* | 3 | - | - | vector |
| *Candidatus* A. ivorensis | *Anaplasma* | 1 | - | - | vector |
| *Candidatus* A. corsicanum | *Anaplasma* | - | - | 1 | domestic animal |
| *Candidatus* A. mediterraneum | *Anaplasma* | - | - | 1 | domestic animal |
| *Anaplasma* sp. HLJ-14 | *Anaplasma* | 1 | - | - | vector |
| *Candidatus* A. sphenisci | *Anaplasma* | - | 1 | - | wildlife |
| *Candidatus* A. dongdaense | *Anaplasma* | - | - | 2 | domestic animal |
| *Candidatus* A. zhouzhiense | *Anaplasma* | - | - | 1 | domestic animal |
| *Anaplasma* sp. Mymensingh | *Anaplasma* | - | - | 1 | domestic animal |
| *Candidatus* A. africae | *Anaplasma* | - | - | 3 | domestic animal |
| *Candidatus* A. brasiliensis | *Anaplasma* | - | 1 | - | wildlife |
| *Anaplasma* sp. 'Mongolian' | *Anaplasma* | - | - | 1 | domestic animal |
| *Candidatus* A. amazonensis | *Anaplasma* | - | - | 1 | domestic animal |
| *Candidatus* A. gabonensis | *Anaplasma* | - | 2 | - | wildlife |
| *Candidatus* A. testudinis | *Anaplasma* | - | 1 | - | wildlife |
| *Anaplasma* sp. 'Omatjenne' | *Anaplasma* | - | - | 3 | domestic animal |
| *Candidatus* A. sparouinense | *Anaplasma* | - | - | - | human |
| *Candidatus* A. cinensis | *Anaplasma* | - | - | 1 | domestic animal |
| *Candidatus* A. turritanum | *Anaplasma* | - | - | 2 | domestic animal |
| *Candidatus* Al. californiense | *Candidatus* Allocryptoplasma | 1 | - | - | vector |
| *Candidatus* M. longicola | *Candidatus* Mesenet | - | 1 | - | wildlife |
| *Candidatus* N. mikurensis | *Candidatus* Neoehrlichia | 17 | 36 | 1 | vector; wildlife; domestic animal |
| *Candidatus* N. lotoris | *Candidatus* Neoehrlichia | 1 | 2 | 1 | vector; wildlife; domestic animal |
| *Candidatus* Neoehrlichia sp. (FU98) | *Candidatus* Neoehrlichia | - | 2 | - | wildlife |
| *Candidatus* N. arcana | *Candidatus* Neoehrlichia | 5 | - | - | vector |
| *Candidatus* N. australis | *Candidatus* Neoehrlichia | 2 | - | - | vector |
| Candidatus N. chilensis | *Candidatus* Neoehrlichia | 1 | 1 | - | vector; wildlife |
| *Candidatus* X. californiensis | *Candidatus* Xenohaliotis | - | 1 | - | wildlife |
| *E. ruminantium* | *Ehrlichia* | 20 | 3 | 5 | vector; wildlife; domestic animal |
| *E. canis* | *Ehrlichia* | 28 | 35 | 7 | vector; wildlife; domestic animal |
| *E. ovina* | *Ehrlichia* | 2 | - | 1 | vector; domestic animal |
| *E. chaffeensis* | *Ehrlichia* | 26 | 46 | 8 | vector; wildlife; domestic animal |
| *E. ewingii* | *Ehrlichia* | 10 | 4 | 3 | vector; wildlife; domestic animal |
| *E. muris* | *Ehrlichia* | 12 | 11 | 1 | vector; wildlife; domestic animal |
| *Ehrlichia* sp. Tibet | *Ehrlichia* | 1 | - | - | vector |
| *Candidatus* E. walkerii | *Ehrlichia* | 1 | - | - | vector |
| *E. walkeri* | *Ehrlichia* | 1 | - | - | vector |
| Panola Mountain *Ehrlichia* | *Ehrlichia* | 3 | 1 | 1 | vector; wildlife; domestic animal |
| *Candidatus* E. shimanensis | *Ehrlichia* | 1 | 1 | - | vector; wildlife |
| *Ehrlichia* sp. HF | *Ehrlichia* | 3 | - | - | vector |
| *Ehrlichia* sp. 360 | *Ehrlichia* | 1 | - | - | vector |
| *Ehrlichia* sp. HF565 | *Ehrlichia* | 1 | - | - | vector |
| *Ehrlichia* sp. Yamaguchi | *Ehrlichia* | 1 | - | - | vector |
| *Ehrlichia* sp. Yonaguni138 | *Ehrlichia* | 1 | - | - | vector |
| *Candidatus* E. khabarensis | *Ehrlichia* | 3 | 3 | - | vector; wildlife |
| *Candidatus* E. regneryi | *Ehrlichia* | 3 | - | 1 | vector; domestic animal |
| *E. minasensis* | *Ehrlichia* | 9 | - | 3 | vector; domestic animal |
| *Candidatus* E. rustica | *Ehrlichia* | 5 | - | - | vector |
| *Candidatus* E. urmitei | *Ehrlichia* | 5 | - | - | vector |
| *Ehrlichia* sp. hc-hlj209 | *Ehrlichia* | 1 | - | - | vector |
| *Candidatus* E. occidentalis | *Ehrlichia* | 3 | - | - | vector |
| *Ehrlichia* sp. BL157-9 | *Ehrlichia* | 2 | - | - | vector |
| *Ehrlichia* sp. clone SY36 | *Ehrlichia* | 1 | - | - | vector |
| *Candidatus* E. ornithorhynchi | *Ehrlichia* | 1 | 2 | - | vector; wildlife |
| *Ehrlichia* sp. H7 | *Ehrlichia* | - | - | 1 | domestic animal |
| *Candidatus* E. ovata | *Ehrlichia* | 1 | - | - | vector |
| *Candidatus* E. senegalensis | *Ehrlichia* | - | 2 | - | wildlife |
| *Candidatus* E. corsicanum | *Ehrlichia* | 1 | - | - | vector |
| *Candidatus* E. pampeana | *Ehrlichia* | 1 | 1 | - | vector; wildlife |
| *Candidatus* E. hainanensis | *Ehrlichia* | - | 1 | - | wildlife |
| *Candidatus* E. zunyiensis | *Ehrlichia* | - | 1 | - | wildlife |
| *Candidatus* E. dumleri | *Ehrlichia* | - | 1 | - | wildlife |
| *Candidatus* E. hydrochoerus | *Ehrlichia* | - | 1 | - | wildlife |
| *Candidatus* E. andalusi | *Ehrlichia* | 1 | - | - | vector |
| *N. helminthoeca* | *Neorickettsia* | - | 1 | 1 | wildlife; domestic animal |
| *N. sennetsu* | *Neorickettsia* | - | 3 | - | wildlife |
| *N. risticii* | *Neorickettsia* | - | 10 | 3 | wildlife; domestic animal |
| *N. finleia* | *Neorickettsia* | - | - | 1 | domestic animal |
| *W. pipientis* | *Wolbachia* | 27 | 9 | 1 | vector; wildlife; domestic animal |
| *Candidatus* W. ivorensis | *Wolbachia* | 1 | - | - | vector |
| *Candidatus* W. massiliensis | *Wolbachia* | 1 | 2 | - | vector; wildlife |

# **Supplementary table 17: The** **coinfection of Anaplasmataceae species and their infected vectors.**

| Anaplasmataceae1 | Anaplasmataceae2 | Anaplasmataceae3 | Infected vectors |
| --- | --- | --- | --- |
| *A. phagocytophilum* | *A. capra* | *A. bovis* | *Ha. longicornis* |
| *A. phagocytophilum* | *A. capra* | *A. ovis* | *Ha. longicornis* |
| *A. phagocytophilum* | *A. bovis* |  | *Ha. longicornis* |
| *A. phagocytophilum* | *E. chaffeensis* |  | *Ha. longicornis*; *D. silvarum* |
| *A. phagocytophilum* | *E. muris* |  | *I. persulcatus*; *I. scapularis* |
| *A. phagocytophilum* | *E. canis* |  | *Rh. sanguineus* |
| *A. bovis* | *A. capra* |  | *Ha. longicornis* |
| *A. bovis* | *A. ovis* |  | *Ha. longicornis* |
| *A. ovis* | *A. capra* |  | *Ha. longicornis* |
| *A. ovis* | *A. marginale* |  | *Ha. longicornis*; *Haematopinus suis*;  *Linognathus stenopsis*; *Linognathus vituli* |
| *A. bovis* | *E. chaffeensis* |  | *Ha. longicornis* |
| *A. marginale* | *E. minasensis* |  | *Hy. excavatum*; *Rh. annulatus* |
| *A. marginale* | *E. chaffeensis* |  | *Hy. excavatum* |
| *A. platys* | *E. canis* |  | *Rh. sanguineus*; *Rh. haemaphysaloides* |
| *E. chaffeensis* | *E. canis* |  | *Rh. sanguineus* |
| *E. chaffeensis* | *E. ewingii* |  | *Am. americanum* |

**Supplementary table 18: The source of detected positions for six main Anaplasmataceae species and their main host animal information.**

| Anaplasmataceae species | Main source of detected locations | Proportion in all detected positions (%)  (total in the category) | Infection rate (%)/Number of infections in domestic animals | Infection rate (%)/Number of infections in wild animals | Main host animal species | Infection rate (%)/Number of positive infections in the main host animal species |
| --- | --- | --- | --- | --- | --- | --- |
| *A. phagocytophilum** | vector | 52 3 (1219) | 9·5 (7169) | 20·5 (7140) | *Capreolus capreolus* | 27·6 (1968) |
| *A. marginale** | animal | 78·9 (527) | 25·5 (17512) | 13·4 (61) | Cattle | 82·3 (14411) |
| *E. canis** | animal | 74 3 (387) | 14·1 (6169) | 9·1 (273) | Dog | 96·7 (5966) |
| *A. ovis** | animal | 78 8 (349) | 20·2 (8595) | 29·8 (180) | Sheep | 66·9 (5749) |
| *A. platys** | animal | 79 1 (351) | 10·4 (3855) | 12·1 (176) | Dog | 70·8 (2731) |
| *Candidatus* N. mikurensis* | vector | 74 7 (219) | 0·3 (5) | 14·9 (1150) | *Myodes glareolus* | 40·0 (460) |

# **Supplementary table 19: The number of** **human cases with pathogenic Anaplasmataceae species infection by diagnosis methods.**

| **Anaplasmataceae species** | **All infections** | **Molecular assays** | **Serological positives^#^** | | **Cases with reported clinical manifestations** |
| --- | --- | --- | --- | --- | --- |
|  |  |  | **Paired serum samples** | **A single serum sample** |  |
| Overall | 52315 (100·0) * | 41231 (100·0) | 1337 (100·0) | 9747 (100·0) | 7070 (100·0) ^§^ |
| *A. phagocytophilum* | 34796 (66·5) | 26333 (63·9) | 883 (66·0) | 7580 (77·8) | 6299 (89·1) |
| *E. chaffeensis* | 16961 (32·4) | 14541 (35·3) | 343 (25·7) | 2077 (21·3) | 489 (6·9) |
| *Candidatus* N. mikurensis | 160 (0·3) | 105 (0·3) |  | 55 (0·6) | 56 (0·8) |
| *E. muris* | 139 (0·3) | 119 (0·3) |  | 20 (0·2) | 73 (1·0) |
| *E. canis* | 123 (0·2) | 19 (~0) | 89 (6·7) | 15 (0·2) | 92 (1·3) |
| *E. ewingii* | 71 (0·1) | 71 (0·2) |  |  | 19 (0·3) |
| *A. capra* | 28 (0·1) | 28 (0·1) |  |  | 28 (0·4) |
| *N. sennetsu* | 17 (~0) | 5 (~0) | 12 (0·9) |  | 14 (0·2) |
| *N. risticii* | 8 (~0) |  | 8 (0·6) |  | 8 (/) |
| *A. bovis* | 3 (~0) | 1 (~0) | 2 (0·1) |  | 3 (/) |
| *A. platys* | 3 (~0) | 3 (~0) |  |  | 3 (/) |
| *E. ruminantium* | 3 (~0) | 3 (~0) |  |  | 3 (/) |
| *A. ovis* | 1 (~0) | 1 (~0) |  |  |  |
| *Candidatus* A. sparouinense | 1 (~0) | 1 (~0) |  |  | 1 (/) |
| Panola Mountain *Ehrlichia* | 1 (~0) | 1 (~0) |  |  |  |

*Data are presented as numbers of positive infections and proportions (%). ^§^Cases less than 10 are not included in the overall cases for they are not used to analyze the clinical manifestation. **^#^**Serological positives refer to those patients are detected positive by serological assays. Paired serum samples refer to cases with either a four-fold rise or a seroconversion when comparing acute and convalescent samples. A single serum sample refer to cases confirmed by a single positive serum sample.

**Supplementary table 20: Clinical characteristics of human infections with Anaplasmataceae species.**

| **Symptom** | **Human anaplasmosis** | | **Human ehrlichiosis** | | | | **Human neoehrlichiosis** | **Human neorickettsiosis** |
| --- | --- | --- | --- | --- | --- | --- | --- | --- |
|  | ***A. phagocytophilum***  **(n=6299)** | ***A. capra***  **(n=28)** | ***E. chaffeensis***  **(n=489)** | ***E. ewingii***  **(n=19)** | ***E. canis***  **(n=92)** | ***E. muris***  **(n=93)** | ***Candidatus* N. mikurensis**  **(n=56)** | ***N. sennetsu***  **(n=14)** |
| **Influenza-like symptom** |  |  |  |  |  |  |  |  |
| fever | **5644**‒**5652 (89·6**‒**89·7)** | **23 (82·1)** | **429 (87·7)** | **16 (84·2)** | **75 (81·5)** | **64 (87·7)** | **40**‒**44 (71·4**‒**78·6)** | **6**‒**9 (42·9**‒**64·3)** |
| headache | **4038**‒**4168 (64·1**‒**66·2)** | **14 (50·0)** | **324**‒**362 (66·3**‒**74)** | **11**‒**16 (57·9**‒**84·2)** | **68 (73·9)** | **50 (68·5)** | **21**‒**39 (37·5**‒**69·6)** | **9**‒**10 (64·3**‒**71·4)** |
| myalgia | **5398**‒**5440 (85·7**‒**86·4)** | 4 (14·3) | **213**‒**274 (43·6**‒**56)** | 5‒8 (26·3‒42·1) | **63**‒**72 (68·5**‒**78·3)** | **41 (56·2)** | **13**‒**29 (23·2**‒**51·8)** | **11 (78·6)** |
| chills | **4156**‒**4485 (66·0**‒**71·2)** | 4 (14·3) | **158**‒**246 (32·3**‒**50·3)** |  | **36**‒**48 (39·1**‒**52·2)** |  | 10‒22 (17·9‒39·3) | 1 (7·1) |
| fatigue | **3479**‒**3715 (55·2**‒**59·0)** |  | **171**‒**232 (35·0**‒**47·4)** | 1‒4 (5·3‒21·1) | 18‒41 (19·6‒44·6) | **47 (64·4)** | 16‒29 (28·6‒51·8) | 4‒7 (28·6‒50·0) |
| dizziness | 66‒401 (1·0‒6·4) | **9 (32·1)** | 5‒71 (1·0‒14·5) |  |  |  | 6‒14 (10·7‒25·0) |  |
| weakness | 60‒504 (1·0‒8·0) |  | 4‒68 (0·8‒13·9) |  | 1‒11 (1·1‒12·0) |  | 2 (3·6) |  |
| sweats | 33‒372 (0·5‒5·9) |  | 84‒176 (17·2‒36·0) |  | 6‒17 (6·5‒18·5) |  | 11‒22 (19·6‒39·3) |  |
| cough | 40‒173 (0·6‒2·8) |  | 55‒97 (11·2‒19·8) | 2‒6 (10·5‒31·6) | 2‒12 (2·2‒13·0) |  | 5 (8·9) | 3‒4 (21·4‒28·6) |
| sore throat | 5‒7 (0·1‒0·1) |  | 45‒85 (9·2‒17·4) |  | 7‒19 (7·6‒20·7) |  |  | 2 (14·3) |
| **Gastrointestinal symptoms** |  |  |  |  |  |  |  |  |
| nausea | **2111**‒**2461 (33·5**‒**39·1)** |  | **169**‒**205 (34·6**‒**41·9)** | **7**‒**10 (36·8**‒**52·6)** | **37**‒**41 (40·2**‒**44·6)** | 15 (20·5) | 9‒20 (16·1‒35·7) | 3‒7 (21·4‒50·0) |
| vomit | **2245**‒**2456 (35·6**‒**39)** |  | **129**‒**169 (26·4**‒**34·6)** | 5‒7 (26·3‒36·8) | **29**‒**41 (31·5**‒**44·6)** | 15 (20·5) | 5‒13 (8·9‒23·2) | 1 (7·1) |
| anorexia | **4092**‒**4259 (65·0**‒**67·6)** |  | **133**‒**207 (27·2**‒**42·3)** |  | **57**‒**63 (62·0**‒**68·5)** |  |  | 3 (21·4) |
| diarrhea | **2915**‒**3113 (46·3**‒**49·4)** | 8 (28·6) | **215**‒**284 (44·0**‒**58·1)** | 1‒4 (5·3‒21·1) | 3‒18 (3·3‒19·6) |  | 5‒15 (8·9‒26·8) | 2 (14·3) |
| abdominal pain | 32‒2241 (0·5‒35·6) |  | 72‒125 (14·7‒25·6) | 1‒5 (5·3‒26·3) | 12‒22 (13·0‒23·9) |  |  | 2 (14·3) |
| **Skin symptoms** |  |  |  |  |  |  |  |  |
| rash | 631‒973 (10·0‒15·5) | **10 (35·7)** | 123‒188 (25·2‒38·5) | 1‒5 (5·3‒26·3) | **33**‒**46 (35·9**‒**50·0)** | 8 (11·0) | 5‒14 (8·9‒25·0) | 3‒6 (21·4‒42·9) |
| eschar | 30‒257 (0·5‒4·1) | **10 (35·7)** | 1‒41 (0·2‒8·4) |  |  |  |  |  |
| skin erythema | 63‒439 (1·0‒7·0) |  | 1‒50 (0·2‒10·2) |  |  |  | 1 (1·8) |  |
| **Neurological symptoms** |  |  |  |  |  |  |  |  |
| malaise | **4721**‒**5033 (74·9**‒**79·9)** | **13 (46·4)** | **232**‒**346 (47·4**‒**70·8)** | 2‒5 (10·5‒26·3) | 4‒17 (4·3‒18·5) | 51 (69·9) | 10‒27 (17·9‒48·2) |  |
| neck stiffness | 880‒1324 (14·0‒21·0) | 3 (10·7) | 5‒73 (1·0‒14·9) | 1‒3 (5·3‒15·8) | 3‒12 (3·3‒13·0) |  | 5 (8·9) | 2 (14·3) |
| confusion | 18‒228 (0·3‒3·6) |  | 36‒97 (7·4‒19·8) |  | 4‒24 (4·3‒26·1) |  | 1 (1·8) |  |
| **Other symptoms** |  |  |  |  |  |  |  |  |
| arthralgia | **3268**‒**3500 (51·9**‒**55·6)** |  | 122‒182 (24·9‒37·2) | 1‒6 (5·3‒31·6) | 11‒22 (12·0‒23·9) |  | 9‒19 (16·1‒33·9) | **11 (78·6)** |
| conjunctivitis | 48‒67 (0·8‒1·1) |  | 4‒51 (0·8‒10·4) |  |  |  |  |  |
| dyspnea | 48‒193 (0·8‒3·1) |  | 4‒40 (0·8‒8·2) |  |  |  | 1 (1·8) |  |
| lymphadenopathy | 23‒243 (0·4‒3·9) | 8 (28·6) | 59‒128 (12·1‒26·2) |  | 1 (1·1) |  |  |  |
| weight loss | 6‒269 (0·1‒4·3) |  |  |  | 6‒18 (6·5‒19·6) |  | 3 (5·4) |  |
| backache | 4‒117 (0·1‒1·9) |  | 5‒70 (1·0‒14·3) |  | 2‒13 (2·2‒14·1) |  |  | 3 (21·4) |
| eye pain | 3‒132 (0‒2·1) |  |  |  | 3‒8 (3·3‒8·7) |  | 1 (1·8) | 4‒7 (28·6‒50·0) |
| chest pain | 3‒126 (0‒2·0) |  | 2‒40 (0·4‒8·2) |  | 1‒2 (1·1‒2·2) |  | 2 (3·6) | 3 (21·4) |

Data are presented as numbers of positive cases and proportions (%). Only Anaplasmataceae species with ≥10 cases are shown. Symptoms of less than 10 cases in all Anaplasmataceae species were not displayed. When calculating the frequency of each clinical feature, we divided publications into two groups, case reports and case series. A case report describes clinical features of a single patient in detail, for which it is reasonable to assume unmentioned symptoms as absent. In contrast, a case series study summarizes clinical characteristics of a group of confirmed patients, for which it is unclear if unmentioned symptoms are truly absent from the whole group or just rare, especially when the group size is large. For case series studies, we therefore made a conservative assumption that the frequency of an unreported symptom could vary from 0 to the minimum frequency of all reported symptoms. Consequently, we report a range for each symptom if relevant data involve case series. Results are presented as intervals, where the lower bound is obtained assuming the frequency of each unreported symptom in each case series study was zero, and the upper bound is obtained assuming the frequency of each unreported symptom was the same as the minimum frequency among all reported symptoms in each case series study. Cells with a lower bound of the proportion ≥30% are bolded.

# **Supplementary table 21: The predictive accuracy of the predictive models for the eight ticks based on BRT models.**

| Tick species | Grid for occurrences after 2020 | Predicted number of positive grids | Predictive accuracy |
| --- | --- | --- | --- |
| *I. scapularis* | 478 | 469 | 0·98 |
| *I. pacificus* | 125 | 122 | 0·98 |
| *I. persulcatus* | 272 | 262 | 0·96 |
| *I. ricinus* | 1173 | 1077 | 0·92 |
| *Ha. longicornis* | 18 | 17 | 0·94 |
| *D. marginatus* | 50 | 47 | 0·94 |
| *Rh. microplus* | 226 | 201 | 0·89 |
| *Rh. sanguineus* | 167 | 151 | 0·90 |

# **Supplementary table 22: The** **RCs of** **significant contributors to the occurrence of eight main species of ticks based on BRT models.**

| Variable | *I. scapularis* | | *I. pacificus* | *I. persulcatus* | | *I. ricinus* | *Ha. longicornis* | *D. marginatus* | | | *Rh. microplus* | *Rh. sanguineus* | | |  |
| --- | --- | --- | --- | --- | --- | --- | --- | --- | --- | --- | --- | --- | --- | --- | --- |
| Annual mean temperature |  | |  | 6·5 (5·8‒7·2) | | 4·8 (4·4‒5·2) | 13·6 (11·3‒15·9) | 14·7 (12·6‒16·8) | | | |  | | |  |
| Mean diurnal range |  | |  |  | |  |  |  | | |  | 3.3 (2.8‒3.8) | | |  |
| Isothermality | 3·7 (3·1‒4·3) | | | 6·2 (5·4‒7·0) | | | 4·3 (3·2‒5·4) | 4·8 (4·0‒5·6) | | | |  | | |  |
| Temperature seasonality |  | |  | 7·0 (6·1‒7·9) | | 13·9 (13·1‒14·7) | |  | | | **17·1 (15·6‒18·6)** | | | |  |
| Min temperature of coldest month |  | |  |  | |  |  |  | | |  | **60·0 (58.6‒61.4)** | | |  |
| Annual range of temperature |  | |  |  | | 9·5 (7·6‒11·4) | |  | | |  |  | | |  |
| Mean temperature of wettest quarter |  | | 3·1 (2·8‒3·4) | | |  |  | 3·4 (2·7‒4·1) | | | 4·1 (3·8‒4·4) | | | |  |
| Mean temperature of driest quarter |  | 5·3 (4·8‒5·8) | | | 4·8 (3·0‒6·6) | | | |  |  | | |  |  |  |
| Mean temperature of warmest quarter | **41·9 (39·2‒44·6)** | | |  | |  | 5·8 (4·5‒7·1) | 3·5 (2·4‒4·6) | | | |  | | |  |
| Mean temperature of coldest quarter |  | |  |  | |  | 6·2 (4·3‒8·1) | 4·3 (3·2‒5·4) | | | |  | | |  |
| Annual precipitation |  | |  |  | | **29·7 (28·1‒31·3)** | |  | | |  |  | | |  |
| Precipitation of wettest month | 3·5 (1·8‒5·2) | | |  | |  |  |  | | | 7·9 (6·4‒9·4) | | | |  |
| Precipitation of driest month | 16·4 (15·0‒17·8) | | |  | |  |  |  | | |  |  | | |  |
| Precipitation of wettest quarter |  | | 3·1 (2·0‒4·2) | | |  |  |  | | |  |  | |  |  |
| Precipitation of driest quarter |  |  | |  |  | |  | |  |  |  | |  |  | |
| Precipitation of warmest quarter | 4·1 (2·7‒5·5) | | **68·1 (67·1‒69·1)** | | |  | **31·9 (30·1‒33·7)** | | | | 6·7 (6·2‒7·2) | | | |  |
| Precipitation of coldest quarter |  | | 12·1 (10·8‒13·4) | | |  |  | 9·1 (7·1‒11·1) | | | |  | | |  |
| Mixed cropland and nature vegetation |  | |  |  | |  |  |  | | | 3.7 (3.2‒4.2) | | | |  |
| Cropland |  | |  |  | |  |  | 15·7 (13·3‒18·1) | | | |  | | |  |
| Urban built‒up land | 12·5 (10·9‒14·1) | | | 7·7 (7·2‒8·2) | | | 11·9 (9·4‒14·4) | 7·8 (5·8‒9·8) | | | 3·2 (2·9‒3·5) | 7·3 (6·7‒7·9) | | |  |
| Leaf area index |  | |  | **17·0 (15·9‒18·1)** | | |  |  | | |  |  | | |  |
| Buffalo |  | |  | 13·9 (13·1‒14·7) | | |  |  | | |  |  | | |  |
| Cattle |  | |  |  | |  |  |  | | | 14.2 (12.4‒16·0) | | | |  |
| Goat |  | |  | 3·9 (3·5‒4·3) | | 3·8 (3·5‒4·1) | |  | | |  |  | | |  |
| Horse |  | |  |  | | 6·6 (6·0‒7·2) | |  | | | 8·6 (8·0‒9·2) | | | |  |
| Sheep |  | |  |  | |  |  | **17·2 (14·8‒19·6)** | | | | 9·4 (8·7‒10·1) | | |  |
| Mammalian richness |  | |  | 3·6 (3·2‒4·0) | | |  |  | | | 5·1 (4·7‒5·5) | | | |  |
| Rodent richness |  | |  | 4·2 (3·7‒4·7) | | | 3·6 (3·2‒4·0) | | | |  |  | | |  |

The mean (95% CI) RCs of each model were displayed and the max values of tick species were bolded. We only demonstrated effects with mean RC >3%. RC, relative contribution. BRT, boosted regression trees.

# **Supplementary table 23: Sensitive analysis of different threshold for polygon occurrences used in RF models of the six Anaplasmataceae species**·

| Anaplasmataceae species | Threshold | AUC | Sensitivity | Specificity | Accuracyci | F1 score | Kappaci |  |
| --- | --- | --- | --- | --- | --- | --- | --- | --- |
| *A. phagocytophilum* | 100 km^2^ | 0 959 (0 937‒0 974) | 0 931 (0 926‒0 936) | 0 877 (0 873‒0 881) | 0 892 (0 888‒0 895) | 0 809 (0 199‒0 986) | 0 735 (0 727‒0 743) |  |
| *A. phagocytophilum* | 400 km^2^ | 0 964 (0 949‒0 978) | 0 937 (0 931‒0 942) | 0 896 (0 892‒0 899) | 0 900 (0 897‒0 903) | 0 822 (0 207‒0 988) | 0 754 (0 747‒0 761) |  |
| *A. phagocytophilum* | 900 km^2^ | 0 961 (0 944‒0 977) | 0 911 (0 905‒0 916) | 0 890 (0 886‒0 894) | 0 899 (0 896‒0 902) | 0 818 (0 204‒0 987) | 0 749 (0 742‒0 756) |  |
| *Candidatus* N. mikurensis | 100 km^2^ | 0 956 (0 912‒0 986) | 0 882 (0 867‒0 895) | 0 860 (0 851‒0 869) | 0 848 (0 841‒0 856) | 0 748 (0 164‒0 978) | 0 644 (0 627‒0 662) |  |
| *Candidatus* N. mikurensis | 400 km^2^ | 0 957 (0 913‒0 983) | 0 910 (0 897‒0 922) | 0 828 (0 819‒0 837) | 0 866 (0 858‒0 873) | 0 765 (0 172‒0 981) | 0 673 (0 655‒0 691) |  |
| *Candidatus* N. mikurensis | 900 km^2^ | 0 953 (0 902‒0 993) | 0 905 (0 892‒0 917) | 0 844 (0 835‒0 853) | 0 859 (0 852‒0 867) | 0 761 (0 171‒0 980) | 0 665 (0 648‒0 682) |  |
| *E. canis* | 100 km^2^ | 0 963 (0 911‒0 999) | 0 892 (0 874‒0 908) | 0 897 (0 887‒0 906) | 0 896 (0 887‒0 904) | 0 808 (0 197‒0 986) | 0 737 (0 716‒0 759) |  |
| *E. canis* | 400 km^2^ | 0 968 (0 925‒0 999) | 0 894 (0 877‒0 909) | 0 900 (0 890‒0 908) | 0 934 (0 928‒0 940) | 0 872 (0 239‒0 993) | 0 828 (0 812‒0 843) |  |
| *E. canis* | 900 km^2^ | 0 963 (0 912‒0 999) | 0 893 (0 877‒0 907) | 0 948 (0 942‒0 954) | 0 898 (0 890‒0 906) | 0 814 (0 201‒0 987) | 0 745 (0 725‒0 764) |  |
| *A. platys* | 100 km^2^ | 0 921 (0 815‒0 985) | 0 799 (0 777‒0 820) | 0 883 (0 873‒0 892) | 0 862 (0 853‒0 871) | 0 743 (0 158‒0 978) | 0 649 (0 627‒0 672) |  |
| *A. platys* | 400 km^2^ | 0 926 (0 852‒0 981) | 0 834 (0 816‒0 851) | 0 892 (0 884‒0 900) | 0 876 (0 869‒0 884) | 0 772 (0 174‒0 982) | 0 687 (0 669‒0 706) |  |
| *A. platys* | 900 km^2^ | 0 926 (0 880‒0 979) | 0 830 (0 812‒0 846) | 0 853 (0 843‒0 863) | 0 849 (0 840‒0 857) | 0 733 (0 154‒0 977) | 0 630 (0 609‒0 650) |  |
| *A. ovis* | 100 km^2^ | 0 917 (0 860‒0 967) | 0 858 (0 844‒0 871) | 0 838 (0 830‒0 846) | 0 843 (0 836‒0 850) | 0 727 (0 152‒0 976) | 0 621 (0 605‒0 637) |  |
| *A. ovis* | 400 km^2^ | 0 925 (0 871‒0 967) | 0 886 (0 873‒0 897) | 0 838 (0 830‒0 845) | 0 868 (0 862‒0 874) | 0 760 (0 170‒0 980) | 0 672 (0 657‒0 686) |  |
| *A. ovis* | 900 km^2^ | 0 925 (0 880‒0 964) | 0 885 (0 873‒0 896) | 0 862 (0 855‒0 869) | 0 849 (0 843‒0 856) | 0 742 (0 160‒0 977) | 0 639 (0 624‒0 654) |  |
| *A. marginale* | 100 km^2^ | 0 911 (0 852‒0 955) | 0 815 (0 802‒0 828) | 0 831 (0 823‒0 838) | 0 828 (0 821‒0 834) | 0 698 (0 136‒0 971) | 0 581 (0 566‒0 596) |  |
| *A. marginale* | 400 km^2^ | 0 926 (0 868‒0 963) | 0 819 (0 805‒0 832) | 0 881 (0 875‒0 886) | 0 864 (0 859‒0 869) | 0 747 (0 161‒0 979) | 0 655 (0 642‒0 668) |  |
| *A. marginale* | 900 km^2^ | 0 917 (0 876‒0 962) | 0 814 (0 801‒0 826) | 0 867 (0 860‒0 873) | 0 854 (0 848‒0 860) | 0 734 (0 154‒0 977) | 0 635 (0 621‒0 649) |  |

# **Supplementary table 24: The RCs of significant contributors to the spatial distribution of the six major Anaplasmataceae species based on RF models**·

| Variables | *A. phagocytophilum* | *Candidatus* N. mikurensis | *E. canis* | *A. platys* | *A. ovis* | *A. marginale* |
| --- | --- | --- | --- | --- | --- | --- |
| Annual mean temperature | 3 0 (2 0‒4 0) |  |  |  | 3 1 (2 4‒3 8) |  |
| Isothermality |  |  | 3 1 (2 3‒3 9) |  |  |  |
| Temperature seasonality |  | 3 2 (2 4‒4 0) |  |  |  |  |
| Min temperature of coldest month |  |  |  | 3 5 (2 8‒4 2) |  | 3 1 (2 5‒3 7) |
| Annual range of temperature |  | 3 0 (2 4‒3 6) | 3 2 (2 4‒4 0) |  |  |  |
| Mean temperature of wettest quarter | 3 4 (2 6‒4 2) |  |  |  |  |  |
| Mean temperature of driest quarter |  |  |  |  | 3 1 (2 5‒3 7) |  |
| Mean temperature of warmest quarter |  | 3 3 (2 7‒3 9) |  |  |  |  |
| Mean temperature of coldest quarter |  |  |  | 3 1 (2 3‒3 9) |  | 3 1 (2 5‒3 7) |
| Precipitation of wettest month | 3 0 (2 3‒3 7) |  |  |  |  |  |
| Precipitation of driest month |  | 3 1 (2 5‒3 7) |  |  |  |  |
| Precipitation of driest quarter |  | 3 1 (2 4‒3 8) |  |  |  |  |
| Urban built-up land | 4 4 (2 7‒6 1) | 5 4 (3 8‒7 0) | 7 5 (6 1‒8 9) | 5 3 (4 3‒6 3) | 3 4 (2 4‒4 4) | 3 7 (2 3‒5 1) |
| Leaf area index |  |  |  | 3 8 (2 8‒4 8) |  |  |
| Cattle |  |  |  |  |  | 3 2 (2 1‒4 3) |
| Goat |  |  |  |  | 4 5 (2 7‒6 3) | 3 2 (2 1‒4 3) |
| Sheep |  |  |  | 3 1 (1 9‒4 3) |  |  |
| Mammalian richness |  |  |  |  | 3 3 (2 3‒4 3) |  |
| Habitat suitability index of ticks | 5 1 (1 6‒8 6) | 5 8 (3 2‒8 4) | 5 2 (4 3‒6 1) | 7 5 (4 9‒10 1) | 3 8 (2 5‒5 1) | 3 0 (2 3‒3 7) |
| Human Footprint | 4 0 (2 9‒5 1) | 4 5 (3 5‒5 5) | 5 5 (4 7‒6 3) | 5 8 (4 5‒7 1) | 3 2 (2 3‒4 1) |  |
| Population density | 4 3 (2 8‒5 8) | 4 5 (3 5‒5 5) | 8 7 (6 4‒11 0) | 6 3 (4 8‒7 8) | 4 6 (3 3‒5 9) | 4 0 (2 5‒5 5) |

The mean (95% CI) RCs of each model were displayed. We only demonstrated effects with mean RC >3%. RC, relative contribution; RF, random forest.

**Supplementary figure 1: The relationship matrix of Anaplasmataceae species and involved ticks.**

The Anaplasmataceae species determined to infect humans are marked by red fonts. Names of ticks are marked in blue if they bite humans. The red square indicates pathogenic Anaplasmataceae species to humans carried by human-biting vectors, and it turns into purple if the vector was not found to bite humans. Blue squares indicate Anaplasmataceae species were not pathogenic to humans carried by human-biting vectors, and it turns into grey if carried by vectors of non-biting human.


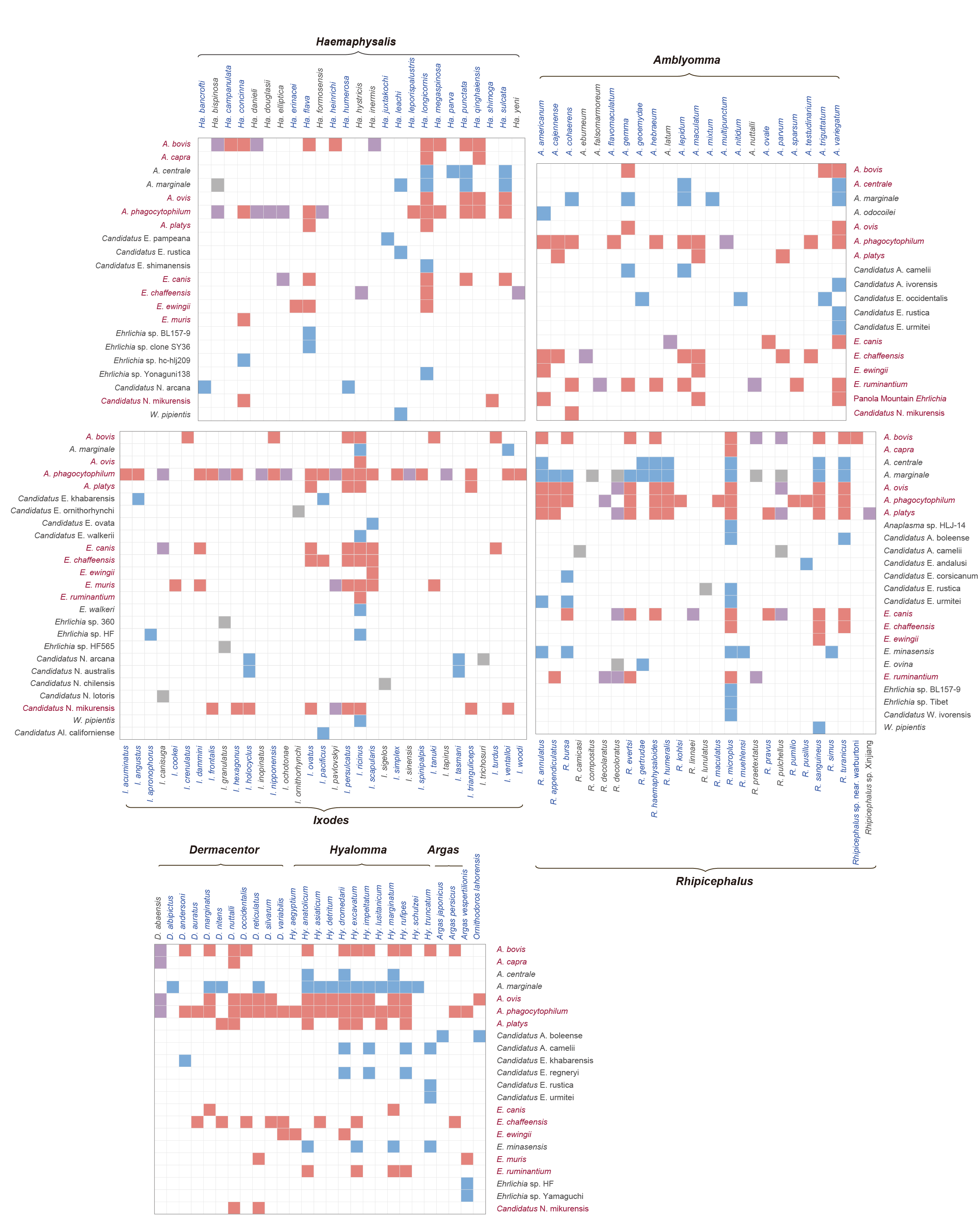


**Supplementary figure 2: The relationship matrix of Anaplasmataceae species and other vectors.**

The Anaplasmataceae species determined to infect humans are marked by red fonts. Names of other vectors are marked in blue if they bite humans. The red square indicates pathogenic Anaplasmataceae species to humans carried by human-biting vectors, and it turns into purple if the vector was not found to bite humans. Blue squares indicate Anaplasmataceae species were not pathogenic to humans carried by human-biting vectors, and it turns into grey if carried by vectors of non-biting human.


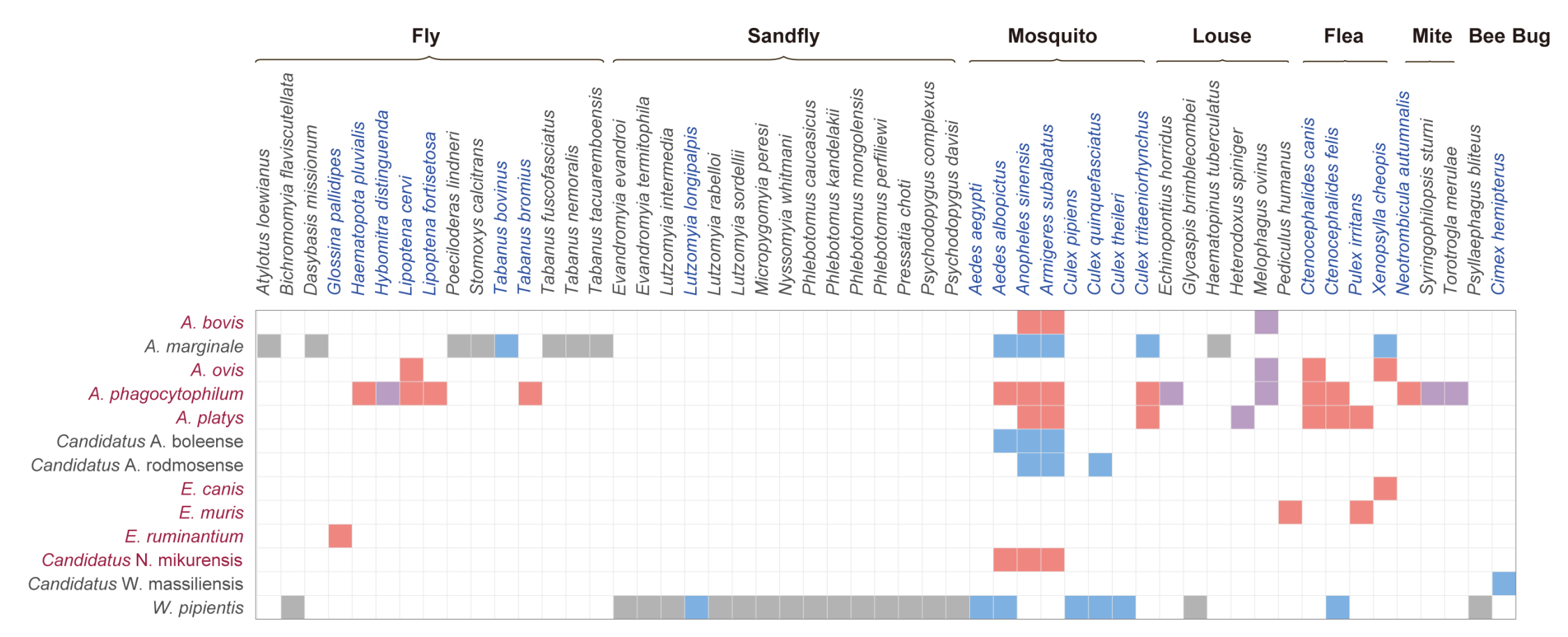


# **Supplementary figure 3: The relationship matrix of Anaplasmataceae species and animals.**

**
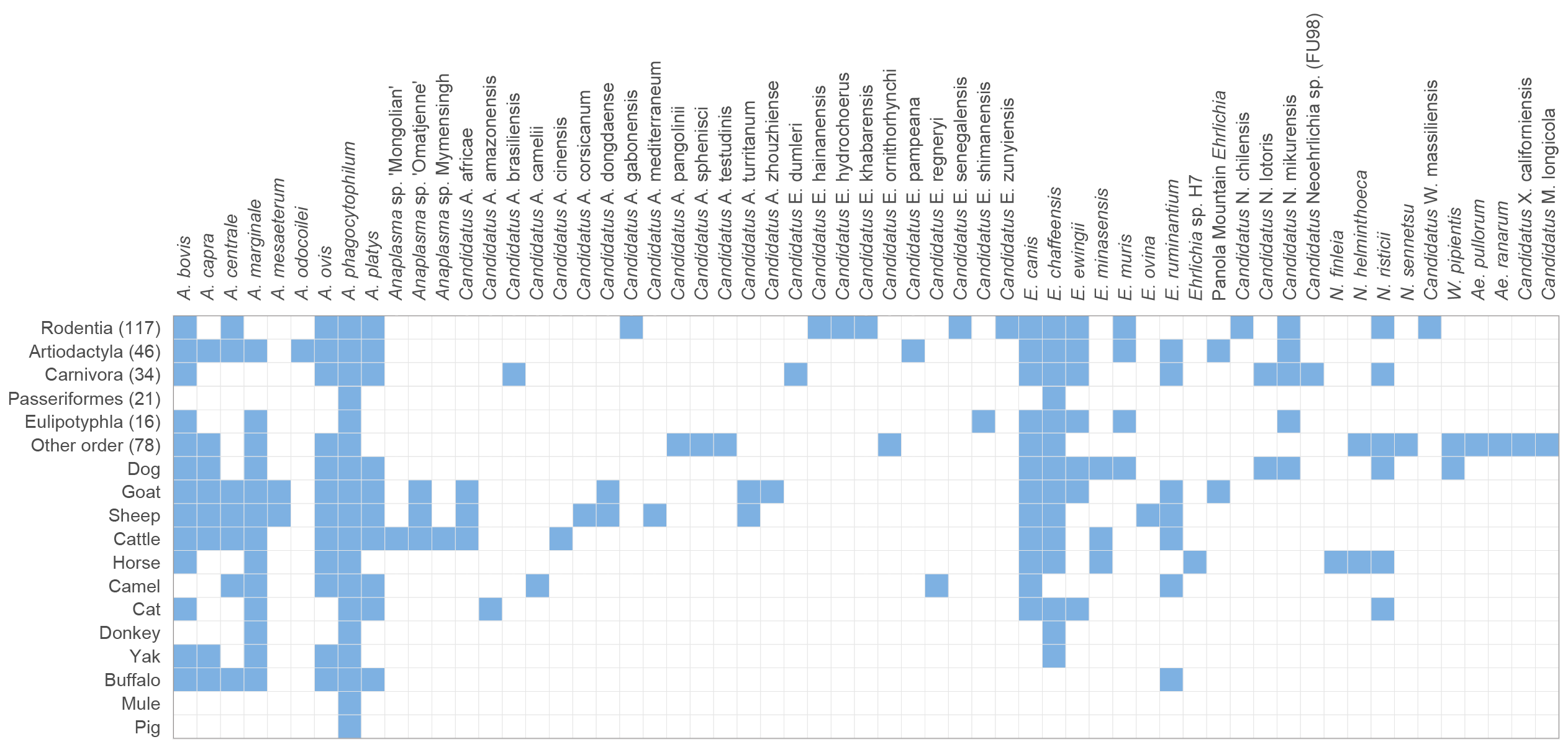
**

# **Supplementary figure 4: The global distribution of the other 73 Anaplasmataceae species.**

(a) *Anaplasma* spp.; (b) *Ehrlichia* spp.; (c) Other genera.


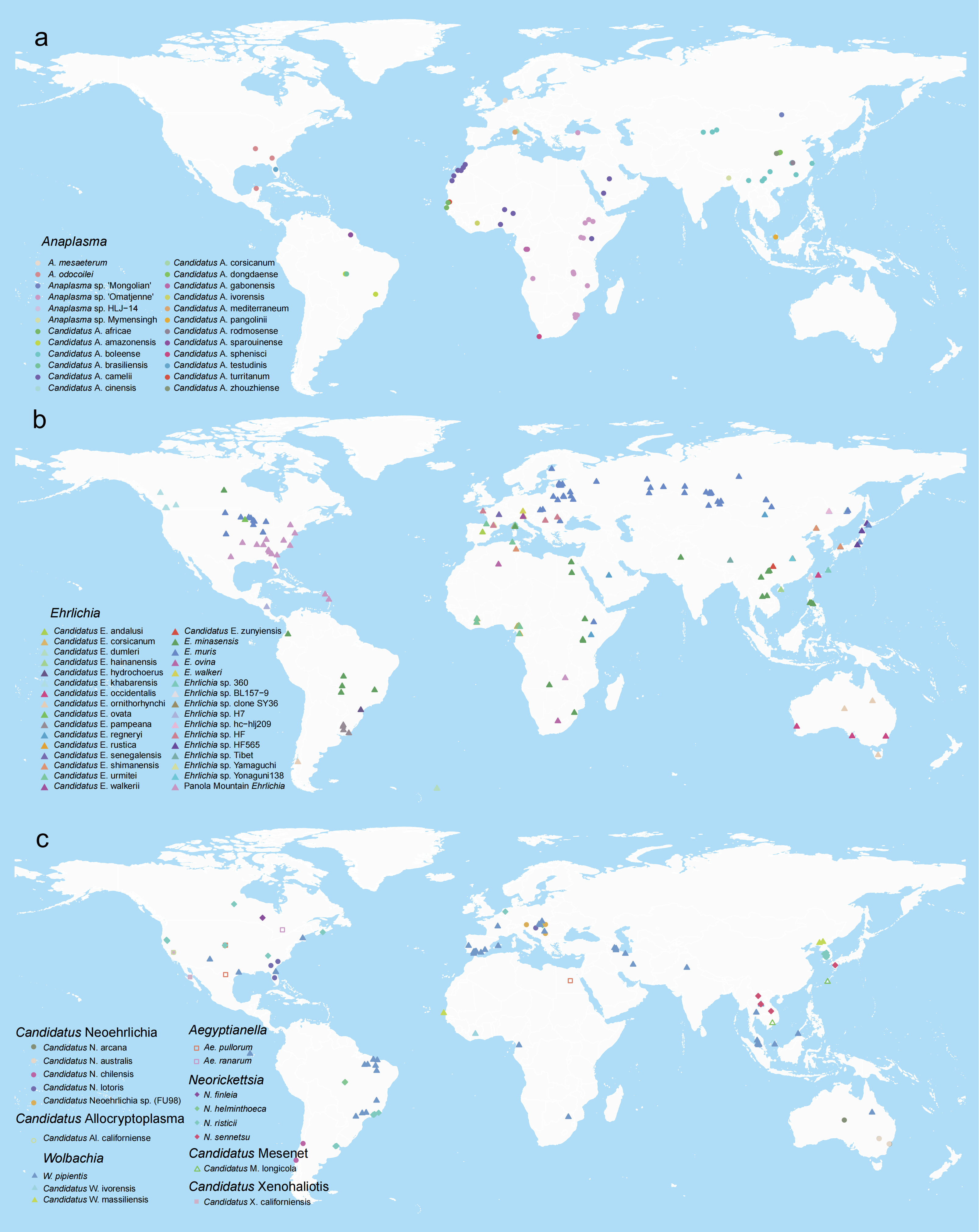


**Supplementary figure 5: Predictive performance of BRT models for eight main ticks.**

ROC curves and AUC values of the BRT over 100 models are shown. Roman numerals 1-8 correspond to the eight major tick species: (Ⅰ) *Ixodes scapularis*; (Ⅱ) *Ixodes pacificus*; (Ⅲ) *Ixodes persulcatus*; (Ⅳ) *Ixodes ricinus*; (Ⅴ) *Haemaphysalis longicornis*; (Ⅵ) *Dermacentor marginatus*; (Ⅶ) *Rhipicephalus microplus*; (Ⅷ) *Rhipicephalus sanguineus*.


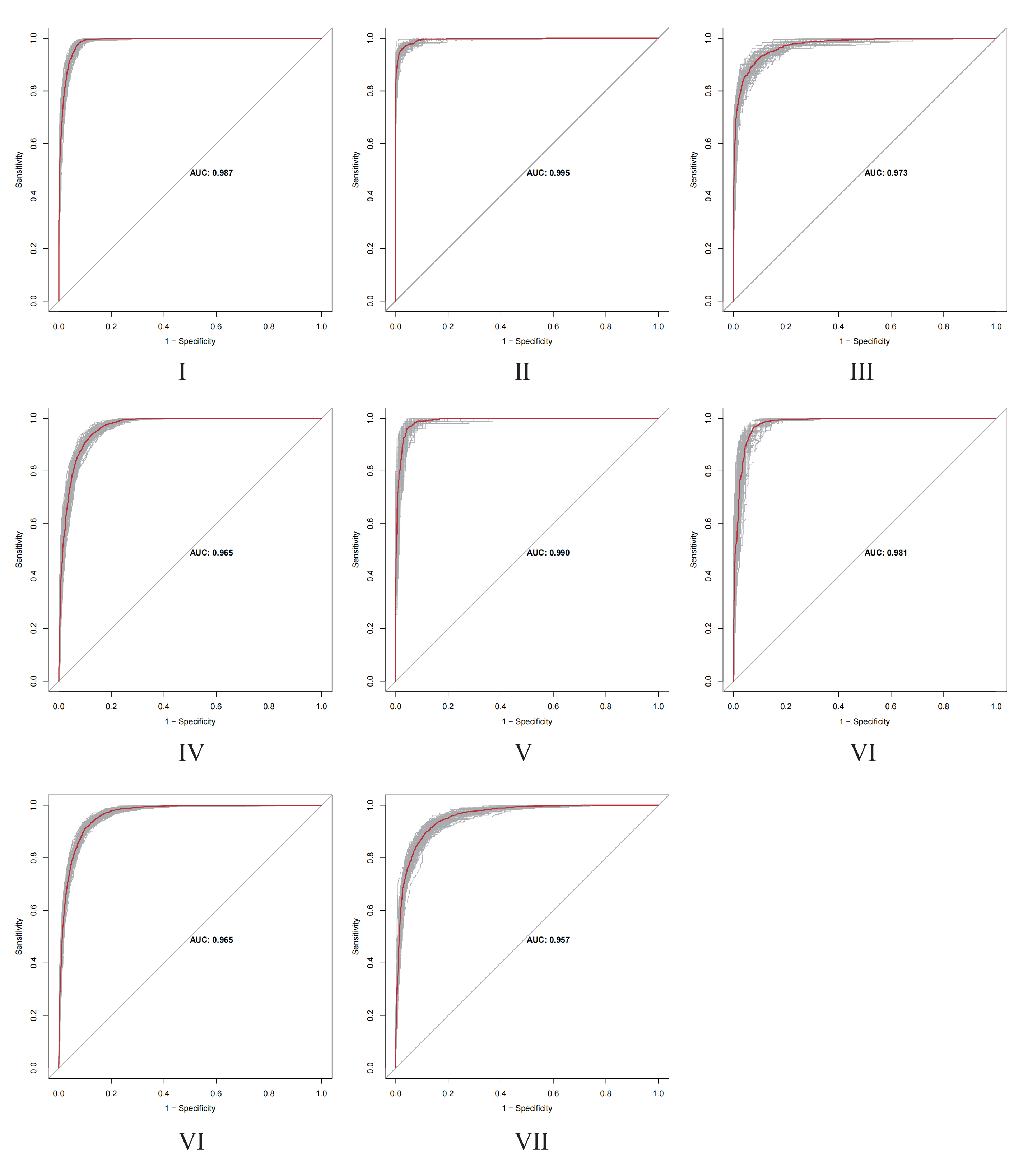


**Supplementary figure 6: The predicted HSI of *Ixodes scapularis* in North America and results of internal and external validation of models.**

(a) Predicted HSI of *Ixodes scapularis* based on BRT model. (b) Predicted HSI and relative uncertainty into four segments by their corresponding 80th, 90th and 95th percentiles, respectively, with the colors from light to deep representing the values from low to high. (c) Occurrence grids after 2020 and potential suitable habitat areas.


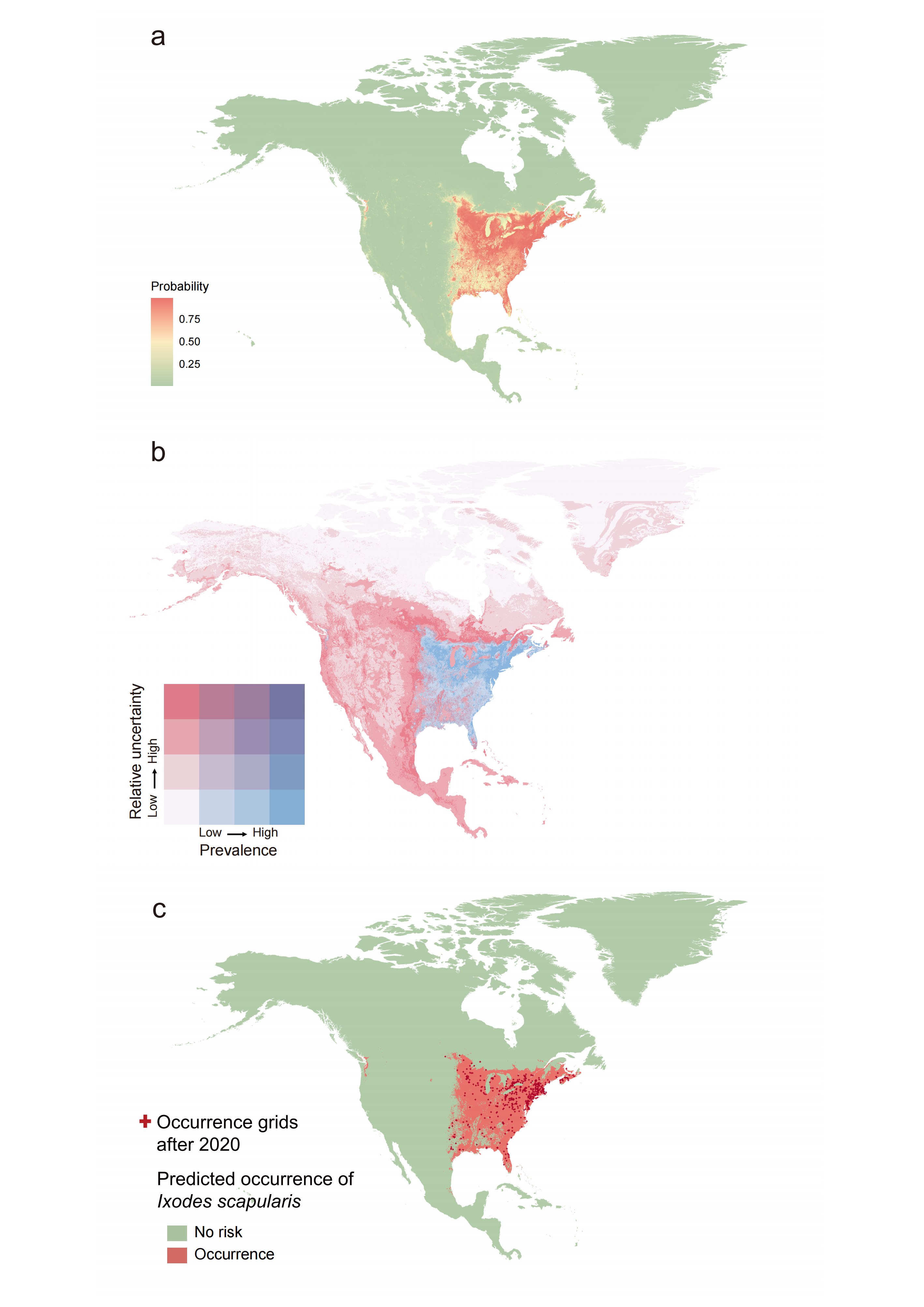


**Supplementary figure 7: The predicted HSI of *Ixodes pacificus* in North America and results of internal and external validation of models.**

(a) Predicted HSI of *Ixodes pacificus* based on BRT model. (b) Predicted HSI and relative uncertainty into four segments by their corresponding 80th, 90th and 95th percentiles, respectively, with the colors from light to deep representing the values from low to high. (c) Occurrence grids after 2020 and potential suitable habitat areas.

**
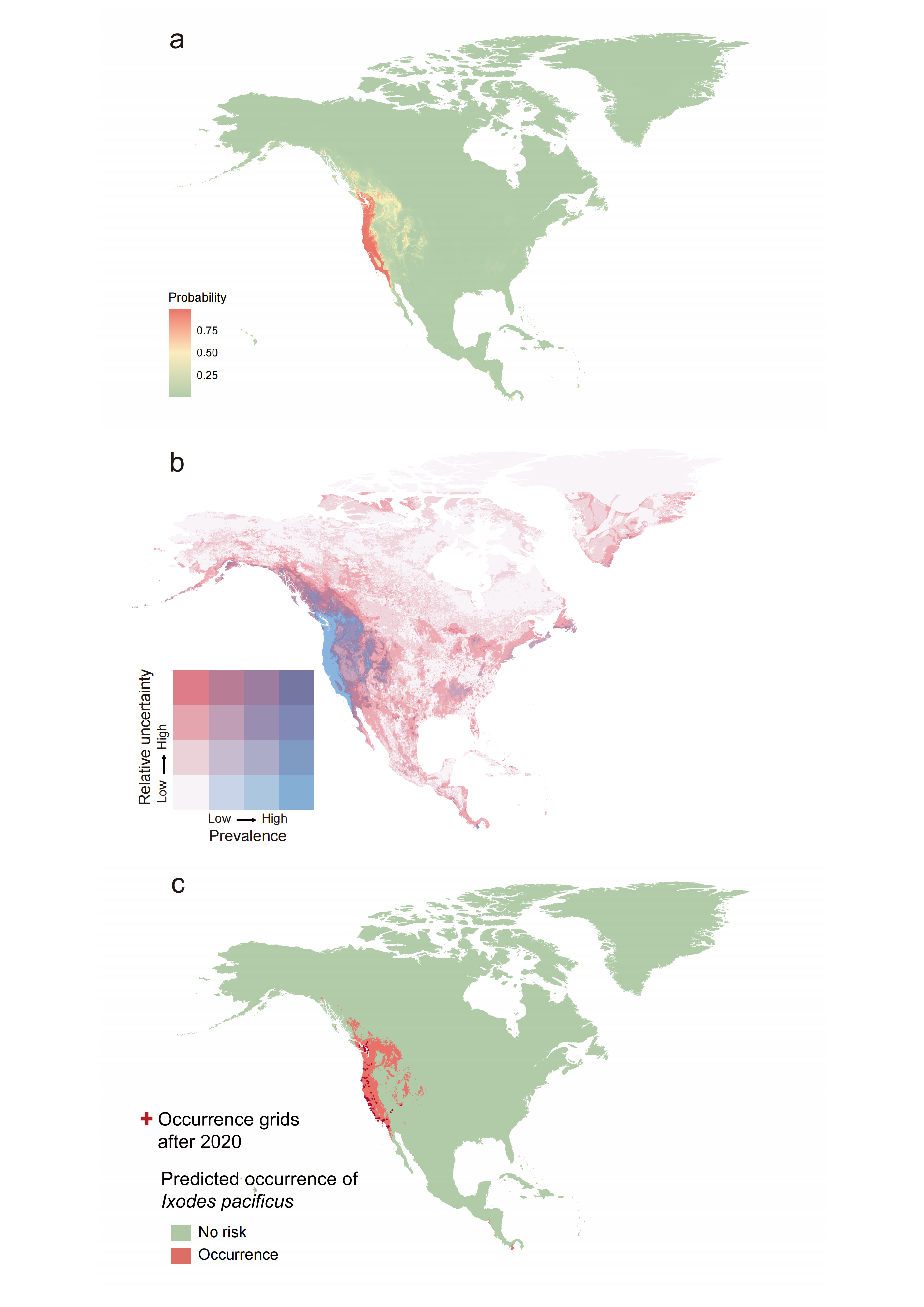
**

**Supplementary figure 8: The predicted HSI of *Ixodes persulcatus* in Eurasia and results of internal and external validation of models.**

(a) Predicted HSI of *Ixodes persulcatus* based on BRT model. (b) Predicted HSI and relative uncertainty into four segments by their corresponding 80th, 90th and 95th percentiles, respectively, with the colors from light to deep representing the values from low to high. (c) Occurrence grids after 2020 and potential suitable habitat areas.


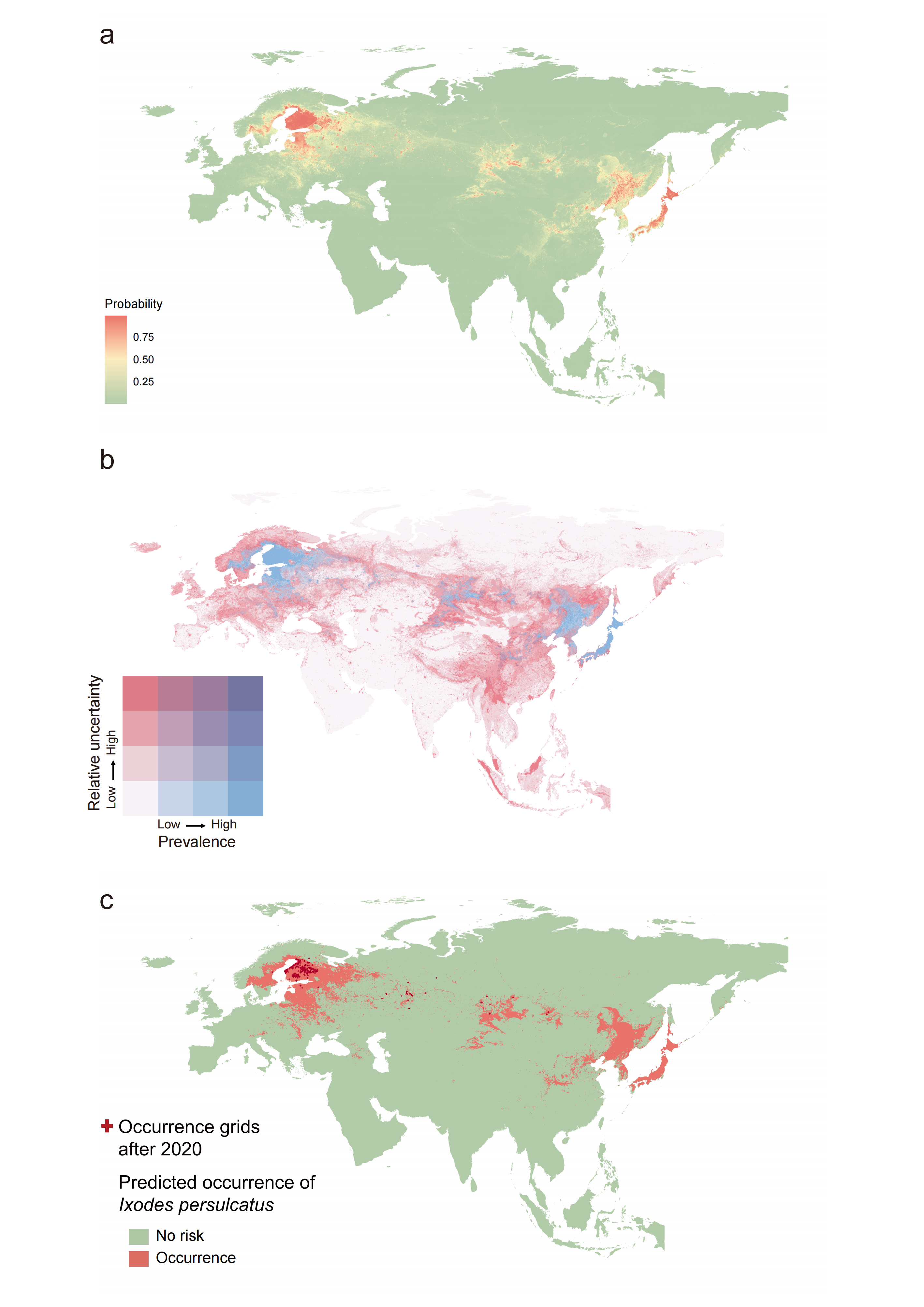


**Supplementary figure 9: The predicted HSI of *Ixodes ricinus* and results of internal and external validation of models.**

(a) Predicted HSI of *Ixodes ricinus* based on BRT model. (b) Predicted HSI and relative uncertainty into four segments by their corresponding 80th, 90th and 95th percentiles, respectively, with the colors from light to deep representing the values from low to high. (c) Occurrence grids after 2020 and potential suitable habitat areas.

**
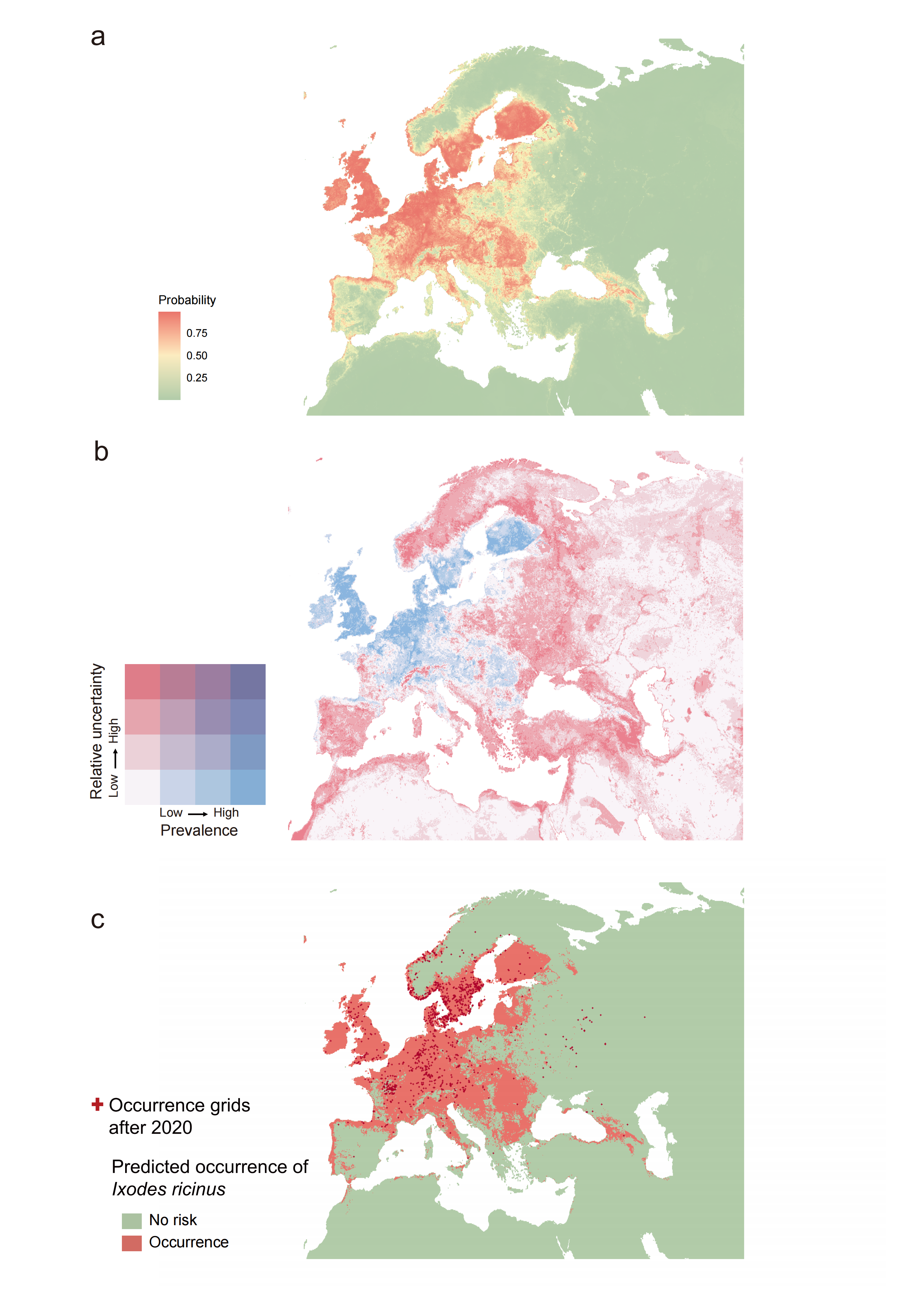
**

**Supplementary figure 10: The predicted HSI of *Haemaphysalis longicornis* at worldwide and results of internal and external validation of models.**

(a) Predicted HSI of *Haemaphysalis longicornis* based on BRT model. (b) Predicted HSI and relative uncertainty into four segments by their corresponding 80th, 90th and 95th percentiles, respectively, with the colors from light to deep representing the values from low to high. (c) Occurrence grids after 2020 and potential suitable habitat areas.


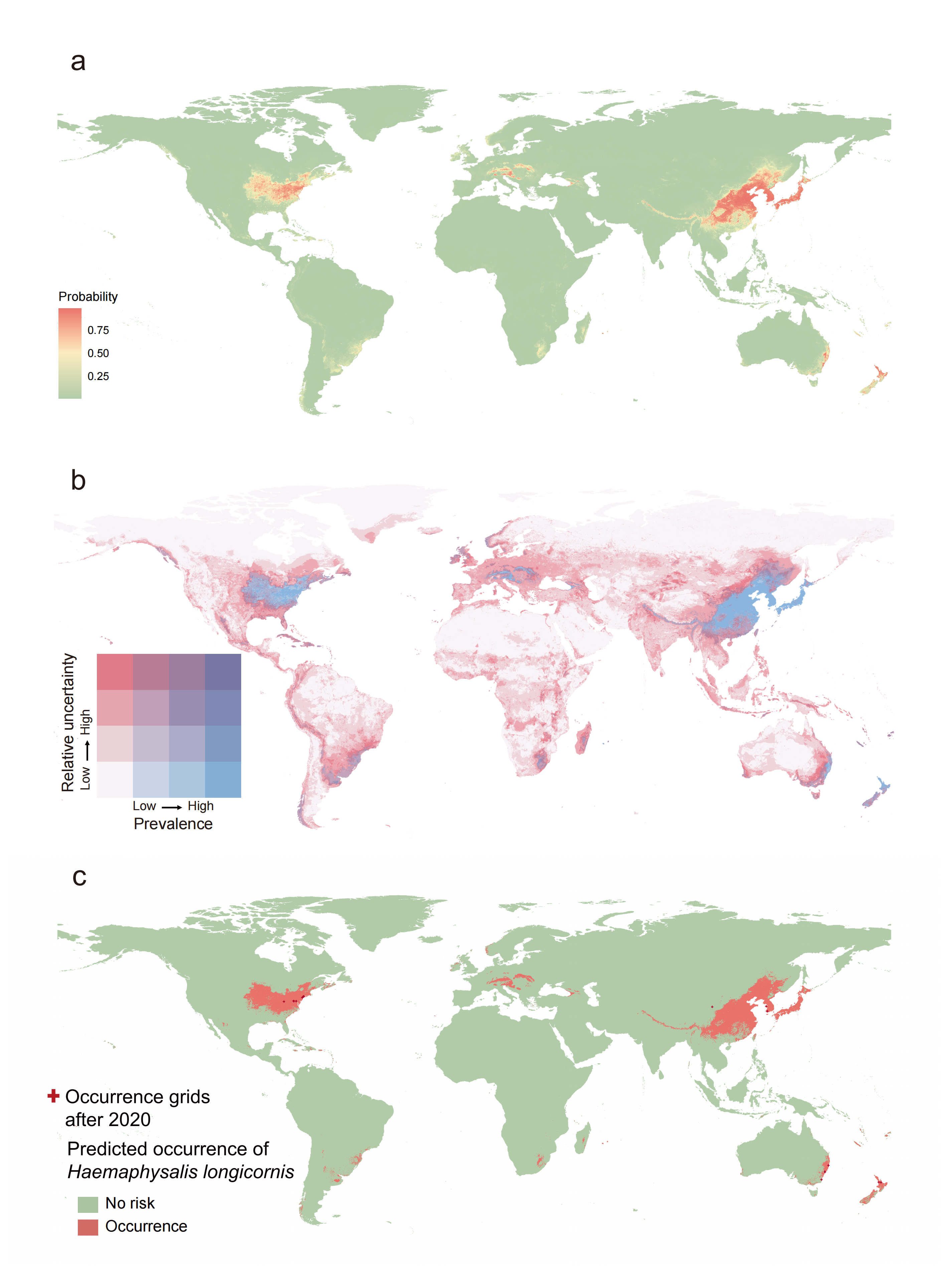


**Supplementary figure 11: The predicted HSI of *Dermacentor marginatus* in Eurasia and Africa and results of internal and external validation of models.**

(a) Predicted HSI of *Dermacentor marginatus* based on BRT model. (b) Predicted HSI and relative uncertainty into four segments by their corresponding 80th, 90th and 95th percentiles, respectively, with the colors from light to deep representing the values from low to high. (c) Occurrence grids after 2020 and potential suitable habitat areas.


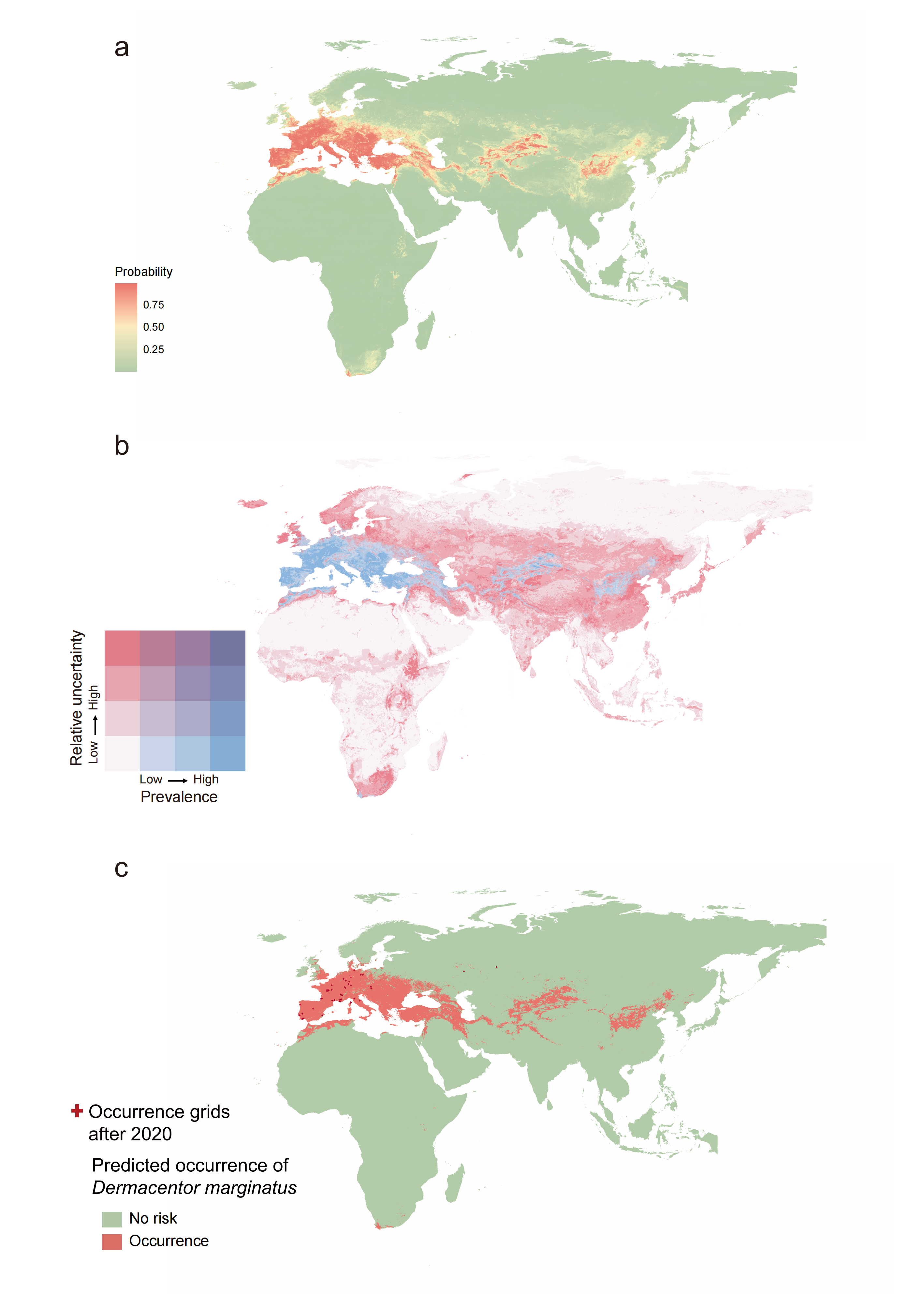


**Supplementary figure 12: The predicted HSI of *Rhipicephalus microplus* at worldwide and results of internal and external validation of models.**

(a) Predicted HSI of *Rhipicephalus microplus* based on BRT model. (b) Predicted HSI and relative uncertainty into four segments by their corresponding 80th, 90th and 95th percentiles, respectively, with the colors from light to deep representing the values from low to high. (c) Occurrence grids after 2020 and potential suitable habitat areas.


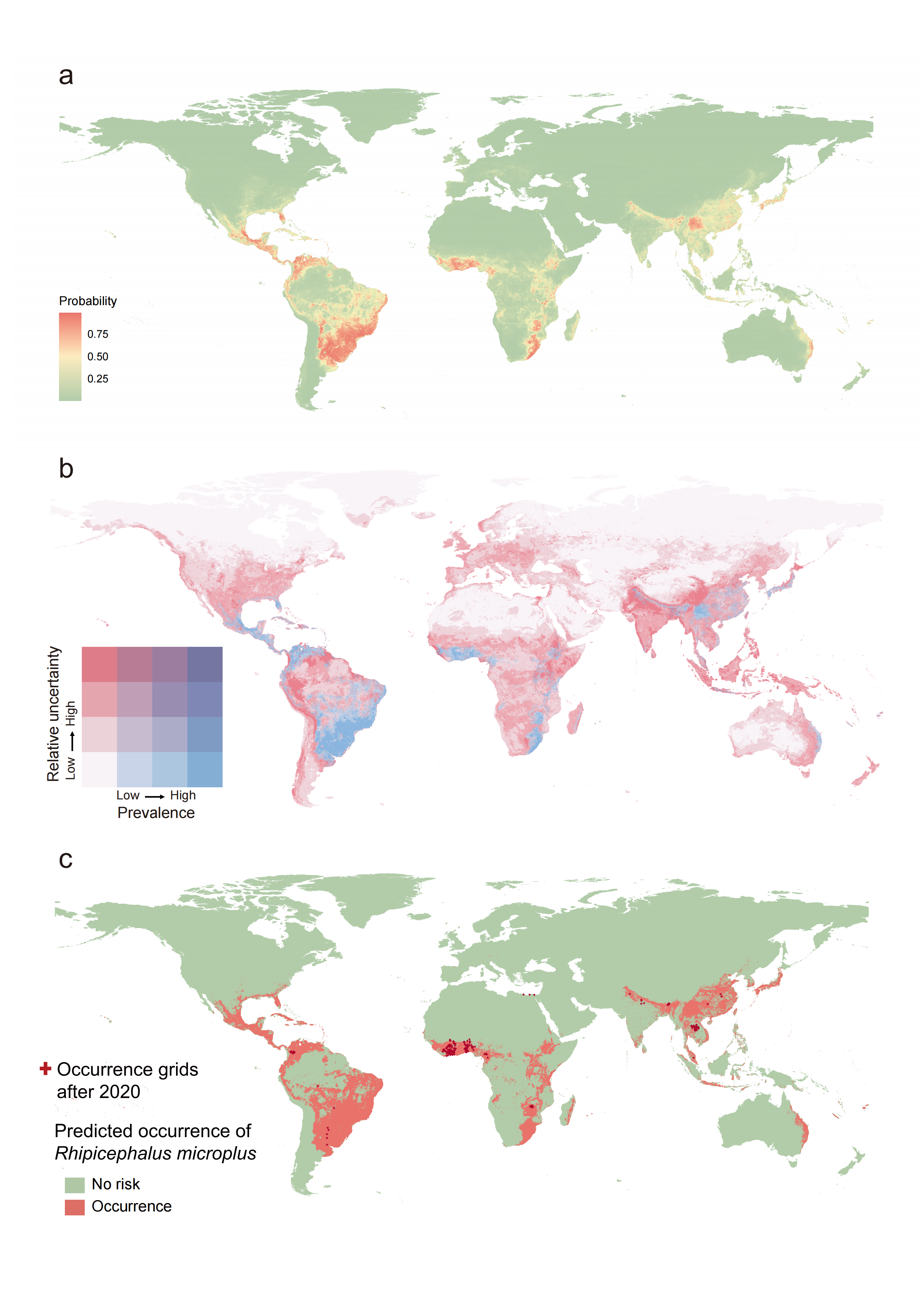


**Supplementary figure 13: The predicted HSI of *Rhipicephalus sanguineus* at worldwide and results of internal and external validation of models.**

(a) Predicted HSI of *Rhipicephalus sanguineus* based on BRT model. (b) Predicted HSI and relative uncertainty into four segments by their corresponding 80th, 90th and 95th percentiles, respectively, with the colors from light to deep representing the values from low to high. (c) Occurrence grids after 2020 and potential suitable habitat areas.


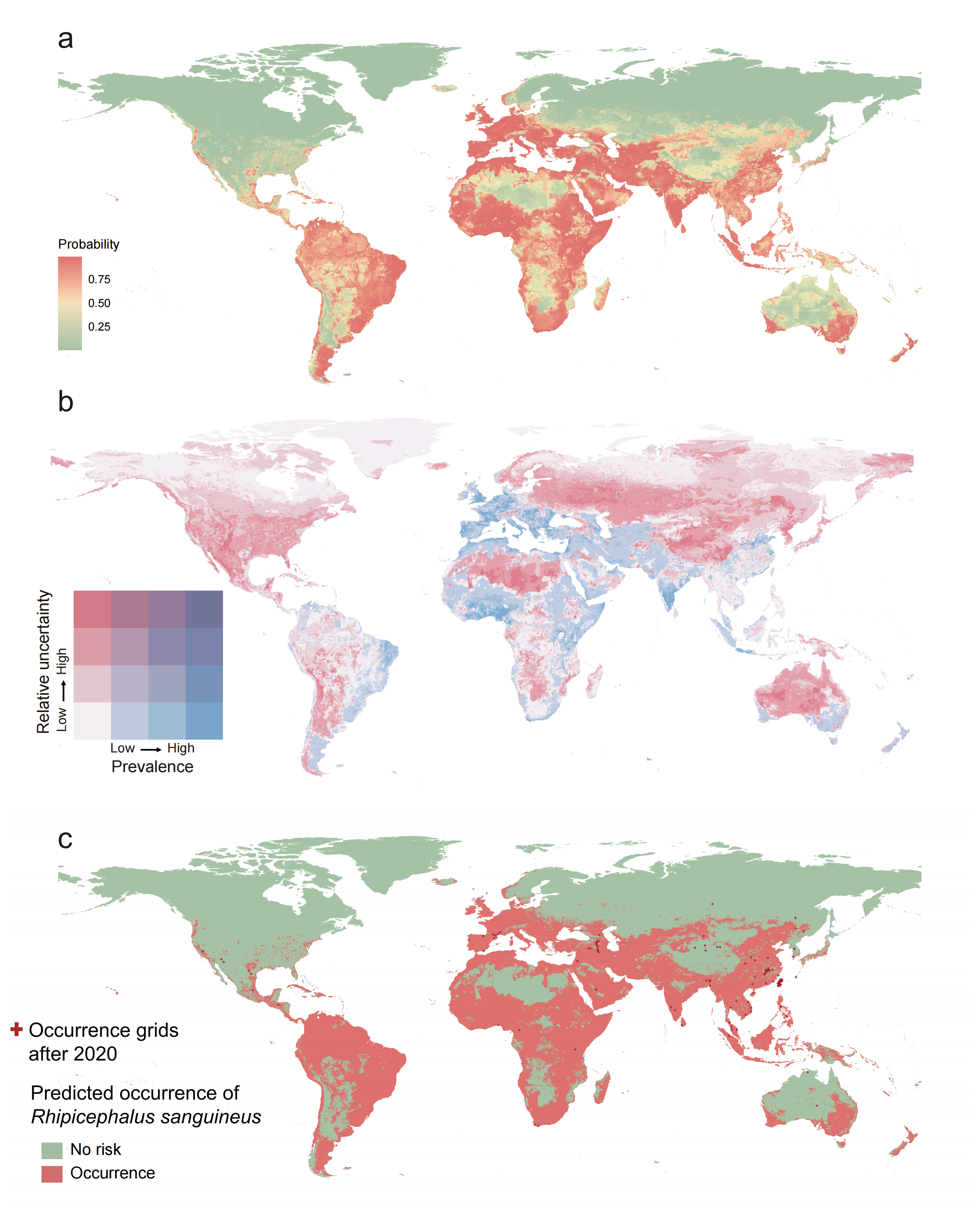


**Supplementary figure 14: Effects of major predictors (RCs >3%) for presence of *Ixodes scapularis* based on BRT models.**

The mean curves (red) and 95% percentiles (purple, ecoclimatic variables; green, environmental variables; blue, biological variables) show the predicted habitat suitability index at the logit scale. The histograms show the frequency distributions of the predictors.


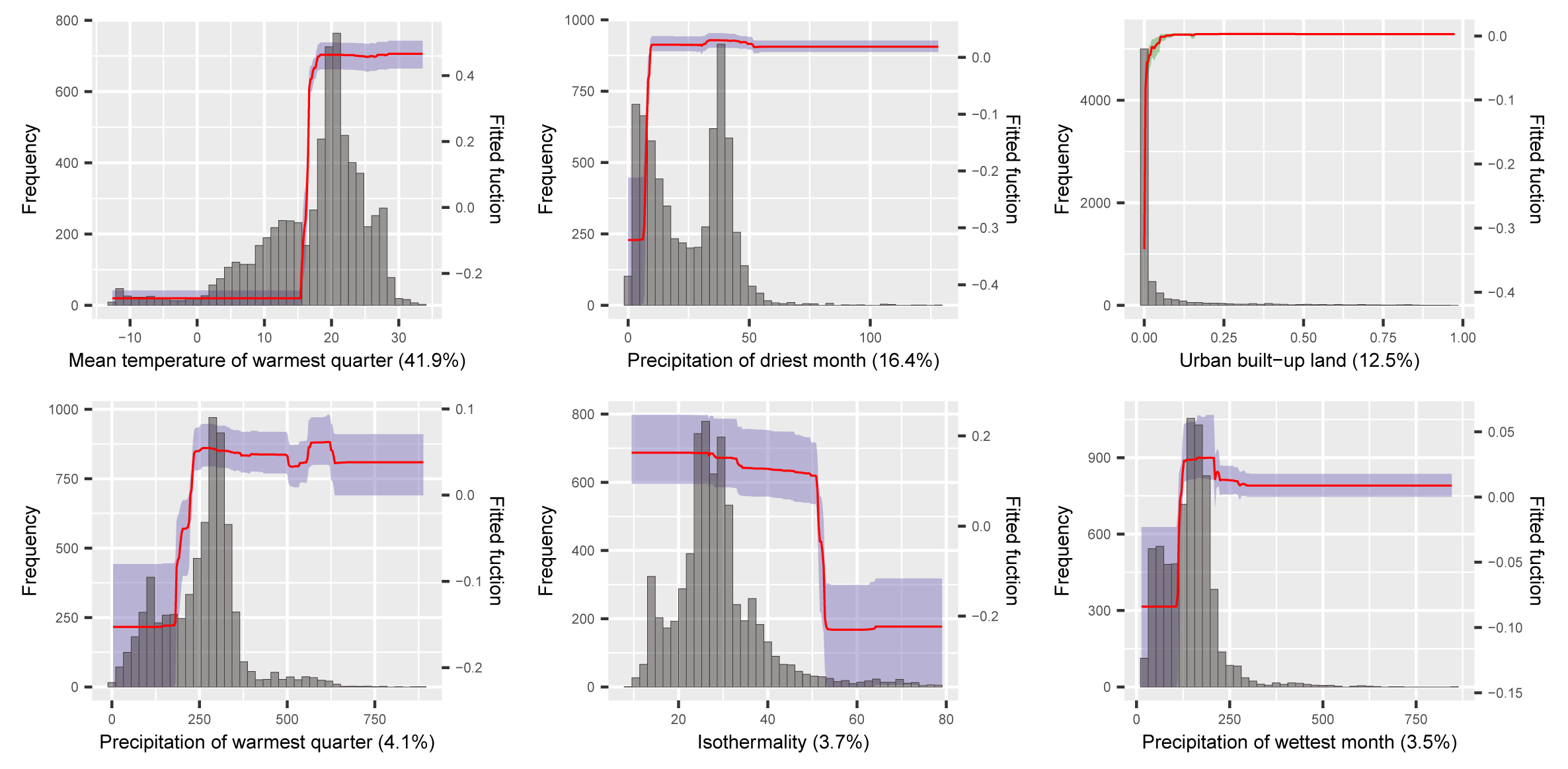


**Supplementary figure 15: Effects of major predictors (RCs >3%) for presence of *Ixodes pacificus* based on BRT models.**

The mean curves (red) and 95% percentiles (purple, ecoclimatic variables; green, environmental variables; blue, biological variables) show the predicted habitat suitability index at the logit scale. The histograms show the frequency distributions of the predictors.


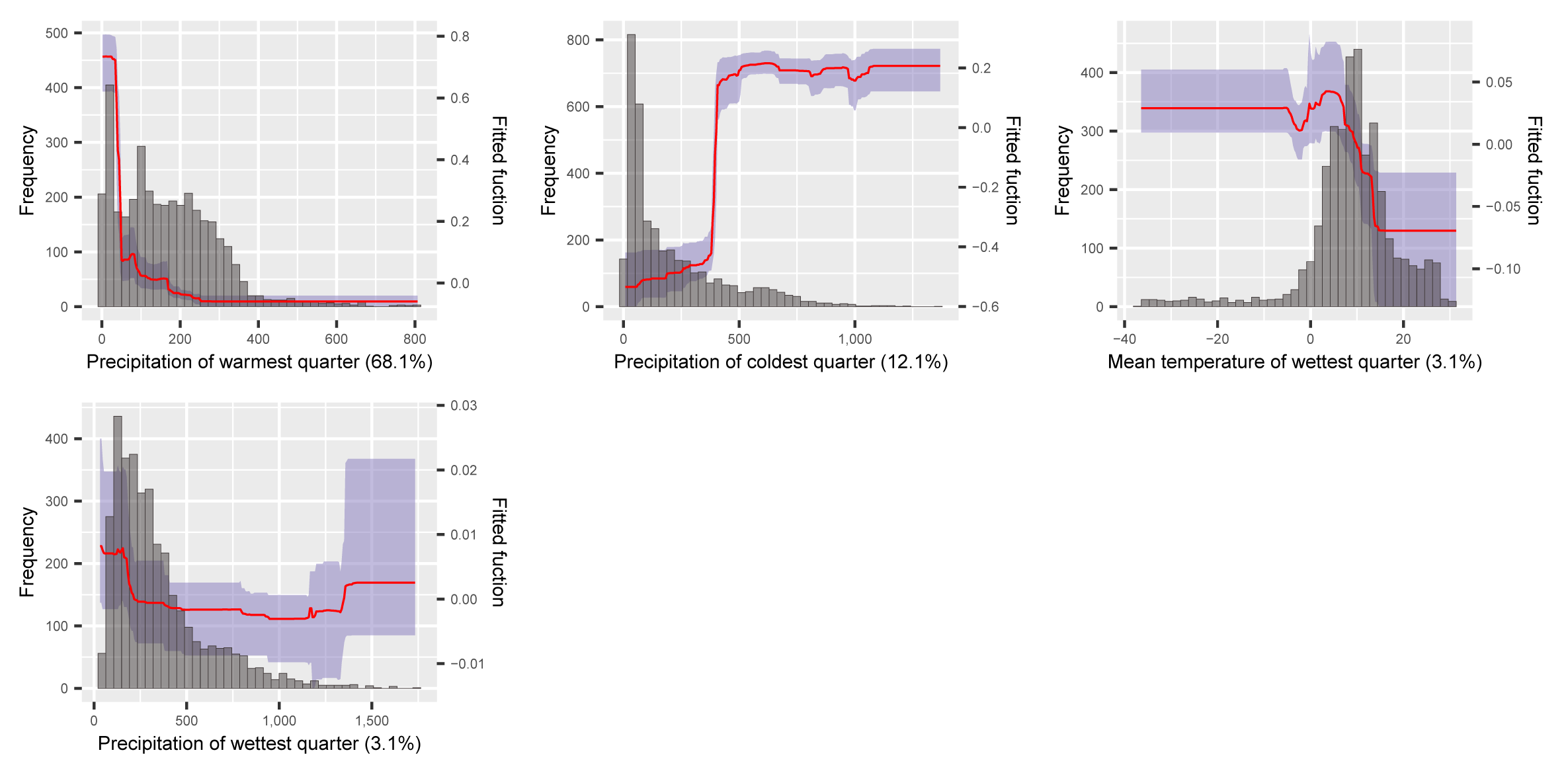


**Supplementary figure 16: Effects of major predictors (RCs >3%) for presence of *Ixodes persulcatus* based on BRT models.**

The mean curves (red) and 95% percentiles (purple, ecoclimatic variables; green, environmental variables; blue, biological variables) show the predicted habitat suitability index at the logit scale. The histograms show the frequency distributions of the predictors.


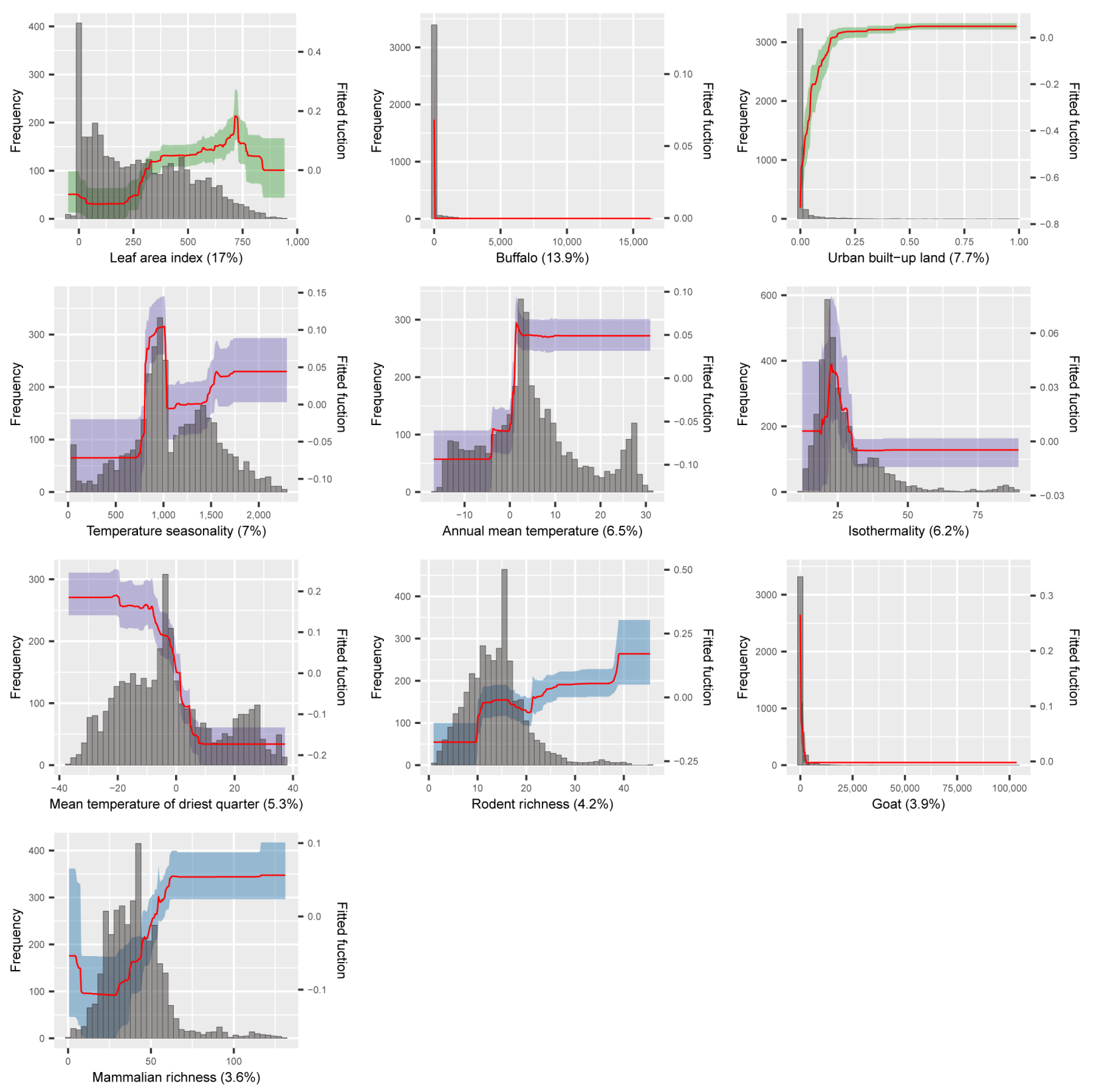


**Supplementary figure 17: Effects of major predictors (RCs >3%) for presence of *Ixodes ricinus* based on BRT models.**

The mean curves (red) and 95% percentiles (purple, ecoclimatic variables; green, environmental variables; blue, biological variables) show the predicted habitat suitability index at the logit scale. The histograms show the frequency distributions of the predictors.


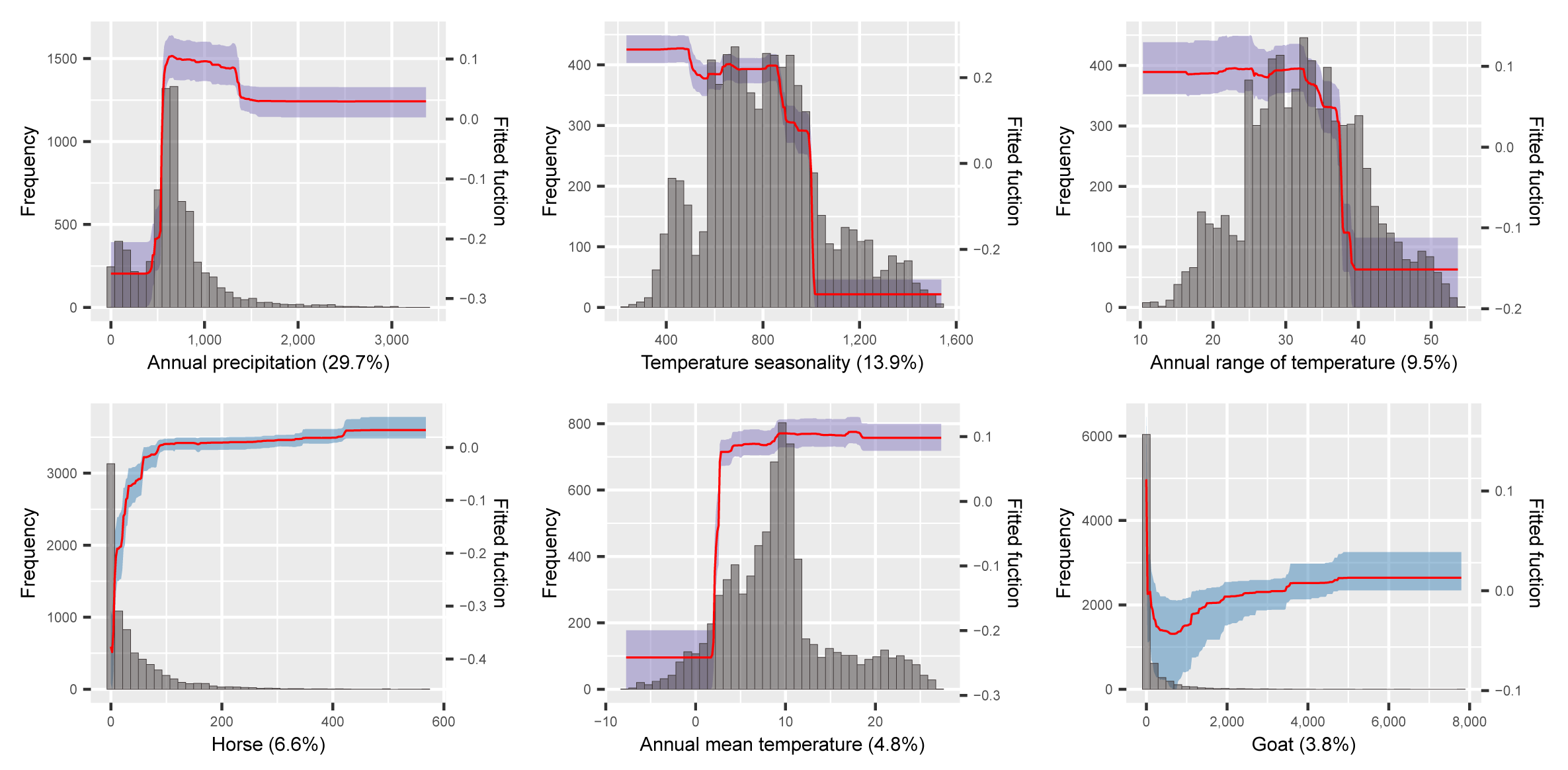


**Supplementary figure 18: Effects of major predictors (RCs >3%) for presence of *Haemaphysalis longicornis* based on BRT models.**

The mean curves (red) and 95% percentiles (purple, ecoclimatic variables; green, environmental variables; blue, biological variables) show the predicted habitat suitability index at the logit scale. The histograms show the frequency distributions of the predictors.


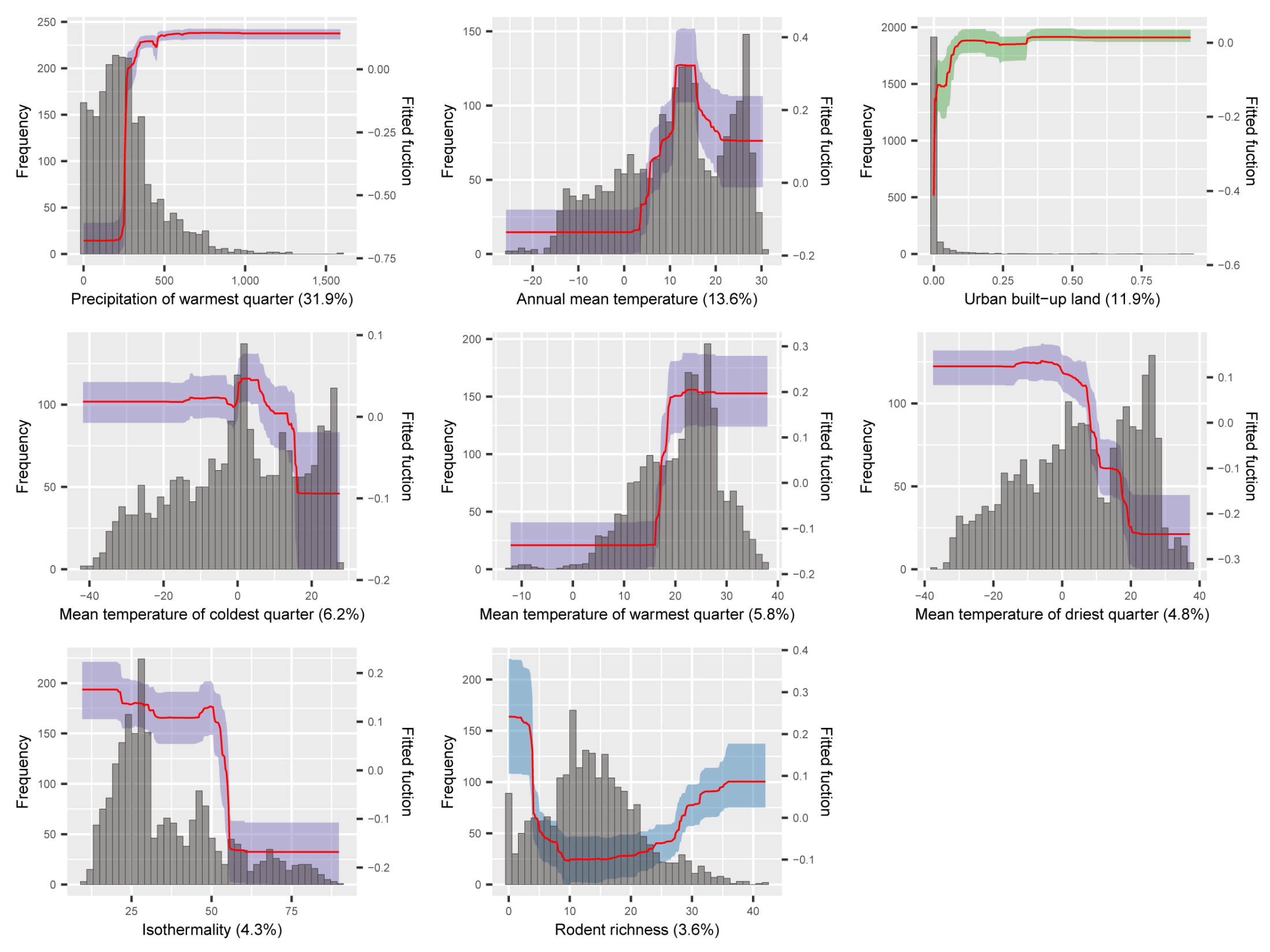


**Supplementary figure 19: Effects of major predictors (RCs >3%) for presence of *Dermacentor marginatus* based on BRT models.**

The mean curves (red) and 95% percentiles (purple, ecoclimatic variables; green, environmental variables; blue, biological variables) show the predicted habitat suitability index at the logit scale. The histograms show the frequency distributions of the predictors.


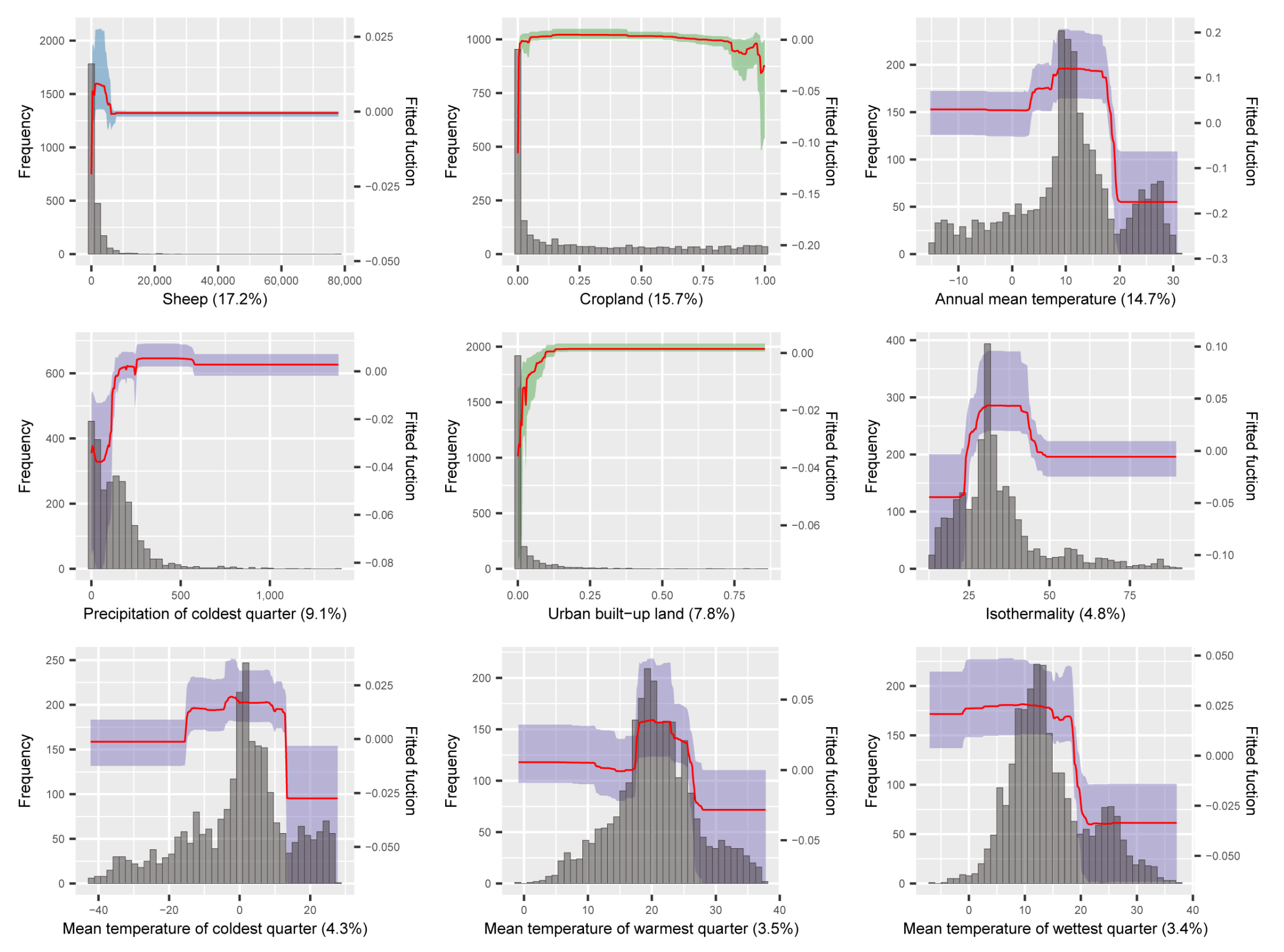


**Supplementary figure 20: Effects of major predictors (RCs >3%) for presence of *Rhipicephalus microplus* based on BRT models.**

The mean curves (red) and 95% percentiles (purple, ecoclimatic variables; green, environmental variables; blue, biological variables) show the predicted habitat suitability index at the logit scale. The histograms show the frequency distributions of the predictors.


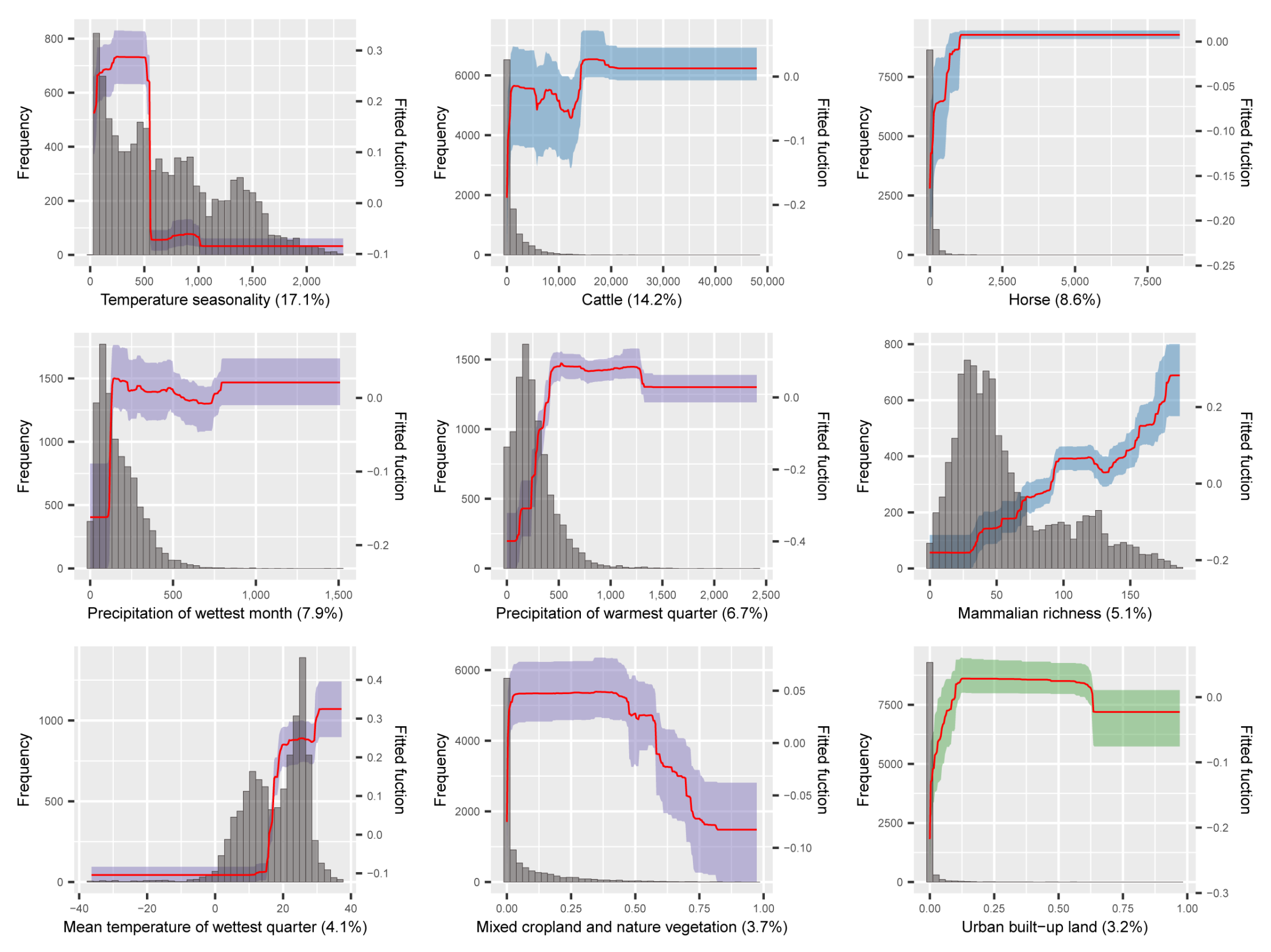


**Supplementary figure 21: Effects of major predictors (RCs >3%) for presence of *Rhipicephalus sanguineus* based on BRT models.**

The mean curves (red) and 95% percentiles (purple, ecoclimatic variables; green, environmental variables; blue, biological variables) show the predicted habitat suitability index at the logit scale. The histograms show the frequency distributions of the predictors.


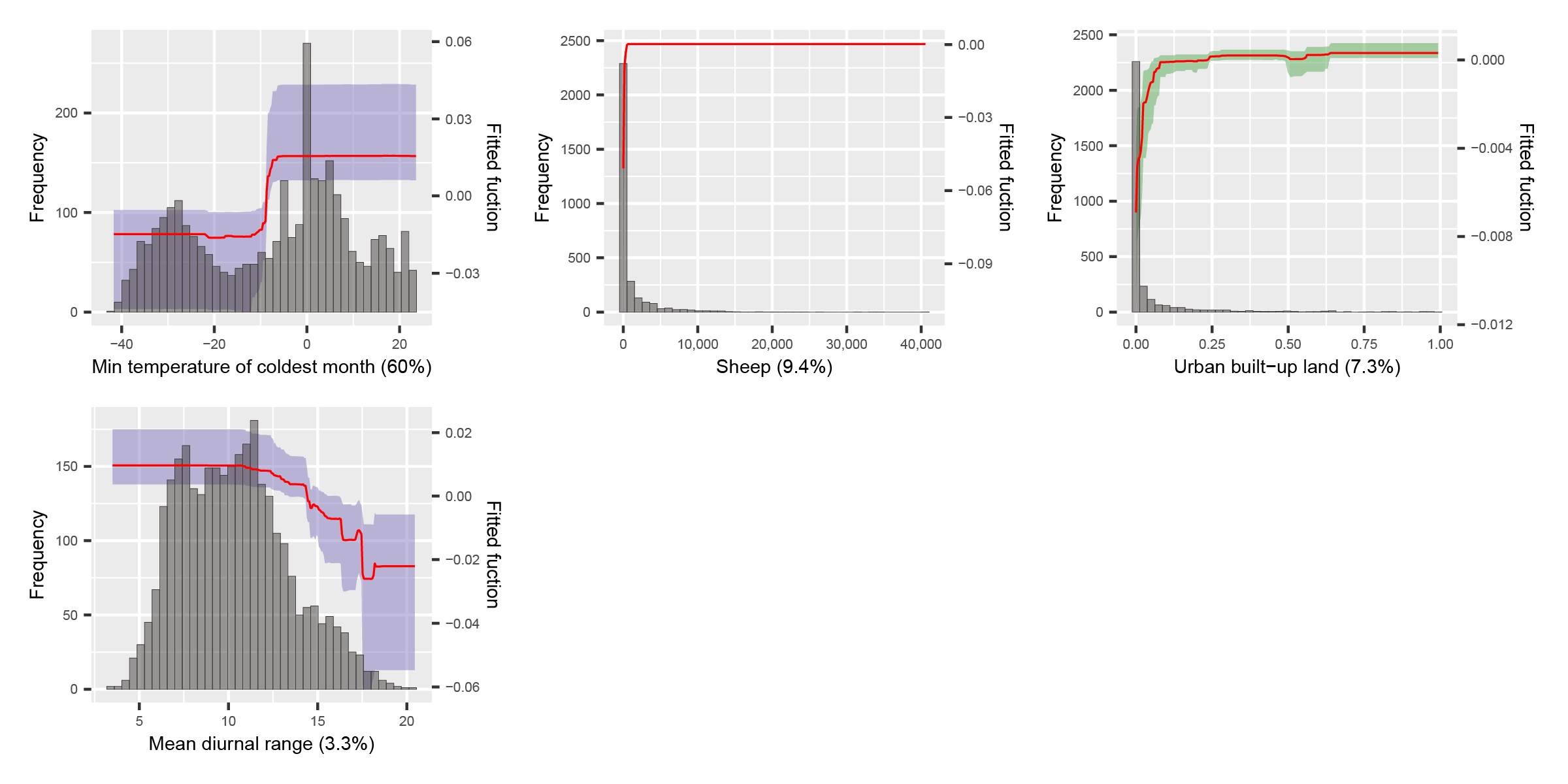


# **Supplementary figure 22: Predictive performance of the three machine-learning algorithms.**

ROC curves and AUC values of the BRT (left, red), RF (middle, blue) and LASSO regression (right, orange) over 100 models are shown. Roman numerals 1-6 correspond to the six major Anaplasmataceae species: (Ⅰ) *A. phagocytophilum*; (Ⅱ) *Candidatus.* N. mikurensis; (Ⅲ) *E. canis*; (Ⅳ) *A. ovis*; (Ⅴ) *A. platys*; (Ⅵ) *A. marginale*.


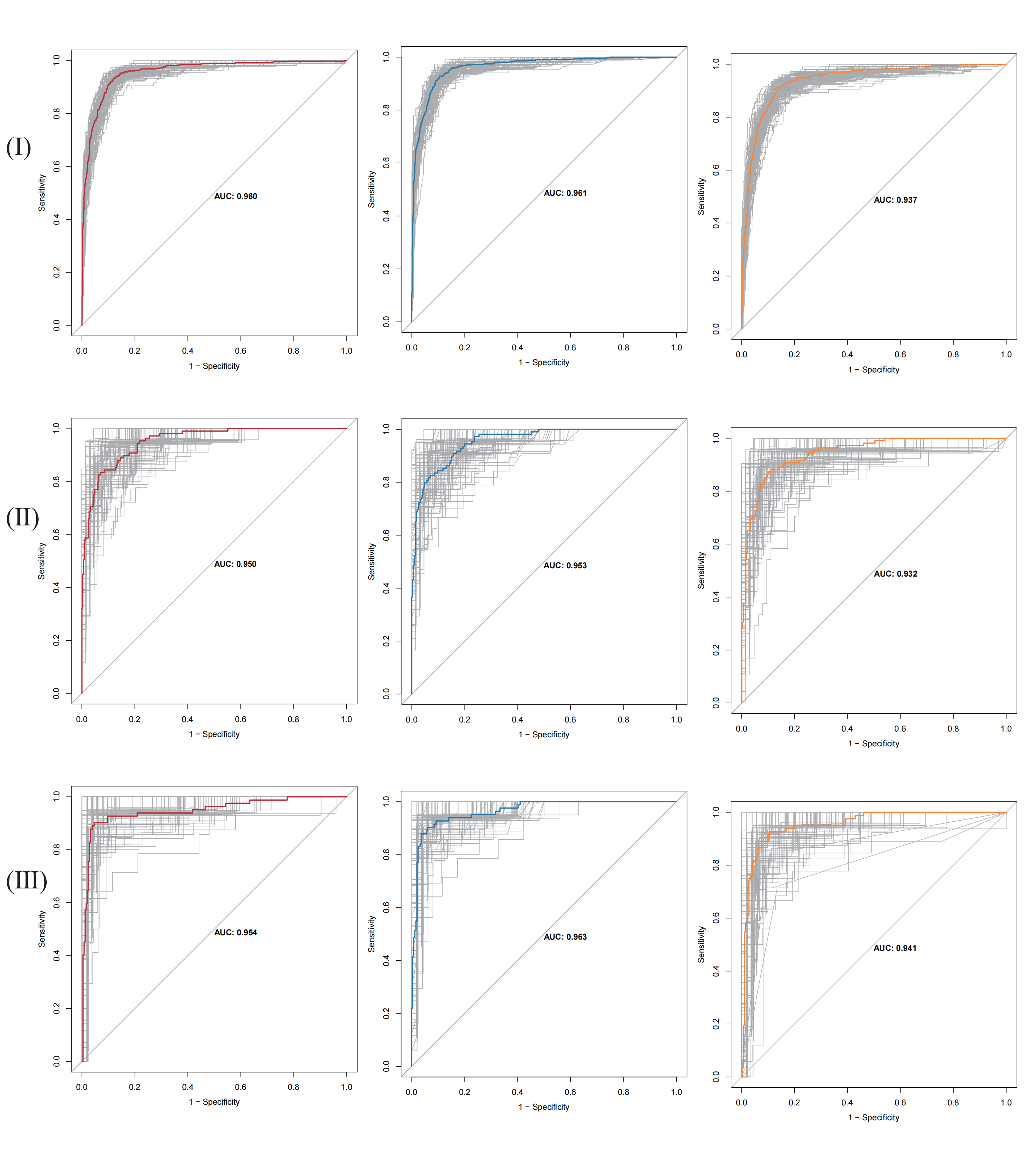


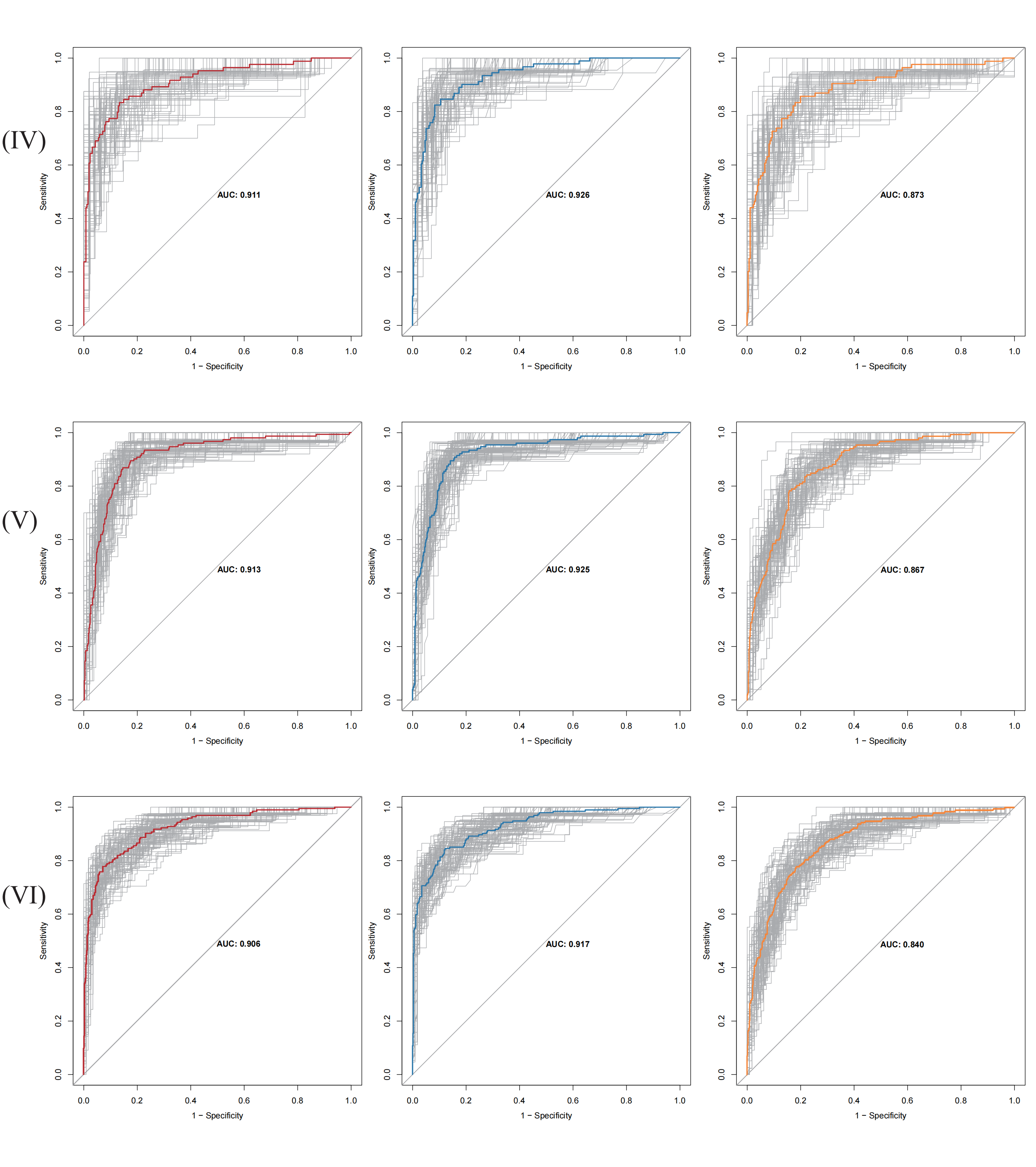


ROC, receiver operating characteristic. AUC, area under the curve. BRT, boosted regression trees. RF, random forest. LASSO, least absolute shrinkage and selection operator.

**Supplementary figure 23: Effects of major predictors (RCs >3%) for presence of *Anaplasma phagocytophilum* based on RF model.**

The mean curves (red) and 95% percentiles (purple, ecoclimatic variables; green, environmental variables; blue, biological variables; red, socioeconomic variables) show the predicted probability of occurrence at the logit scale. The histograms show the frequency distributions of the predictors.


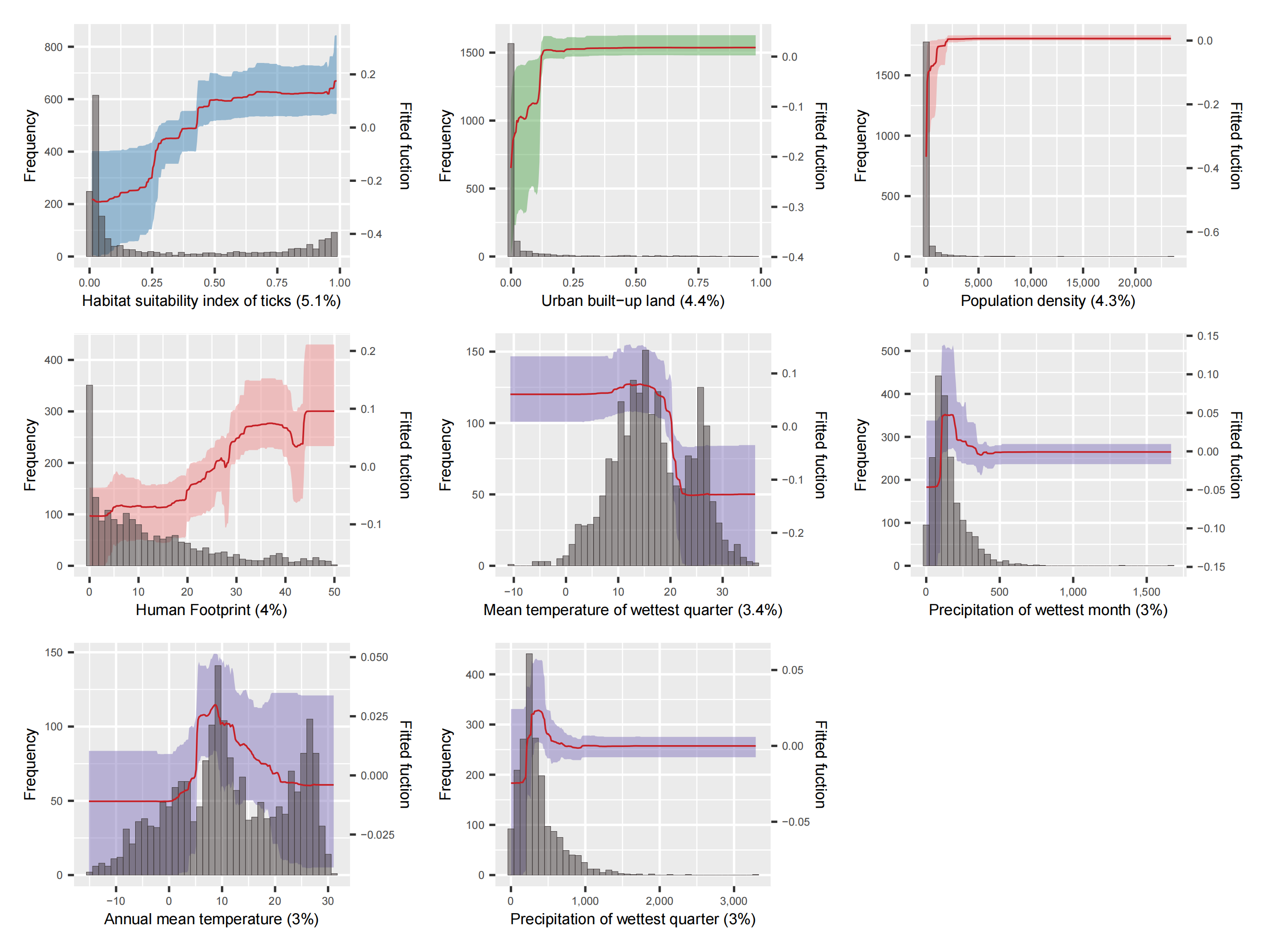


RC, relative contribution. **RF, random forest.**

**Supplementary figure 24: Effects of major predictors (RCs >3%) for presence of *Candidatus* Neoehrlichia mikurensis based on RF models.**

The mean curves (red) and 95% percentiles (purple, ecoclimatic variables; green, environmental variables; blue, biological variables; red, socioeconomic variables) show the predicted probability of occurrence at the logit scale. The histograms show the frequency distributions of the predictors.


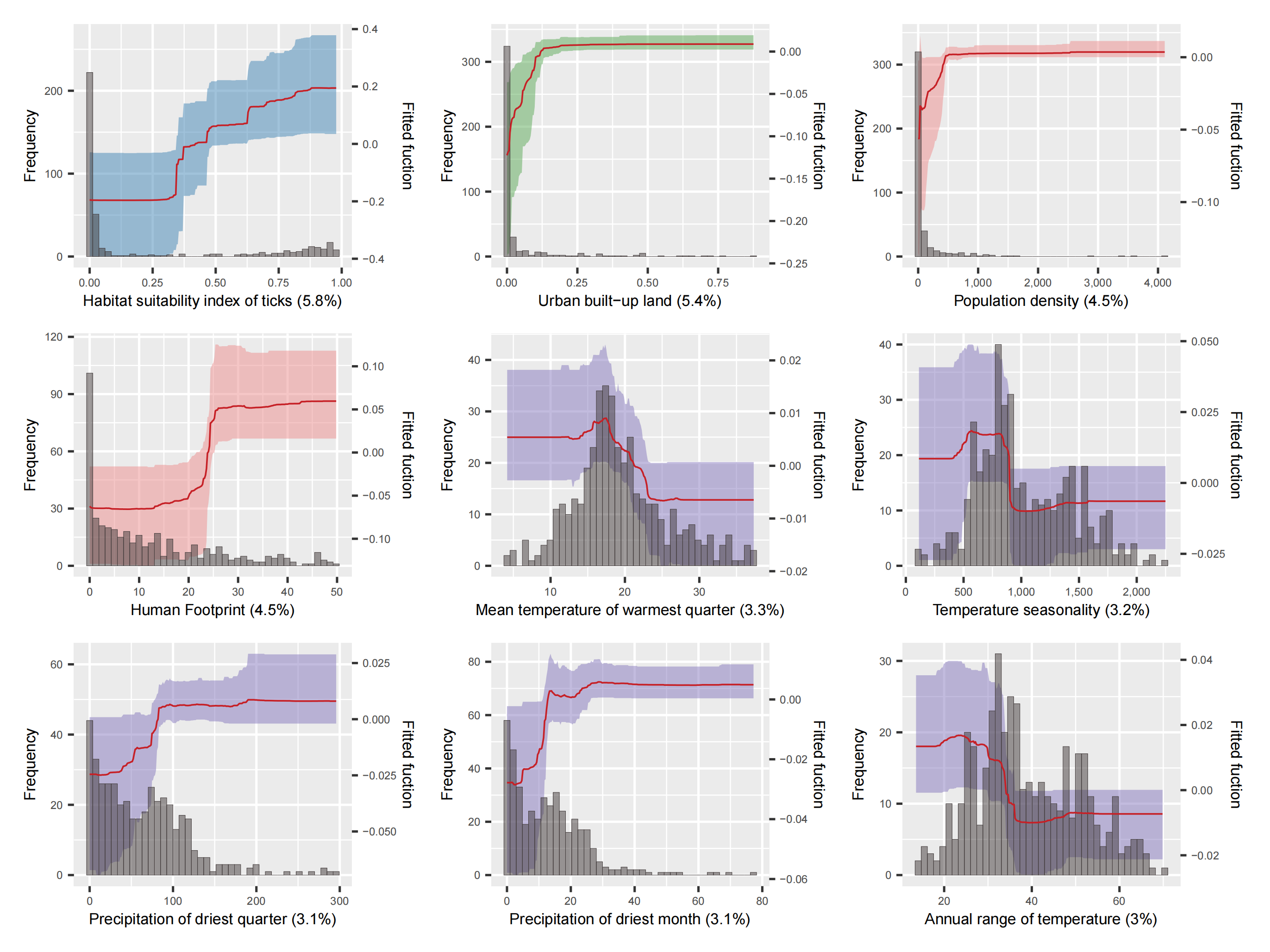


**Supplementary figure 25: Effects of major predictors (RCs >3%) for presence of *Ehrlichia canis* based on RF models.**

The mean curves (red) and 95% percentiles (purple, ecoclimatic variables; green, environmental variables; blue, biological variables; red, socioeconomic variables) show the predicted probability of occurrence at the logit scale. The histograms show the frequency distributions of the predictors.


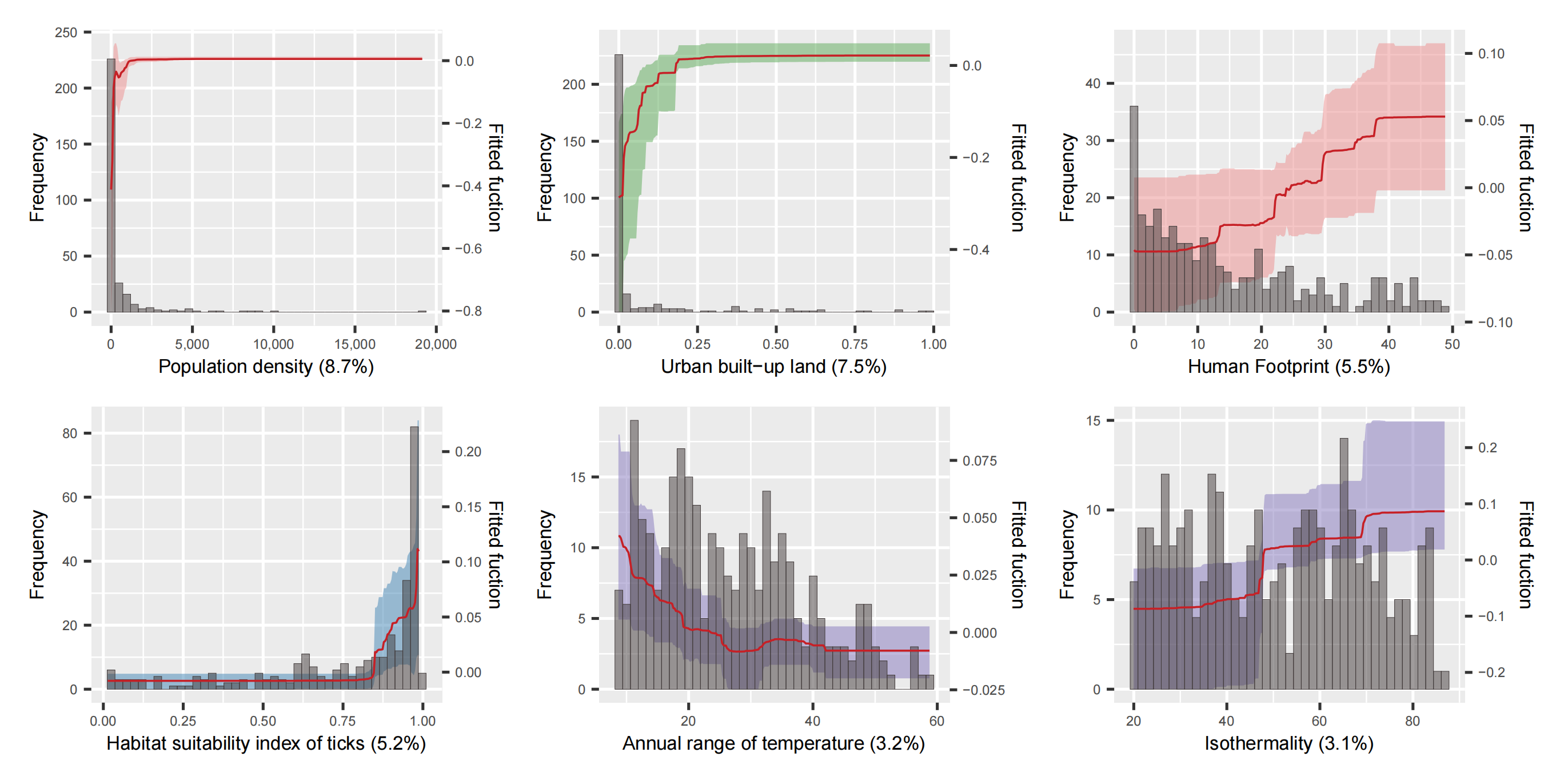


**Supplementary figure 26: Effects of major predictors (RCs >3%) for presence of *Anaplasma ovis* based on RF models.**

The mean curves (red) and 95% percentiles (purple, ecoclimatic variables; green, environmental variables; blue, biological variables; red, socioeconomic variables) show the predicted probability of occurrence at the logit scale. The histograms show the frequency distributions of the predictors.


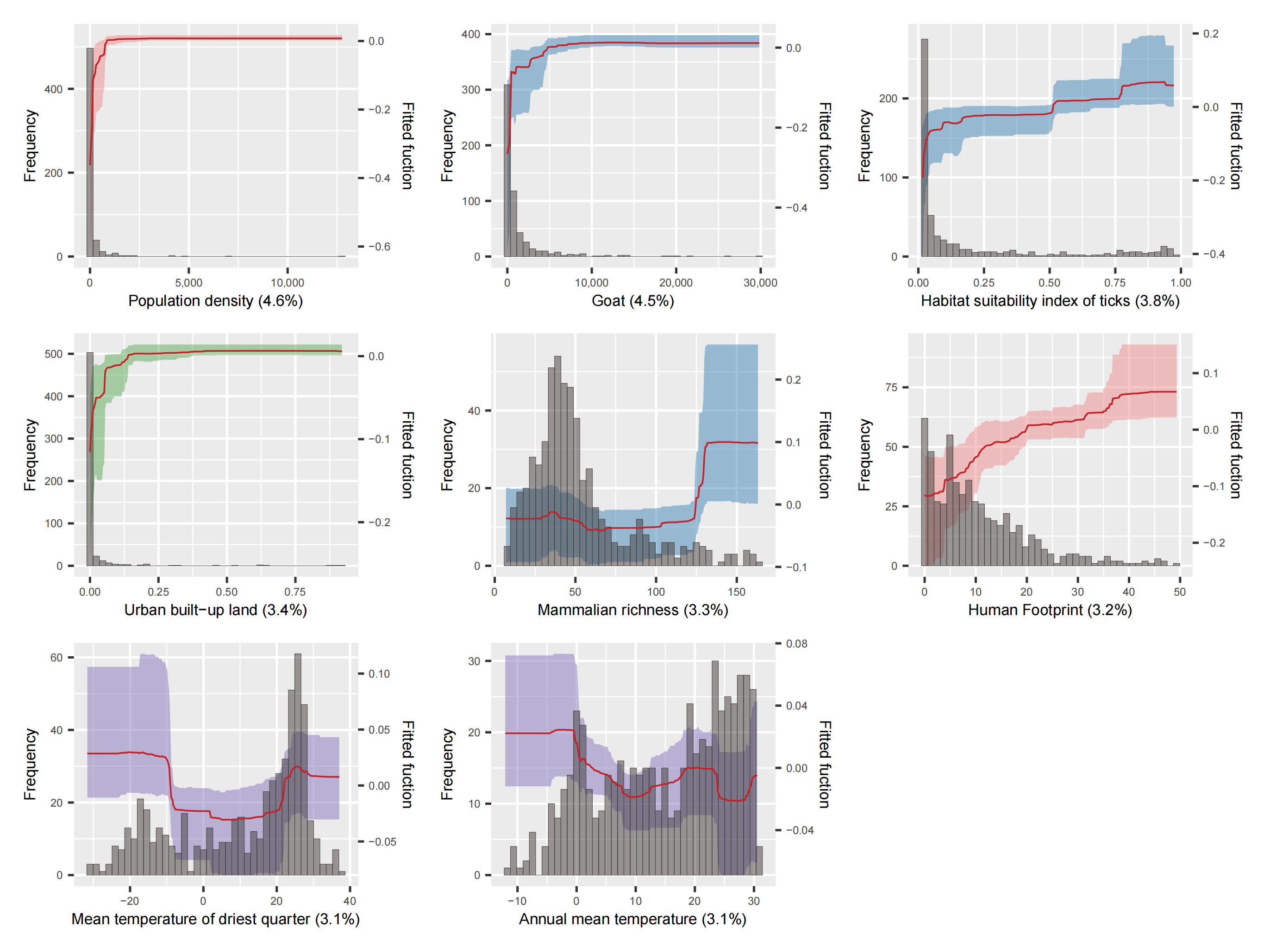


**Supplementary figure 27: Effects of major predictors (RCs >3%) for presence of *Anaplasma platys* based on RF models.**

The mean curves (red) and 95% percentiles (purple, ecoclimatic variables; green, environmental variables; blue, biological variables; red, socioeconomic variables) show the predicted probability of occurrence at the logit scale. The histograms show the frequency distributions of the predictors.


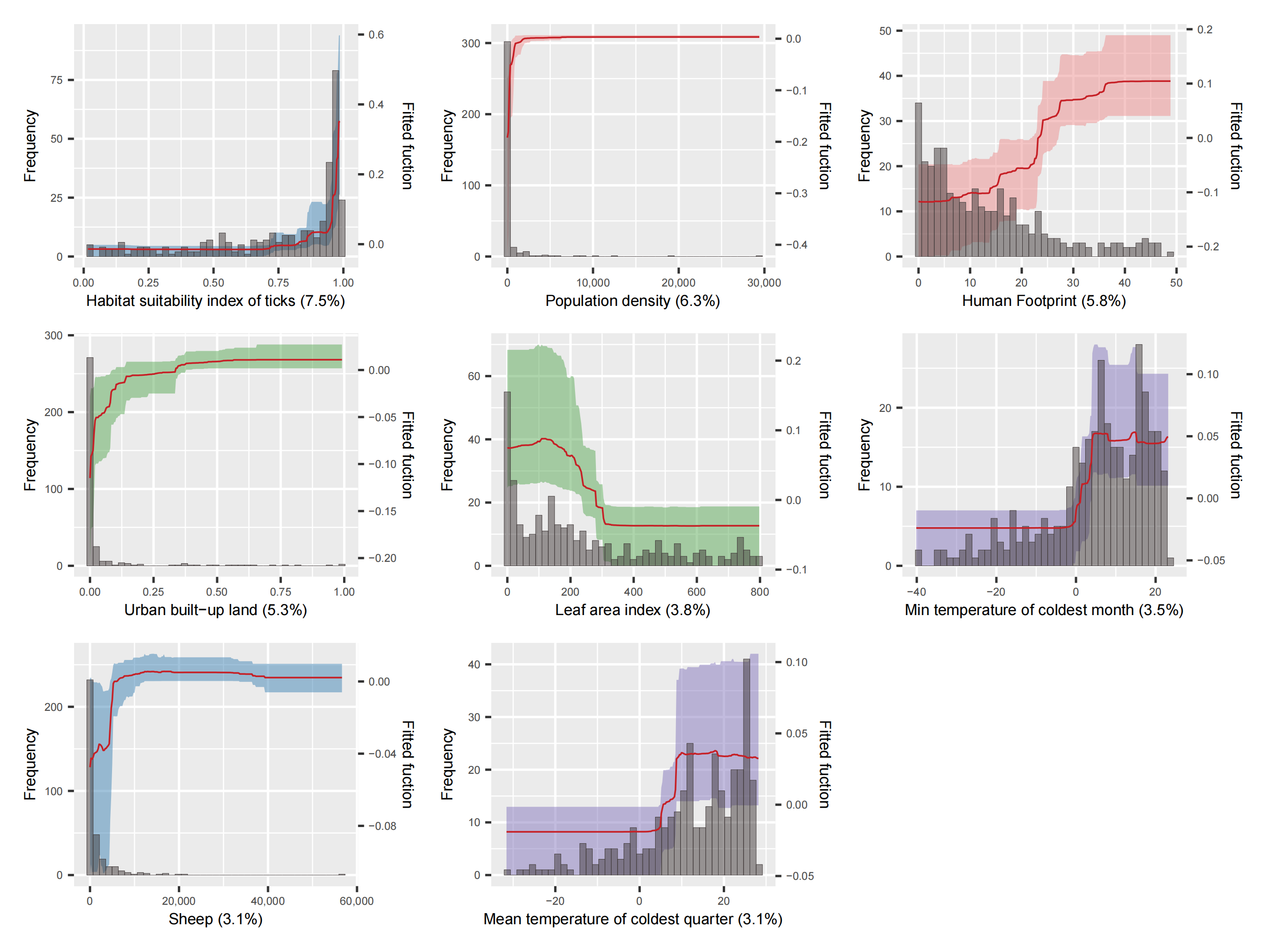


**Supplementary figure 28: Effects of major predictors (RCs >3%) for presence of *Anaplasma marginale* based on RF models.**

The mean curves (red) and 95% percentiles (purple, ecoclimatic variables; green, environmental variables; blue, biological variables; red, socioeconomic variables) show the predicted probability of occurrence at the logit scale. The histograms show the frequency distributions of the predictors.


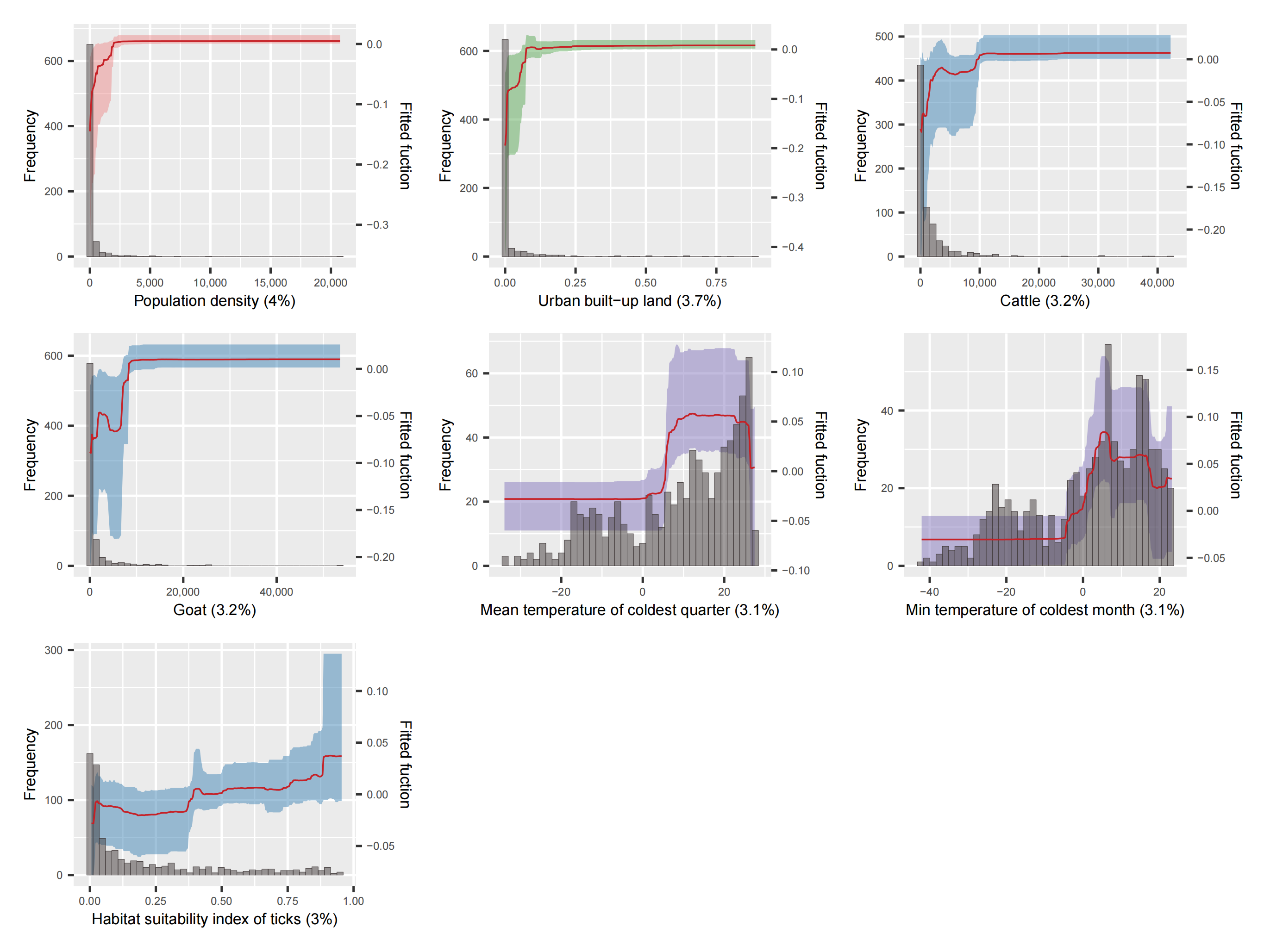


# **Supplementary figure 29: The global recorded and predicted distributions of *A. phagocytophilum*.**

(a-c) Recorded locations of *A. phagocytophilum* which was detected from arthropod vectors, animal hosts and human beings. (d) Heat map of predicted relative risk distribution based on RF models about *A. phagocytophilum*.


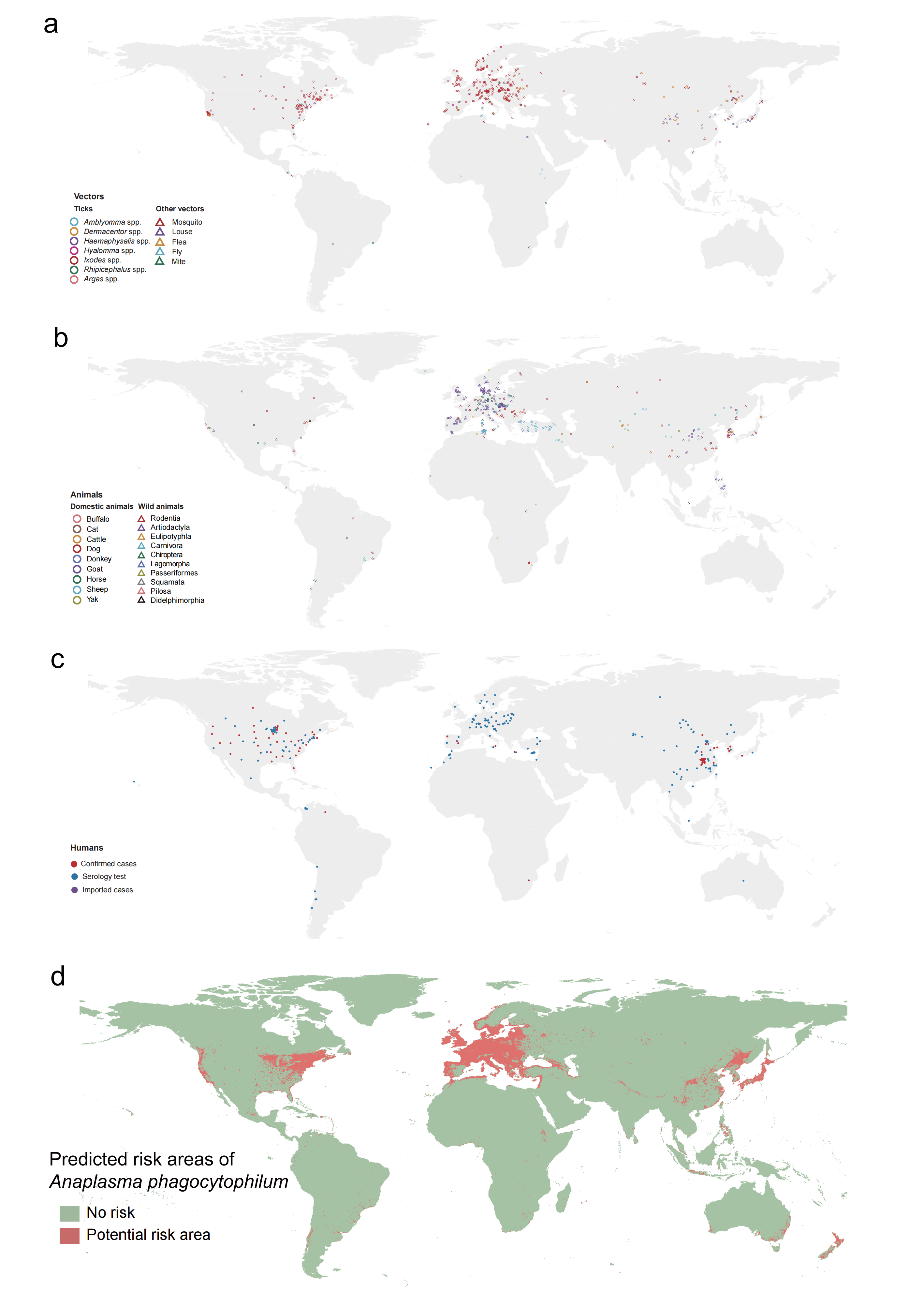


**Supplementary figure 30: The recorded and predicted distributions of *Candidatus* N. mikurensis in Eurasia and Africa.**

(a-c) Recorded locations of *Candidatus* N. mikurensis which was detected from arthropod vectors, animal hosts and human beings. (d) Heat map of predicted relative risk distribution based on RF models about *Candidatus* N. mikurensis.


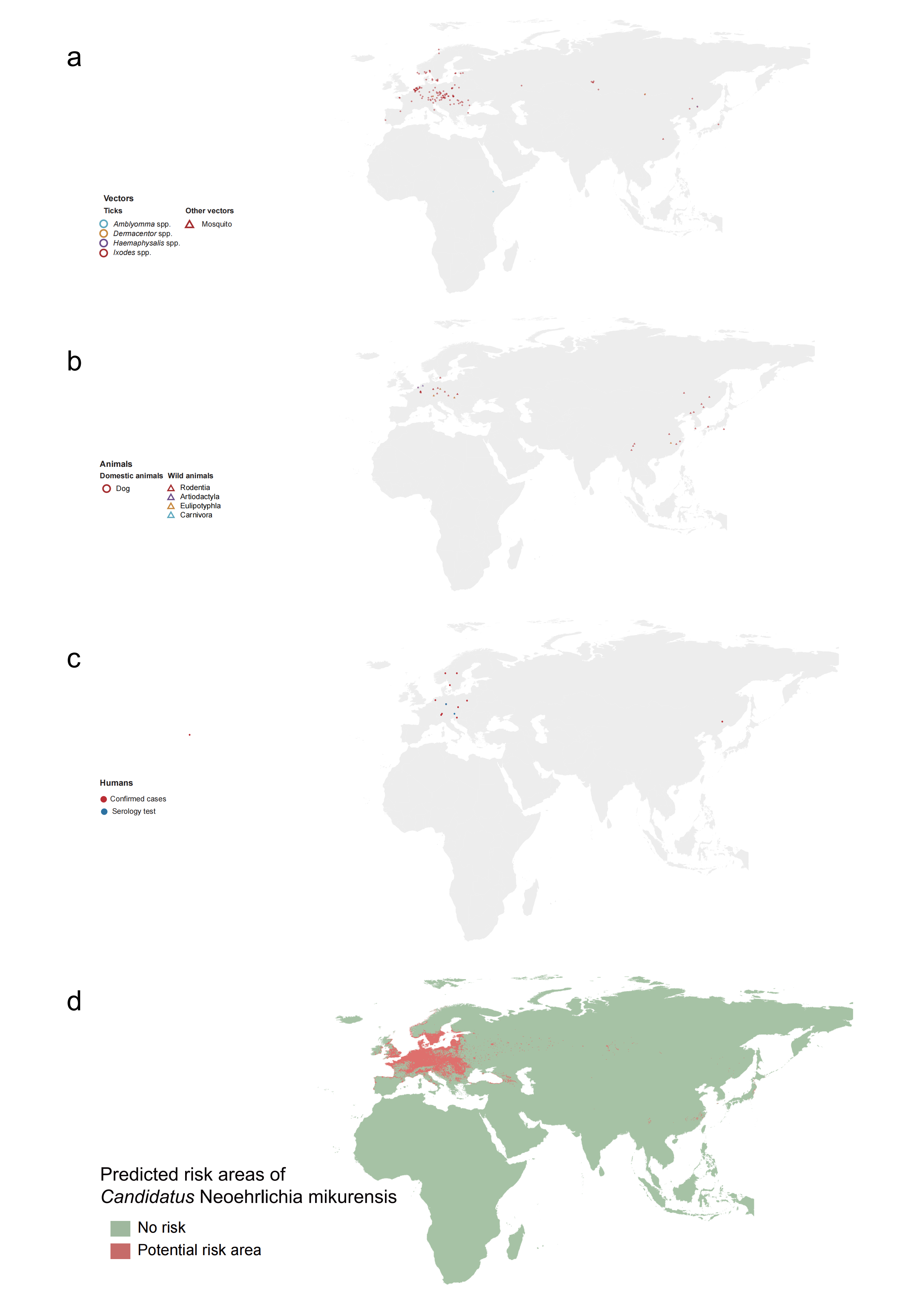


**Supplementary figure 31: The global recorded and predicted distributions of** ***E. canis*.**

(a-c) Recorded locations of *E. canis* which was detected from arthropod vectors, animal hosts and human beings. (d) Heat map of predicted relative risk distribution based on RF models about *E. canis*.


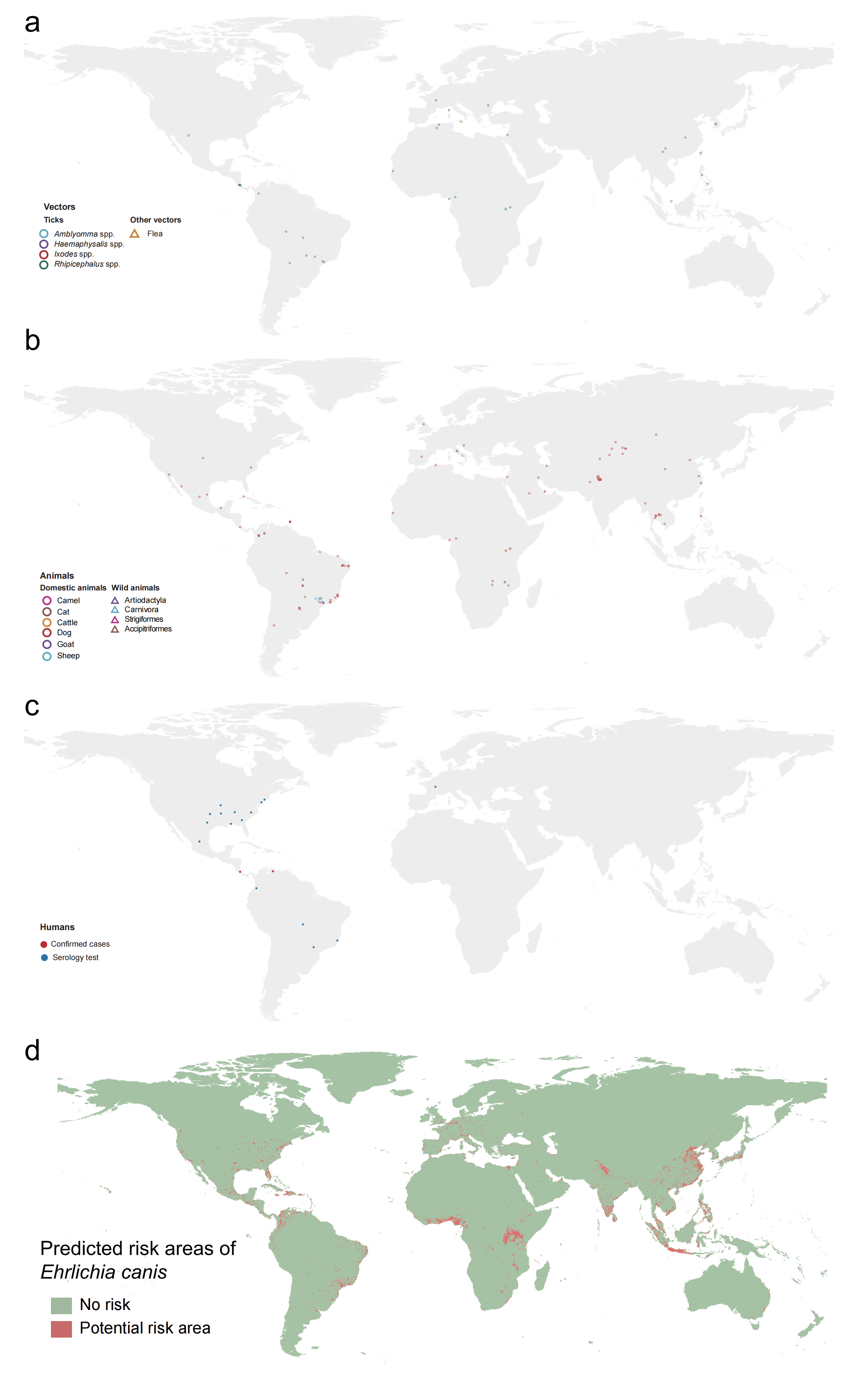


**Supplementary figure 32: The recorded and predicted distributions of *A. ovis* in Eurasia and Africa.**

(a-c) Recorded locations of *A. ovis* which was detected from arthropod vectors, animal hosts and human beings. (d) Heat map of predicted relative risk distribution based on RF models about *A. ovis*.


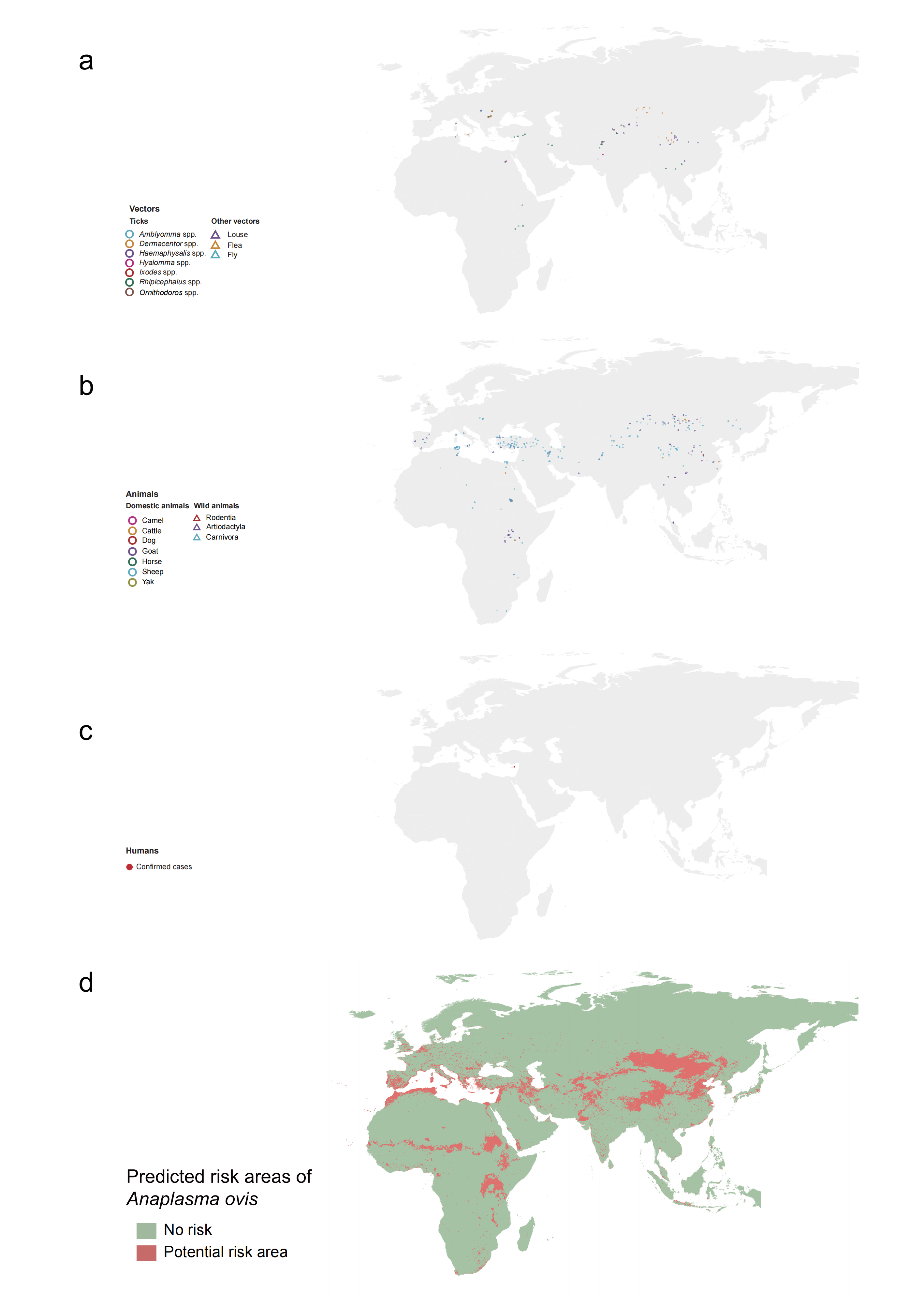


**Supplementary figure 33: The global recorded and predicted distributions of** ***A. platys*.**

(a-c) Recorded locations of *A. platys* which was detected from arthropod vectors, animal hosts and human beings. (d) Heat map of predicted relative risk distribution based on RF models about *A. platys*.


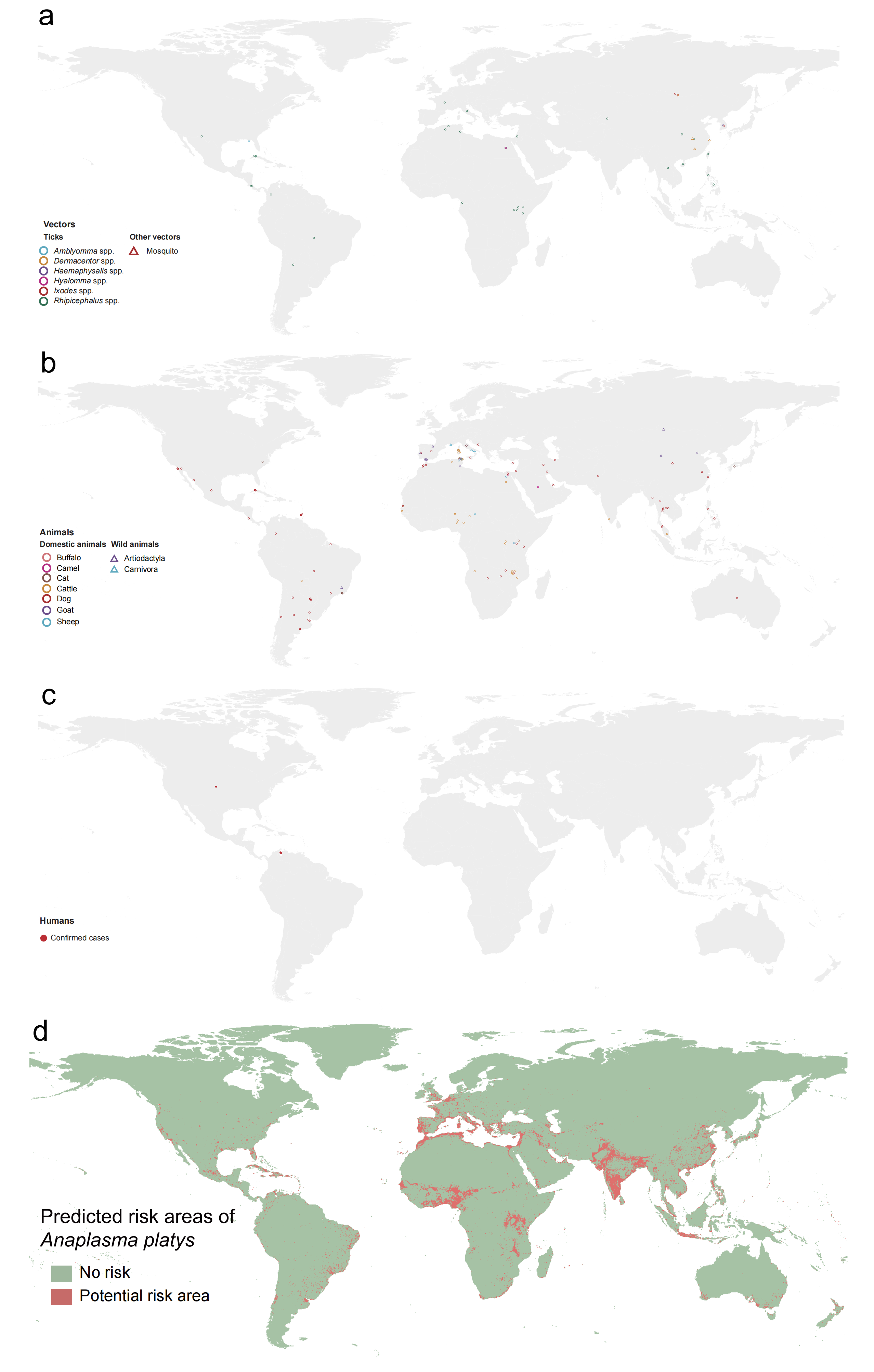


# **Supplementary figure 34: The global recorded and predicted distributions of *A. marginale*.**

(a-b) Recorded locations of *A. marginale* which was detected from arthropod vectors and animal hosts. (c) Heat map of predicted relative risk distribution based on RF models about *A. marginale*.


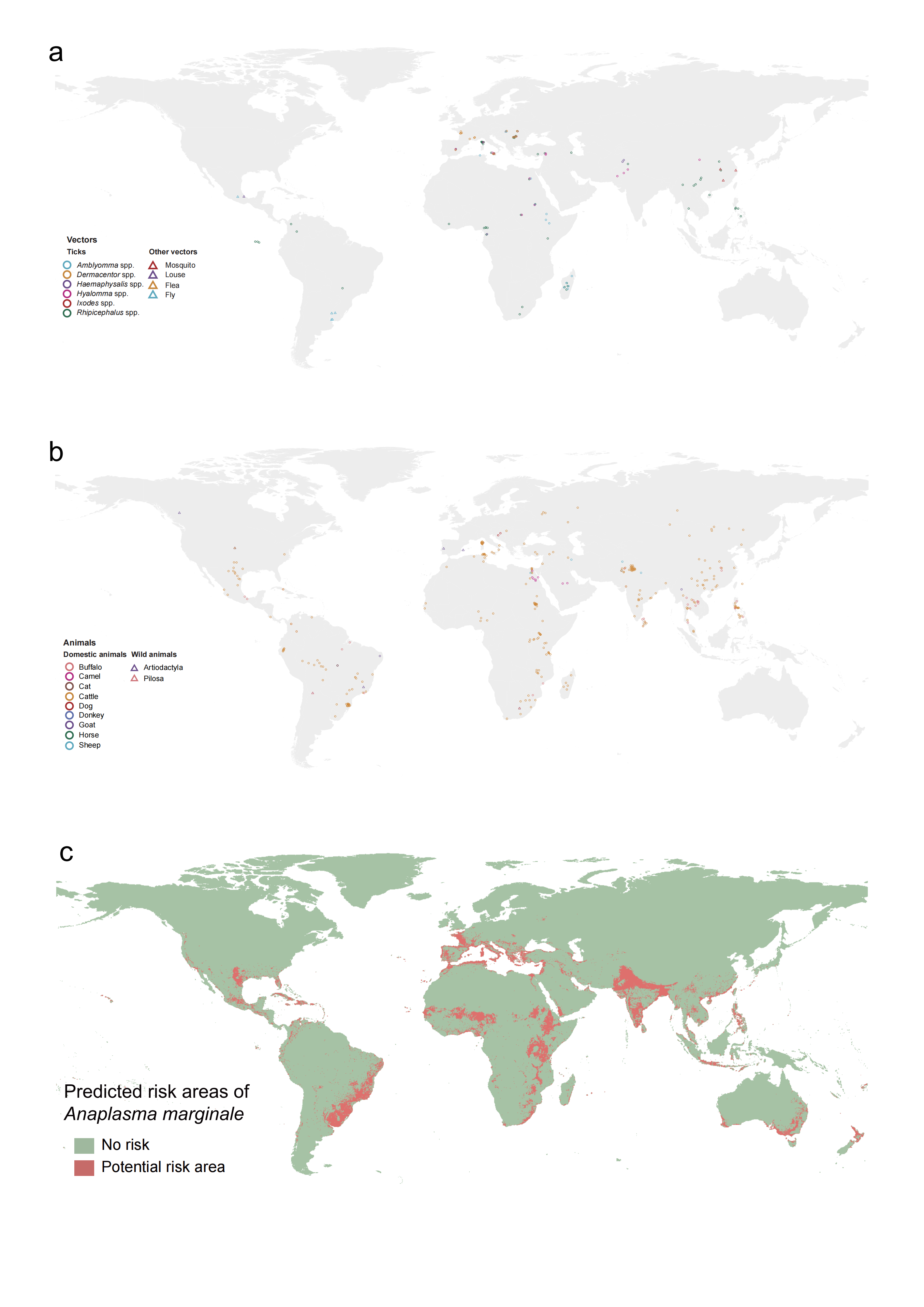


# **Supplementary figure 35: Publications searched from 1910 to 2022 on Anaplasmataceae species infection in animals, vectors, and humans after molecular validation.**

(a) Annual number of publications stratified by host type; (b) Number of newly identified Anaplasmataceae members per year; (c) Total number of publications on Anaplasmataceae species detected in vectors, animals, and humans; (d) Chord diagram illustrating the relationship between Anaplasmataceae species and host types.
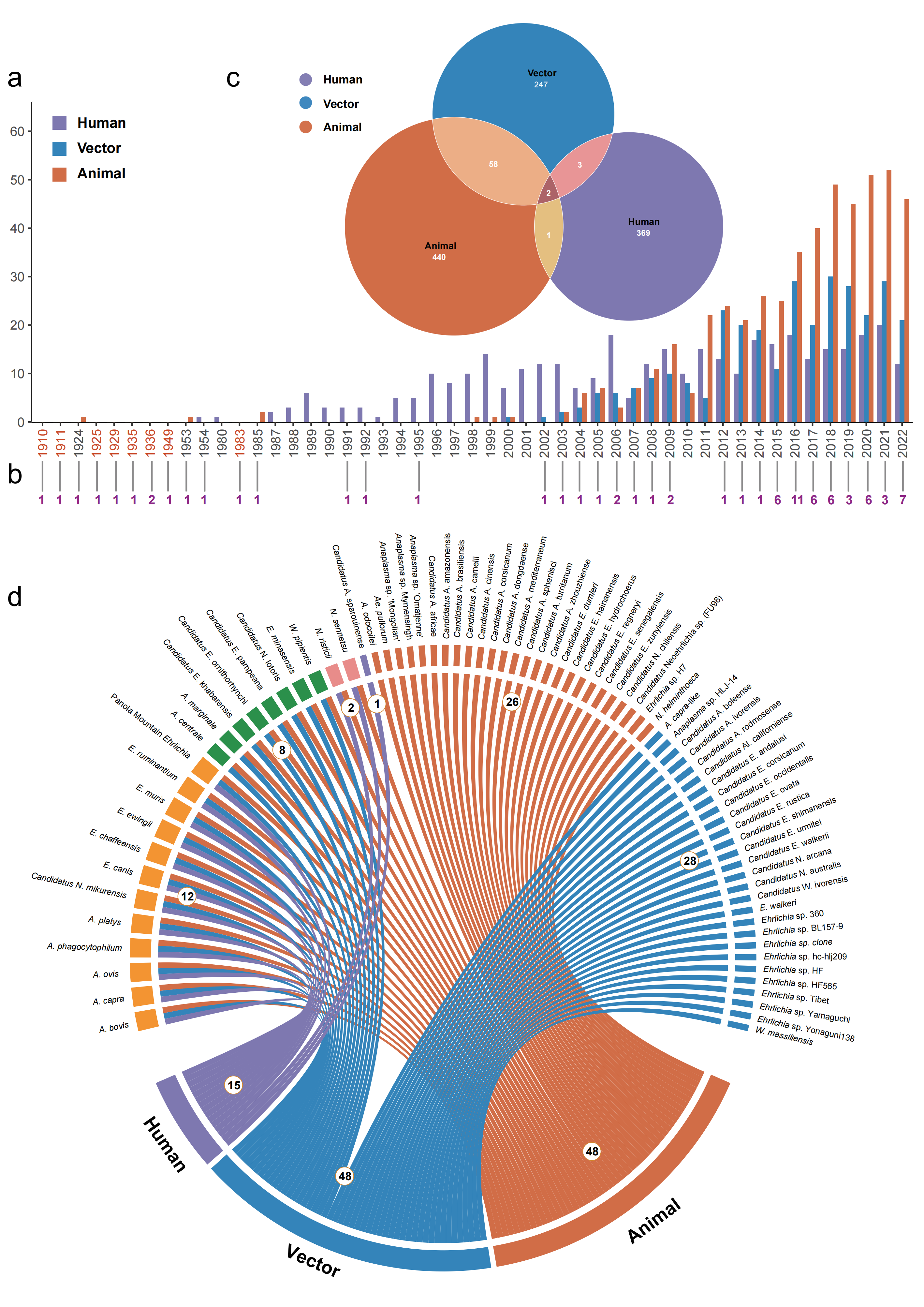


# **Supplementary figure 36: Vectors and numbers of animal species from which each Anaplasmataceae species was detected after molecular validation.**

Vectors include tick, fly, sandfly, mosquito, mite, louse, flea, bee, and bug. Ticks are further classified by genus. Animals include wildlife and livestock, with wild animals categorized by order. The total number of different host species harbouring Anaplasmataceae species in each host category is shown in the column header. The total number of Anaplasmataceae species detected in each host category is shown in the matrix.
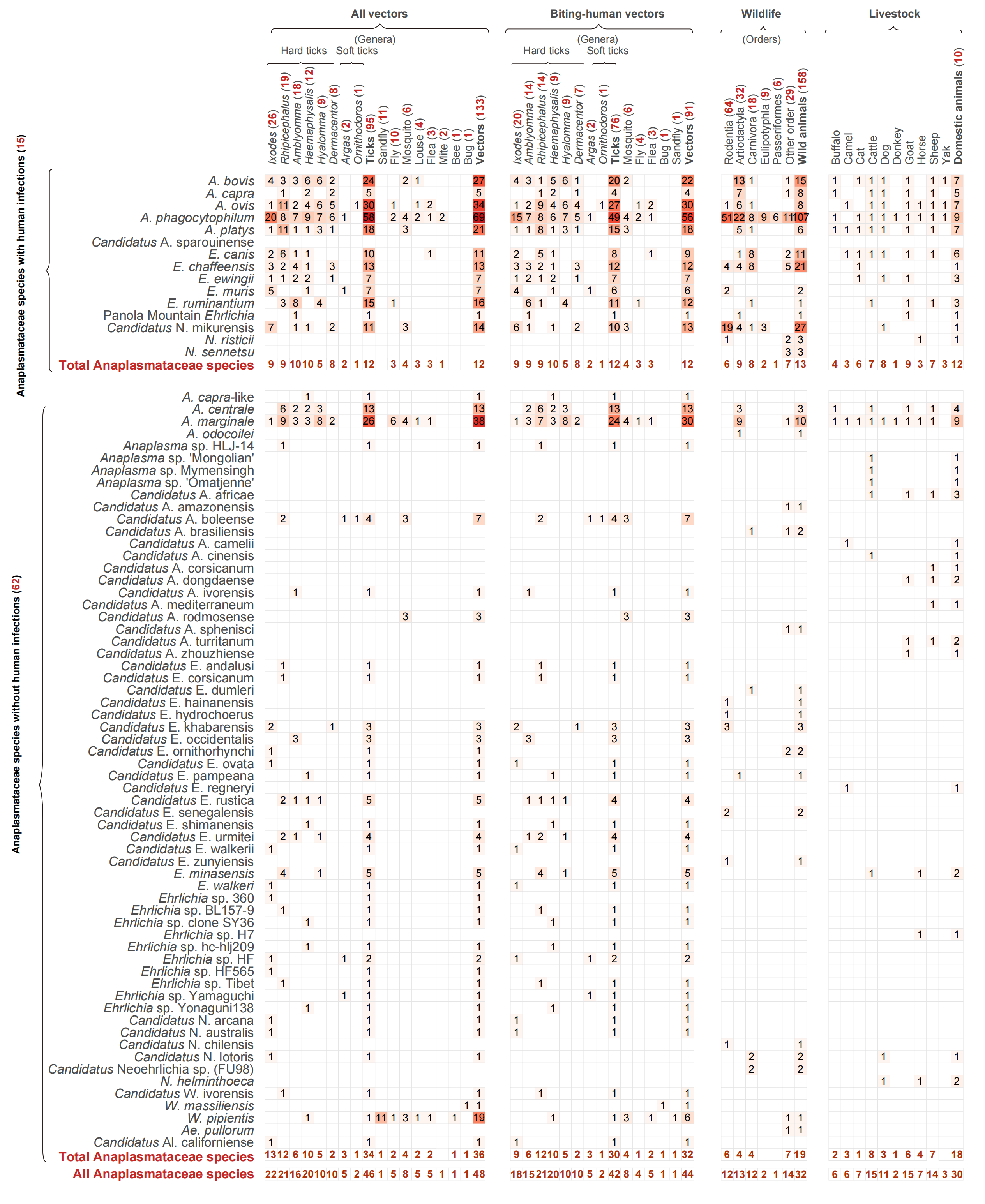


# **Supplementary figure 37: Global distribution of laboratory confirmed Anaplasmataceae species detection in vectors, animals, and humans from literature sources after molecular validation.**

(a) Vectors. (b) Animals. (c) Humans.

Figure a: Different shapes distinguish ticks from other vectors; Figure b: Different shapes distinguish domestic animals from wild animals; Figure c: Colors indicate different detection methods, shapes represent the type of location where they occur, and the size of the shapes indicates the number of people infected.
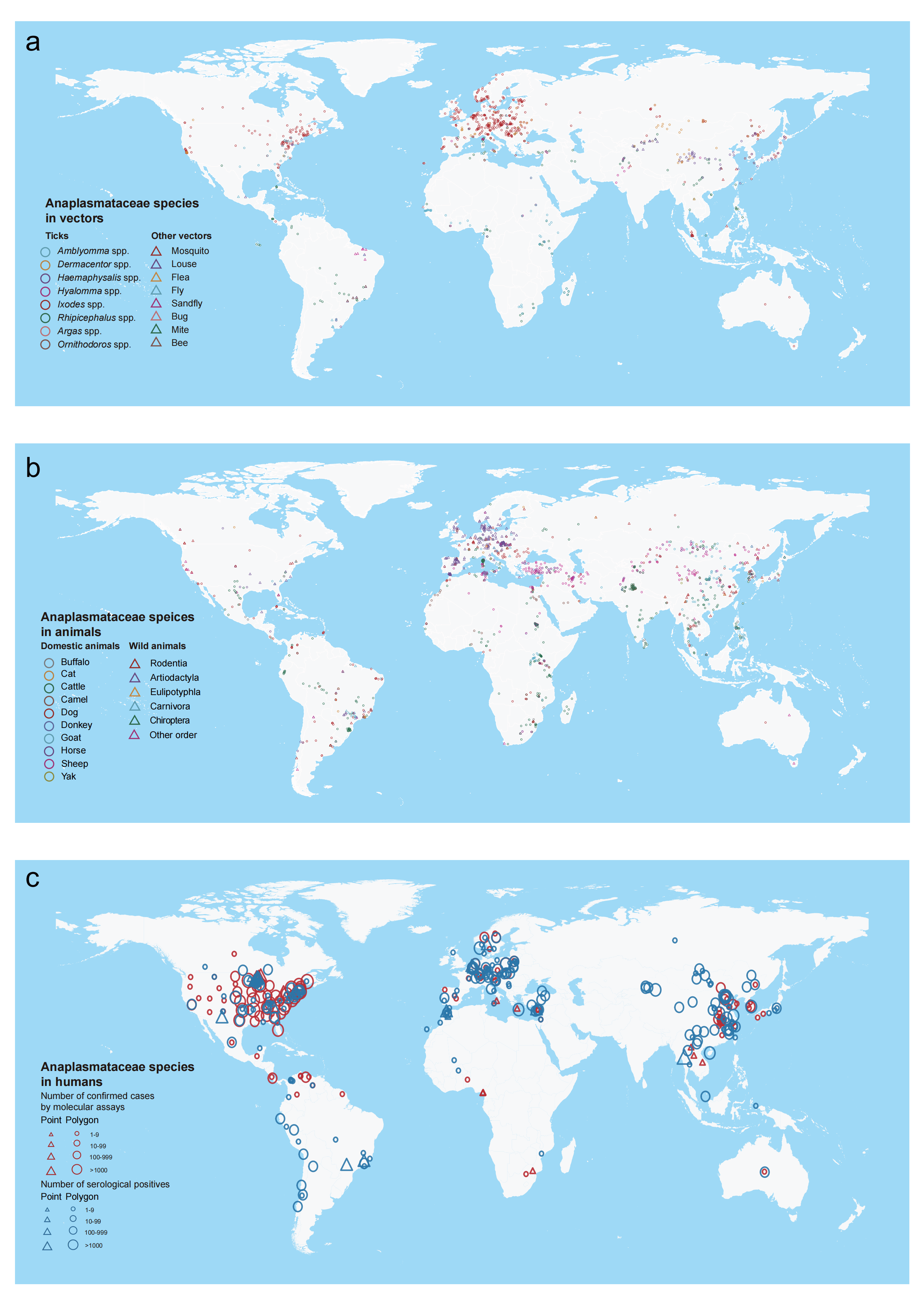


# **Supplementary figure 38: Spatial distribution of the major Anaplasmataceae species on a global scale after molecular validation.**

(a) The global distribution of major *Anaplasma* species. (b) The global distribution of major *Ehrlichia* and *Candidatus* Neoehrlichia species. (c) Number of detection locations in six continents for each predominant Anaplasmataceae species.


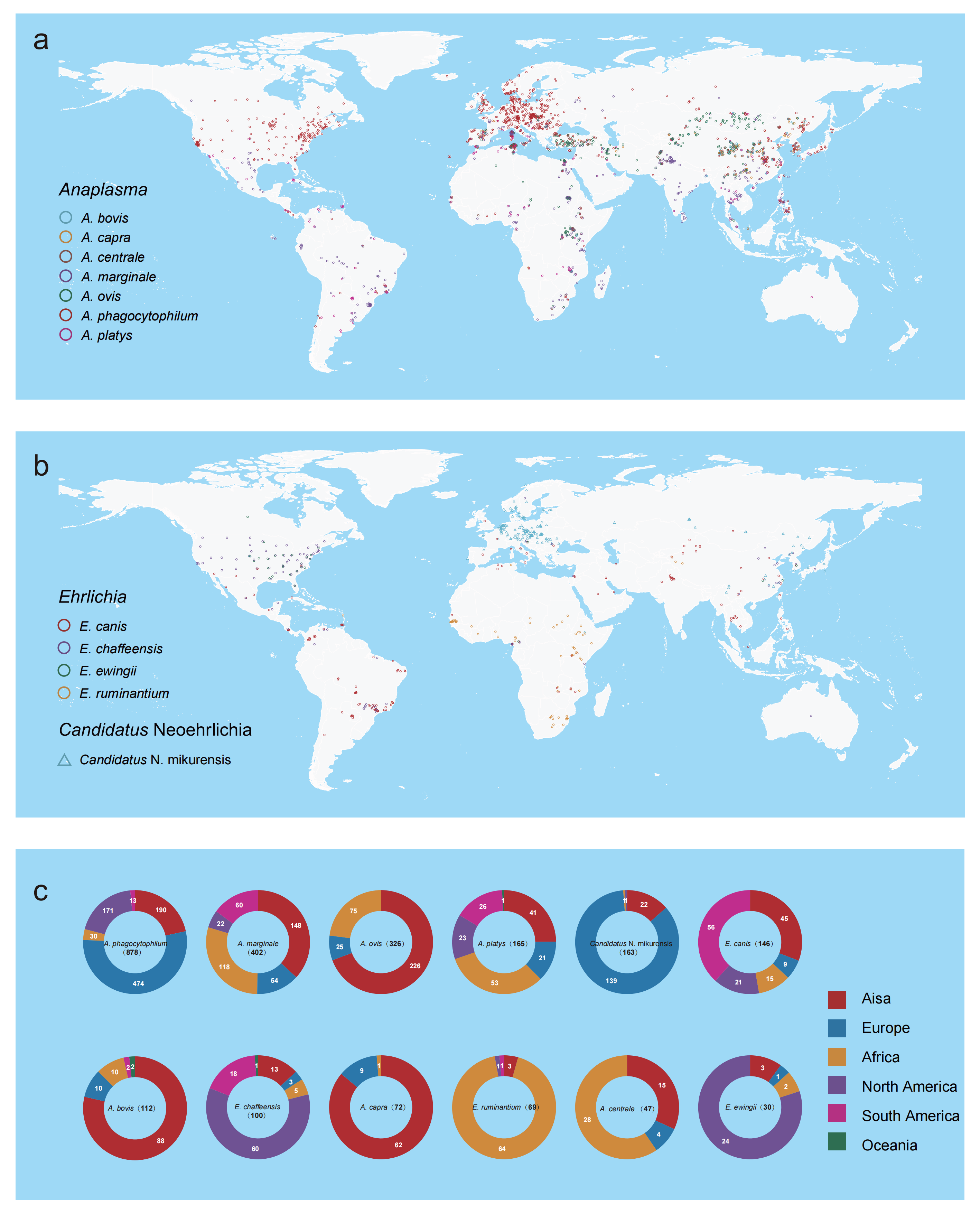


# **Supplementary figure 39: The relationship matrix of Anaplasmataceae species and involved ticks after molecular validation.**

The Anaplasmataceae species determined to infect humans are marked by red fonts. Names of ticks are marked in blue if they bite humans. The red square indicates pathogenic Anaplasmataceae species to humans carried by human-biting vectors, and it turns into purple if the vector was not found to bite humans. Blue squares indicate Anaplasmataceae species were not pathogenic to humans carried by human-biting vectors, and it turns into grey if carried by vectors of non-biting human.
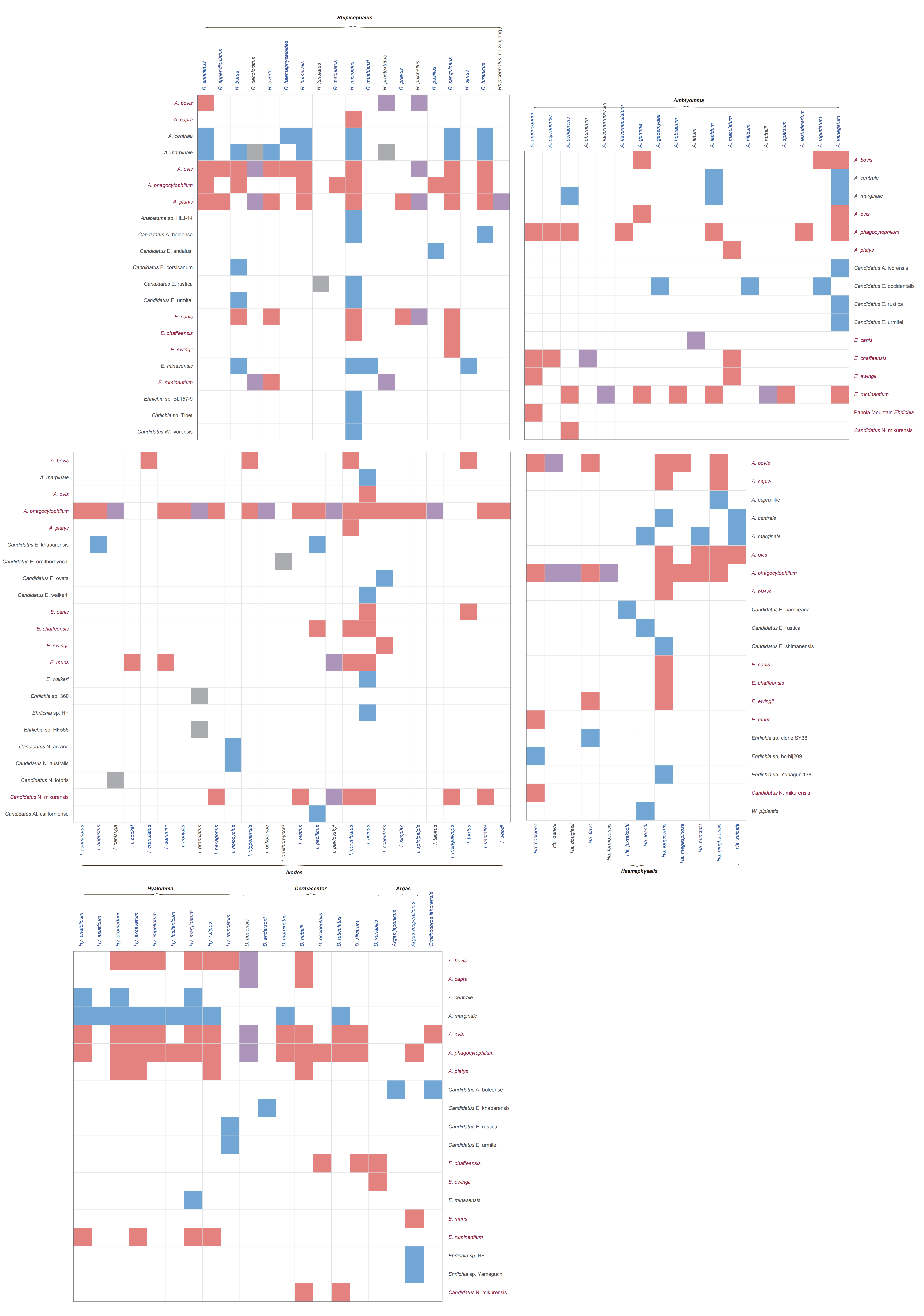


# **Supplementary figure 40: The relationship matrix of Anaplasmataceae species and other vectors after molecular validation.**

The Anaplasmataceae species determined to infect humans are marked by red fonts. Names of ticks are marked in blue if they bite humans. The red square indicates pathogenic Anaplasmataceae species to humans carried by human-biting vectors, and it turns into purple if the vector was not found to bite humans. Blue squares indicate Anaplasmataceae species were not pathogenic to humans carried by human-biting vectors, and it turns into grey if carried by vectors of non-biting human.
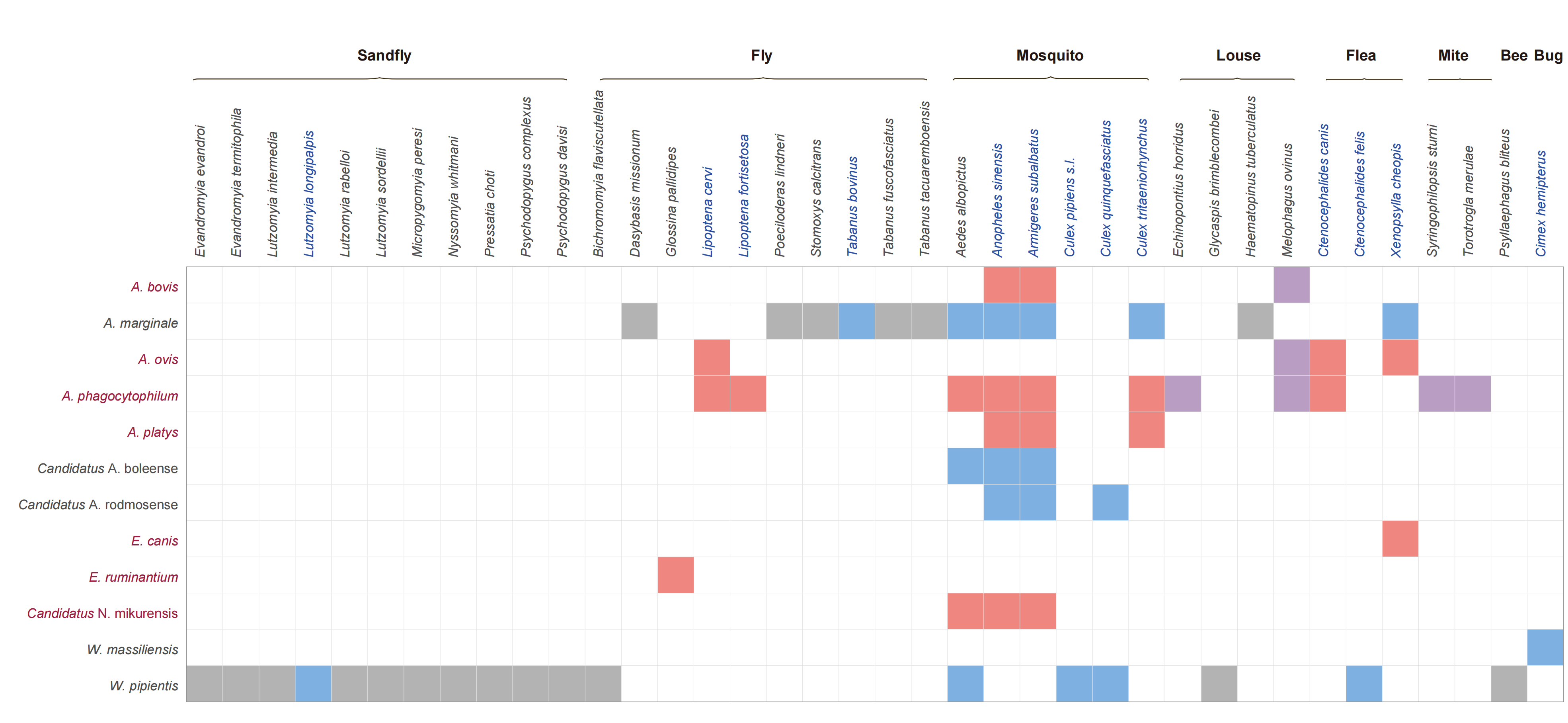


# **Supplementary figure 41: The relationship matrix of Anaplasmataceae species and animals after molecular validation.**


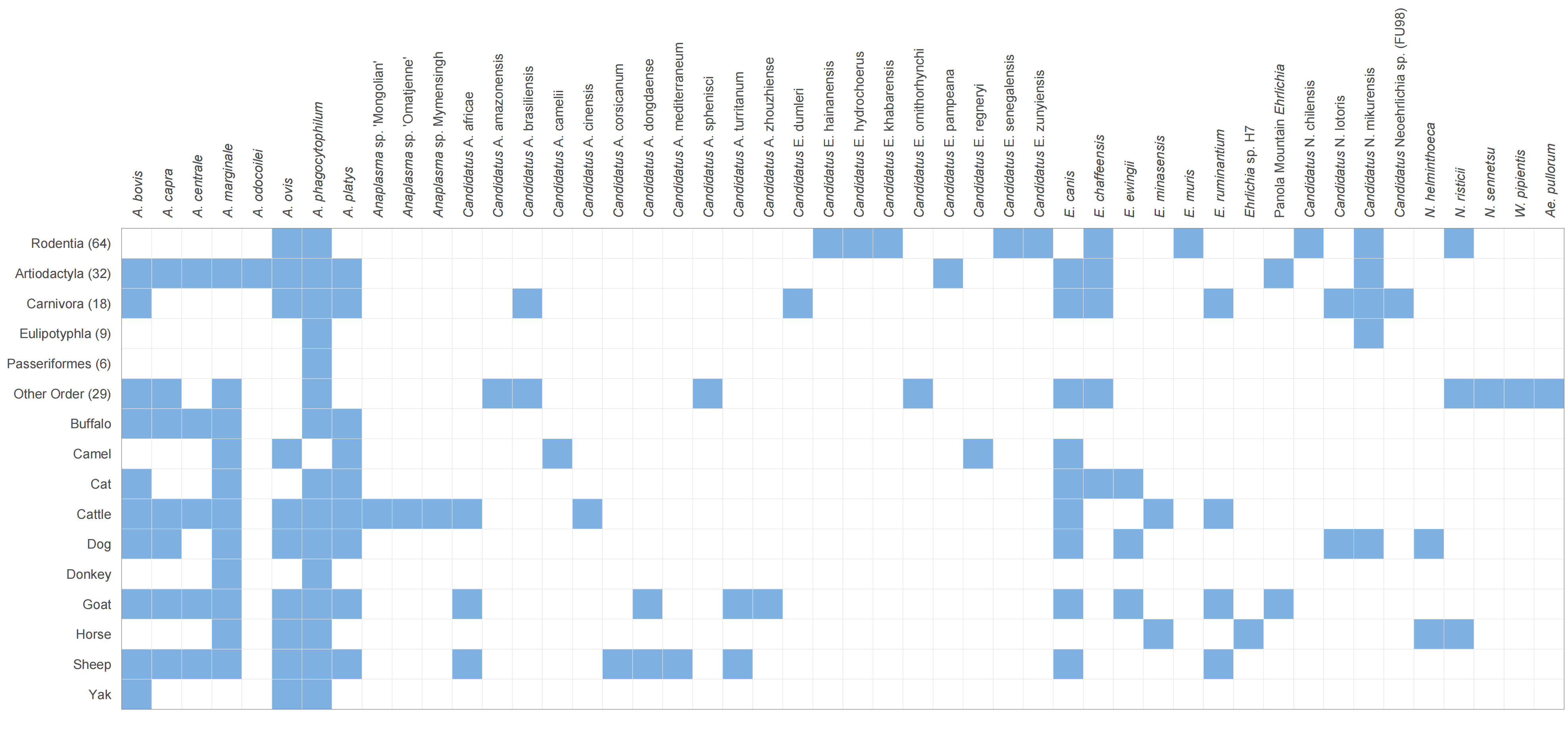


# **Supplementary figure 42: The global distribution of the other 65 Anaplasmataceae species after molecular validation.**

(a) *Anaplasma* spp.; (b) *Ehrlichia* spp.; (c) Other genera.


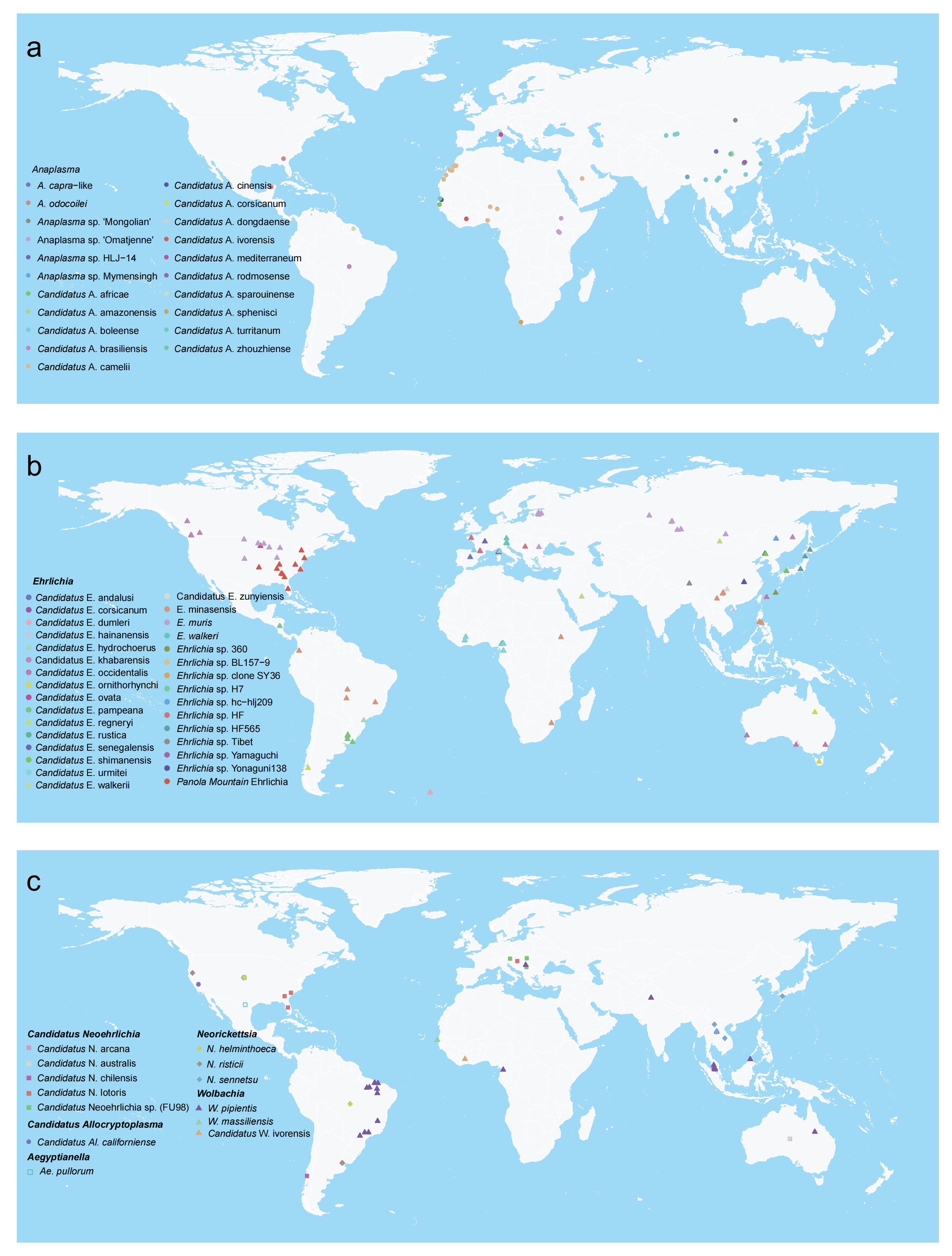

Supplement: Appendix 1 [file mmc1.docx]
