# Supplementary figures and images for: The global distribution and risk prediction of Anaplasmataceae species: a systematic review and geospatial modelling analysis
Source: eBioMedicine. 2025 Apr 23;115:105722. doi: 10.1016/j.ebiom.2025.105722 (PMC12051633; doi:10.1016/j.ebiom.2025.105722)

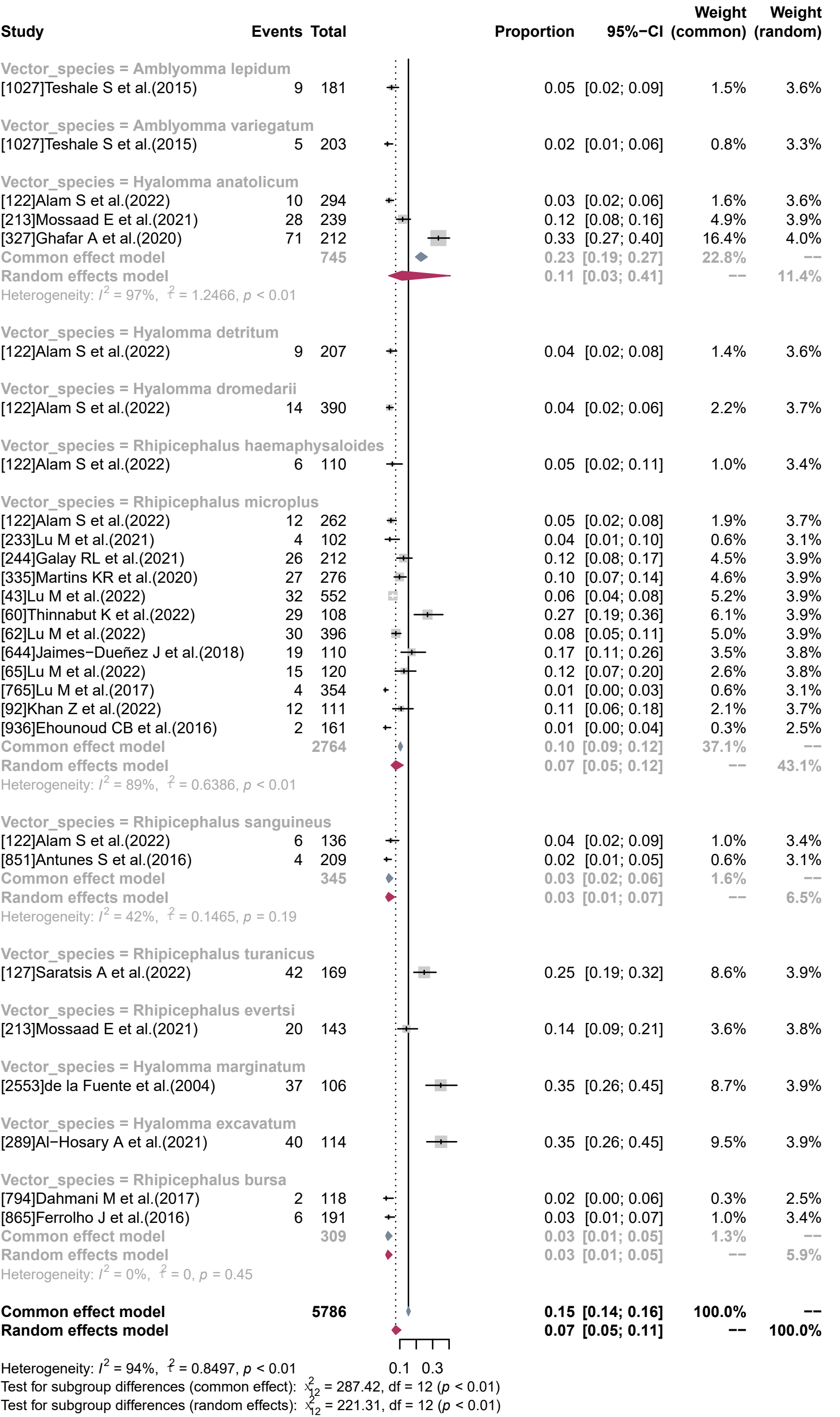

Supplement: Appendix 4_A. marginale_forest plot [file mmc4.pdf]

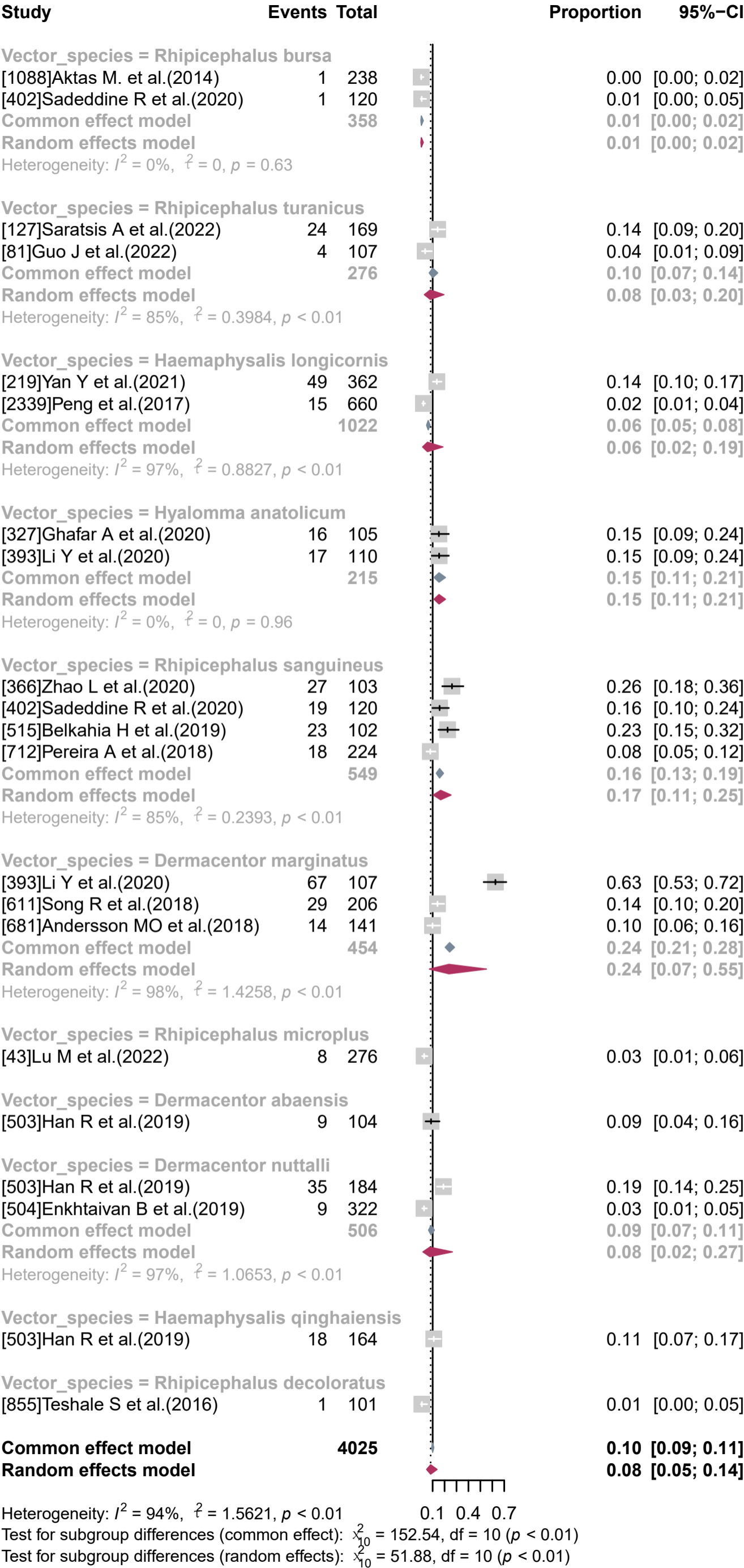

Supplement: Appendix4_A. ovis_forest plot [file mmc5.pdf]

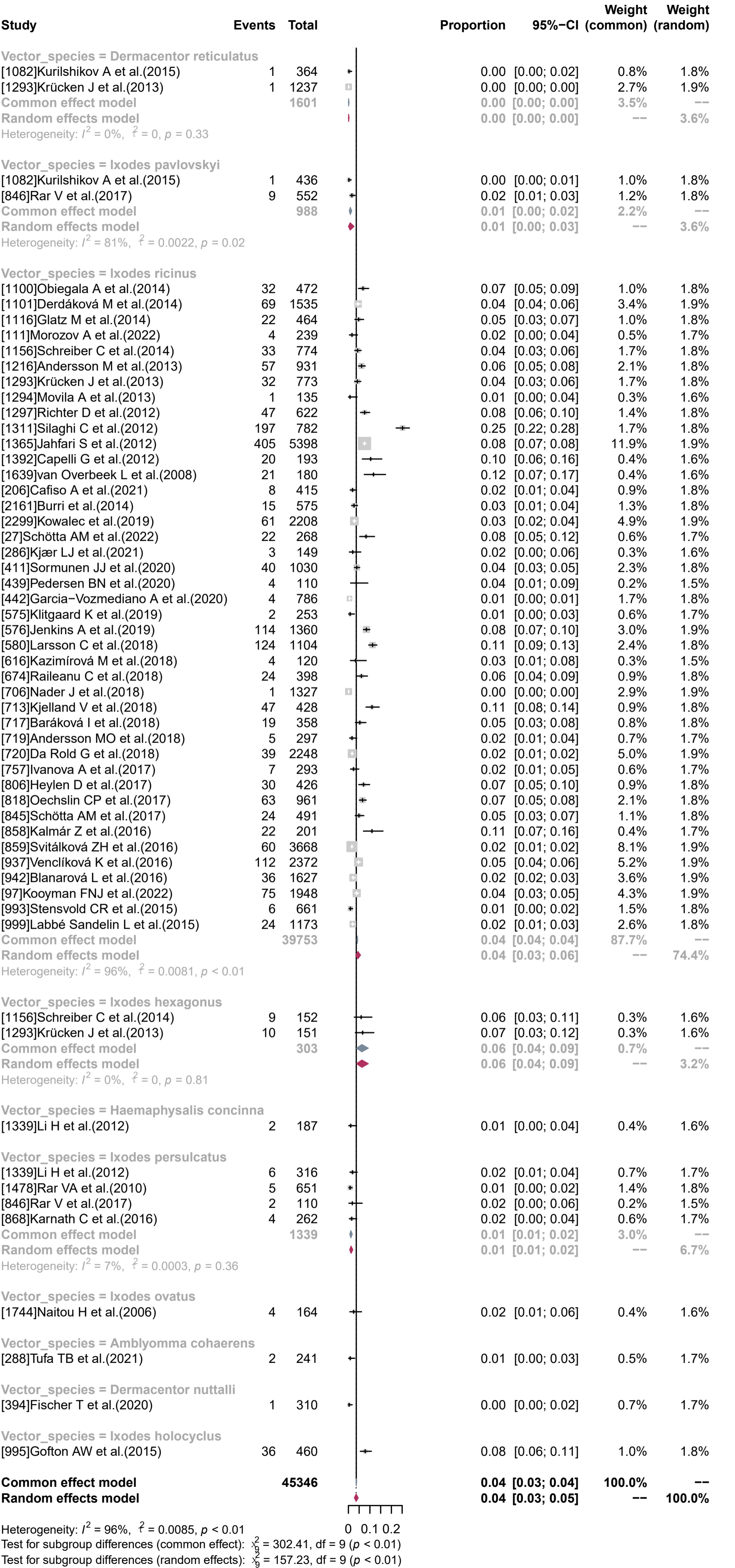

Supplement: Appendix4_Candidatus N. mikurensis_forest plot [file mmc7.pdf]
