# Supplementary material for: The global distribution and risk prediction of Anaplasmataceae species: a systematic review and geospatial modelling analysis
Source: eBioMedicine. 2025 Apr 23;115:105722. doi: 10.1016/j.ebiom.2025.105722 (PMC12051633; doi:10.1016/j.ebiom.2025.105722)
Supplement: Appendix4_A. phagocytophilum_forest plot [file mmc6.pdf]

Study	Events	Total	Proportion	95%−CI
**Vector_species = Ixodes ricinus**				
[1008]Stanczak J et al.(2015)	17	541	0.03	[0.02; 0.05]
[1049]Ebani VV et al.(2015)	66	252	0.26	[0.21; 0.32]
[1090]Víchová B et al.(2014)	29	1075	0.03	[0.02; 0.04]
[1098]Eshoo MW et al.(2014)	5	226	0.02	[0.01; 0.05]
[1100]Obiegala A et al.(2014)	16	918	0.02	[0.01; 0.03]
[1101]Derdáková M et al.(2014)	53	1404	0.04	[0.03; 0.05]
[110]Gandy S et al.(2022)	122	2128	0.06	[0.05; 0.07]
[1116]Glatz M et al.(2014)	5	464	0.01	[0.00; 0.02]
[111]Morozov A et al.(2022)	18	246	0.07	[0.04; 0.11]
[113]Grochowska A et al.(2022)	10	784	0.01	[0.01; 0.02]
[1154]Hornok S et al.(2014)	21	240	0.09	[0.05; 0.13]
[1156]Schreiber C et al.(2014)	50	774	0.06	[0.05; 0.08]
[1159]Tveten AK. et al.(2014)	56	1804	0.03	[0.02; 0.04]
[1169]Cisak E et al.(2014)	23	861	0.03	[0.02; 0.04]
[1176]Venclikova K et al.(2014)	46	1473	0.03	[0.02; 0.04]
[1194]Kiewra D et al.(2014)	92	1290	0.07	[0.06; 0.09]
[1203]Chai HN et al.(2013)	44	5343	0.01	[0.01; 0.01]
[1206]Tappe J et al.(2013)	94	572	0.16	[0.13; 0.20]
[1207]Overzier E et al.(2013)	214	3804	0.06	[0.05; 0.06]
[1211]Soleng A et al.(2013)	6	220	0.03	[0.01; 0.06]
[1221]Overzier E et al.(2013)	293	530	0.55	[0.51; 0.60]
[1237]Pangrácová L et al.(2013)	18	670	0.03	[0.02; 0.04]
[1247]Movila A et al.(2013)	3	126	0.02	[0.00; 0.07]
[124]Raileanu C et al.(2022)	10	680	0.01	[0.01; 0.03]
[1254]Dumitrache MO et al.(2013)	115	959	0.12	[0.10; 0.14]
[126]Flattery A et al.(2022)	62	1376	0.05	[0.03; 0.06]
[1282]Asman M et al.(2013)	4	788	0.01	[0.00; 0.01]
[1284]Geller J et al.(2013)	2	496	0.00	[0.00; 0.01]
[1285]Claerebout E et al.(2013)	127	679	0.19	[0.16; 0.22]
[1292]Richter et al.(2013)	24	384	0.06	[0.04; 0.09]
[1293]Krücken J et al.(2013)	49	773	0.06	[0.05; 0.08]
[1294]Movila A et al.(2013)	2	135	0.01	[0.00; 0.05]
[1295]Volgina NS et al.(2013)	22	358	0.06	[0.04; 0.09]
[1296]Reye AL et al.(2013)	12	327	0.04	[0.02; 0.06]
[1297]Richter D et al.(2012)	30	683	0.04	[0.03; 0.06]
[1301]Aktas M et al.(2012)	8	210	0.04	[0.02; 0.07]
[1306]Aureli S et al.(2012)	29	258	0.11	[0.08; 0.16]
[1328]Maioli G et al.(2012)	4	128	0.03	[0.01; 0.08]
[1340]Lommano E et al.(2012)	22	812	0.03	[0.02; 0.04]
[1341]Lempereur L et al.(2012)	47	653	0.07	[0.05; 0.09]
[1347]Paulauskas A et al.(2012)	14	488	0.03	[0.02; 0.05]
[1351]Sytykiewicz H et al.(2012)	131	1448	0.09	[0.08; 0.11]
[1367]Wallménius K et al.(2012)	6	260	0.02	[0.01; 0.05]
[1370]Palomar AM et al.(2012)	1	181	0.01	[0.00; 0.03]
[1393]Katargina O et al.(2012)	70	3088	0.02	[0.02; 0.03]
[1400]Portillo A et al.(2011)	30	257	0.12	[0.08; 0.16]
[1408]Hildebrandt A et al.(2011)	54	2000	0.03	[0.02; 0.04]
[1432]Sen E et al.(2011)	48	482	0.10	[0.07; 0.13]
[1449]Schicht S et al.(2011)	52	1646	0.03	[0.02; 0.04]
[1451]Schorn S et al.(2011)	500	5485	0.09	[0.08; 0.10]
[1455]Franke J et al.(2011)	3	119	0.03	[0.01; 0.07]
[1457]Reis C et al.(2011)	2	126	0.02	[0.00; 0.06]
[1489]Franke J et al.(2010)	11	447	0.02	[0.01; 0.04]
[1498]Hildebrandt A et al.(2010)	54	1000	0.05	[0.04; 0.07]
[1500]Halos L et al.(2010)	15	225	0.07	[0.04; 0.11]
[1517]Tomanovic S et al.(2010)	40	287	0.14	[0.10; 0.18]
[1524]Cotté V et al.(2010)	2	222	0.01	[0.00; 0.03]
[1540]Santos AS et al.(2009)	6	142	0.04	[0.02; 0.09]
[1543]Paulauskas A et al.(2009)	196	597	0.33	[0.29; 0.37]
[1555]Wójcik−Fatla A et al.(2009)	160	3240	0.05	[0.04; 0.06]
[1557]Bown KJ et al.(2009)	47	4840	0.01	[0.01; 0.01]
[1559]Kybicová K et al.(2009)	10	118	0.08	[0.04; 0.15]
[155]Luu L et al.(2021)	7	436	0.02	[0.01; 0.03]
[1562]Movila A et al.(2009)	11	240	0.05	[0.02; 0.08]
[1584]Michalik J et al.(2009)	60	301	0.20	[0.16; 0.25]
[1596]Rosef O et al.(2009)	116	852	0.14	[0.11; 0.16]
[15]Žákovská A et al.(2022)	14	563	0.02	[0.01; 0.04]
[1611]Rosef O et al.(2009)	90	932	0.10	[0.08; 0.12]
[1626]Silaghi C et al.(2008)	103	2494	0.04	[0.03; 0.05]
[1629]Milutinovic M et al.(2008)	40	255	0.16	[0.11; 0.21]
[1636]de Carvalho IL et al.(2008)	1	300	0.00	[0.00; 0.02]
[1654]Zygner W et al.(2008)	6	209	0.03	[0.01; 0.06]
[1657]Barandika JF et al.(2008)	16	288	0.06	[0.03; 0.09]
[1676]Skarphédinsson S et al.(2007)	25	106	0.24	[0.16; 0.33]
[1677]Matsumoto K et al.(2007)	18	1706	0.01	[0.01; 0.02]
[1684]Koci J et al.(2007)	231	1126	0.21	[0.18; 0.23]
[1716]Piccolin G et al.(2006)	85	1931	0.04	[0.04; 0.05]
[1719]Grzeszczuk A. et al.(2006)	108	737	0.15	[0.12; 0.17]
[1725]Smetanová K et al.(2006)	12	271	0.04	[0.02; 0.08]
[1734]Grzeszczuk A et al.(2006)	207	1130	0.18	[0.16; 0.21]
[1749]Pichon B et al.(2006)	1	127	0.01	[0.00; 0.04]
[174]Kirczuk L et al.(2021)	60	1401	0.04	[0.03; 0.05]
[1751]Halos L et al.(2006)	79	521	0.15	[0.12; 0.19]
[1754]Mantelli B et al.(2006)	100	921	0.11	[0.09; 0.13]
[1755]Ferquel E et al.(2006)	6	1236	0.00	[0.00; 0.01]
[1770]Bown KJ et al.(2006)	3	158	0.02	[0.00; 0.05]
[1786]Sarih M et al.(2005)	2	197	0.01	[0.00; 0.04]
[1809]Cisak E et al.(2005)	7	109	0.06	[0.03; 0.13]
[1830]Sréter T et al.(2004)	6	452	0.01	[0.00; 0.03]
[1837]Santos AS et al.(2004)	7	154	0.05	[0.02; 0.09]
[1848]Grzeszczuk A et al.(2004)	49	559	0.09	[0.07; 0.11]
[1850]Stanczak J et al.(2004)	98	697	0.14	[0.12; 0.17]
[1854]Polin H et al.(2004)	128	1760	0.07	[0.06; 0.09]
[1856]Hartelt K et al.(2004)	54	4759	0.01	[0.01; 0.01]
[1887]Christova I et al.(2003)	122	412	0.30	[0.25; 0.34]
[1890]Sixl W et al.(2003)	12	235	0.05	[0.03; 0.09]
[1899]Santino I et al.(2003)	11	141	0.08	[0.04; 0.14]
[1900]Hildebrandt A et al.(2003)	6	140	0.04	[0.02; 0.09]
[1913]Santino I et al.(2002)	40	1475	0.03	[0.02; 0.04]
[1926]Hildebrandt A et al.(2002)	7	305	0.02	[0.01; 0.05]
[1936]Alekseev AN et al.(2001)	1	295	0.00	[0.00; 0.02]
[1943]Bjöersdorff A et al.(2001)	9	112	0.08	[0.04; 0.15]
[1950]Christova I et al.(2001)	40	202	0.20	[0.15; 0.26]
[1956]Walker AR et al.(2001)	44	1476	0.03	[0.02; 0.04]
[1995]Fingerle V et al.(1999)	8	492	0.02	[0.01; 0.03]
[1997]Schouls LM et al.(1999)	12	121	0.10	[0.05; 0.17]
[2004]Pusterla N et al.(1999)	21	1667	0.01	[0.01; 0.02]
[2008]Baumgarten BU et al.(1999)	14	287	0.05	[0.03; 0.08]
[2022]Pusterla N et al.(1998)	58	1163	0.05	[0.04; 0.06]
[208]Václavík T et al.(2021)	467	13340	0.04	[0.03; 0.04]
[2167]Fedoniuk LY et al.(2019)	82	278	0.29	[0.24; 0.35]
[218]Remesar S et al.(2021)	7	1121	0.01	[0.00; 0.01]
[2193]Henningsson AJ et al.(2015)	31	2253	0.01	[0.01; 0.02]
[2240]Spitalska et al.(2021)	91	616	0.15	[0.12; 0.18]
[2299]Kowalec et al.(2019)	58	1586	0.04	[0.03; 0.05]
[2318]Asman et al.(2018)	11	459	0.02	[0.01; 0.04]
[2340]Rybarova et al.(2017)	53	598	0.09	[0.07; 0.11]
[2349]Butler et al.(2016)	2	130	0.02	[0.00; 0.05]
[2374]Marcutan et al.(2015)	8	459	0.02	[0.01; 0.03]
[2376]Henningsson et al.(2015)	22	749	0.03	[0.02; 0.04]
[2412]Dziegiel et al.(2014)	22	214	0.10	[0.07; 0.15]
[2442]Christova et al.(2012)	47	129	0.36	[0.28; 0.45]
[2494]Meier et al.(2009)	5	151	0.03	[0.01; 0.08]
[2497]Hildebrandt et al.(2009)	54	1000	0.05	[0.04; 0.07]
[2505]Skotarczak et al.(2008)	47	324	0.15	[0.11; 0.19]
[2510]Radzijevskaja et al.(2008)	14	206	0.07	[0.04; 0.11]
[2533]Grzeszczuk et al.(2006)	54	372	0.15	[0.11; 0.19]
[256]Olsthoorn F et al.(2021)	132	2828	0.05	[0.04; 0.06]
[258]Keyte S et al.(2021)	131	2682	0.05	[0.04; 0.06]
[260]Kovryha N et al.(2021)	23	155	0.15	[0.10; 0.21]
[27]Schötta AM et al.(2022)	4	150	0.03	[0.01; 0.07]
[286]Kjær LJ et al.(2021)	17	149	0.11	[0.07; 0.18]
[291]Michalski MM et al.(2021)	56	240	0.23	[0.18; 0.29]
[297]Audino T et al.(2021)	6	443	0.01	[0.00; 0.03]
[370]Capligina V et al.(2020)	46	3820	0.01	[0.01; 0.02]
[386]Michalski MM et al.(2020)	5	423	0.01	[0.00; 0.03]
[395]Kocon A et al.(2020)	4	119	0.03	[0.01; 0.08]
[411]Sormunen JJ et al.(2020)	16	1030	0.02	[0.01; 0.03]
[442]Garcia−Vozmediano A et al.(2020)	15	786	0.02	[0.01; 0.03]
[449]Lejal E et al.(2019)	53	999	0.05	[0.04; 0.07]
[44]Rataud A et al.(2022)	60	1039	0.06	[0.04; 0.07]
[463]Akl T et al.(2019)	16	214	0.07	[0.04; 0.12]
[487]Stigum VM et al.(2019)	555	9241	0.06	[0.06; 0.07]
[532]Asman M et al.(2019)	6	198	0.03	[0.01; 0.06]
[535]Hamšíková Z et al.(2019)	656	10535	0.06	[0.06; 0.07]
[561]Vaculová T et al.(2019)	52	932	0.06	[0.04; 0.07]
[567]Namina A et al.(2019)	35	581	0.06	[0.04; 0.08]
[575]Klitgaard K et al.(2019)	103	1013	0.10	[0.08; 0.12]
[594]Duplan F et al.(2018)	4	540	0.01	[0.00; 0.02]
[600]Kocíková B et al.(2018)	11	410	0.03	[0.01; 0.05]
[616]Kazimírová M et al.(2018)	248	343	0.72	[0.67; 0.77]
[674]Raileanu C et al.(2018)	6	165	0.04	[0.01; 0.08]
[698]Chvostác M et al.(2018)	26	543	0.05	[0.03; 0.07]
[708]Mysterud A et al.(2018)	415	4508	0.09	[0.08; 0.10]
[713]Kjelland V et al.(2018)	6	158	0.04	[0.01; 0.08]
[717]Baráková I et al.(2018)	14	257	0.05	[0.03; 0.09]
[719]Andersson MO et al.(2018)	4	297	0.01	[0.00; 0.03]
[720]Da Rold G et al.(2018)	74	2248	0.03	[0.03; 0.04]
[726]Blazejak K et al.(2017)	87	1486	0.06	[0.05; 0.07]
[732]Asman M et al.(2017)	2	140	0.01	[0.00; 0.05]
[738]Honig V et al.(2017)	9	342	0.03	[0.01; 0.05]
[762]Didyk YM et al.(2017)	36	458	0.08	[0.06; 0.11]
[770]Chastagner A et al.(2017)	22	1837	0.01	[0.01; 0.02]
[797]Morganti G et al.(2017)	20	212	0.09	[0.06; 0.14]
[806]Heylen D et al.(2017)	31	440	0.07	[0.05; 0.10]
[818]Oechslin CP et al.(2017)	14	882	0.02	[0.01; 0.03]
[837]Matei IA et al.(2017)	29	506	0.06	[0.04; 0.08]
[845]Schötta AM et al.(2017)	4	183	0.02	[0.01; 0.06]
[852]Sormunen JJ et al.(2016)	38	989	0.04	[0.03; 0.05]
[858]Kalmár Z et al.(2016)	16	199	0.08	[0.05; 0.13]
[87]Del Cerro A et al.(2022)	16	356	0.04	[0.03; 0.07]
[919]Skotarczak B et al.(2016)	5	262	0.02	[0.01; 0.04]
[937]Venclíková K et al.(2016)	108	2266	0.05	[0.04; 0.06]
[949]Klubal R et al.(2016)	3	142	0.02	[0.00; 0.06]
[96]Ebani VV et al.(2022)	66	162	0.41	[0.33; 0.49]
[97]Kooyman FNJ et al.(2022)	26	1922	0.01	[0.01; 0.02]
[984]Aureli S et al.(2015)	23	210	0.11	[0.07; 0.16]
[987]Matei IA et al.(2015)	359	10483	0.03	[0.03; 0.04]
[989]Svitálková Z et al.(2015)	287	4374	0.07	[0.06; 0.07]
Common effect model		196475	0.05	[0.05; 0.05]
Random effects model			0.05	[0.04; 0.05]
Heterogeneity: $I^2 = 98\%$, $\tau^2 = 1.1485$, $p = 0$				
**Vector_species = Ixodes scapularis**				
[1010]Brown SM et al.(2015)	114	325	0.35	[0.30; 0.41]
[1055]Werden L et al.(2015)	74	1043	0.07	[0.06; 0.09]
[1059]Hutchinson ML et al.(2015)	60	1855	0.03	[0.02; 0.04]
[1061]Edwards MJ et al.(2015)	8	423	0.02	[0.01; 0.04]
[1108]Stromdahl E et al.(2014)	75	767	0.10	[0.08; 0.12]
[1109]Herrin BH et al.(2014)	234	712	0.33	[0.29; 0.36]
[1115]Hojgaard A et al.(2014)	7	185	0.04	[0.02; 0.08]
[1162]Prusinski MA et al.(2014)	283	3304	0.09	[0.08; 0.10]
[1163]Lee X et al.(2014)	15	378	0.04	[0.02; 0.06]
[1196]Aliota MT et al.(2014)	152	628	0.24	[0.21; 0.28]
[1199]Krakowetz CN et al.(2014)	169	12606	0.01	[0.01; 0.02]
[1305]Clark KL. et al.(2012)	3	236	0.01	[0.00; 0.04]
[1316]Roellig DM et al.(2012)	158	730	0.22	[0.19; 0.25]
[131]Schwartz S et al.(2022)	18	170	0.11	[0.06; 0.16]
[1369]Keesing F et al.(2012)	327	4640	0.07	[0.06; 0.08]
[1417]Lovrich SD et al.(2011)	48	402	0.12	[0.09; 0.16]
[1675]Swanson KI et al.(2007)	1	333	0.00	[0.00; 0.02]
[167]Scott JD et al.(2021)	1	111	0.01	[0.00; 0.05]
[1718]Michalski M et al.(2006)	17	167	0.10	[0.06; 0.16]
[1724]Steiner FE et al.(2006)	52	276	0.19	[0.14; 0.24]
[1778]Massung RF et al.(2005)	134	337	0.40	[0.34; 0.45]
[1808]Schulze TL et al.(2005)	9	141	0.06	[0.03; 0.12]
[180]Russell A et al.(2021)	2516	42401	0.06	[0.06; 0.06]
[1829]Holman MS et al.(2004)	11	301	0.04	[0.02; 0.06]
[183]Duplaix L et al.(2021)	210	9688	0.02	[0.02; 0.02]
[1859]Adelson ME et al.(2004)	2	107	0.02	[0.00; 0.07]
[1891]Courtney JW et al.(2003)	81	389	0.21	[0.17; 0.25]
[207]Ghosh P et al.(2021)	11	434	0.03	[0.01; 0.04]
[2092]Magnarelli LA et al.(1995)	25	170	0.15	[0.10; 0.21]
[2304]Foley et al.(2018)	1	425	0.00	[0.00; 0.01]
[2485]Cherepko et al.(2010)	27	773	0.03	[0.02; 0.05]
[2513]Berry et al.(2008)	27	886	0.03	[0.02; 0.04]
[254]Nelder MP et al.(2021)	74	17230	0.00	[0.00; 0.01]
[2559]Courtney et al.(2002)	81	389	0.21	[0.17; 0.25]
[2590]Schwartz et al.(1997)	100	273	0.37	[0.31; 0.43]
[262]Lehane A et al.(2021)	620	12315	0.05	[0.05; 0.05]
[278]Rounsville TF et al.(2021)	192	1849	0.10	[0.09; 0.12]
[413]Little EAH et al.(2020)	20	112	0.18	[0.11; 0.26]
[414]Westwood ML et al.(2020)	66	461	0.14	[0.11; 0.18]
[420]Pokutnaya D et al.(2020)	25	459	0.05	[0.04; 0.08]
[486]Xu G et al.(2019)	8	111	0.07	[0.03; 0.14]
[66]Foster E et al.(2022)	764	8262	0.09	[0.09; 0.10]
[687]Johnson TL et al.(2018)	78	1240	0.06	[0.05; 0.08]
[721]Nieto NC et al.(2018)	382	9113	0.04	[0.04; 0.05]
[753]Tokarz R et al.(2017)	27	318	0.08	[0.06; 0.12]
[756]Wroblewski D et al.(2017)	111	411	0.27	[0.23; 0.32]
[809]Murphy DS et al.(2017)	62	971	0.06	[0.05; 0.08]
[810]Johnson TL et al.(2017)	46	980	0.05	[0.03; 0.06]
Common effect model		139837	0.05	[0.05; 0.06]
Random effects model			0.07	[0.05; 0.09]
Heterogeneity: $I^2 = 99\%$, $\tau^2 = 1.4765$, $p = 0$				
**Vector_species = Haemaphysalis punctata**				
[1013]Palomar AM et al.(2015)	4	181	0.02	[0.01; 0.06]
[1049]Ebani VV et al.(2015)	66	180	0.37	[0.30; 0.44]
[1657]Barandika JF et al.(2008)	1	109	0.01	[0.00; 0.05]
Common effect model		470	0.15	[0.12; 0.19]
Random effects model			0.05	[0.01; 0.31]
Heterogeneity: $I^2 = 96\%$, $\tau^2 = 3.3495$, $p < 0.01$				
**Vector_species = Amblyomma variegatum**				
[1027]Teshale S et al.(2015)	5	203	0.02	[0.01; 0.06]
**Vector_species = Ixodes pacificus**				
[1072]Eshoo MW et al.(2015)	2	250	0.01	[0.00; 0.03]
[1467]Rejmanek D et al.(2011)	4	138	0.03	[0.01; 0.07]
[1722]Holden K et al.(2006)	5	168	0.03	[0.01; 0.07]
[182]Salkeld DJ et al.(2021)	19	1056	0.02	[0.01; 0.03]
[1879]Holden K et al.(2003)	48	6208	0.01	[0.01; 0.01]
[1998]Kramer VL et al.(1999)	10	195	0.05	[0.02; 0.09]
[2057]Barlough JE et al.(1997)	9	680	0.01	[0.01; 0.02]
[2552]Lane et al.(2004)	8	234	0.03	[0.01; 0.07]
[262]Lehane A et al.(2021)	8	501	0.02	[0.01; 0.03]
[344]Dykstra EA et al.(2020)	5	240	0.02	[0.01; 0.05]
[486]Xu G et al.(2019)	2	302	0.01	[0.00; 0.02]
[721]Nieto NC et al.(2018)	46	4048	0.01	[0.01; 0.02]
[880]Nieto NC et al.(2016)	106	710	0.15	[0.12; 0.18]
Common effect model		14730	0.02	[0.02; 0.02]
Random effects model			0.02	[0.01; 0.03]
Heterogeneity: $I^2 = 97\%$, $\tau^2 = 0.7308$, $p < 0.01$				
**Vector_species = Dermacentor reticulatus**				
[1082]Kurilshikov A et al.(2015)	1	364	0.00	[0.00; 0.02]
[113]Grochowska A et al.(2022)	3	261	0.01	[0.00; 0.03]
[1293]Krücken J et al.(2013)	1	1237	0.00	[0.00; 0.00]
[129]Panczuk A et al.(2022)	13	152	0.09	[0.05; 0.14]
[168]Grochowska A et al.(2021)	3	366	0.01	[0.00; 0.02]
[171]Levytska VA et al.(2021)	12	335	0.04	[0.02; 0.06]
[2215]Grochowska et al.(2022)	3	366	0.01	[0.00; 0.02]
[2278]Kartashov et al.(2019)	2	160	0.01	[0.00; 0.04]
[2340]Rybarova et al.(2017)	14	321	0.04	[0.02; 0.07]
[291]Michalski MM et al.(2021)	37	218	0.17	[0.12; 0.23]
[814]Zajac V et al.(2017)	7	486	0.01	[0.01; 0.03]
Common effect model		4266	0.02	[0.02; 0.03]
Random effects model			0.02	[0.01; 0.04]
Heterogeneity: $I^2 = 92\%$, $\tau^2 = 2.0255$, $p < 0.01$				
**Vector_species = Ixodes pavlovskyi**				
[1082]Kurilshikov A et al.(2015)	1	436	0.00	[0.00; 0.01]
[846]Rar V et al.(2017)	19	519	0.04	[0.02; 0.06]
Common effect model		955	0.02	[0.01; 0.03]
Random effects model			0.01	[0.00; 0.08]
Heterogeneity: $I^2 = 87\%$, $\tau^2 = 1.7581$, $p < 0.01$				
**Vector_species = Ixodes persulcatus**				
[1151]Javkhlan G et al.(2014)	14	214	0.07	[0.04; 0.11]
[1157]Masuzawa T et al.(2014)	8	129	0.06	[0.03; 0.12]
[1319]Ybañez AP et al.(2012)	29	134	0.22	[0.15; 0.30]
[1398]Jiang JF et al.(2011)	43	686	0.06	[0.05; 0.08]
[1433]Murase Y et al.(2011)	20	281	0.07	[0.04; 0.11]
[1478]Rar VA et al.(2010)	63	2365	0.03	[0.02; 0.03]
[1667]Eremeeva ME et al.(2007)	54	429	0.13	[0.10; 0.16]
[1888]Kim CM et al.(2003)	36	156	0.23	[0.17; 0.30]
[2278]Kartashov et al.(2019)	16	530	0.03	[0.02; 0.05]
[2448]Li et al.(2012)	91	2172	0.04	[0.03; 0.05]
[2503]Rar et al.(2008)	14	834	0.02	[0.01; 0.03]
[2550]Zhao et al.(2005)	31	454	0.07	[0.05; 0.10]
[29]Lagunova EK et al.(2022)	47	342	0.14	[0.10; 0.18]
[846]Rar V et al.(2017)	21	295	0.07	[0.04; 0.11]
[868]Karnath C et al.(2016)	17	262	0.06	[0.04; 0.10]
[979]Liu X et al.(2015)	19	204	0.09	[0.06; 0.14]
Common effect model		9487	0.06	[0.05; 0.06]
Random effects model			0.07	[0.05; 0.10]
Heterogeneity: $I^2 = 94\%$, $\tau^2 = 0.5301$, $p < 0.01$				
**Vector_species = Ixodes hexagonus**				
[1156]Schreiber C et al.(2014)	6	152	0.04	[0.01; 0.08]
[1285]Claerebout E et al.(2013)	37	679	0.05	[0.04; 0.07]
[1293]Krücken J et al.(2013)	5	151	0.03	[0.01; 0.08]
[258]Keyte S et al.(2021)	6	292	0.02	[0.01; 0.04]
[594]Duplan F et al.(2018)	1	540	0.00	[0.00; 0.01]
Common effect model		1814	0.03	[0.02; 0.04]
Random effects model			0.02	[0.01; 0.05]
Heterogeneity: $I^2 = 75\%$, $\tau^2 = 0.8865$, $p < 0.01$				
**Vector_species = Amblyomma americanum**				
[1167]Lee S et al.(2014)	2	821	0.00	[0.00; 0.01]
[1305]Clark KL. et al.(2012)	6	223	0.03	[0.01; 0.06]
[721]Nieto NC et al.(2018)	6	2078	0.00	[0.00; 0.01]
Common effect model		3122	0.00	[0.00; 0.01]
Random effects model			0.01	[0.00; 0.02]
Heterogeneity: $I^2 = 89\%$, $\tau^2 = 1.0394$, $p < 0.01$				
**Vector_species = Haemaphysalis leporispalustris**				
[1171]Schmitz KM et al.(2014)	4	375	0.01	[0.00; 0.03]
**Vector_species = Dermacentor silvarum**				
[1218]Dong T et al.(2013)	40	122	0.33	[0.25; 0.42]
[1398]Jiang JF et al.(2011)	13	515	0.03	[0.01; 0.04]
[1747]Cao WC et al.(2006)	2	111	0.02	[0.00; 0.06]
Common effect model		748	0.07	[0.06; 0.09]
Random effects model			0.06	[0.01; 0.26]
Heterogeneity: $I^2 = 98\%$, $\tau^2 = 2.2276$, $p < 0.01$				
**Vector_species = Haemaphysalis longicornis**				
[1218]Dong T et al.(2013)	30	128	0.23	[0.16; 0.32]
[1252]Kang SW et al.(2013)	31	576	0.05	[0.04; 0.08]
[1430]Zhang L et al.(2011)	30	170	0.18	[0.12; 0.24]
[219]Yan Y et al.(2021)	4	371	0.01	[0.00; 0.03]
[221]Seo HJ et al.(2021)	5	603	0.01	[0.00; 0.02]
[2339]Peng et al.(2017)	10	660	0.02	[0.01; 0.03]
[2448]Li et al.(2012)	14	321	0.04	[0.02; 0.07]
[2502]Moon et al.(2009)	27	1392	0.02	[0.01; 0.03]
[444]Seo MG et al.(2020)	11	576	0.02	[0.01; 0.03]
[498]Su H et al.(2019)	6	483	0.01	[0.00; 0.03]
[53]Price KJ et al.(2022)	4	624	0.01	[0.00; 0.02]
[595]Qin XR et al.(2018)	3	3025	0.00	[0.00; 0.00]
[791]Liu XY et al.(2017)	28	500	0.06	[0.04; 0.08]
[816]Zhang H et al.(2017)	11	841	0.01	[0.01; 0.02]
[944]Kang JG et al.(2016)	38	228	0.17	[0.12; 0.22]
[947]Kang JG et al.(2016)	12	239	0.05	[0.03; 0.09]
Common effect model		10737	0.02	[0.02; 0.03]
Random effects model			0.03	[0.01; 0.05]
Heterogeneity: $I^2 = 96\%$, $\tau^2 = 1.9658$, $p < 0.01$				
**Vector_species = Rhipicephalus sanguineus**				
[1249]Santos HA et al.(2013)	5	388	0.01	[0.00; 0.03]
[2427]Li Yan et al.(2013)	15	192	0.08	[0.04; 0.13]
[332]Manoj RRS et al.(2020)	1	215	0.00	[0.00; 0.03]
Common effect model		795	0.03	[0.02; 0.04]
Random effects model			0.02	[0.00; 0.07]
Heterogeneity: $I^2 = 89\%$, $\tau^2 = 1.2445$, $p < 0.01$				
**Vector_species = Rhipicephalus turanicus**				
[127]Saratsis A et al.(2022)	31	169	0.18	[0.13; 0.25]
[81]Guo J et al.(2022)	22	436	0.05	[0.03; 0.08]
Common effect model		605	0.09	[0.07; 0.11]
Random effects model			0.10	[0.04; 0.23]
Heterogeneity: $I^2 = 96\%$, $\tau^2 = 0.4810$, $p < 0.01$				
**Vector_species = Ixodes dammini**				
[1454]Telford Iii SR et al.(2011)	3	760	0.00	[0.00; 0.01]
**Vector_species = Amblyomma flavomaculatum**				
[1497]Nowak M et al.(2010)	2	125	0.02	[0.00; 0.06]
**Vector_species = Ixodes ventalloi**				
[1540]Santos AS et al.(2009)	3	144	0.02	[0.00; 0.06]
**Vector_species = Hyalomma marginatum**				
[1577]Fernández−Soto P et al.(2009)	2	515	0.00	[0.00; 0.01]
[2553]de la Fuente et al.(2004)	52	120	0.43	[0.34; 0.53]
Common effect model		635	0.09	[0.07; 0.11]
Random effects model			0.05	[0.00; 0.71]
Heterogeneity: $I^2 = 98\%$, $\tau^2 = 7.2872$, $p < 0.01$				
**Vector_species = Dermacentor marginatus**				
[1613]Toledo A et al.(2009)	4	265	0.02	[0.00; 0.04]
[1820]Merino FJ et al.(2005)	4	103	0.04	[0.01; 0.10]
[2553]de la Fuente et al.(2004)	54	102	0.53	[0.43; 0.63]
Common effect model		470	0.13	[0.10; 0.17]
Random effects model			0.08	[0.01; 0.43]
Heterogeneity: $I^2 = 98\%$, $\tau^2 = 3.5263$, $p < 0.01$				
**Vector_species = Hyalomma lusitanicum**				
[1613]Toledo A et al.(2009)	23	795	0.03	[0.02; 0.04]
**Vector_species = Dermacentor occidentalis**				
[1879]Holden K et al.(2003)	4	1059	0.00	[0.00; 0.01]
[721]Nieto NC et al.(2018)	1	264	0.00	[0.00; 0.02]
Common effect model		1323	0.00	[0.00; 0.01]
Random effects model			0.00	[0.00; 0.01]
Heterogeneity: $I^2 = 0\%$, $\tau^2 = 0$, $p = 1.00$				
**Vector_species = Ixodes nipponensis**				
[2267]Tang et al.(2020)	1	380	0.00	[0.00; 0.01]
**Vector_species = Haemaphysalis concinna**				
[2353]Yang et al.(2016)	1	106	0.01	[0.00; 0.05]
**Vector_species = Haemaphysalis qinghaiensis**				
[2427]Li Yan et al.(2013)	27	135	0.20	[0.14; 0.28]
[503]Han R et al.(2019)	22	249	0.09	[0.06; 0.13]
Common effect model		384	0.13	[0.10; 0.16]
Random effects model			0.13	[0.07; 0.23]
Heterogeneity: $I^2 = 89\%$, $\tau^2 = 0.1783$, $p < 0.01$				
**Vector_species = Rhipicephalus microplus**				
[2427]Li Yan et al.(2013)	7	216	0.03	[0.01; 0.07]
**Vector_species = Rhipicephalus pumilio**				
[2443]Liu et al.(2012)	18	519	0.03	[0.02; 0.05]
**Vector_species = Hyalomma aegyptium**				
[33]Rjeibi MR et al.(2022)	26	191	0.14	[0.09; 0.19]
**Vector_species = Dermacentor andersoni**				
[423]Francis L et al.(2020)	3	203	0.01	[0.00; 0.04]
[721]Nieto NC et al.(2018)	1	132	0.01	[0.00; 0.04]
Common effect model		335	0.01	[0.00; 0.03]
Random effects model			0.01	[0.00; 0.03]
Heterogeneity: $I^2 = 0\%$, $\tau^2 = 0$, $p = 0.56$				
**Vector_species = Ixodes simplex**				
[513]Hornok S et al.(2019)	2	138	0.01	[0.00; 0.05]
**Vector_species = Dermacentor nuttalli**				
[702]Narankhajid M et al.(2018)	8	110	0.07	[0.03; 0.14]
**Vector_species = Dermacentor variabilis**				
[721]Nieto NC et al.(2018)	28	5853	0.00	[0.00; 0.01]
**Vector_species = Haemaphysalis formosensis**				
[9]Gaowa et al.(2013)	25	715	0.03	[0.02; 0.05]
**Common effect model**		**397818**	**0.05**	**[0.05; 0.05]**
**Random effects model**			**0.04**	**[0.04; 0.05]**

0.1 0.4 0.7

Heterogeneity: $I^2 = 98\%$, $\tau^2 = 1.5235$, $p = 0$
Test for subgroup differences (common effect): $\chi^2_{32} = 1210.60$, df = 32 ($p < 0.01$)
Test for subgroup differences (random effects): $\chi^2_{32} = 314.35$, df = 32 ($p < 0.01$)
