# Supplementary material for: The global distribution and risk prediction of Anaplasmataceae species: a systematic review and geospatial modelling analysis
Source: eBioMedicine. 2025 Apr 23;115:105722. doi: 10.1016/j.ebiom.2025.105722 (PMC12051633; doi:10.1016/j.ebiom.2025.105722)
Supplement: Appendix4_E. canis_forest plot [file mmc8.pdf]

Study	Events	Total	Proportion	95%−CI
**Vector_species = Rhipicephalus sanguineus**				
[1002]Lee GK et al.(2015)	4	175	0.02	[0.01; 0.06]
[128]Backus L et al.(2022)	18	155	0.12	[0.07; 0.18]
[1334]Ybañez AP et al.(2012)	8	146	0.05	[0.02; 0.11]
[1444]Satta G et al.(2011)	1	209	0.00	[0.00; 0.03]
[1458]Foongladda S et al.(2011)	10	304	0.03	[0.02; 0.06]
[1699]Aguiar DM et al.(2007)	20	489	0.04	[0.03; 0.06]
[2238]Celik et al.(2021)	38	250	0.15	[0.11; 0.20]
[2244]Ceylan et al.(2021)	38	250	0.15	[0.11; 0.20]
[2444]Parto Ferreira de Almeida et al.(2012)	38	380	0.10	[0.07; 0.13]
[332]Manoj RRS et al.(2020)	27	215	0.13	[0.08; 0.18]
[379]Mengfan Q et al.(2020)	108	709	0.15	[0.13; 0.18]
[391]Nguyen VL et al.(2020)	4	130	0.03	[0.01; 0.08]
[41]Lineberry MW et al.(2022)	1	375	0.00	[0.00; 0.01]
[569]Nguyen VL et al.(2019)	1	288	0.00	[0.00; 0.02]
[610]Low VL et al.(2018)	1	140	0.01	[0.00; 0.04]
[667]Galay RL et al.(2018)	5	157	0.03	[0.01; 0.07]
[672]Ipek NDS et al.(2018)	378	3325	0.11	[0.10; 0.12]
[763]Zhang J et al.(2017)	50	3624	0.01	[0.01; 0.02]
[836]Yuasa Y et al.(2017)	3	306	0.01	[0.00; 0.03]
[905]Campos−Calderón L et al.(2016)	42	161	0.26	[0.19; 0.34]
[958]Çetinkaya H et al.(2016)	20	284	0.07	[0.04; 0.11]
[978]Ionita M et al.(2016)	19	120	0.16	[0.10; 0.24]
Common effect model		12192	0.07	[0.06; 0.07]
Random effects model			0.04	[0.03; 0.08]
Heterogeneity: $I^2 = 95\%$, $\tau^2 = 1.6484$, $p < 0.01$				
**Vector_species = Rhipicephalus bursa**				
[1088]Aktas M. et al.(2014)	1	238	0.00	[0.00; 0.02]
[794]Dahmani M et al.(2017)	1	118	0.01	[0.00; 0.05]
Common effect model		356	0.01	[0.00; 0.02]
Random effects model			0.01	[0.00; 0.02]
Heterogeneity: $I^2 = 0\%$, $\tau^2 = 0$, $p = 0.62$				
**Vector_species = Dermacentor marginatus**				
[1251]Hornok S et al.(2013)	4	2011	0.00	[0.00; 0.01]
**Vector_species = Ixodes canisuga**				
[1251]Hornok S et al.(2013)	3	2011	0.00	[0.00; 0.00]
**Vector_species = Haemaphysalis longicornis**				
[1252]Kang SW et al.(2013)	127	550	0.23	[0.20; 0.27]
[175]Seo MG et al.(2021)	1	245	0.00	[0.00; 0.02]
[1773]Kim CM et al.(2006)	17	1450	0.01	[0.01; 0.02]
Common effect model		2245	0.06	[0.06; 0.08]
Random effects model			0.02	[0.00; 0.18]
Heterogeneity: $I^2 = 99\%$, $\tau^2 = 3.4333$, $p < 0.01$				
**Vector_species = Ixodes ricinus**				
[1277]Coipan EC et al.(2013)	5	5343	0.00	[0.00; 0.00]
[1740]Wielinga PR et al.(2006)	3	1099	0.00	[0.00; 0.01]
[44]Rataud A et al.(2022)	1	1039	0.00	[0.00; 0.01]
Common effect model		7481	0.00	[0.00; 0.00]
Random effects model			0.00	[0.00; 0.00]
Heterogeneity: $I^2 = 12\%$, $\tau^2 = 0$, $p = 0.32$				
**Vector_species = Rhipicephalus turanicus**				
[127]Saratsis A et al.(2022)	1	169	0.01	[0.00; 0.03]
**Vector_species = Ixodes scapularis**				
[2092]Magnarelli LA et al.(1995)	40	231	0.17	[0.13; 0.23]
**Vector_species = Ixodes dammini**				
[2119]Magnarelli LA et al.(1991)	60	609	0.10	[0.08; 0.12]
**Vector_species = Rhipicephalus microplus**				
[43]Lu M et al.(2022)	17	276	0.06	[0.04; 0.10]
[62]Lu M et al.(2022)	71	196	0.36	[0.29; 0.43]
[65]Lu M et al.(2022)	16	138	0.12	[0.07; 0.18]
[765]Lu M et al.(2017)	1	354	0.00	[0.00; 0.02]
Common effect model		964	0.11	[0.09; 0.13]
Random effects model			0.06	[0.01; 0.30]
Heterogeneity: $I^2 = 96\%$, $\tau^2 = 3.3610$, $p < 0.01$				
**Vector_species = Rhipicephalus pravus**				
[786]Omondi D et al.(2017)	13	195	0.07	[0.04; 0.11]
**Common effect model**		**28464**	**0.04**	**[0.04; 0.05]**
**Random effects model**			**0.03**	**[0.01; 0.05]**

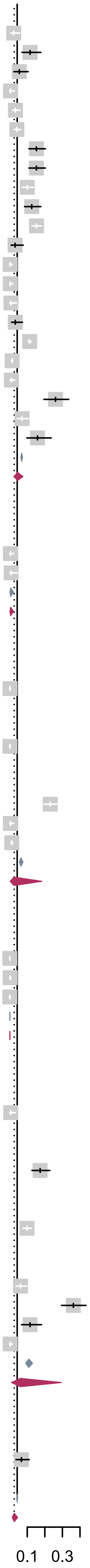

Heterogeneity: $I^2 = 96\%$, $\tau^2 = 3.3012$, $p < 0.01$
Test for subgroup differences (common effect): $\chi^2_{10} = 337.77$, df = 10 ($p < 0.01$)
Test for subgroup differences (random effects): $\chi^2_{10} = 313.53$, df = 10 ($p < 0.01$)
